# Global burden and cross-country inequality of infectious skin diseases in children: the Global Burden of Disease Study 2021

**DOI:** 10.1093/inthealth/ihaf109

**Published:** 2025-10-14

**Authors:** Lang Rao, Shu-Xia Chang, Qiao Peng, Li-Ming Xiang, Wei-Sen Zhao, Pan Ran, Long Zhao, Yong-Hong Lu, Dong-Ze Wu, Yong-Feng Chen

**Affiliations:** Department of Dermatology, Dermatology Hospital, Southern Medical University, Guangzhou, Guangdong Province 510091, China; Department of Dermatology, Chengdu Second People’s Hospital, Chengdu, Sichuan Province 610017, China; The First School of Clinical Medicine, Southern Medical University, Guangzhou, Guangdong Province 510515, China; Department of Dermatology and Venereology, Shenzhen Longgang Center for Chronic Disease Control, Shenzhen, Guangdong Province 518172, China; Department of Dermatology, Chengdu Second People’s Hospital, Chengdu, Sichuan Province 610017, China; Department of Dermatology, Chengdu Second People’s Hospital, Chengdu, Sichuan Province 610017, China; Department of Dermatology and Venereology, Shenzhen Longgang Center for Chronic Disease Control, Shenzhen, Guangdong Province 518172, China; School of Bioscience and Technology, Chengdu Medical College, Chengdu, Sichuan Province 610500, China; School of Bioscience and Technology, Chengdu Medical College, Chengdu, Sichuan Province 610500, China; Department of Dermatology, Chengdu Second People’s Hospital, Chengdu, Sichuan Province 610017, China; Department of Rheumatology and Immunology, Sichuan Provincial People’s Hospital, School of Medicine, University of Electronic Science and Technology of China, Chengdu, Sichuan Province 610072, China; Department of Dermatology, Dermatology Hospital, Southern Medical University, Guangzhou, Guangdong Province 510091, China

**Keywords:** children, global burden of disease study, health inequality, infectious skin diseases, trend

## Abstract

**Background:**

Infectious skin diseases (ISDs) in children require greater attention; this study aims to explore their global burden from 1990 to 2021.

**Methods:**

We obtained data from the Global Burden of Disease Study 2021 and present information on the number, rates of incidence and disability-adjusted life years (DALYs) for ISDs among children, including bacterial, fungal and viral skin diseases.

**Results:**

In 2021, an estimated 685 367 728 new cases of ISDs in children were identified globally. Among these, bacterial, fungal and viral skin diseases accounted for 37.81%, 55.70% and 6.49%, respectively. From 1990 to 2021, a significant increase was observed in the incidence of ISDs, but a notable decrease was noted in the rate of DALYs. In 2021, low Socio-Demographic Index (SDI) regions had the highest incidence and DALYs rates for bacterial and fungal skin diseases; high-SDI regions exhibited the highest rates for viral skin diseases. India had the highest incidence rate of ISDs, while Ethiopia revealed the highest DALYs rate. Absolute inequality analyses showed the slope index of inequality narrowed from 1990 to 2021.

**Conclusions:**

The incidence of ISDs among children is rising globally; children in low- and middle-income countries (LMICs) bear a disproportionately higher disease burden. Intervention strategies are required to reduce the disease burden, particularly in LMICs.

## Introduction

Infectious skin diseases (ISDs) are common dermatological illnesses, ranging in severity from benign to life-threatening.^1^ Over the past three decades, there has been an obvious increase in ISDs worldwide.^1^ The types of ISDs vary based on the pathogen involved, which are mainly divided into bacterial, fungal and viral skin diseases.^1^ Cellulitis and pyoderma are the most common types of bacterial skin diseases, usually caused by Group A *Streptococcus* (GAS) and *Staphylococcus aureus*.^2^ Recent analyses have indicated an increasing incidence of these diseases, particularly with the emergence of methicillin-resistant *Staphylococcus aureus* (MRSA), thereby contributing to a higher prevalence and economic burdens.^2,3^ More than 40 different species of dermatophytes have been implicated in fungal skin diseases; however, there are great disparities in the pathogens susceptible to infection among different regions, age groups and gender.^4^ For instance, tinea nigra is more common in tropical areas; tinea capitis usually affects children aged 3–7 y; and *Candida* spp. are more abundant in females.^5^ Viral skin infections are mainly caused by papillomaviruses and the molluscum contagiosum (MC) virus. Although many viral infections are self-limited, some produce chronic or even lifelong infections, causing a substantial healthcare burden and adversely affecting quality of life.^6^

Skin is the human body’s largest organ, which not only acts as a physical barrier to prevent the invasion of foreign pathogens but provides a home to the balanced commensal microbiota, including bacteria, fungi, viruses and mites.^7^ The immune system has evolved closely with resident microorganisms on the skin to allow the maintenance of commensal partners and the elimination of pathogens. Disruptions in the balance on either side of this equation can result in skin disorders or infections. Overall, the immune response plays a paramount role in ISDs, but the innate and adaptive immune systems develop differently with age.

Immature immune systems in children exhibit a decreased response to chemokines and decreased anti-infective activity compared with those of adults.^8^ Hence, ISDs in children are more frequent and generally more severe than in adults, especially in neonates.^9^ According to the Global Burden of Disease (GBD) study, the incidence of bacterial, fungal and viral skin diseases was higher in children than in young and middle-aged adults (20–54 y) in 2021. ISDs may affect not only physical health but mental health and quality of life, placing a heavy burden on children and their families, as well as on national healthcare systems. Despite the growing concerns about ISDs in children, little is known about how their incidence and disability-adjusted life years (DALYs) have changed within this population on a national scale. In this study, we first used the GBD database to focus on ISDs in children and aim to estimate their patterns, trends and inequality.

## Methods

### Data sources and collection

We performed a secondary analysis of data on ISDs among children from GBD 2021. This study follows the Guidelines for Accurate and Transparent Health Estimates Reporting Guidelines for cross-sectional studies. The data sources can be found through Sources Tool (https://ghdx.healthdata.org/gbd-2021/sources) from the Institute for Health Metrics and Evaluation website. The WHO defined children as aged 0–14 y.^10^ We extracted data regarding the incidence and DALYs of ISDs among children through the GBD Results Tool (https://vizhub.healthdata.org/gbd-results/).

### Definitions of infectious skin diseases

ISDs encompass bacterial, fungal and viral conditions. In the GBD 2021 study, cellulitis and pyoderma are identified as the two defined bacterial skin diseases. Fungal skin diseases consist of tinea capitis and a residual group of ‘any’ other fungal diseases, excluding onychomycosis. Viral skin diseases consist of human papillomavirus (HPV) infection (viral warts) and MC. The International Classification of Diseases codes assigned for ISDs in the GBD 2021 study are listed in Table S1.

### Socio-Demographic Index

GBD 2021 also calculated a Socio-Demographic Index (SDI) for each country, which represents a comprehensive development status that correlates with health outcomes. Briefly, the SDI is the geometric mean of the 0 to 1 index of the total fertility rate for those aged <25 y, education for those aged ≥15 y and lag-distributed income per capita.^11,12^

### Cross-country inequality analysis

Number and age-specific rate of incidence and DALYs were extracted for analysis of inequality. Slope index of inequality and concentration index were used for quantifying the SDI-related inequality of ISDs burden across countries. The slope index of inequality was calculated by regressing the national DALYs rates for countries ranked by SDI. Concentration index was computed by fitting a Lorenz curve to the cumulative relative distributions of the population ranked by SDI and the corresponding national burden in DALYs, calculated as twice the area between the 45° diagonal line (line of equality) and the Lorenz curve.

### Statistical analysis

To estimate the global trends in incidence and DALYs for ISDs, we calculated the age-specific rates and their average annual percentage change (AAPC) using linear regression. Rates are presented per 100 000 population, along with 95% uncertainty interval (UI) according to the GBD algorithm. We calculated the AAPCs from 1990 to 2021 and three segment ranges (1990–1999, 2000–2009 and 2010–2021). The 95% CI of AAPC was determined through linear modeling. An increasing or decreasing trend was identified if both the AAPC and its 95% CI were above or below zero, respectively. Otherwise, the rates were considered stable. Joinpoint regression analysis was used to identify the year with the most significant changes. Spearman correlation analysis was used to assess the correlation between age-specific rates and SDI. All statistical analyses were conducted using R Studio, version 4.1.2 (R Project for Statistical Computing) and the Joinpoint Regression Program (version 4.9.0.0). All p values were two-sided and p*<*0.05 was considered statistically significant.

## Results

### Global trends by cause

**Figure 1.**
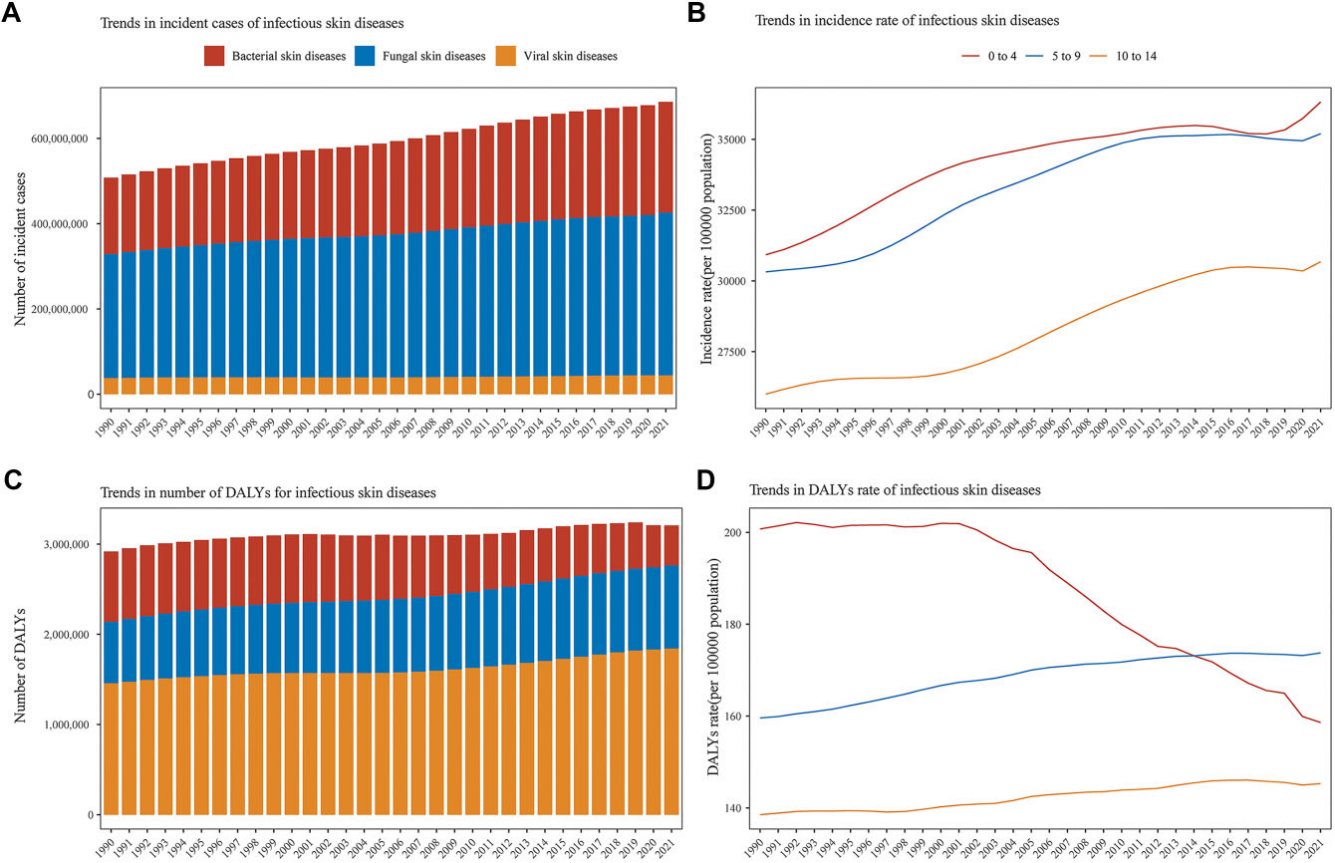
Trends in infectious skin diseases incidence and DALYs among children from 1990 to 2021. (A) Number of incident cases of infectious skin diseases from 1990 to 2021, (B) incidence rate of different age groups in infectious skin diseases from 1990 to 2021, (C) number of DALYs in infectious skin diseases from 1990 to 2021 and (D) DALYs rate of different age groups in infectious skin diseases from 1990 to 2021. DALYs: disability-adjusted life years.

Globally, 685 367 728 (95% UI, 603 280 570–786 790 877) new ISDs in children were identified in 2021, most of which were fungal skin diseases accounting for 55.70%, followed by bacterial skin diseases at 37.81% and the least were viral skin diseases (6.49%) (Figure 1A). From 1990 to 2021, the overall incidence rate of ISDs in children exhibited an upward trend. The incidence increased from 1990 to 1999 (AAPC=0.58), increased at a greater rate from 2000 to 2009 (AAPC=0.73) and continued to increase at a slower rate from 2010 to 2021 (AAPC=0.22). Joinpoint regression analysis identified a substantial change in incidence of ISDs in 1996, 2009, 2012, 2015 and 2019. From 1990 to 2021, bacterial skin diseases presented the largest increase by 45% (AAPC=0.72), followed by fungal skin diseases (31%) and viral skin diseases (18%) (Table 1).

**Table 1.** The incident cases and incidence rate of infectious skin diseases in 1990 and 2021, and their average annual percentage changes from 1990 to 2021

	1990		2021					
*Rate per 100 000 AAPC (95% CI)*	Incident cases	Incidence rate	Incident cases	Incidence rate	1990–2021 AAPC	1990–1999 AAPC	2000–2009 AAPC	2010–2021 AAPC
Overall	507 861 851 (445 301 651–580 853 609)	29 201.71 (25 604.54–33 398.68)	685 367 728 (603 280 570–786 790 877)	34 066.35 (29 986.19–39 107.61)	0.49 (0.48 to 0.51)	0.58 (0.57 to 0.59)	0.73 (0.72 to 0.73)	0.22 (0.19 to 0.25)
Male	269 569 515 (235 013 838–309 567 497)	30 170.34 (26 302.86–34 646.93)	362 924 139 (317 737 123–418 742 625)	34 958.21 (30 605.63–40 334.86)	0.48 (0.46 to 0.5)	0.58 (0.56 to 0.61)	0.71 (0.69 to 0.73)	0.17 (0.11 to 0.23)
Female	238 292 336 (210 599 504–271 561 798)	28 178.28 (24 903.58–32 112.43)	322 443 589 (284 992 171–367 841 867)	33 115.44 (29 269.12–37 777.91)	0.51 (0.5 to 0.52)	0.54 (0.53 to 0.55)	0.74 (0.73 to 0.75)	0.26 (0.22 to 0.29)
**Cause**								
Bacterial skin diseases	179 189 301 (170 362 311–188 569 867)	10 303.26 (9795.72–10 842.64)	259 111 300 (246 067 503–273 425 523)	12 879.18 (12 230.84–13 590.68)	0.72 (0.69–0.74)	0.7 (0.64 to 0.77)	1.08 (1.05 to 1.11)	0.39 (0.36 to 0.42)
Fungal skin diseases	290 930 380 (239 071 081–352 548 040)	16 728.30 (13 746.42–20 271.27)	381 786 200 (314 958 733–466 426 730)	18 976.77 (15 655.09–23 183.84)	0.4 (0.37–0.44)	0.58 (0.57 to 0.59)	0.58 (0.57 to 0.59)	0.09 (−0.01 to 0.19)
Viral skin diseases	37 742 170 (35 868 260–39 735 703)	2170.15 (2062.40–2284.78)	44 470 228 (42 254 333–46 938 624)	2210.40 (2100.26–2333.09)	0.06 (0.05–0.07)	−0.09 (−0.11 to −0.07)	0.11 (0.09 to 0.13)	0.15 (0.14 to 0.16)
**SDI region**								
High SDI	31 687 582 (28 453 835–35 477 628)	17 053.96 (15 313.59–19 093.73)	28 793 476 (25 909 787–32 157 574)	16 688.34 (15 016.99–18 638.13)	−0.07 (−0.08 to −0.06)	−0.3 (−0.31 to −0.29)	−0.1 (−0.12 to −0.08)	0.15 (0.14 to 0.17)
High-middle SDI	47 404 468 (42 045 359–53 252 339)	17 324.73 (15 366.16–19 461.93)	38 964 830 (34 461 888–43 956 260)	16 875.9 (14 925.65–19 037.72)	−0.09 (−0.1 to −0.07)	−0.09 (−0.11 to −0.08)	0.08 (0.07 to 0.1)	−0.24 (−0.27 to −0.22)
Middle SDI	125 699 381 (109 350 871–143 233 825)	21 776.91 (18 944.6–24 814.69)	131 112 812 (114 619 861–148 000 098)	23 129.63 (20 220.1–26 108.71)	0.19 (0.18 to 0.2)	0.26 (0.25 to 0.28)	0.4 (0.39 to 0.41)	−0.06 (−0.08 to −0.03)
Low-middle SDI	168 273 129 (145 717 512–195 826 087)	35 642.62 (30 865.02–41 478.72)	214 574 752 (187 885 013–246 747 645)	37 005.78 (32 402.84–42 554.34)	0.12 (0.1 to 0.14)	0.15 (0.14 to 0.16)	0.11 (0.06 to 0.16)	0.09 (0.06 to 0.13)
Low SDI	134 483 127 (117 266 143–157 615 012)	58 748.46 (51 227.28–68 853.54)	271 553 585 (236 502 654–316 176 408)	59 004.45 (51 388.42–68 700.31)	0.01 (−0.04 to 0.05)	0.12 (0.06 to 0.17)	−0.09 (−0.19 to 0.01)	−0.01 (−0.09 to 0.06)
**GBD region**								
Andean Latin America	3 987 483 (3 394 767–4 633 644)	26 848.06 (22 857.26–31 198.72)	4 711 023 (4 009 173–5 479 101)	26 035.24 (22 156.5–30 279.98)	0.1 (−0.11 to −0.09)	−0.17 (−0.18 to −0.16)	−0.08 (−0.09 to −0.06)	−0.07 (−0.08 to −0.06)
Australasia	1 370 159 (1 230 863–1 541 091)	29 877.26 (26 839.83–33 604.57)	1 693 260 (1 511 552–1 911 094)	29 544.87 (26 374.33–33 345.74)	0.03 (−0.04 to −0.03)	−0.08 (−0.09 to −0.08)	0 (−0.02 to 0.02)	−0.03 (−0.04 to −0.02)
Caribbean	2 499 314 (2 156 183–2 892 421)	21 899.94 (18 893.29–25 344.49)	2 512 667 (2 172 959–2 919 146)	21 839.59 (18 886.92–25 372.63)	0.01 (−0.02 to 0)	−0.07 (−0.1 to −0.05)	0 (−0.01 to 0.01)	0.03 (0.01 to 0.05)
Central Asia	4 292 403 (3 788 718–4 912 975)	17 175.65 (15 160.2–19 658.81)	4 795 900 (4 231 754–5 496 153)	17 328.86 (15 290.45–19 859.06)	0.03 (0.02 to 0.03)	−0.05 (−0.05 to −0.04)	0.06 (0.06 to 0.07)	0.06 (0.06 to 0.07)
Central Europe	4 646 753 (4 038 524–5 339 658)	15 760.46 (13 697.52–18 110.59)	2 784 864 (2 424 202–3 203 173)	15 732.62 (13 695.12–18 095.78)	0.01 (−0.01 to 0)	−0.02 (−0.03 to −0.02)	0.02 (0.01 to 0.02)	−0.02 (−0.03 to −0.01)
Central Latin America	10 948 768 (9 493 683–12 659 395)	17 006.15 (14 746.05–19 663.18)	10 736 164 (9 283 535–12 431 313)	16 911.35 (14 623.2–19 581.51)	0.02 (−0.03 to −0.01)	−0.19 (−0.2 to −0.19)	−0.16 (−0.17 to −0.15)	0.27 (0.25 to 0.29)
Central sub-Saharan Africa	16 700 002 (14 014 756–20 402 606)	66 011.5 (55 397.3–80 647.1)	36 800 082 (30 737 295–44 553 125)	62 711.42 (52 379.76–75 923.47)	0.15 (−0.27 to −0.04)	0.12 (0.05 to 0.19)	0 (−0.23 to 0.23)	−0.6 (−0.85 to −0.35)
East Asia	45 930 705 (39 807 597–52 734 775)	13 925.41 (12 068.99–15 988.29)	34 564 248 (29 984 954–39 288 480)	12 928.36 (11 215.53–14 695.41)	0.24 (−0.26 to −0.22)	−0.09 (−0.13 to −0.05)	−0.52 (−0.56 to −0.48)	−0.13 (−0.15 to −0.11)
Eastern Europe	11 206 695 (9 941 822–12 643 694)	21 776.65 (19 318.77–24 569)	7 702 884 (6 791 793–8 727 037)	21 732.53 (19 162.02–24 622.02)	0.01 (−0.02 to 0)	−0.15 (−0.17 to −0.13)	0.13 (0.12 to 0.15)	−0.02 (−0.03 to −0.01)
Eastern sub-Saharan Africa	68 984 646 (60 277 525–80 556 567)	76 166.6 (66 552.98–88 943.27)	132 082 021 (115 279 171–153 656 637)	74 023.94 (64 606.96–86 115.2)	0.11 (−0.14 to −0.07)	0.05 (0 to 0.1)	−0.18 (−0.21 to −0.16)	−0.16 (−0.24 to −0.08)
High-income Asia Pacific	6 264 517 (5 482 054–7 173 195)	17 797.16 (15 574.22–20 378.66)	3 939 506 (3 448 493–4 528 706)	17 567.05 (15 377.52–20 194.41)	0.04 (−0.05 to −0.03)	−0.15 (−0.17 to −0.13)	−0.03 (−0.05 to −0.01)	0.04 (0.03 to 0.05)
High-income North America	7 260 047 (6 698 118–7 823 284)	11 770.95 (10 859.88–12 684.15)	7 734 029 (7 130 078–8 327 200)	11 786.28 (10 865.89–12 690.24)	0.04 (−0.04 to 0.04)	−0.51 (−0.57 to −0.45)	0.09 (0.03 to 0.16)	0.36 (0.3 to 0.43)
North Africa and Middle East	22 730 746 (20 830 001–24 763 583)	16 180.15 (14 827.16–17 627.16)	28 268 015 (25 949 521–30 797 818)	15 419.87 (14 155.16–16 799.85)	0.16 (−0.17 to −0.15)	−0.29 (−0.31 to −0.27)	−0.06 (−0.07 to −0.06)	−0.14 (−0.16 to −0.13)
Oceania	445 694 (383 934–514 345)	16 631.22 (14 326.62–19 192.94)	847 274 (730 178–976 434)	16 675.77 (14 371.12–19 217.87)	0.01 (−0.01 to 0.02)	0.01 (−0.01 to 0.04)	0.03 (0 to 0.05)	−0.01 (−0.03 to 0.01)
South Asia	166 993 038 (143 422 212–197 720 532)	38 534.32 (33 095.25–45 624.81)	187 969 174 (163 481 514–218 175 144)	37 073.21 (32 243.51–43 030.75)	0.13 (−0.14 to −0.12)	−0.08 (−0.09 to −0.06)	−0.18 (−0.19 to −0.17)	−0.13 (−0.14 to −0.11)
Southeast Asia	34 699 399 (29 630 519–40 519 606)	20 322.05 (17 353.41–23 730.71)	34 818 548 (29 604 427–40 645 134)	20 166.71 (17 146.72–23 541.44)	0.03 (−0.03 to −0.02)	0.01 (0 to 0.03)	−0.06 (−0.07 to −0.06)	−0.02 (−0.03 to −0.01)
Southern Latin America	3 275 921 (2 912 406–3 692 700)	21 947.11 (19 511.73–24 739.33)	3 149 414 (2 796 177–3 586 544)	21 726.78 (19 289.92–24 742.41)	0.03 (−0.04 to −0.02)	−0.11 (−0.12 to −0.1)	−0.02 (−0.04 to 0)	0.03 (0.01 to 0.04)
Southern sub-Saharan Africa	10 235 442 (9 071 879–11 543 032)	49 472.1 (43 848.12–55 792.21)	11 868 728 (10 532 740–13 403 762)	49 317.71 (43 766.33–55 696.19)	0.02 (−0.04 to 0)	0.1 (0.09 to 0.12)	0.04 (−0.01 to 0.08)	−0.19 (−0.22 to −0.15)
Tropical Latin America	11 941 810 (10 175 094–13 969 942)	22 273.69 (18 978.43–26 056.53)	10 890 385 (9 351 025–12 701 998)	21 697 (18 630.12–25 306.29)	0.09 (−0.09 to −0.08)	−0.18 (−0.19 to −0.17)	−0.13 (−0.13 to −0.12)	0.03 (0.01 to 0.04)
Western Europe	15 354 109 (13 522 722–17 593 154)	21 619.94 (19 041.18–24 772.71)	14 519 423 (12 717 589–16 686 813)	21 315 (18 669.85–24 496.8)	0.05 (−0.06 to −0.04)	−0.07 (−0.09 to −0.06)	−0.15 (−0.15 to −0.14)	0.07 (0.05 to 0.09)
Western sub-Saharan Africa	58 094 201 (50 423 025–68 089 113)	66 106.41 (57 377.25–77 479.8)	142 980 118 (124 131 388–166 285 471)	66 575.54 (57 799.05–77 427.16)	0.02 (−0.02 to 0.05)	0.23 (0.18 to 0.29)	−0.14 (−0.19 to −0.09)	−0.06 (−0.13 to 0.01)

AAPC: average annual percentage change; GBD: Global Burden of Disease; SDI: Socio-Demographic Index.

From 1990 to 2021, the overall DALYs rate of ISDs in children exhibited a downward trend. The global trend of DALYs rate in bacterial skin diseases dropped by 51% (AAPC=−2.28). Conversely, the trend of fungal skin diseases increased by 17% (AAPC=0.5); it also increased at a slower rate in viral skin diseases with an AAPC of 0.29 (Table S2).

### Global trends by gender and age groups

In 2021, the new cases of overall ISDs in children were higher in males, as was the number of ISDs-associated DALYs in children. The incidence rates of bacterial skin diseases and viral skin diseases were higher in females, whereas the DALYs rates were higher in males. For bacterial skin diseases, the incidence rate was highest among children aged <5 y in both 1990 and 2021. However, children aged 10–14 y showed the fastest increase. The DALYs rate decreased in children of all age groups, especially in children aged <5 y. As regards fungal skin diseases, both the highest incidence rate (in 1990 and 2021) and the largest increase occurred in children aged 5–9 y. The DALYs rate increased for all age groups, especially in children aged 10–14 y. For viral skin diseases, the highest incidence and DALYs rate were observed in children aged 5–9 y in both 1990 and 2021, and the greatest increase occurred in children aged <5 y (Table 1, Figure 1B–D, Figure S1, Figure S2).

### Global trends by SDI

Across five SDI regions, the overall incident cases of ISDs generally exhibited an upward trend in middle, low-middle and low-SDI regions from 1990 to 2021, whereas it declined in high-middle and high-SDI regions. Specifically, low-SDI regions had the highest incidence rate of bacterial skin diseases in both 1990 and 2021, with the 2021 incidence rate being nearly fourfold that of the high-SDI region with the lowest rate. From 1990 to 2021, the high-middle-SDI region is the only one to show a declining trend in the incidence of bacterial skin diseases. Regarding fungal skin diseases, three out of five SDI regions reported a decrease in incidence (except for middle and low-middle-SDI regions). However, the high-SDI region had the highest incidence of viral skin diseases in both 1990 and 2021; the incidence increased continuously across all five SDI regions. Among them, the high-middle-SDI region had the fastest growth (AAPC=0.18) (Tables S3–S5).

From 1990 to 2021, the DALYs rate decreased in all five SDI regions for bacterial skin diseases. For fungal and viral skin diseases the trends were similar to incidence (Figures S3–S6).

### Regional trend

**Figure 2.**
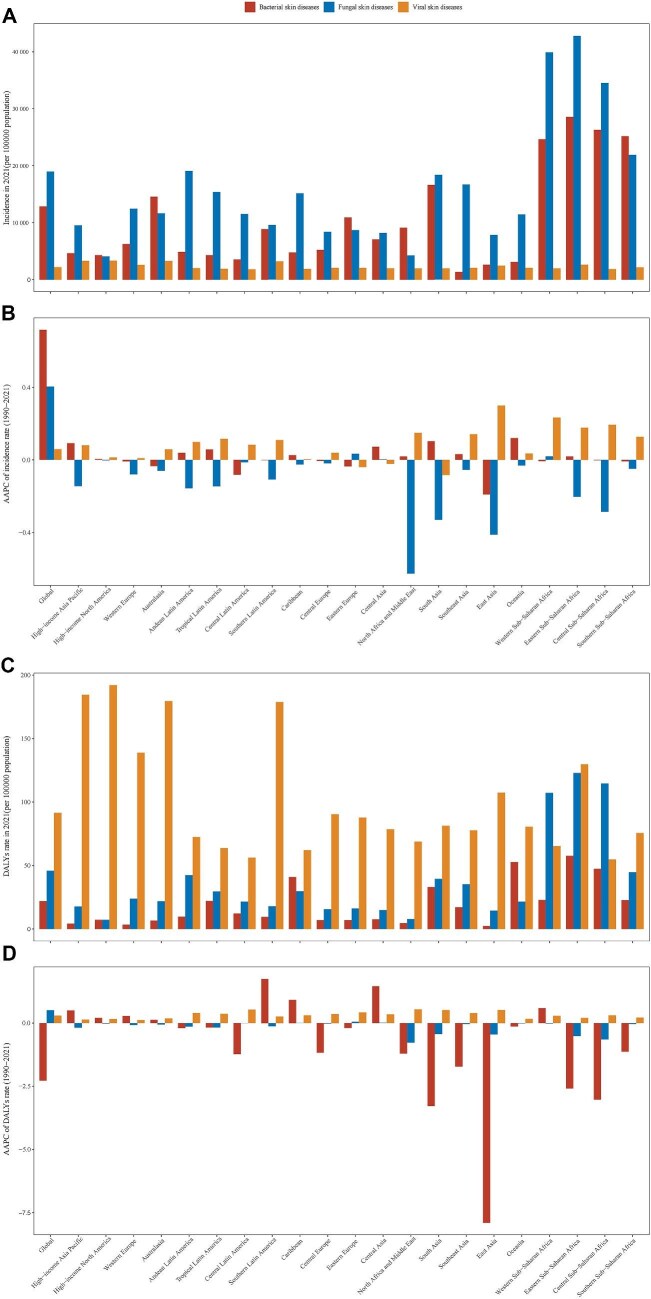
Incidence and DALYs rate in 2021 and their average annual percentage changes from 1990 to 2021 for infectious skin diseases in children, globally and in 21 GBD regions. (A) Incidence rate of bacterial, fungal and viral skin diseases in 2021, (B) average annual percentage changes of incidence rate from 1990 to 2021 for bacterial, fungal and viral skin diseases, (C) DALYs rate of bacterial, fungal and viral skin diseases in 2021 and (D) average annual percentage changes of DALYs rate from 1990 to 2021 for bacterial, fungal and viral skin diseases. DALYs: disability-adjusted life years; GBD: Global Burden of Disease.

**Figure 3.**
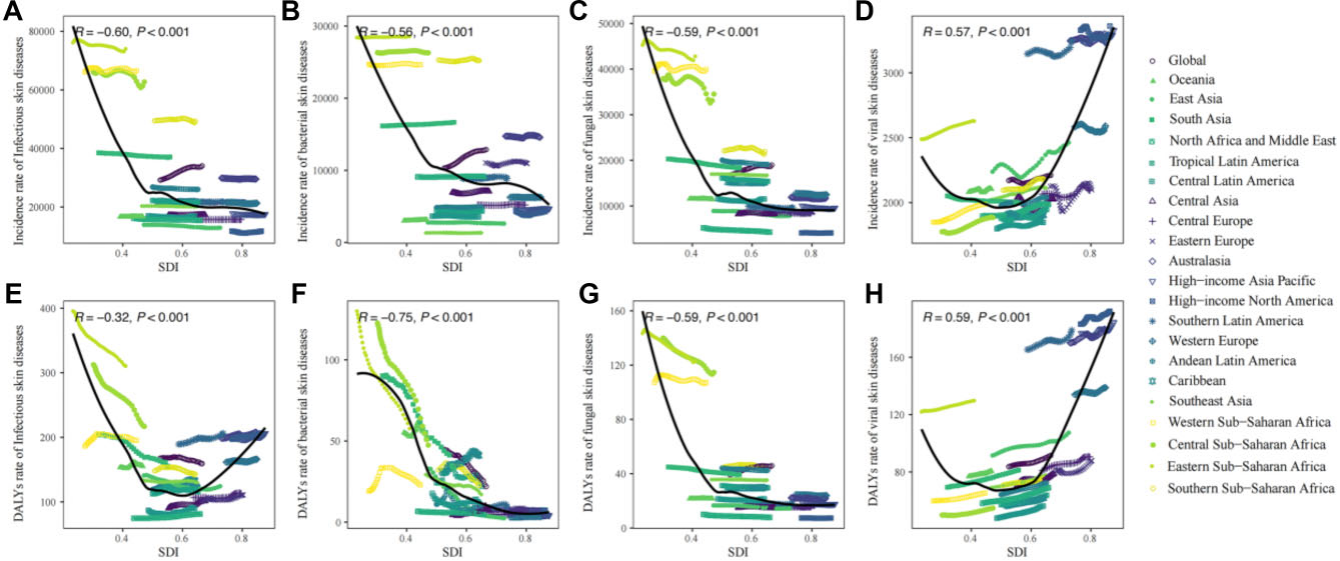
Incidence and DALYs rate of infectious skin diseases in children, globally and for 21 GBD regions, by SDI (2021), from 1990 to 2021; 32 points are plotted for each region and show the observed incidence or DALYs rates for each year from 1990 to 2021. Points above the solid line represent a higher-than-expected burden, and those below the line show a lower-than-expected burden. (A) Incidence rate of overall infectious skin diseases by SDI, (B) incidence rate of bacterial skin diseases by SDI, (C) incidence rate of fungal skin diseases by SDI, (D) incidence rate of viral skin diseases by SDI, (E) DALYs rate of overall infectious skin diseases by SDI, (F) DALYs rate of bacterial skin diseases by SDI, (G) DALYs rate of fungal skin diseases by SDI and (H) DALYs rate of viral skin diseases by SDI. DALYs: disability-adjusted life years; GBD: Global Burden of Disease; SDI: Socio-Demographic Index.

At the regional level in 2021, the highest incidence of overall childhood ISDs was observed in Eastern sub-Saharan Africa; it also had the highest incidence for bacterial and fungal skin diseases. High-income North America reported the highest incidence for viral skin diseases. Similar findings were also observed in DALYs. From 1990 to 2021, the incidence rate increased across 15 regions for viral skin diseases, with the highest increase in East Asia (AAPC=0.3). It decreased across 15 regions for fungal skin diseases, with the highest decrement in North Africa and Middle East (AAPC=−0.63). The incidence of bacterial skin diseases increased across nine regions (the highest in Oceania), decreased in five regions (the highest in East Asia) and remained unchanged in seven regions (Table 1, Figures 2–3, Figure S7).

### National trend

**Figure 4.**
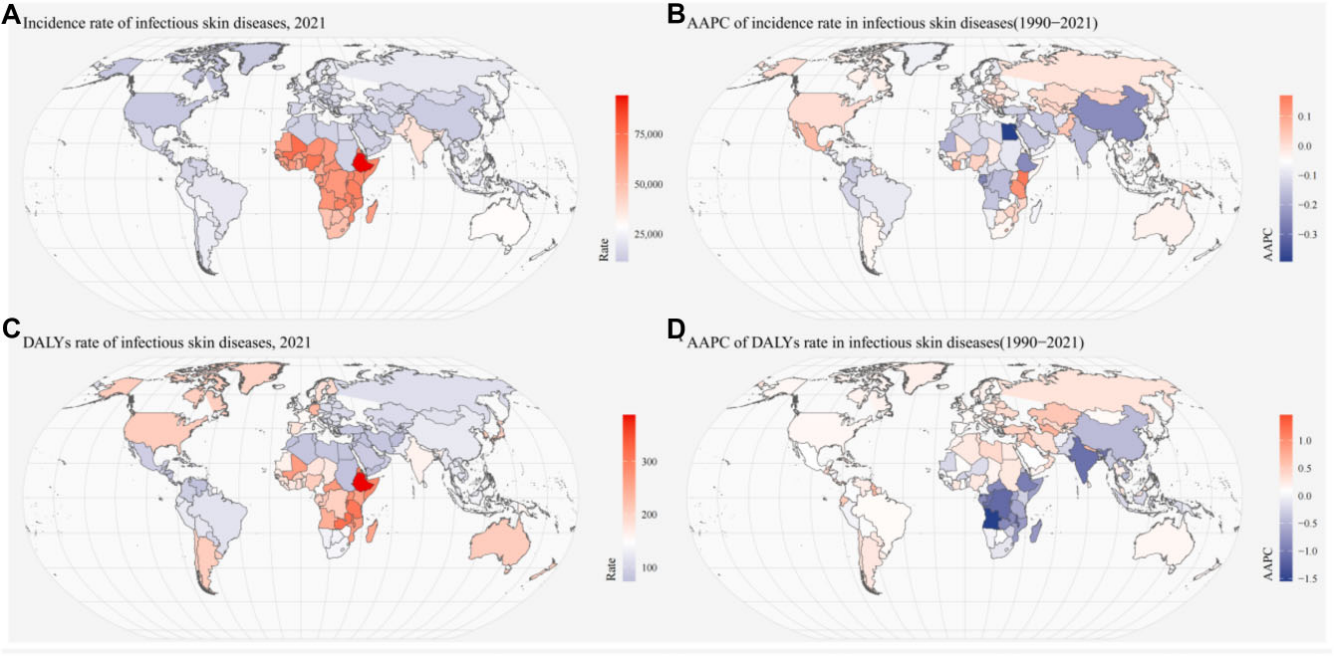
National (204 countries and territories) incidence and DALYs rates of infectious skin diseases in children in 2021, and their AAPCs from 1990 to 2021. (A) Incidence rate of overall infectious skin diseases in 2021, (B) AAPCs of incidence rate from 1990 to 2021 for overall infectious skin diseases, (C) DALYs rate of overall infectious skin diseases in 2021 and (D) AAPCs of DALYs rate from 1990 to 2021 for overall infectious skin diseases. AAPC: average annual percentage change; DALYs: disability-adjusted life years.

In 2021, among 204 countries and territories, the top three countries with the highest incident cases and incidence rate of ISDs in children were India, Nigeria, Ethiopia and Ethiopia, Mali, Rwanda. Meanwhile, the top three countries with the highest number and rate of ISDs-associated DALYs were India, China, Nigeria and Ethiopia, South Sudan, Rwanda. In 2021, Ethiopia had the highest incidence of bacterial and fungal skin diseases, while Germany showed the highest incidence for viral skin diseases (Tables S3–S5, Figure S8, Figure S9). From 1990 to 2021, the fastest increasing trends in the incidence of bacterial, fungal and viral skin diseases were seen in Taiwan (Province of China) (AAPC=0.26), Kenya (AAPC=0.3) and Equatorial Guinea (AAPC=0.58), while Indonesia, Egypt and the Bahamas exhibited the most significant decreases. The trends of DALYs rates are shown in Figure 4 and Tables S6–S8.

### The association between age-specific rate and SDI

From 1990 to 2021, across 21 regions, the overall age-specific incidence rate of ISDs was negatively associated with SDI (R=−0.6). The age-specific incidence rate of bacterial and fungal skin diseases also showed a negative association with SDI (R=−0.56 and −0.59, respectively), whereas the rate of viral skin diseases was positively associated with SDI (R=0.57). Regarding 204 countries and territories in 2021, the association between age-specific rate and SDI was similar in 21 regions (Figure S10). The association between DALYs rate and SDI is shown in Figure 3 and Figure S11.

### Cross-country health inequality of DALYs in children

The inequality slope index of age-specific DALYs rate for ISDs among children in 1990 was −111.81 (95% CI −139.61 to −84), indicating that countries with lower SDI bore disproportionately higher burdens, and this gap narrowed to −68.8 (95% CI −91.18 to −46.41) in 2021. Meanwhile, concentration index remained nearly constant from 1990 (−0.12) to 2021(−0.11), also suggesting that higher DALYs were concentrated in countries with lower SDI. Additionally, the inequality slope index for bacterial, fungal and viral skin diseases changed from −59.91, −82.32 and 30.42 in 1990 to −30.9, −73.73 and 35.84 in 2021, respectively (Figure S12); the concentration index of these diseases burden also remained nearly stable (Table S9, Figure S13).

## Discussion

Over the past three decades, the incidence rate of overall ISDs has increased among children worldwide, similar to the DALYs rates in fungal and viral skin diseases. Specifically, 36 out of 204 countries and territories have experienced increasing DALYs rates for fungal skin diseases, predominantly in low- and middle-income countries (LMICs), with the United Republic of Tanzania, Kenya and Lesotho showing the largest increases. Limited access to broad-spectrum antifungal agents, such as amphotericin B, in LMICs may contribute to higher mortality or persistent infections, potentially driving the observed increase in DALYs rates.^13^ Furthermore, the increasing DALYs rates for viral skin diseases may be attributed to the ability of the MC virus and HPV to evade local immune defenses, leading to chronic or recurrent infections.^6,14^ However, the DALYs rate of bacterial skin diseases has decreased, likely due to improved access to antibiotics, especially in LMICs.^15^

A negative correlation was observed between the SDI and both the incidence and DALYs rate of bacterial skin diseases, suggesting that socioeconomic status plays a critical role in their prevention and control.^12^ Additionally, these disparities were influenced by various factors, including cultural practices, environmental hygiene, public health management and, notably, antimicrobial resistance (AMR).^1,16^ AMR is an escalating concern that contributes to elevated DALYs rates from bacterial infections. From 1990 to 2021, AMR-related mortality declined in children aged <5 y but increased in individuals aged ≥5 y, with MRSA showing the largest global increase in AMR-attributable deaths.^17^ Moreover, the emergence of virulent pathogens—such as novel GAS clones—can enhance treatment resistance and contribute to a higher burden of bacterial skin diseases, particularly among children with immature immune systems.^3^ Fortunately, several novel antibiotics—such as telavancin, tedizolid, dalbavancin and oritavancin—have recently been introduced as effective treatment options for skin infections, including MRSA-related cellulitis.^2^ Several evidence-based interventions have been implemented to reduce the risk of MRSA, including antimicrobial stewardship, contact precautions for individuals colonized or infected with MRSA and routine screening and surveillance.^18^ In parallel, the WHO has developed a research and technology road map for GAS and outlined preferred vaccine characteristics, fostering renewed interest in the development of effective GAS vaccines.^19^

The incidence and DALYs rate of fungal skin diseases are negatively correlated with SDI and disproportionately affect children in LMICs. Multiple factors can contribute to the elevated burden of fungal skin diseases in many LMICs, including low socioeconomic status, under-resourced health systems, high population density, overcrowded living conditions, poor hygiene practices and the overuse of over-the-counter antimicrobials.^20,21^ In 2021, the incidence of fungal skin diseases was highest in Eastern, Western and Central sub-Saharan Africa, while the lowest incidence was recorded in high-income North America. Notably, incidence rates were significantly higher in tropical regions near the Equator compared with other areas. This pattern may be attributed to the optimal growth conditions for dermatophytes, which thrive at surface temperatures of 25–28°C, with warm and humid climates favoring the development of human skin infections.^20^ Climate change is enabling some fungal pathogens to develop thermotolerance. Combined with the extensive use of limited systemic antifungal drugs, this is driving the emergence of antifungal-resistant fungal pathogens such as multidrug-resistant *Candida auris*, fluconazole-resistant *Candida parapsilosis* and *Candida tropicalis*. These pathogens can cause devastating infections, posing significant challenges to clinicians. This has thereby further heightened interest in novel agents for the treatment of priority fungal pathogens.^22^ Exemplary are the newly emerged drugs approved by the US Food and Drug Administration (FDA), such as rezafungin, ibrexafungerp and oteseconazole (novel antifungals for yeast infections) and olorofim and manogepix/fosmanogepix (novel antifungals for mold infections).^23^

Viral skin diseases consist of viral warts and MC. According to the GBD classification framework, these conditions are classified as level 3 non-communicable diseases. By contrast, sexually transmitted infections are categorized under the communicable disease’s module, specifically within the ‘HIV/AIDS and sexually transmitted infections’ group. Therefore, the viral skin diseases analyzed in this study exclude sexually transmitted genital warts, as these are primarily spread through direct contact or, less commonly, via perinatal transmission. MC has the greatest incidence in individuals aged 1–14 y.^24^ Previously, freezing the lesions with liquid nitrogen or laser therapy may have been recommended. Topical cantharidin 0.7% (Ycanth), approved by the FDA in 2023, is the first drug treatment for MC.^25^ However, the incidence and DALYs rate of viral skin diseases among children are positively correlated with SDI, suggesting a heavier disease burden in developed countries. This phenomenon may be attributed to improved healthcare accessibility, greater disease awareness and more advanced diagnostic capabilities in high-SDI regions, all of which contribute to enhanced detection and reporting of skin diseases. By contrast, previous research has documented significant under-reporting and undiagnosed cases in lower-SDI regions.^26^

With ongoing advancements in the global economy and healthcare systems, there is optimism surrounding the declining DALYs rate of ISDs in children. However, the incidence of bacterial, fungal, viral and overall ISDs continues to increase. Contributing to this trend are the increasing global prevalence of childhood obesity and diabetes—both major public health concerns—which are known to elevate the risk of infectious skin diseases.^27,28^ Moreover, the growing number of immunocompromised children, such as those undergoing cancer chemotherapy, organ transplantation or long-term glucocorticoid therapy, further increases susceptibility to these infections.^29,30^ Additionally, global warming is likely to expand the global biological space in which microorganisms, humans and animals interact.^31^ Under the One Health and Global Health concepts, policies need to focus efforts and resources on ISDs among children, especially because a child’s life from the age of 2 to 5 y offers a window of opportunity to promote nurturing and caring environments, establishing healthy behaviors.^11,32^ There are numerous interventions that can be utilized. First, improvement in primary care and public health management of skin infections is needed, such as training of healthcare workers and school-based treatments.^33^ Second, infrastructure and environmental improvements, for instance, high-quality water supply, housing improvement programs and household spraying of insecticides, are essential.^21^ Third, priority should be given to evidence-based antibiotic use, strengthened infection surveillance in immunocompromised populations and the rational implementation of HPV vaccination programs for children.^11,34^ Fourth, promoting a balanced diet and regular physical activity are essential for managing childhood obesity, particularly in high-income settings, while increased attention should also be directed towards screening for childhood diabetes.^35,36^

Our study has several limitations. First, this study relied exclusively on data from the GBD database. The accuracy of this database is limited by the availability and quality of national registry data, with restricted access to original data sources, particularly in LMICs.^37^ Second, this study focuses on three major categories of ISDs—bacterial, fungal and viral—while excluding parasitic ISDs such as scabies. Although these three categories represent the majority of ISDs, scabies also imposes a substantial global burden on children. In 2021, the incidence of scabies among children reached 10 179.23 per 100 000, with the disease disproportionately impacting those living in impoverished and overcrowded conditions.^38^ Third, the lack of subtype-specific data for each ISD precluded further analysis, as this information was not available from the Global Health Data Exchange.

In conclusion, ISDs among children pose a public health challenge worldwide. From 1990 to 2021, the global burden of ISDs incidence in children gradually increased, and this burden is expected to continue rising. Regional disparities and health inequality persist; prevention and control healthcare strategies should be optimized to address the needs of children based on region and disease type, especially in LMICs.

## Supplementary Material

ihaf109_Supplemental_Files

## Data Availability

Datasets that are publicly accessible were analyzed in this study. The data can be found at: https://vizhub.healthdata.org/gbd-results/.
