# Supplementary material for: Global burden and cross-country inequality of infectious skin diseases in children: the Global Burden of Disease Study 2021
Source: Int Health. 2025 Oct 14;18(3):480–9. doi: 10.1093/inthealth/ihaf109 (PMC13154834; doi:10.1093/inthealth/ihaf109)
Supplement: ihaf109_Supplemental_Files [file ihaf109_supplemental_files.zip › Appendix_track_changes.docx]

**Appendix**

**Table of content**

**Supplementary figures**

**sFigure1.** Trend of incidence rate in infectious skin diseases by gender and age groups

**sFigure2.** Trend of DALYs rate in infectious skin diseases by gender and age groups.

**sFigure3.** Trends in incidence and DALYs rate for the burden of infectious skin disease by globally and SDI regions from 1990 to 2021.

**sFigure4.** Trends in incidence and DALYs rate for the burden of bacterial skin diseases by different gender and age groups of globally and SDI regions from 1990 to 2021

**sFigure5.** Trends in incidence and DALYs rate for the burden of fungal skin diseases by different gender and age groups of globally and SDI regions from 1990 to 2021

**sFigure6.** Trends in incidence and DALYs rate for the burden of viral skin diseases by different gender and age groups of globally and SDI regions from 1990 to 2021

**sFigure7.** Number of incident cases and DALYs in infectious skin diseases among children in 1990 and 2021

**sFigure8.** National incident cases and incidence rate of infectious skin diseases among children in 2021

**sFigure9.** National number and rate of DALYAs for infectious skin diseases among children in 2021

**sFigure10.** Age specific rates of incidence in infectious skin diseases among children for 204 countries and territories by SDI, in 2021

**sFigure11.** Age specific rates of DALYs in infectious skin diseases among children for 204 countries and territories by SDI, in 2021

**sFigure12.** Absolute income-related healthy inequality of DALYs in infectious skin diseases for children, presented using regression lines, 1990 vs 2021

**sFigure13.** Relative income-related healthy inequality of DALYs in infectious skin diseases for children, presented using concentration curves, 1990 vs 2021

**Supplementary tables**

**sTable1.** ICD codes assigned for infectious skin diseases, GBD 2021 study

**sTable2.** DALYs of infectious skin diseases and their average annual percentage changes from 1990 to 2021

**sTable3.** The incident cases and incidence rate of bacterial skin diseases and their AAPCs from 1990 to 2021 at the global, regional and national levels

**sTable4.** The incident cases and incidence rate of fungal skin diseases and their AAPCs from 1990 to 2021 at the global, regional and national levels

**sTable5.** The incident cases and incidence rate of fungal skin diseases and their AAPCs from 1990 to 2021 at the global, regional and national levels

**sTable6.** Number of DALYs and DALYs rate of bacterial skin diseases and their AAPCs from 1990 to 2021 at the global, regional and national levels

**sTable7.** Number of DALYs and DALYs rate for fungal skin diseases and their AAPCs from 1990 to 2021 at the global, regional and national levels

**sTable8.** Number of DALYs and DALYs rate for viral skin diseases and their AAPCs from 1990 to 2021 at the global, regional and national levels

**sTable9.** Slope index of inequality and concentration index of infectious skin diseases from 1990 to 2021


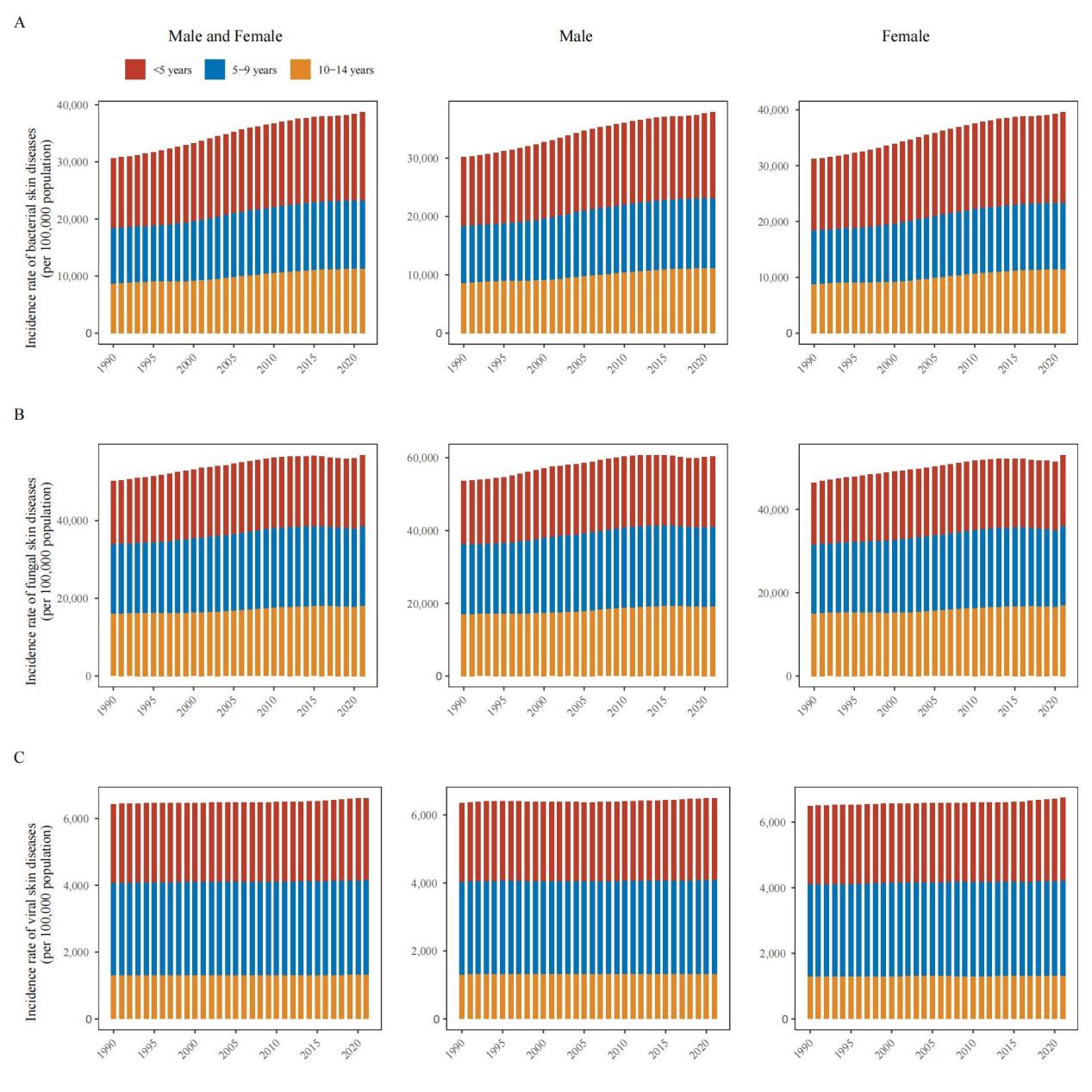


**sFigure1**

Trend of incidence rate in infectious skin diseases by gender and age groups

Incidence rate in bacterial skin diseases from 1990 to 2021 (A),

Incidence rate in fungal skin diseases from 1990 to 2021 (B),

Incidence rate in viral skin diseases from 1990 to 2021 (C).


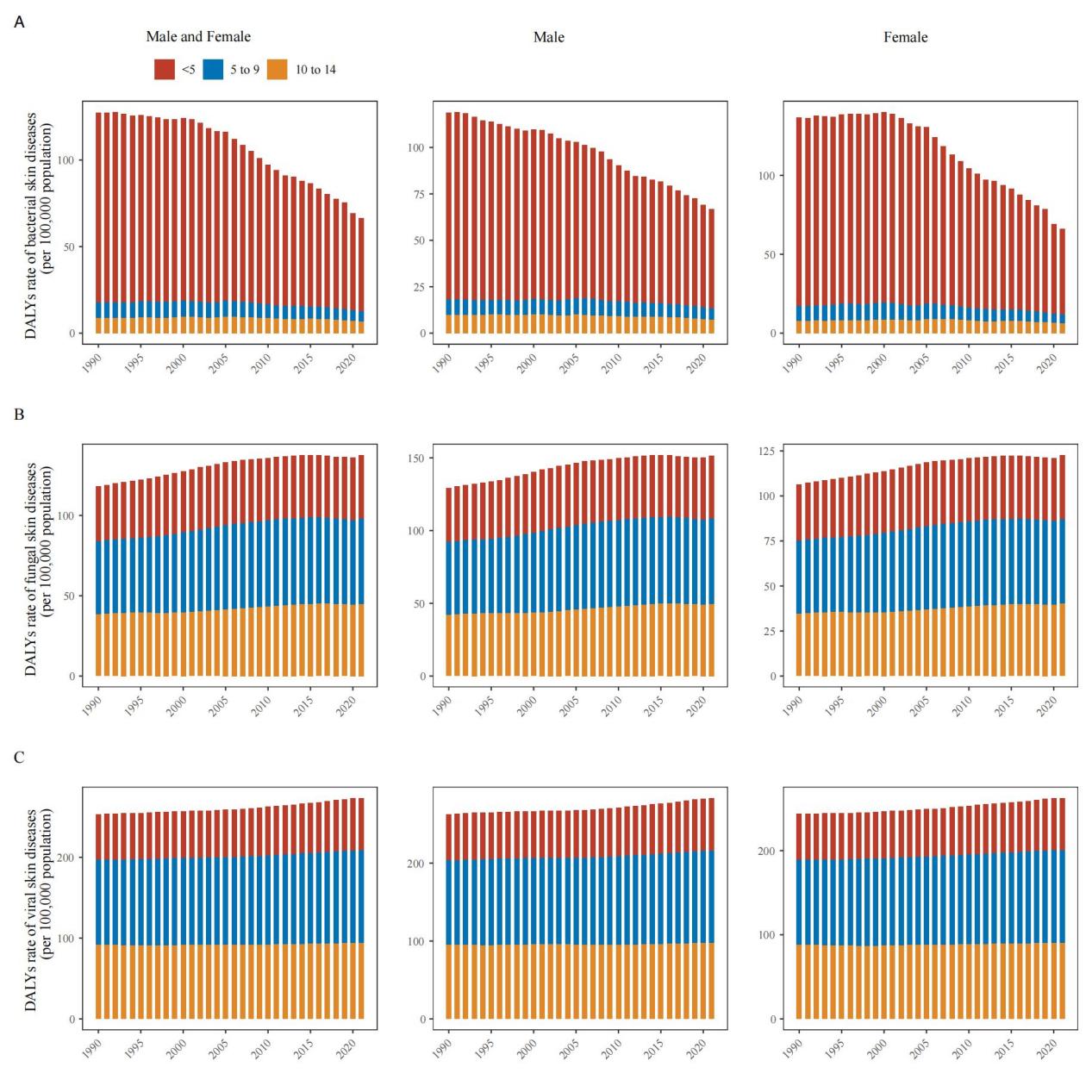


**sFigure2**

Trend of DALYs rate in infectious skin diseases by gender and age groups

DALYs rate in bacterial skin diseases from 1990 to 2021 (A),

DALYs rate in fungal skin diseases from 1990 to 2021 (B),

DALYs rate in viral skin diseases from 1990 to 2021 (C).


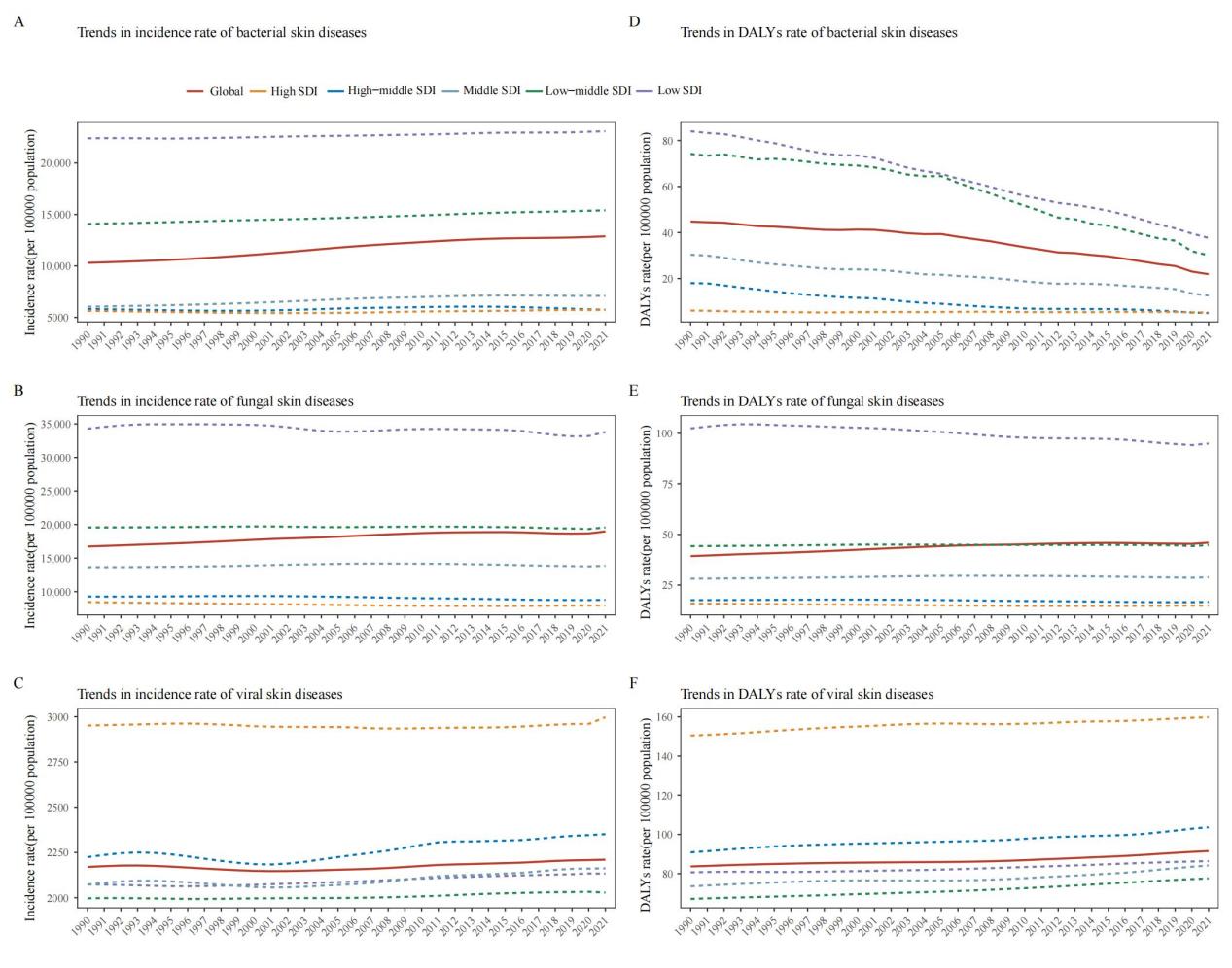


**sFigure3**

Trends in incidence and DALYs rate for the burden of infectious skin diseases by globally and SDI regions from 1990 to 2021

Trends in incidence rate of bacterial skin diseases (A),

Trends in incidence rate of fungal skin diseases (B),

Trends in incidence rate of viral skin diseases (C),

Trends in DALYs rate of bacterial skin diseases (D),

Trends in DALYs rate of fungal skin diseases (E),

Trends in DALYs rate of viral skin diseases (F).


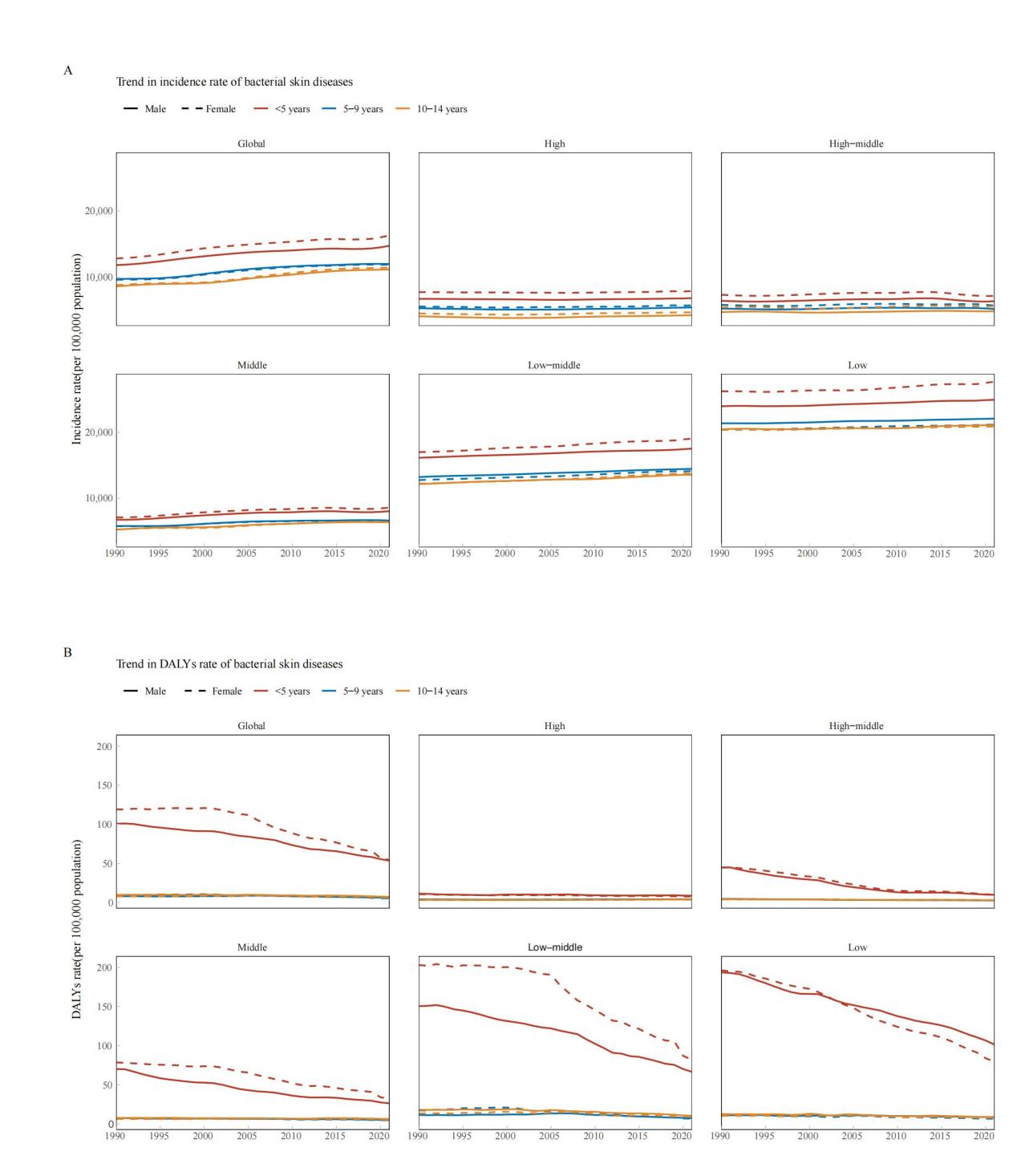


**sFigure4**

Trends in incidence and DALYs rate for the burden of bacterial skin diseases by different gender and age groups of globally and SDI regions from 1990 to 2021

Trend in incidence rate of bacterial skin diseases (A),

Trend in DALYs rate of bacterial skin diseases (B).


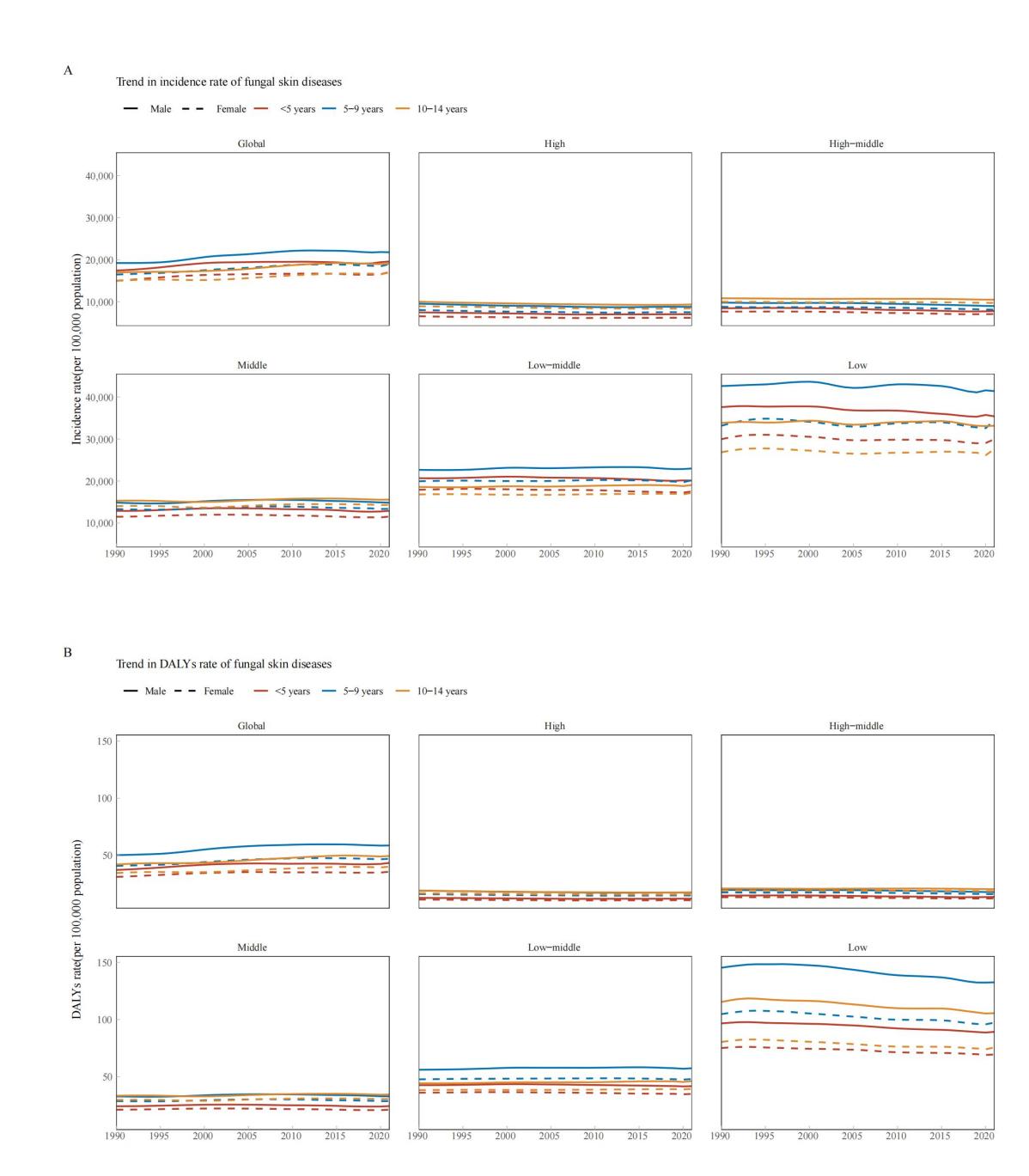


**sFigure5**

Trends in incidence and DALYs rate for the burden of fungal skin diseases by different gender and age groups of globally and SDI regions from 1990 to 2021

Trend in incidence rate of fungal skin diseases (A),

Trend in DALYs rate of fungal skin diseases (B).


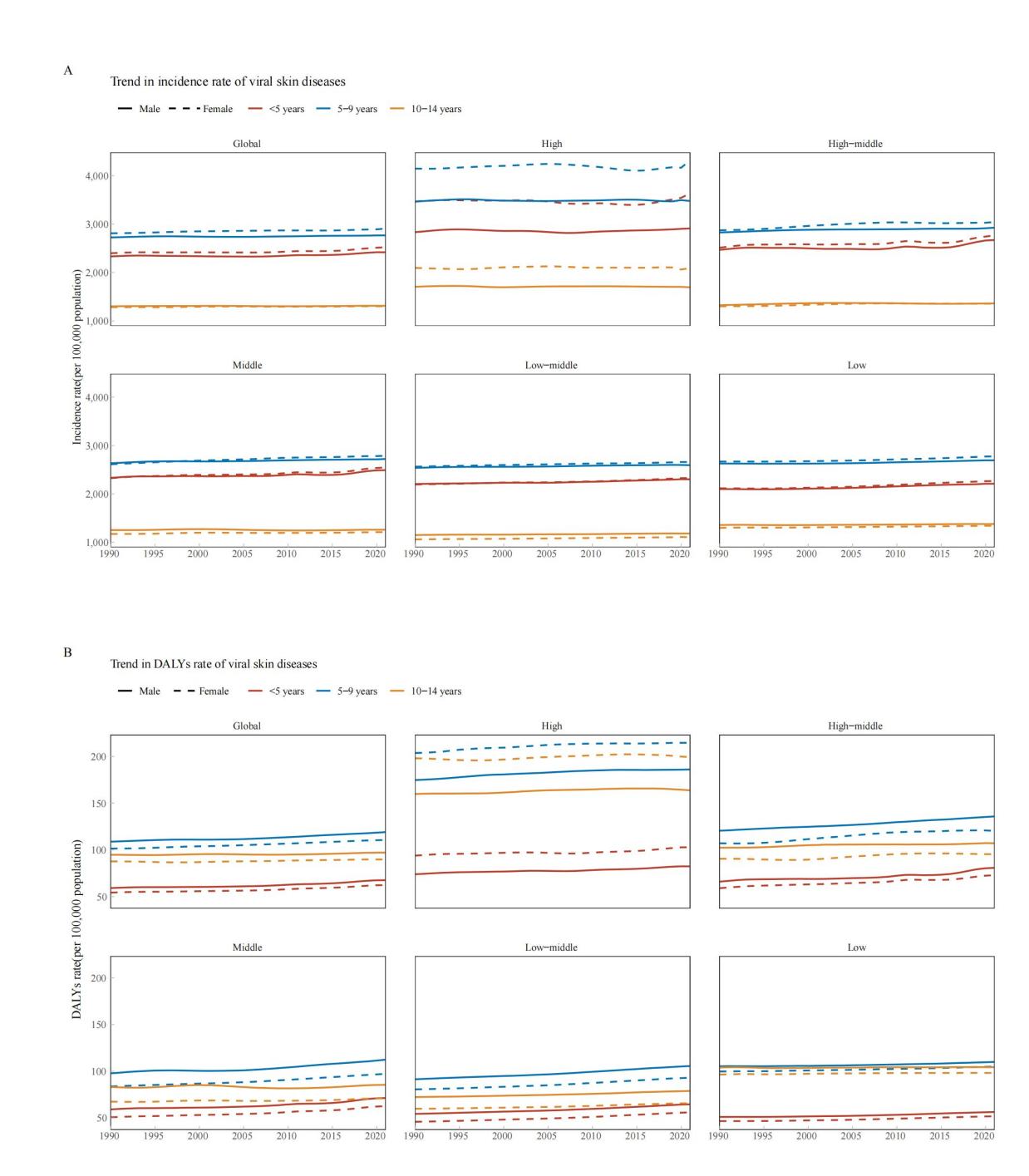


**sFigure6**

Trends in incidence and DALYs rate for the burden of viral skin diseases by different gender and age groups of globally and SDI regions from 1990 to 2021

Trend in incidence rate of viral skin diseases (A),

Trend in DALYs rate of viral skin diseases (B).


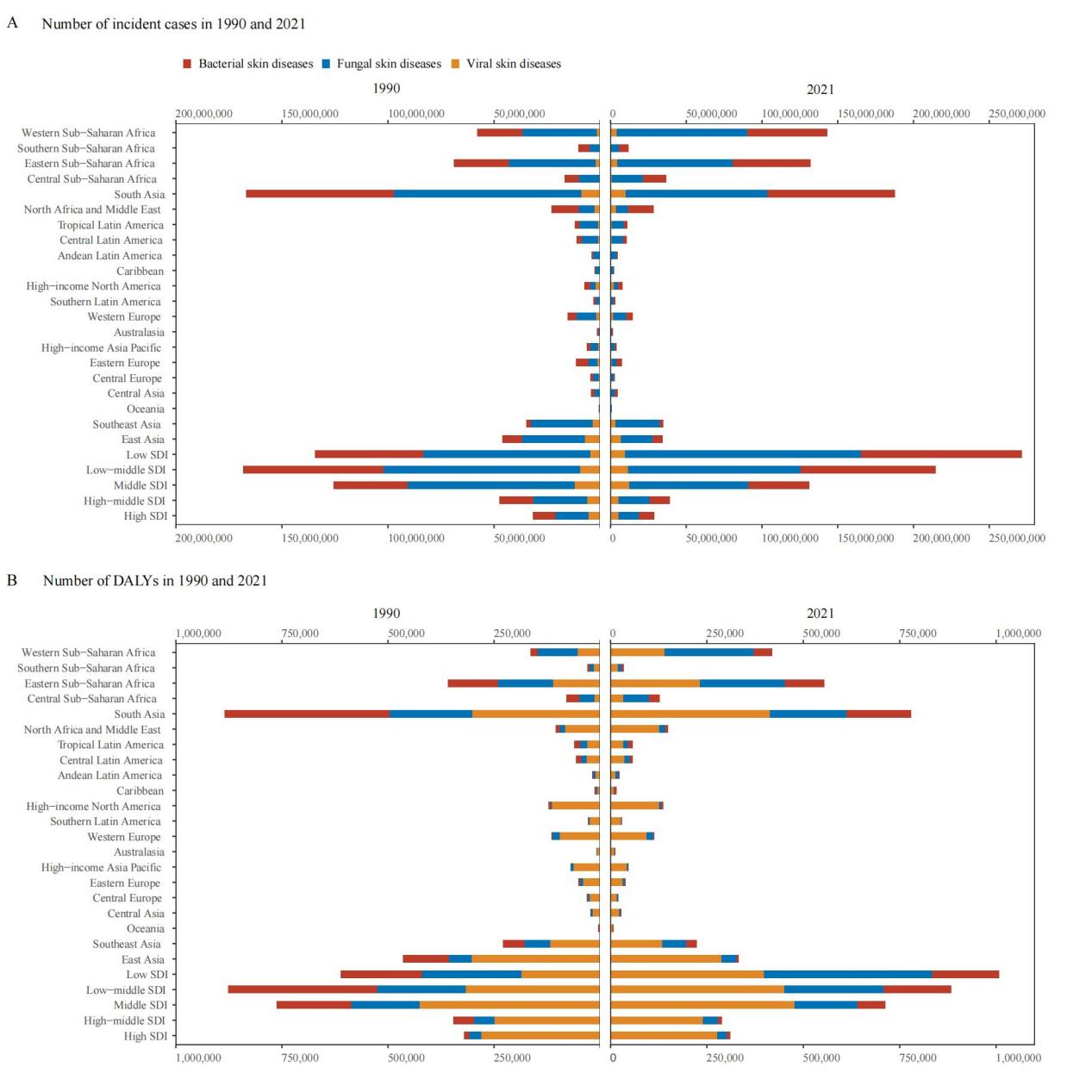


**sFigure7**

Number of incident cases and DALYs in infectious skin diseases among children in 1990 and 2021

Number of incident cases for bacterial, fungal and viral skin diseases in 1990 and 2021 (A),

Number of DALYs for bacterial, fungal and viral skin diseases in 1990 and 2021 (B).


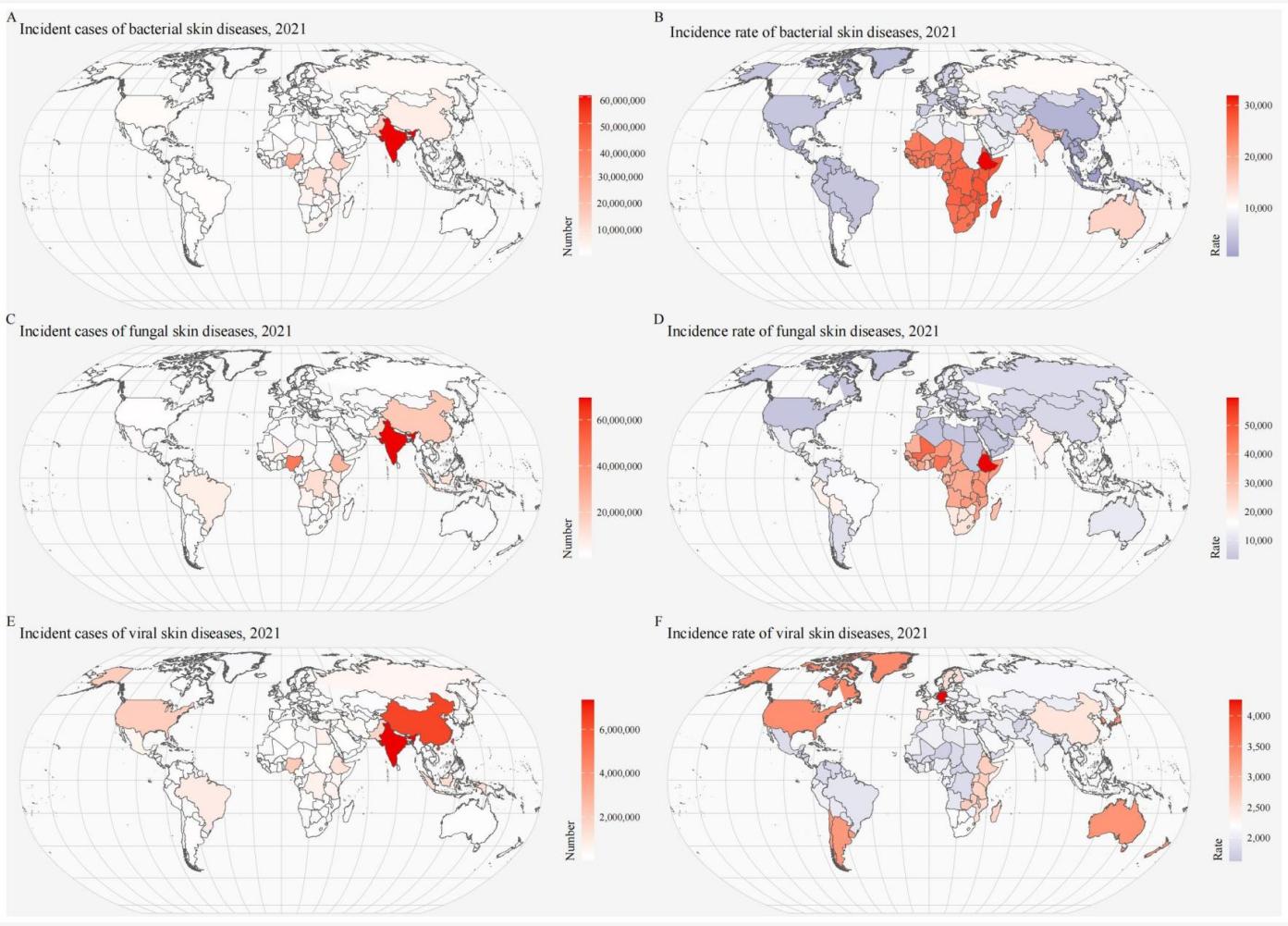


**sFigure8**

National incident cases and incidence rate of infectious skin diseases among children in 2021

Incident cases of bacterial skin diseases (A),

Incidence rate of bacterial skin diseases (B),

Incident cases of fungal skin diseases (C),

Incidence rate of fungal skin diseases (D),

Incident cases of viral skin diseases (E),

Incidence rate of viral skin diseases (F).


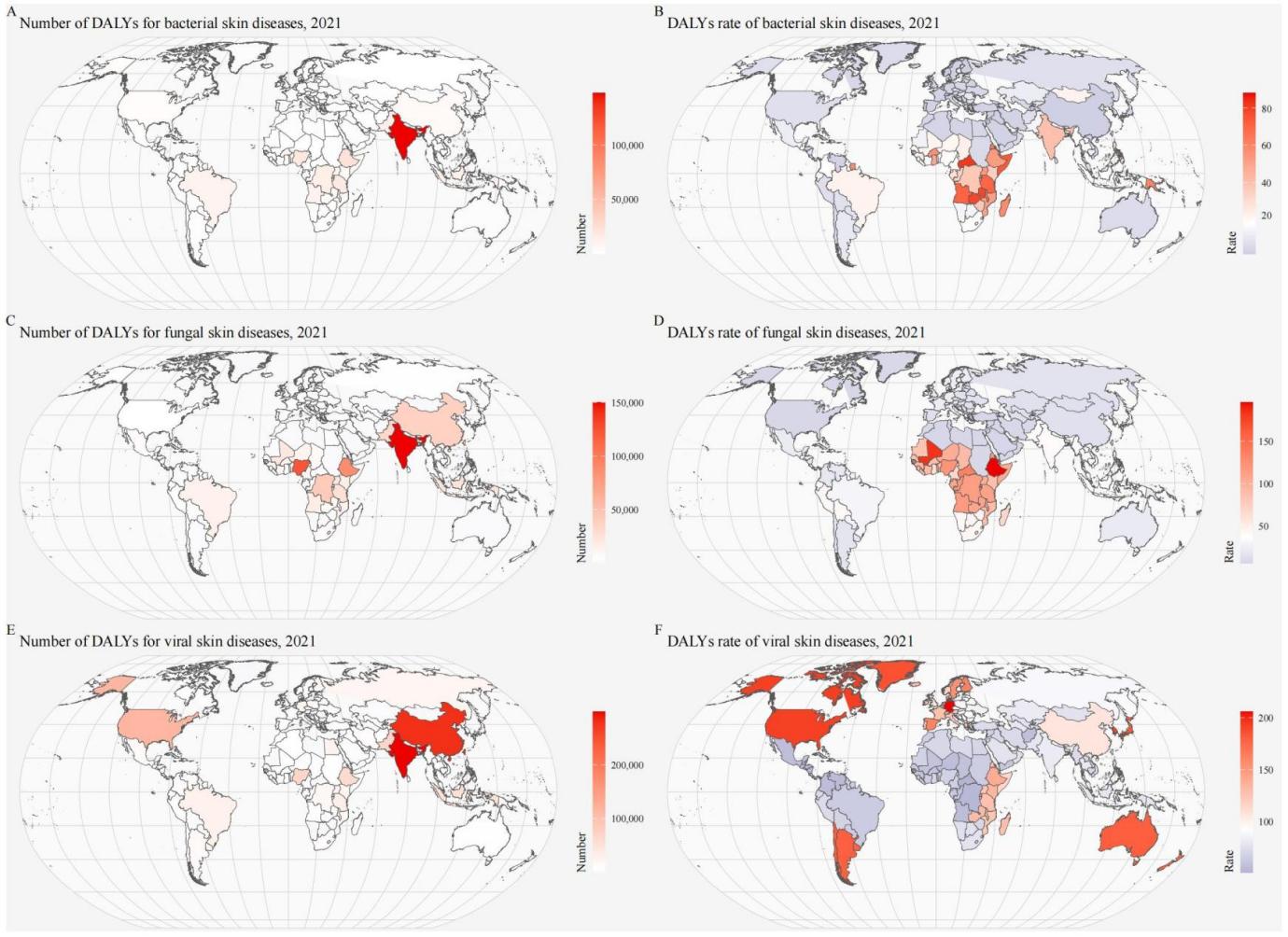


**sFigure9**

National number and rate of DALYAs for infectious skin diseases among children in 2021

Number of DALYs for bacterial skin diseases (A),

DALYs rate of bacterial skin diseases (B),

Number of DALYs for fungal skin diseases (C),

DALYs rate of fungal skin diseases (D),

Number of DALYs for viral skin diseases (E),

DALYs rate of viral skin diseases (F).


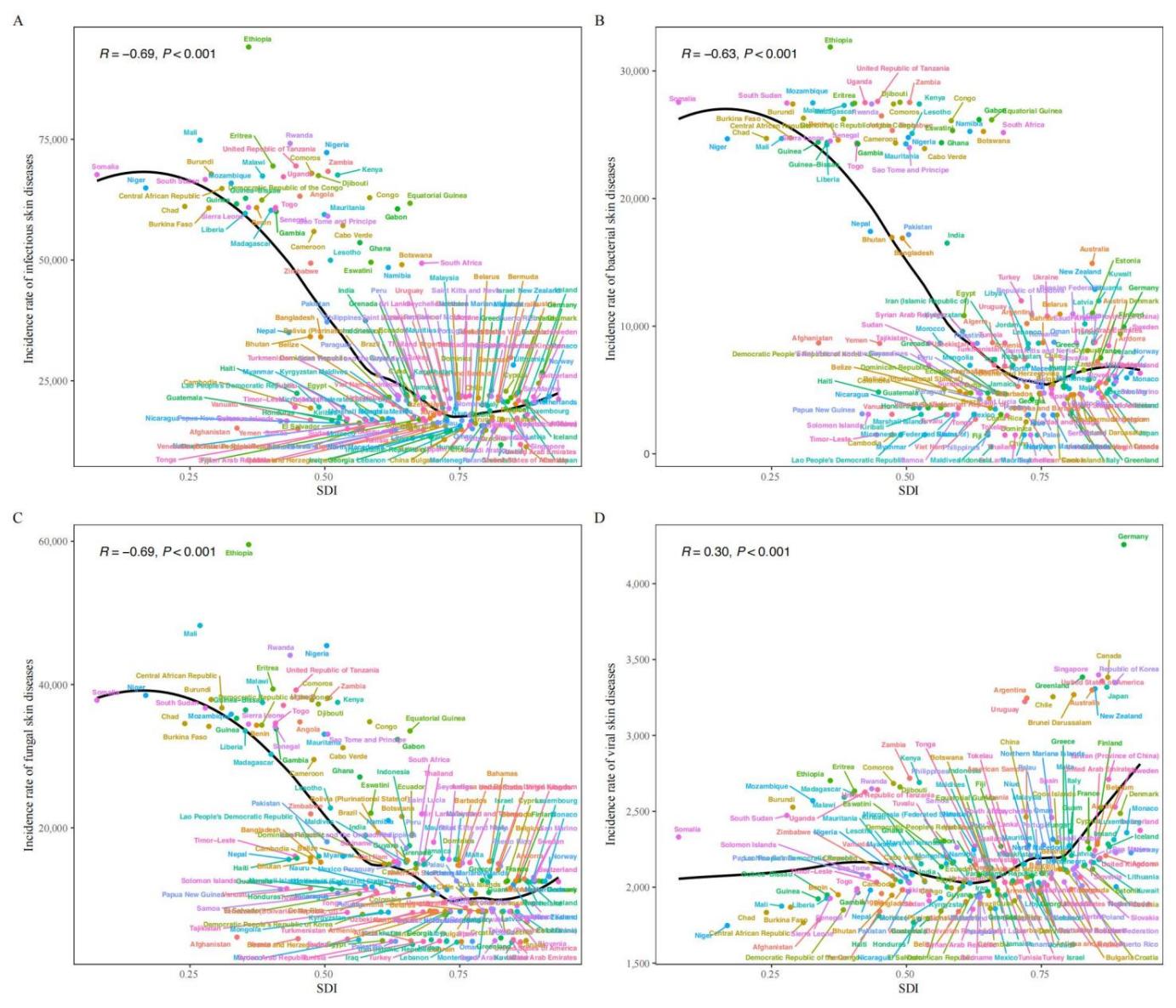


**sFigure10**

Age specific rates of incidence in infectious skin diseases among children for 204 countries and territories by SDI, in 2021

Age specific incidence rate of overall infectious skin diseases (A),

Age specific incidence rate of bacterial skin diseases (B),

Age specific incidence rate of fungal skin diseases (C),

Age specific incidence rate of viral skin diseases (D).


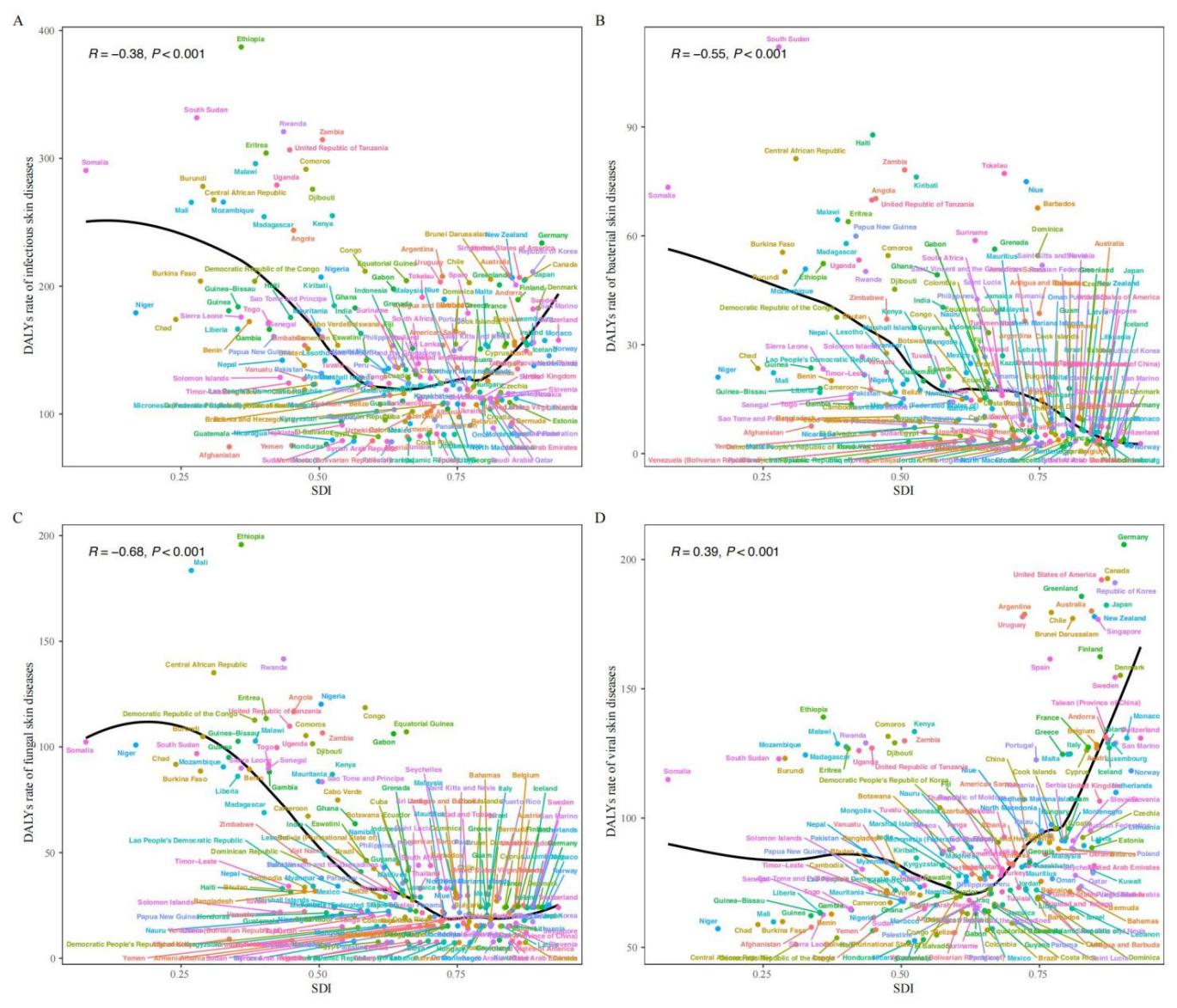


**sFigure11**

Age specific rates of DALYs in infectious skin diseases among children for 204 countries and territories by SDI, in 2021

Age specific DALYs rate of overall infectious skin diseases (A),

Age specific DALYs rate of bacterial skin diseases (B),

Age specific DALYs rate of fungal skin diseases (C),

Age specific DALYs rate of viral skin diseases (D).


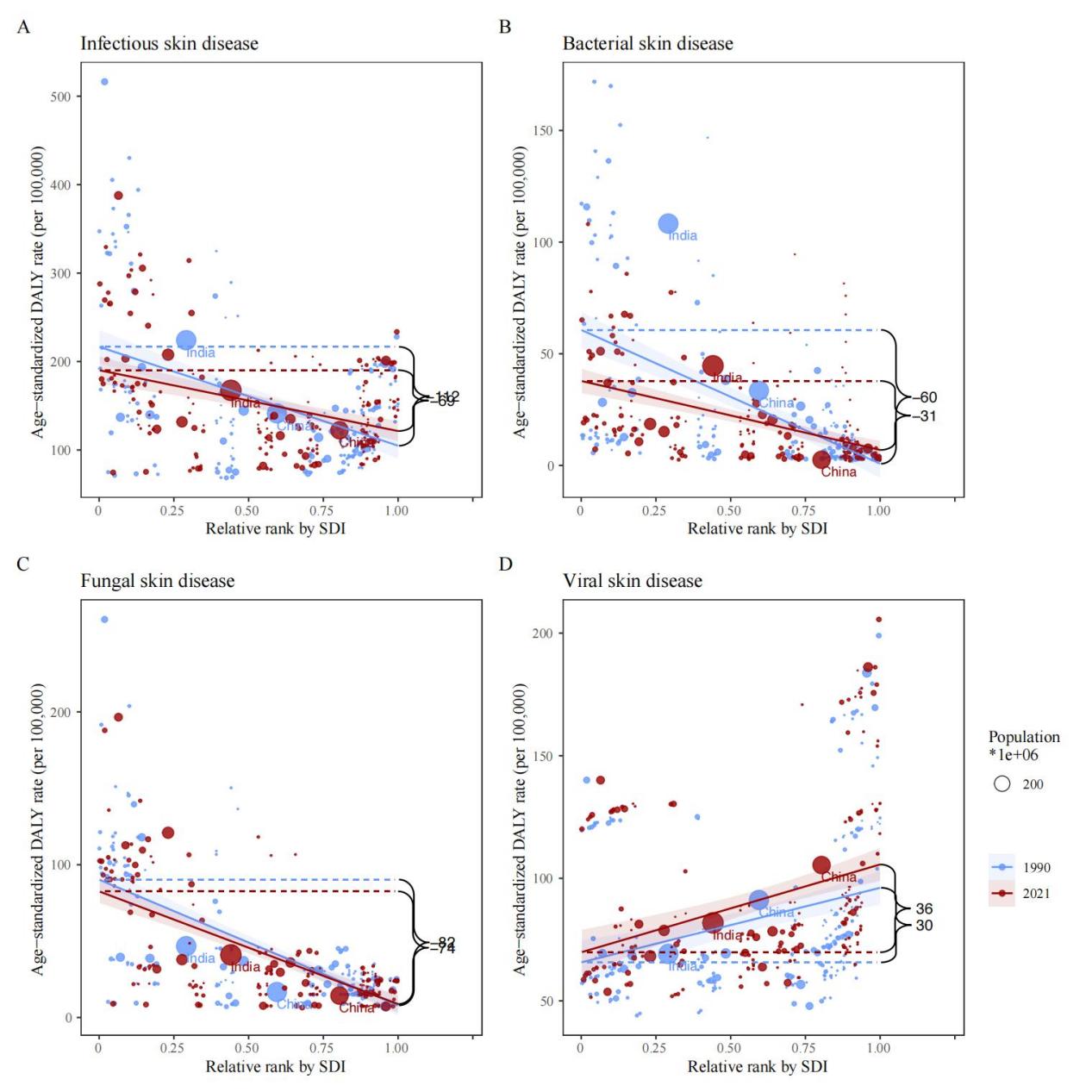


“Viral skin disease” revised to “Viral skin diseases”

“Bacterial skin disease” revised to “Bacterial skin diseases”

“Fungal skin disease” revised to “Fungal skin diseases”

“Infectious skin disease” revised to “Infectious skin diseases”

**sFigure12**

Absolute income-related healthy inequality of DALYs in infectious skin diseases for children, presented using regression lines, 1990 vs 2021. Dots representing countries or territories, with different sizes representing the population sizes.

Slope of the regression line for overall infectious skin diseases (A),

Slope of the regression line for bacterial skin diseases (B),

Slope of the regression line for fungal skin diseases (C),

Slope of the regression line for viral skin diseases (D).


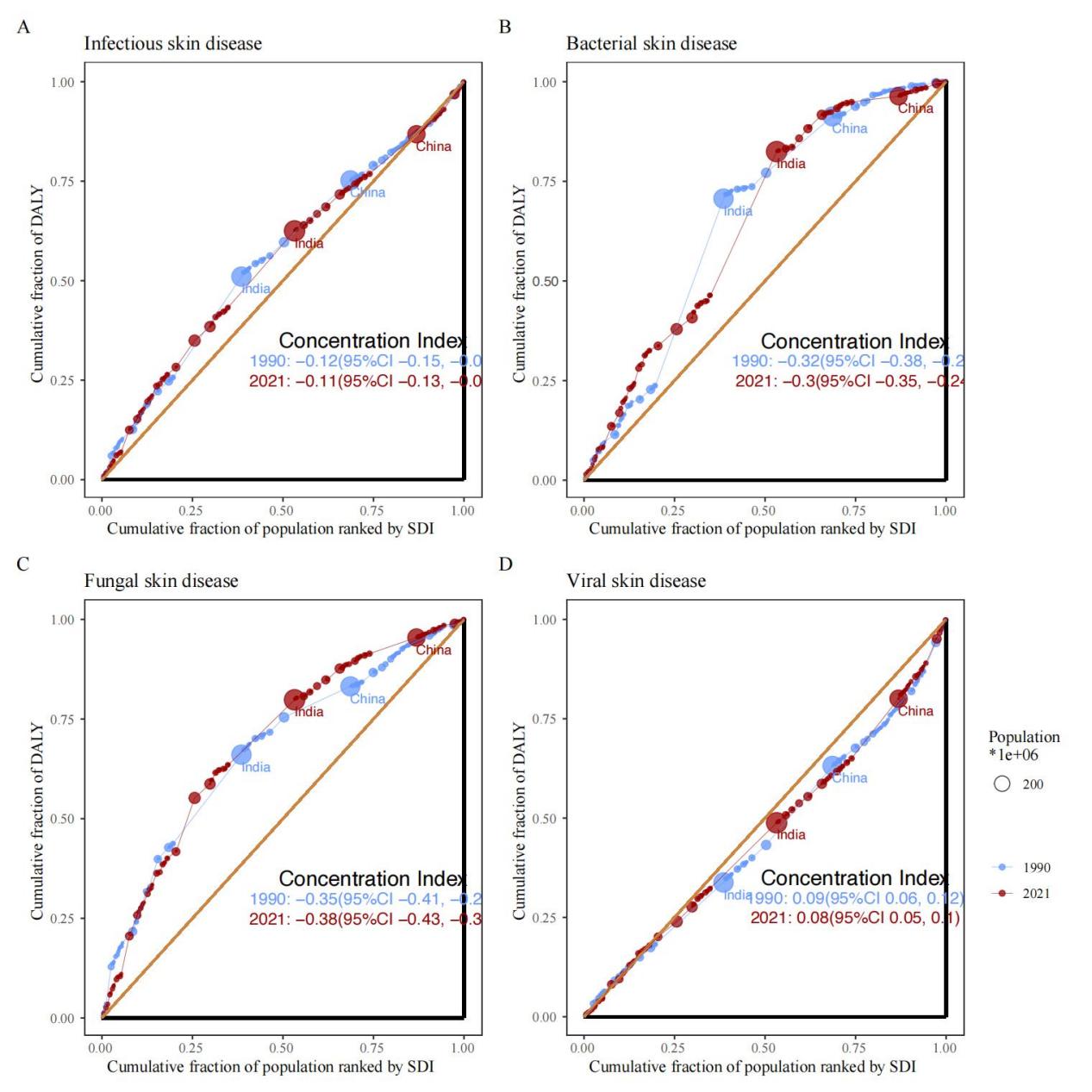


“Bacterial skin disease” revised to “Bacterial skin diseases”

“Infectious skin disease” revised to “Infectious skin diseases”

“Viral skin disease” revised to “Viral skin diseases”

“Fungal skin disease” revised to “Fungal skin diseases”

**sFigure13**

Relative income-related healthy inequality of DALYs in infectious skin diseases for children, presented using concentration curves, 1990 vs 2021. The concentration index representing the relative extent to which the DALYs of infectious skin diseases in children is concentrated among the poor (negative value) or the rich (positive value). Dots representing countries or territories, with different sizes representing the population sizes.

Concentration curve of overall infectious skin diseases (A),

Concentration curve of bacterial skin diseases (B),

Concentration curve of fungal skin diseases (C),

Concentration curve of viral skin diseases (D).

**sTable1 ICD codes assigned for infectious skin diseases, GBD 2021 study**

| **Cause Name** | **ICD-10 code** | **ICD-9 code** |
| --- | --- | --- |
| Bacterial skin diseases | A46-A46.0, A66-A67.3, A67.9, I89.1-I89.8,  L00-L05.92,L08-L08.9,L30.3-L30.4,L88,L97-L98.499,  M72.5-M72.6, N49.2-N49.3 | 035-035.9, 040.0, 102-103.9, 457.2-457.3, 680-689, 728.86, 785.4 |
| Cellulitis | L03-L03.91, M72.5-M72.6 | 681-682.9, 728.86 |
| Pyoderma | A46-A46.0, A66-A67.3, A67.9, I89.1-I89.8,  L00-L02.93, L04-L05.92, L08-L08.9, L30.3-L30.4, L88, L97-L98.499, N49.2-N49.3 | 035-035.9, 040.0, 102-103.9, 457.2-457.3, 680-680.9, 683-689, 785.4 |
| Fungal skin diseases | B35-B36.9 | 110-111.9 |
| Tinea manuum | B35.2 |  |
| Tinea pedis | B35.3 |  |
| Tinea corporis | B35.4 |  |
| Tinea imbricata | B35.5 |  |
| Tinea cruris | B35.6 |  |
| Dermatophytosis | B35.9 |  |
| Viral skin diseases | B07-B09 | 078.0-078.19 |
| Viral warts | B07 |  |
| Molluscum contagiosum | B08.1 |  |

GBD, Global Burden of Disease; ICD, International Classification of Diseases.

**sTable2 DALYs of infectious skin diseases and their average annual percentage changes from 1990 to 2021**

| *Rate per 100,000* | 1990 | | 2021 | | 1990-2021 | 1990-1999 | 2000-2009 | 2010-2021 |
| --- | --- | --- | --- | --- | --- | --- | --- | --- |
| *AAPC(95% CI)* | Number of DALYs | DALYs rate | Number of DALYs | DALYs rate | AAPC | AAPC | AAPC | AAPC |
| Overall | 2,917,657(1,811,153-4,685,706) | 167.76(104.14-269.42) | 3,206,058(1,812,470-5,402,392) | 159.36(90.09-268.53) | -0.17 (-0.22 to -0.12) | 0.07(0.04 to 0.09) | -0.17(-0.22 to -0.11) | -0.36(-0.47 to -0.24) |
| Male | 1,532,492(940,552-2,484,820) | 171.52(105.27-278.1) | 1,739,236(968,338-2,953,942) | 167.53(93.27-284.53) | -0.09(-0.15 to -0.03) | -0.03(-0.09 to 0.03) | -0.03(-0.11 to 0.04) | -0.19(-0.31 to -0.07) |
| Female | 1,385,165(841,034-2,210,046) | 163.8(99.45-261.34) | 1,466,821(830,798-2,470,365) | 150.64(85.32-253.71) | -0.27(-0.34 to -0.21) | 0.15(0.11 to 0.2) | -0.35(-0.51 to -0.18) | -0.55(-0.65 to -0.44) |
| **Cause** | | | | | | | | |
| Bacterial skin diseases | 778,691(618,954-977,771) | 44.77(35.59-56.22) | 440,459(280,002-600,523) | 21.89(13.92-29.85) | -2.28 (-2.5 to -2.05) | -0.85(-0.96 to -0.75) | -1.77(-1.86 to -1.69) | -3.9(-4.5 to -3.31) |
| Fungal skin diseases | 683,592(269,007-1,499,209) | 39.31(15.47-86.2) | 924,335(365,560-2,019,319) | 45.94(18.17-100.37) | 0.5 (0.46 to 0.54) | 0.79(0.78 to 0.81) | 0.64(0.62 to 0.66) | 0.13(0.04 to 0.23) |
| Viral skin diseases | 1,455,375(923,193-2,208,726) | 83.68(53.08-127) | 1,841,264(1,166,908-2,782,549) | 91.52(58-138.31) | 0.29 (0.28 to 0.3) | 0.25(0.23 to 0.27) | 0.11(0.09 to 0.14) | 0.48(0.47 to 0.49) |
| **SDI region** | | | | | | | | |
| High SDI | 320,454(197,160-510,663) | 172.47(106.11-274.83) | 310,505(191,778-491,140) | 179.96(111.15-284.66) | 0.14(0.12 to 0.15) | 0.19(0.17 to 0.21) | 0.04(0 to 0.08) | 0.17(0.16 to 0.18) |
| High-middle SDI | 345,905(218,211-541,842) | 126.42(79.75-198.02) | 289,405(174,122-465,179) | 125.34(75.41-201.47) | -0.03(-0.05 to -0.01) | -0.14(-0.2 to -0.09) | -0.25(-0.28 to -0.22) | 0.24(0.21 to 0.27) |
| Middle SDI | 762,230(484,466-1,202,666) | 132.05(83.93-208.36) | 712,310(419,132-1,179,769) | 125.66(73.94-208.12) | -0.17(-0.22 to -0.13) | -0.24(-0.3 to -0.18) | -0.27(-0.32 to -0.23) | -0.03(-0.12 to 0.06) |
| Low-middle SDI | 875,659(551,658-1,386,827) | 185.48(116.85-293.75) | 883,740(493,265-1,505,526) | 152.41(85.07-259.64) | -0.64(-0.71 to -0.57) | -0.12(-0.18 to -0.06) | -0.77(-0.9 to -0.64) | -0.96(-1.11 to -0.82) |
| Low SDI | 611,442(344,840-1,056,643) | 267.11(150.64-461.59) | 1,007,893(529,390-1,791,587) | 219(115.03-389.28) | -0.65(-0.71 to -0.6) | -0.4(-0.51 to -0.3) | -0.79(-0.84 to -0.74) | -0.76(-0.84 to -0.67) |
| **GBD region** | | | | | | | | |
| Andean Latin America | 17,744(9,628-31,242) | 119.47(64.83-210.36) | 22,548(12,741-38,824) | 124.61(70.41-214.56) | 0.15(0.07 to 0.22) | 0.82(0.7 to 0.95) | -0.96(-1.05 to -0.88) | 0.38(0.24 to 0.53) |
| Australasia | 9,110(5,551-14,694) | 198.65(121.05-320.41) | 11,926(7,210-19,115) | 208.09(125.80-333.52) | 0.15(0.12 to 0.18) | 0.24(0.2 to 0.27) | -0.06(-0.14 to 0.02) | 0.25(0.21 to 0.29) |
| Caribbean | 13,393(6,943-23,448) | 117.36(60.84-205.46) | 15,282(8,174-26,537) | 132.83(71.05-230.65) | 0.41(0.34 to 0.47) | 0.54(0.38 to 0.71) | 0.51(0.45 to 0.57) | 0.23(0.14 to 0.32) |
| Central Asia | 22,565(13,232-36,752) | 90.29(52.95-147.06) | 28,042(16,837-45,087) | 101.32(60.84-162.91) | 0.37(0.31 to 0.44) | 0.48(0.41 to 0.55) | 0.04(0 to 0.08) | 0.61(0.46 to 0.77) |
| Central Europe | 31,466(19,480-49,648) | 106.72(66.07-168.39) | 20,009(12,273-31,669) | 113.04(69.33-178.91) | 0.19(0.14 to 0.24) | -0.11(-0.24 to 0.01) | 0.12(0.09 to 0.16) | 0.43(0.34 to 0.53) |
| Central Latin America | 56,000(35,057-89,241) | 86.98(54.45-138.61) | 57,081(33,985-94,409) | 89.91(53.53-148.71) | 0.11(-0.04 to 0.27) | -0.65(-0.82 to -0.48) | 0.34(0.07 to 0.62) | 0.57(0.26 to 0.88) |
| Central Sub-Saharan Africa | 79,054(41,700-140,747) | 312.48(164.83-556.34) | 127,289(57,611-253,205) | 216.92(98.18-431.49) | -1.17(-1.22 to -1.12) | -0.61(-0.64 to -0.59) | -0.98(-1.09 to -0.88) | -1.85(-1.97 to -1.74) |
| East Asia | 465,139(302,334-705,047) | 141.02(91.66-213.76) | 332,366(199,609-532,617) | 124.32(74.66-199.22) | -0.43(-0.49 to -0.38) | -0.85(-0.9 to -0.8) | -0.89(-0.99 to -0.79) | 0.3(0.19 to 0.41) |
| Eastern Europe | 51,705(31,079-83,796) | 100.47(60.39-162.83) | 39,300(23,797-62,972) | 110.88(67.14-177.67) | 0.32(0.29 to 0.35) | 0.4(0.34 to 0.46) | -0.06(-0.1 to -0.02) | 0.65(0.59 to 0.71) |
| Eastern Sub-Saharan Africa | 358,143(191,843-617,715) | 395.43(211.82-682.02) | 553,859(293,272-975,870) | 310.4(164.36-546.92) | -0.78(-0.81 to -0.76) | -0.75(-0.83 to -0.67) | -0.71(-0.73 to -0.7) | -0.88(-0.92 to -0.85) |
| High-income Asia Pacific | 70,077(42,591-112,257) | 199.09(121-318.92) | 46,257(28,181-73,739) | 206.27(125.67-328.82) | 0.11(0.09 to 0.13) | -0.14(-0.19 to -0.09) | 0.24(0.21 to 0.27) | 0.24(0.22 to 0.26) |
| High-income North America | 121,823(77,037-185,413) | 197.52(124.9-300.62) | 135,657(86,153-205,909) | 206.73(131.29-313.8) | 0.15(0.14 to 0.16) | 0.23(0.2 to 0.26) | -0.02(-0.05 to 0) | 0.22(0.21 to 0.23) |
| North Africa and Middle East | 105,218(64,114-167,667) | 74.9(45.64-119.35) | 149,246(91,404-237,839) | 81.41(49.86-129.74) | 0.27(0.26 to 0.28) | 0.05(0.04 to 0.07) | 0.31(0.28 to 0.33) | 0.42(0.4 to 0.44) |
| Oceania | 4,117(2,394-6,679) | 153.62(89.34-249.25) | 7,877(4,555-12,794) | 155.02(89.65-251.81) | 0.03(-0.01 to 0.07) | 0.05(-0.05 to 0.15) | 0.37(0.31 to 0.42) | -0.28(-0.32 to -0.23) |
| South Asia | 885,821(558,076-1,381,544) | 204.41(128.78-318.8) | 779,886(439,737-1,314,695) | 153.82(86.73-259.3) | -0.94(-1.04 to -0.84) | -0.27(-0.35 to -0.2) | -1.24(-1.41 to -1.07) | -1.22(-1.43 to -1.01) |
| Southeast Asia | 228,719(133,904-379,372) | 133.95(78.42-222.18) | 224,386(130,387-373,624) | 129.96(75.52-216.4) | -0.1(-0.14 to -0.06) | -0.27(-0.3 to -0.24) | 0.06(0.02 to 0.1) | -0.11(-0.21 to -0.01) |
| Southern Latin America | 28,254(17,122-44,976) | 189.29(114.71-301.32) | 29,899(18,487-47,267) | 206.27(127.53-326.08) | 0.28(0.26 to 0.3) | 0.35(0.31 to 0.39) | 0.11(0.1 to 0.13) | 0.38(0.35 to 0.4) |
| Southern Sub-Saharan Africa | 30,625(17,007-51,507) | 148.02(82.2-248.96) | 34,418(18,975-58,985) | 143.01(78.85-245.1) | -0.13(-0.17 to -0.08) | 0.41(0.31 to 0.5) | -0.23(-0.28 to -0.18) | -0.44(-0.51 to -0.36) |
| Tropical Latin America | 60,361(37,016-98,859) | 112.58(69.04-184.39) | 57,961(35,214-94,727) | 115.48(70.16-188.72) | 0.12(-0.15 to 0.39) | 0.9(0.76 to 1.05) | -0.3(-1.02 to 0.42) | -0.25(-0.68 to 0.18) |
| Western Europe | 114,743(68,071-188,438) | 161.57(95.85-265.34) | 113,163(68,047-183,449) | 166.13(99.90-269.31) | 0.09(0.07 to 0.11) | 0.13(0.09 to 0.17) | -0.09(-0.1 to -0.08) | 0.22(0.19 to 0.25) |
| Western Sub-Saharan Africa | 163,580(83,481-307,327) | 186.14(94.99-349.71) | 419,605(213,132-779,057) | 195.38(99.24-362.75) | 0.13(0.06 to 0.21) | 0.84(0.76 to 0.92) | -0.09(-0.23 to 0.05) | -0.28(-0.37 to -0.19) |

**sTable3 The incident cases and incidence rate of bacterial skin diseases and their AAPCs from 1990 to 2021 at the global, regional and national levels**

| *Rate per 100,000* | 1990 | | 2021 | | 1990-2021 | 1990-1999 | 2000-2009 | 2010-2021 |
| --- | --- | --- | --- | --- | --- | --- | --- | --- |
| *AAPC(95% CI)* | Incident cases | Incidence rate | Incident cases | Incidence rate | AAPC | AAPC | AAPC | AAPC |
| Bacterial skin diseases | 179,189,301(170,362,311-188,569,867) | 10,303.26(9,795.72-10,842.64) | 259,111,300(246,067,503-273,425,523) | 12,879.18(12,230.84-13,590.68) | 0.72(0.69 to 0.74) | 0.7(0.64 to 0.77) | 1.08(1.05 to 1.11) | 0.39(0.36 to 0.42) |
| Male | 90,467,887(85,859,968-95,439,828) | 10,125.21(9,609.49-10,681.67) | 130,925,839(124,100,869-138,375,389) | 12,611.27(11,953.86-13,328.84) | 0.71(0.7 to 0.73) | 0.7(0.67 to 0.73) | 1.05(1.03 to 1.06) | 0.4(0.37 to 0.43) |
| Female | 88,721,414(844,330,301-93,316,864) | 10,491.39(9,984.28-11,034.8) | 128,185,461(121,766,196-135,321,641) | 13,164.84(12,505.57-13,897.74) | 0.74(0.72 to 0.76) | 0.71(0.67 to 0.75) | 1.11(1.1 to 1.13) | 0.41(0.37 to 0.46) |
| **Age groups** |  |  |  |  |  |  |  |  |
| 0-4 years | 101,867,150(96,409,568-107,829,328) | 12301.35(11612.94-13006.07) | 101,867,150(96,409,568-107,829,328) | 15,477.29(14,648.09-16,383.16) | 0.75 (0.72 to 0.78) | 1.12(1.07 to 1.16) | 0.67(0.63 to 0.72) | 0.48(0.42 to 0.53) |
| 5-9 years | 81,960,474(74,932,069-89,504,617) | 9,659.67(8,823.96-10,529.82) | 819,604,74(74,320,069-895,461,17) | 11,929.27(10,906.29-13,027.31) | 0.68 (0.65-0.71) | 0.69(0.61 to 0.77) | 1.1(1.07 to 1.13) | 0.25(0.21 to 0.29) |
| 10-14 years | 75,283,675(83,479,946-68,490,117) | 8,691.98(7,887.09-9,607.83) | 752,836,75(83,799,946-684,011,17) | 11293.07(10273.99-12522.57) | 0.84 (0.82-0.87) | 0.49(0.44 to 0.54) | 1.47(1.44 to 1.5) | 0.66(0.63 to 0.69) |
| **SDI region** |  |  |  |  |  |  |  |  |
| High SDI | 10,491,018(10,030,424-10,968,538) | 5,646.17(5,398.28-5,903.16) | 9,909,575(9,470,889-10,334,298) | 5,743.47(5,489.21-5,989.63) | 0.05 (0.02 to 0.08) | -0.39(-0.41 to -0.37) | 0.25(0.21 to 0.3) | 0.25(0.18 to 0.32) |
| High-middle SDI | 15,950,916(15,127,184-16,837,036) | 5,829.52(5,528.47-6,153.37) | 13,283,351(12,548,025-14,037,730) | 5,753.1(5,434.62-6,079.82) | -0.04 (-0.07 to -0.01) | -0.34(-0.37 to -0.31) | 0.6(0.56 to 0.65) | -0.39(-0.45 to -0.33) |
| Middle SDI | 34,914,752(33,196,890-36,796,536) | 6,048.84(5,751.23-6,374.85) | 40,249,514(38,264,713-42,486,123) | 7,100.42(6,750.28-7,494.98) | 0.52 (0.5 to 0.53) | 0.58(0.55 to 0.62) | 0.89(0.86 to 0.92) | 0.14(0.12 to 0.15) |
| Low-middle SDI | 66,485,549(63,106,744-70,160,675) | 14,082.58(13,366.9-14,861.02) | 89,346,497(84,850,421-94,561,863) | 15,408.79(14,633.39-16,308.23) | 0.29 (0.28 to 0.3) | 0.27(0.24 to 0.3) | 0.3(0.29 to 0.31) | 0.29(0.28 to 0.31) |
| Low SDI | 51,265,857(48,815,544-54,076,267) | 22,395.3(21,324.89-23,623.02) | 106,215,457(100,638,972-112,235,990) | 23,079(21,867.31-24,387.17) | 0.1 (0.08 to 0.11) | 0.03(0.02 to 0.04) | 0.11(0.1 to 0.12) | 0.13(0.1 to 0.16) |
| **GBD region** |  |  |  |  |  |  |  |  |
| Andean Latin America | 716,272(676,584-760,571) | 4,822.72(4,555.5-5,120.99) | 882,807(835,132-933,239) | 4,878.79(4,615.31-5,157.5) | 0.04(0.03 to 0.05) | 0.02(0.02 to 0.03) | -0.02(-0.03 to -0.01) | 0.1(0.08 to 0.11) |
| Australasia | 675,653(641,075-714,924) | 14,733.08(13,979.1-15,589.41) | 835,101(789,543-884,603) | 14,571.27(13,776.36-15,435) | -0.03(-0.05 to -0.02) | -0.11(-0.13 to -0.09) | 0.16(0.13 to 0.2) | -0.17(-0.18 to -0.15) |
| Caribbean | 540,039(511,615-571,185) | 4,732.03(4,482.96-5,004.94) | 549,331(518,975-582,858) | 4,774.68(4,510.83-5,066.09) | 0.03(0.02 to 0.03) | -0.03(-0.04 to -0.01) | 0.02(0.01 to 0.02) | 0.09(0.07 to 0.1) |
| Central Asia | 1,732,207(1,631,881-1,845,899) | 6,931.26(6,529.82-7,386.19) | 1,961,649(1,849,184-2,094,198) | 7,087.96(6,681.59-7,566.89) | 0.07(0.05 to 0.09) | -0.37(-0.4 to -0.34) | 0.46(0.42 to 0.5) | 0.12(0.09 to 0.15) |
| Central Europe | 1,539,000(1,452,301-1,628,611) | 5,219.85(4,925.79-5,523.78) | 922,920(873,107-978,197) | 5,213.88(4,932.47-5,526.16) | -0.01(-0.02 to 0.01) | -0.21(-0.23 to -0.19) | 0.34(0.31 to 0.37) | -0.18(-0.21 to -0.15) |
| Central Latin America | 2,344,690(2,229,417-2,466,842) | 3,641.88(3,462.84-3,831.62) | 2,253,115(2,142,299-2,375,012) | 3,549.05(3,374.5-3,741.06) | -0.08(-0.1 to -0.06) | 0.19(0.17 to 0.21) | -0.31(-0.35 to -0.28) | -0.08(-0.11 to -0.06) |
| Central Sub-Saharan Africa | 6,659,050(6,219,564-7,104,734) | 26,321.79(24,584.59-28,083.48) | 15,428,054(14,411,984-16,502,164) | 26,291.12(24,559.62-28,121.52) | 0(-0.01 to 0.01) | -0.03(-0.04 to -0.02) | 0.08(0.07 to 0.1) | -0.07(-0.09 to -0.04) |
| East Asia | 9,168,813(8,655,962-9,768,203) | 2,779.83(2,624.34-2,961.55) | 6,997,759(6,606,304-7,453,346) | 2,617.43(2,471.01-2,787.84) | -0.19(-0.2 to -0.18) | -0.07(-0.09 to -0.06) | -0.23(-0.25 to -0.21) | -0.21(-0.23 to -0.19) |
| Eastern Europe | 5,685,587(5,363,996-6,011,639) | 11,048.13(10,423.22-11,681.71) | 3,877,061(3,650,481-4,109,899) | 10,938.54(10,299.28-11,595.46) | -0.04(-0.06 to -0.01) | -0.45(-0.49 to -0.41) | 0.61(0.59 to 0.63) | -0.23(-0.27 to -0.18) |
| Eastern Sub-Saharan Africa | 25,739,541(24,534,572-27,086,658) | 28,419.27(27,088.85-29,906.63) | 50,992,784(48,483,075-53,749,856) | 28,578.35(27,171.81-30,123.52) | 0.02(0.01 to 0.03) | 0(-0.01 to 0) | 0.02(0.02 to 0.03) | 0.04(0.02 to 0.05) |
| High-income Asia Pacific | 1,596,791(1,519,839-1,672,692) | 4,536.4(4,317.78-4,752.03) | 1,048,281(997,444-1,097,698) | 4,674.49(4,447.8-4,894.85) | 0.09(0.06 to 0.12) | 0.14(0.06 to 0.22) | 0.2(0.18 to 0.21) | -0.05(-0.11 to 0.01) |
| High-income North America | 2,678,028(2,495,270-2,864,423) | 4,341.97(4,045.66-4,644.18) | 2,845,052(2,638,670-3,026,032) | 4,335.72(4,021.2-4,611.52) | 0(-0.04 to 0.05) | -1.49(-1.52 to -1.46) | 0.48(0.35 to 0.61) | 0.75(0.7 to 0.8) |
| North Africa and Middle East | 12,782,989(12,204,861-13,428,403) | 9,099.16(8,687.64-9,558.57) | 16,770,201(15,942,165-17,659,955) | 9,147.95(8,696.26-9,633.3) | 0.02(0 to 0.03) | 0(-0.01 to 0.01) | 0.04(0.02 to 0.07) | 0(-0.03 to 0.03) |
| Oceania | 80,743(76,043-86,397) | 3,012.95(2,837.57-3,223.94) | 158,874(149,558-169,095) | 3,126.9(2,943.56-3,328.07) | 0.12(0.11 to 0.13) | 0.09(0.08 to 0.1) | 0.13(0.11 to 0.15) | 0.13(0.12 to 0.15) |
| South Asia | 69,969,502(66,216,407-73,977,875) | 16,145.75(15,279.70-17,070.69) | 84,546,060(80,262,377-89,744,721) | 16,675.04(15,830.17-17,700.38) | 0.1(0.1 to 0.11) | 0.08(0.07 to 0.08) | 0.05(0.04 to 0.07) | 0.17(0.16 to 0.18) |
| Southeast Asia | 2,268,025(2,142,034-2,416,384) | 1,328.29(1,254.50-1,415.18) | 2,313,896(2,178,152-2,466,248) | 1,340.2(1,261.57-1,428.44) | 0.03(0.01 to 0.05) | 0.14(0.09 to 0.19) | -0.38(-0.42 to -0.34) | 0.3(0.27 to 0.33) |
| Southern Latin America | 1,322,811(1,250,710-1,397,386) | 8,862.21(8,379.16-9,361.82) | 1,286,385(1,216,563-1,365,339) | 8,874.35(8,392.68-9,419.03) | 0(-0.03 to 0.03) | -0.03(-0.07 to 0.01) | 0.17(0.14 to 0.2) | -0.15(-0.22 to -0.07) |
| Southern Sub-Saharan Africa | 5,235,108(4,954,247-5,530,982) | 25,303.43(23,945.91-26,733.51) | 6,069,941(5,751,414-6,418,709) | 25,222.21(23,898.65-26,671.44) | -0.01(-0.01 to 0) | -0.1(-0.1 to -0.09) | 0.11(0.1 to 0.11) | -0.03(-0.05 to -0.02) |
| Tropical Latin America | 2,295,653(2,171,506-2,420,771) | 4,281.82(4,050.26-4,515.19) | 2,178,898(2,065,511-2,301,333) | 4,341.04(4,115.13-4,584.96) | 0.06(0 to 0.12) | -0.02(-0.05 to 0.01) | -0.35(-0.47 to -0.24) | 0.47(0.34 to 0.6) |
| Western Europe | 4,441,535(4,247,374-4,660,728) | 6,254.07(5,980.68-6,562.72) | 4,263,402(4,073,598-4,466,452) | 6,258.82(5,980.18-6,556.9) | -0.01(-0.06 to 0.04) | -0.02(-0.1 to 0.05) | 0.02(-0.03 to 0.07) | 0(-0.12 to 0.12) |
| Western Sub-Saharan Africa | 21,717,264(20,636,528-22,907,691) | 24,712.46(23,482.67-26,067.06) | 52,929,728(50,208,690-55,851,470) | 24,645.56(23,378.57-26,006.01) | -0.01(-0.02 to 0) | -0.07(-0.09 to -0.06) | 0.07(0.06 to 0.07) | -0.03(-0.05 to 0) |
| **204 countries and territories** |  |  |  |  |  |  |  |  |
| China | 8,445,881(7,969,649-9,016,737) | 2,652.8(2,503.22-2,832.1) | 6,505,469(6,135,926-6,936,484) | 2,505.65(2,363.32-2,671.66) | -0.18(-0.2 to -0.17) | -0.09(-0.11 to -0.07) | -0.33(-0.36 to -0.3) | -0.07(-0.1 to -0.04) |
| Democratic People's Republic of Korea | 292,288(273,942-310,808) | 4,913.02(4,604.63-5,224.31) | 242,862(227,076-259,844) | 5,087.49(4,756.81-5,443.23) | 0.11(0.1 to 0.13) | 0.05(0.04 to 0.07) | 0.15(0.11 to 0.18) | 0.14(0.12 to 0.16) |
| Taiwan (Province of China) | 430,643(405,685-456,697) | 7,818.33(7,365.21-8,291.33) | 249,428(235,593-265,598) | 8,464.54(7,995.03-9,013.29) | 0.26(0.24 to 0.27) | 0.22(0.2 to 0.23) | 0.24(0.23 to 0.26) | 0.28(0.25 to 0.32) |
| Cambodia | 68,399(64,783-72,346) | 1,467.49(1,389.92-1,552.18) | 76,192(71,807-80,963) | 1,489.13(1,403.43-1,582.37) | 0.05(0.03 to 0.06) | -0.07(-0.1 to -0.04) | 0.15(0.13 to 0.17) | 0.05(0.04 to 0.06) |
| Indonesia | 407,005(374,309-442,692) | 600.86(552.59-653.54) | 372,195(342,015-403,324) | 553.13(508.28-599.4) | -0.27(-0.3 to -0.24) | 0.07(0.06 to 0.08) | -1.21(-1.31 to -1.11) | 0.17(0.15 to 0.19) |
| Lao People's Democratic Republic | 27,054(25,664-28,588) | 1,467.86(1,392.45-1,551.09) | 34,441(32,433-36,514) | 1,499.8(1,412.35-1,590.09) | 0.07(0.06 to 0.08) | 0.06(0.04 to 0.07) | 0.04(0.03 to 0.05) | 0.11(0.09 to 0.13) |
| Malaysia | 95,486(90,012-101,419) | 1,452.7(1,369.42-1,542.96) | 112,816(106,511-119,640) | 1,481.77(1,398.94-1,571.39) | 0.06(0.05 to 0.07) | 0.06(0.05 to 0.06) | 0.06(0.05 to 0.07) | 0.07(0.05 to 0.09) |
| Maldives | 1,540(1,457-1,631) | 1,466.2(1,386.6-1,553.06) | 1,481(1,395-1,562) | 1,478.27(1,392.95-1,559.07) | 0.02(0.01 to 0.04) | -0.16(-0.17 to -0.14) | 0.22(0.19 to 0.26) | -0.01(-0.04 to 0.02) |
| Myanmar | 214,803(202,628-227,732) | 1,453.7(1,371.3-1,541.2) | 232,803(220,300-246,253) | 1,490.91(1,410.84-1,577.05) | 0.08(0.08 to 0.09) | 0.08(0.08 to 0.08) | 0.1(0.1 to 0.11) | 0.06(0.05 to 0.07) |
| Philippines | 417,786(398,334-440,874) | 1,656.94(1,579.79-1,748.50) | 576,256(548,359-607,628) | 1,694.94(1,612.89-1,787.22) | 0.07(0.07 to 0.08) | 0.03(0.03 to 0.04) | 0.09(0.08 to 0.11) | 0.08(0.06 to 0.09) |
| Sri Lanka | 79,532(74,912-84,142) | 1,437.4(1,353.9-1,520.71) | 75,330(70,889-79,944) | 1,475.91(1,388.89-1,566.3) | 0.09(0.08 to 0.1) | 0.15(0.13 to 0.17) | 0.17(0.14 to 0.19) | -0.01(-0.03 to 0) |
| Thailand | 242,991(228,690-257,275) | 1,441.35(1,356.52-1,526.08) | 143,645(134,753-152,444) | 1,470.83(1,379.78-1,560.91) | 0.07(0.06 to 0.08) | 0.08(0.07 to 0.09) | 0.08(0.06 to 0.09) | 0.04(0.02 to 0.07) |
| Timor-Leste | 4,919(4,668-5,202) | 1,478.79(1,403.41-1,563.85) | 7,785(7,383-8,244) | 1,495.26(1,418.02-1,583.5) | 0.04(0.03 to 0.04) | 0.03(0.03 to 0.04) | -0.01(-0.02 to 0) | 0.08(0.07 to 0.09) |
| Viet Nam | 700,123(652,044-762,484) | 2,640.73(2,459.39-2,875.95) | 674,305(622,441-729,453) | 2,723.07(2,513.63-2,945.78) | 0.1(0.08 to 0.12) | -0.06(-0.1 to -0.02) | 0.42(0.38 to 0.46) | -0.05(-0.08 to -0.02) |
| Fiji | 8,380(7,859-8,945) | 2,977.61(2,792.5-3,178.37) | 8,403(7,863-9,006) | 3,083.3(2,885.33-3,304.8) | 0.11(0.11 to 0.12) | 0.13(0.12 to 0.15) | 0.16(0.15 to 0.16) | 0.06(0.05 to 0.07) |
| Kiribati | 899(841-957) | 3,042.93(2,848.45-3,238.78) | 1,297(1,215-1,388) | 3,087.22(2,891.71-3,304.55) | 0.04(0.03 to 0.06) | -0.07(-0.08 to -0.05) | 0.13(0.12 to 0.15) | 0.05(0.01 to 0.09) |
| Marshall Islands | 654(611-702) | 2,979.39(2,785.1-3,200.05) | 536(501-572) | 3,068.95(2,871.62-3,273.82) | 0.1(0.09 to 0.1) | 0.12(0.11 to 0.13) | 0.12(0.11 to 0.13) | 0.05(0.04 to 0.06) |
| Micronesia (Federated States of) | 1,368(1,280-1,461) | 2,979.67(2,787.03-3,182.23) | 935(873-1,002) | 3,053.87(2,852.39-3,276.03) | 0.08(0.07 to 0.08) | 0.04(0.03 to 0.04) | 0.08(0.08 to 0.08) | 0.11(0.09 to 0.13) |
| Papua New Guinea | 51,319(48,459-55,041) | 3,018.64(2,850.45-3,237.57) | 122,827(115,377-131,058) | 3,135.71(2,945.51-3,345.83) | 0.12(0.12 to 0.13) | 0.08(0.08 to 0.09) | 0.13(0.12 to 0.14) | 0.15(0.14 to 0.16) |
| Samoa | 2,135(1,998-2,280) | 2,995.64(2,804.26-3,199.6) | 2,477(2,328-2,644) | 3,098.84(2,912.03-3,307.19) | 0.11(0.1 to 0.12) | 0.08(0.07 to 0.09) | 0.14(0.11 to 0.17) | 0.11(0.09 to 0.12) |
| Solomon Islands | 4,698(4,395-5,017) | 3,017.1(2,822.88-3,222.21) | 8,065(7,589-8,607) | 3,101.25(2,918.41-3,309.88) | 0.09(0.08 to 0.09) | 0.14(0.14 to 0.15) | 0.08(0.07 to 0.1) | 0.05(0.04 to 0.06) |
| Tonga | 1,255(1,179-1,342) | 3,002.7(2,820.57-3,210.66) | 1,209(1,133-1,288) | 3,097.93(2,902.38-3,301.62) | 0.1(0.09 to 0.11) | 0.1(0.09 to 0.1) | 0.11(0.11 to 0.12) | 0.09(0.08 to 0.11) |
| Vanuatu | 2,063(1,940-2,202) | 3,030.12(2,848.69-3,233.51) | 3,613(3,397-3,847) | 3,099.84(2,914.42-3,300.59) | 0.07(0.07 to 0.08) | 0.05(0.04 to 0.06) | 0.12(0.12 to 0.13) | 0.05(0.04 to 0.07) |
| Armenia | 78,070(71,831-85,957) | 7,483.65(6,885.55-8,239.65) | 44,224(40,584-48,482) | 7,465.73(6,851.10-8,184.52) | -0.01(-0.07 to 0.04) | -0.57(-0.66 to -0.48) | 0.56(0.46 to 0.66) | -0.08(-0.15 to -0.01) |
| Azerbaijan | 166,127(155,853-177,739) | 6,845.11(6,421.81-7,323.59) | 160,794(149,941-172,622) | 6,811.63(6,351.87-7,312.68) | -0.02(-0.03 to 0) | -0.49(-0.52 to -0.46) | 0.48(0.44 to 0.52) | -0.04(-0.07 to -0.01) |
| Georgia | 58,903(54,769-63,623) | 4,303.53(4,001.5-4,648.42) | 31,558(29,229-34,124) | 4,288.14(3,971.71-4,636.76) | -0.01(-0.04 to 0.01) | -0.41(-0.45 to -0.36) | 0.31(0.25 to 0.37) | 0(-0.01 to 0.02) |
| Kazakhstan | 353,099(331,826-378,111) | 6,795.29(6,385.89-7,276.64) | 375,176(351,096-401,541) | 6,913.8(6,470.05-7,399.65) | 0.05(0.02 to 0.08) | -0.45(-0.48 to -0.41) | 0.65(0.58 to 0.72) | -0.05(-0.1 to 0) |
| Kyrgyzstan | 161,457(150,210-175,113) | 9,624.44(8,953.98-10,438.44) | 218,408(202,297-236,614) | 9,602.94(8,894.57-10,403.41) | -0.01(-0.04 to 0.03) | -0.53(-0.57 to -0.49) | 0.52(0.44 to 0.6) | 0(-0.06 to 0.06) |
| Mongolia | 61,754(58,109-65,647) | 6,861.56(6,456.55-7,294.11) | 75,325(70,366-80,355) | 6,932.11(6,475.77-7,395.05) | 0.03(0.01 to 0.06) | -0.45(-0.49 to -0.41) | 0.51(0.47 to 0.56) | 0.03(-0.01 to 0.06) |
| Tajikistan | 160,639(151,240-171,561) | 6,917.98(6,513.21-7,388.35) | 248,658(233,606-264,756) | 6,937.3(6,517.35-7,386.42) | 0.01(-0.01 to 0.02) | -0.39(-0.42 to -0.37) | 0.28(0.25 to 0.31) | 0.11(0.08 to 0.14) |
| Turkmenistan | 103,244(96,230-110,271) | 6,878.97(6,411.68-7,347.16) | 104,943(98,353-111,839) | 6,886(6,453.59-7,338.53) | 0(-0.02 to 0.02) | -0.32(-0.35 to -0.29) | 0.19(0.16 to 0.22) | 0.11(0.08 to 0.14) |
| Uzbekistan | 588,914(550,102-626,957) | 6,883.35(6,429.7-7,328) | 702,563(659,818-751,512) | 6,962.22(6,538.63-7,447.3) | 0.04(0.02 to 0.05) | -0.43(-0.46 to -0.4) | 0.3(0.27 to 0.33) | 0.2(0.17 to 0.23) |
| Albania | 59,543(56,062-63,268) | 5,329.4(5,017.87-5,662.83) | 23,459(22,018-25,046) | 5,287.36(4,962.48-5,645.03) | -0.03(-0.04 to -0.01) | -0.24(-0.26 to -0.22) | -0.13(-0.15 to -0.11) | 0.22(0.19 to 0.25) |
| Bosnia and Herzegovina | 57,240(53,950-60,587) | 5,224.57(4,924.27-5,530.02) | 25,795(24,254-27,554) | 5,259.46(4,945.33-5,618.09) | 0.02(0 to 0.04) | -0.2(-0.25 to -0.15) | 0.13(0.11 to 0.15) | 0.09(0.05 to 0.12) |
| Bulgaria | 90,154(85,146-95,256) | 5,192.52(4,904.08-5,486.37) | 51,400(48,378-54,690) | 5,266.05(4,956.42-5,603.09) | 0.05(0.03 to 0.07) | -0.22(-0.25 to -0.2) | 0.56(0.52 to 0.6) | -0.16(-0.17 to -0.14) |
| Croatia | 42,657(40,288-45,036) | 4,321.87(4,081.81-4,562.83) | 25,941(24,535-27,541) | 4,344.25(4,108.67-4,612.14) | 0.02(0.01 to 0.03) | 0.05(0.03 to 0.07) | 0.05(0.04 to 0.07) | -0.04(-0.05 to -0.04) |
| Czechia | 139,931(129,079-150,765) | 6,349.86(5,857.41-6,841.46) | 112,721(104,775-121,325) | 6,567.48(6,104.52-7,068.79) | 0.12(0.06 to 0.17) | -0.12(-0.24 to -0.01) | 0.62(0.51 to 0.73) | -0.09(-0.15 to -0.02) |
| Hungary | 109,576(103,299-116,604) | 5,141.82(4,847.26-5,471.6) | 73,695(69,364-78,010) | 5,307.52(4,995.58-5,618.25) | 0.11(0.08 to 0.13) | 0.05(0.02 to 0.09) | 0.27(0.21 to 0.33) | 0.05(0.02 to 0.07) |
| North Macedonia | 27,547(25,961-29,370) | 5,229.43(4,928.22-5,575.37) | 17,217(16,132-18,352) | 5,255.71(4,924.39-5,602.25) | 0.02(0.01 to 0.03) | -0.11(-0.12 to -0.09) | 0.08(0.07 to 0.1) | 0.05(0.03 to 0.07) |
| Montenegro | 8,456(7,975-8,980) | 5,232.69(4,935.04-5,557.08) | 5,898(5,584-6,285) | 5,293.86(5,011.46-5,641.09) | 0.04(0.03 to 0.05) | 0(-0.01 to 0.02) | 0.05(0.03 to 0.06) | 0.04(0.03 to 0.06) |
| Poland | 384,800(365,055-406,239) | 4,018.04(3,811.86-4,241.9) | 227,118(215,960-238,600) | 3,858.8(3,669.22-4,053.88) | -0.13(-0.14 to -0.11) | -0.23(-0.25 to -0.21) | 0.27(0.25 to 0.3) | -0.44(-0.46 to -0.41) |
| Romania | 404,892(375,767-437,467) | 7,271.71(6,748.64-7,856.75) | 223,802(208,419-240,335) | 7,435.25(6,924.2-7,984.53) | 0.07(0.06 to 0.08) | -0.16(-0.18 to -0.13) | 0.55(0.54 to 0.57) | -0.16(-0.19 to -0.13) |
| Serbia | 85,830(80,251-91,715) | 3,957.41(3,700.16-4,228.77) | 51,924(48,438-55,648) | 3,910.45(3,647.89-4,190.87) | -0.05(-0.07 to -0.03) | -0.09(-0.15 to -0.04) | 0.12(0.09 to 0.14) | -0.14(-0.17 to -0.12) |
| Slovakia | 83,565(78,301-88,523) | 6,303.59(5,906.46-6,677.6) | 55,000(51,663-58,809) | 6,421.04(6,031.44-6,865.72) | 0.06(0.04 to 0.08) | -0.21(-0.24 to -0.18) | 0.33(0.3 to 0.35) | 0.02(-0.01 to 0.05) |
| Slovenia | 20,196(18,974-21,581) | 4,883.88(4,588.3-5,218.8) | 15,516(14,571-16,564) | 4,968.83(4,666.09-5,304.45) | 0.06(0.04 to 0.07) | -0.09(-0.11 to -0.06) | 0.42(0.39 to 0.46) | -0.16(-0.18 to -0.13) |
| Belarus | 264,623(248,020-280,628) | 11,008.22(10,317.51-11,674.01) | 172,729(162,075-185,377) | 10,944.85(10,269.79-11,746.24) | -0.02(-0.04 to 0) | -0.43(-0.45 to -0.41) | 0.49(0.46 to 0.53) | -0.17(-0.19 to -0.14) |
| Estonia | 38,599(36,216-40,975) | 11,056.62(10,373.98-11,737.14) | 23,851(22,322-25,379) | 11,035.55(10,327.94-11,742.49) | -0.01(-0.06 to 0.03) | -0.46(-0.49 to -0.43) | 0.57(0.52 to 0.62) | -0.14(-0.23 to -0.04) |
| Latvia | 58,170(54,736-62,070) | 10,223.88(9,620.37-10,909.33) | 30,240(28,248-32,526) | 10,179.87(9,509.24-10,949.48) | -0.02(-0.05 to 0.02) | -0.49(-0.53 to -0.45) | 0.71(0.66 to 0.75) | -0.22(-0.23 to -0.2) |
| Lithuania | 99,808(93,156-107,277) | 12,015.04(11,214.16-12,914.15) | 48,861(45,911-52,308) | 11,982.01(11,258.48-12,827.23) | -0.01(-0.03 to 0) | -0.21(-0.24 to -0.17) | 0.21(0.19 to 0.23) | -0.07(-0.09 to -0.04) |
| Republic of Moldova | 136,971(129,528-145,949) | 11,082.48(10,480.29-11,808.92) | 57,068(53,375-60,994) | 10,926.14(10,218.93-11,677.8) | -0.05(-0.06 to -0.03) | -0.48(-0.49 to -0.47) | 0.34(0.32 to 0.37) | -0.08(-0.11 to -0.04) |
| Russian Federation | 3,833,349(3,614,269-4,057,578) | 11,047.58(10,416.2-11,693.8) | 2,859,746(2,692,577-3,028,074) | 10,966.39(10,325.34-11,611.89) | -0.03(-0.05 to 0) | -0.47(-0.52 to -0.43) | 0.62(0.58 to 0.65) | -0.21(-0.25 to -0.18) |
| Ukraine | 1,254,067(1,178,571-1,324,020) | 11,024.86(10,361.16-11,639.84) | 684,565(642,351-732,844) | 10,788.7(10,123.41-11,549.58) | -0.07(-0.1 to -0.04) | -0.4(-0.44 to -0.36) | 0.54(0.48 to 0.6) | -0.36(-0.41 to -0.32) |
| Brunei Darussalam | 4,315(4,102-4,542) | 4,764.25(4,528.73-5,014.02) | 4,384(4,160-4,623) | 4,633.98(4,397.93-4,887.11) | -0.09(-0.11 to -0.07) | -0.17(-0.21 to -0.13) | -0.21(-0.26 to -0.16) | 0.11(0.09 to 0.13) |
| Japan | 1,047,202(997,840-1,097,682) | 4,535.5(4,321.71-4,754.13) | 732,488(698,084-766,162) | 4,742.75(4,519.99-4,960.79) | 0.13(0.09 to 0.18) | 0.08(0.03 to 0.12) | 0.54(0.43 to 0.66) | -0.16(-0.2 to -0.11) |
| Republic of Korea | 515,645(487,448-543,642) | 4,534.91(4,286.93-4,781.14) | 272,750(258,913-288,013) | 4,490.09(4,262.30-4,741.36) | -0.02(-0.07 to 0.02) | 0.35(0.26 to 0.43) | -0.47(-0.52 to -0.41) | 0.08(0.02 to 0.15) |
| Singapore | 29,629(28,052-31,159) | 4,562.72(4,319.91-4,798.29) | 38,659(36,573-40,671) | 4,760.37(4,503.58-5,008.12) | 0.14(0.11 to 0.17) | 0.35(0.27 to 0.43) | -0.25(-0.28 to -0.21) | 0.37(0.31 to 0.42) |
| Australia | 569,883(539,490-603,265) | 15,053.05(14,250.24-15,934.82) | 708,610(669,646-752,097) | 14,919.91(14,099.51-15,835.53) | -0.03(-0.04 to -0.01) | -0.07(-0.09 to -0.05) | 0.16(0.14 to 0.17) | -0.18(-0.22 to -0.15) |
| New Zealand | 105,770(98,414-113,779) | 13,219.13(12,299.82-14,220.07) | 126,490(118,548-134,967) | 12,884.57(12,075.59-13,747.99) | -0.08(-0.1 to -0.06) | -0.27(-0.3 to -0.24) | 0.11(0.06 to 0.15) | -0.1(-0.12 to -0.08) |
| Andorra | 540(516-565) | 5,679.75(5,430.13-5,950.38) | 564(538-592) | 5,545.88(5,295.14-5,827.37) | -0.1(-0.17 to -0.03) | 0.47(0.26 to 0.68) | -0.19(-0.24 to -0.14) | -0.52(-0.6 to -0.44) |
| Austria | 97,181(92,550-102,157) | 7,207.39(6,863.91-7,576.4) | 93,924(89,594-98,625) | 7,241.48(6,907.71-7,603.96) | 0.01(-0.01 to 0.03) | -0.2(-0.24 to -0.16) | 0.09(0.04 to 0.13) | 0.13(0.1 to 0.17) |
| Belgium | 96,617(92,575-101,497) | 5,349.41(5,125.60-5,619.62) | 101,794(97,238-106,626) | 5,323.68(5,085.41-5,576.38) | -0.02(-0.05 to 0) | -0.08(-0.15 to -0.02) | 0.15(0.13 to 0.17) | -0.12(-0.15 to -0.09) |
| Cyprus | 15,723(15,030-16,555) | 7,943.34(7,593.53-8,363.85) | 17,689(16,870-18,670) | 8,088.42(7,713.97-8,536.99) | 0.06(0.03 to 0.08) | -0.36(-0.39 to -0.33) | 0.26(0.2 to 0.33) | 0.22(0.18 to 0.26) |
| Denmark | 82,372(78,244-86,967) | 9,324.94(8,857.63-9,845.09) | 89,712(85,513-94,707) | 9,402.22(8,962.08-9,925.63) | 0(-0.06 to 0.06) | 0.46(0.38 to 0.55) | -0.42(-0.54 to -0.3) | 0.03(-0.05 to 0.11) |
| Finland | 74,696(71,088-78,751) | 7,740.99(7,367.12-8,161.26) | 63,702(60,618-67,369) | 7,520.07(7,156.07-7,952.99) | -0.11(-0.16 to -0.06) | -0.22(-0.29 to -0.16) | 0.17(0.14 to 0.21) | -0.27(-0.4 to -0.14) |
| France | 692,905(660,088-729,128) | 5,915.09(5,634.94-6,224.31) | 675,369(642,051-709,581) | 5,818.32(5,531.28-6,113.06) | -0.06(-0.1 to -0.03) | -0.15(-0.23 to -0.08) | 0.27(0.19 to 0.35) | -0.28(-0.31 to -0.25) |
| Germany | 1,277,247(1,211,840-1,355,351) | 9,866.08(9,360.84-10,469.39) | 1,189,343(1,126,944-1,259,540) | 9,940.5(9,418.97-10,527.2) | 0.02(-0.04 to 0.08) | -0.3(-0.4 to -0.21) | 0.18(0.15 to 0.2) | 0.19(0.05 to 0.33) |
| Greece | 102,250(97,676-107,877) | 5,052.94(4,826.89-5,331.03) | 72,541(69,263-76,285) | 5,201(4,966-5,469.43) | 0.09(0.07 to 0.1) | 0.12(0.1 to 0.15) | 0.48(0.46 to 0.51) | -0.3(-0.33 to -0.27) |
| Iceland | 3,182(3,041-3,337) | 5,016.78(4,793.87-5,260.51) | 3,410(3,250-3,572) | 5,049.24(4,811.54-5,288.6) | 0(-0.03 to 0.04) | 0.03(-0.01 to 0.06) | 0.07(0.01 to 0.14) | -0.07(-0.14 to 0) |
| Ireland | 56,417(53,475-59,482) | 5,742.11(5,442.69-6,054.05) | 57,878(55,218-60,882) | 5,804.06(5,537.36-6,105.29) | 0.03(-0.02 to 0.08) | 0.1(-0.04 to 0.24) | 0.4(0.37 to 0.44) | -0.4(-0.43 to -0.36) |
| Israel | 90,964(86,058-95,788) | 5,933.49(5,613.46-6,248.18) | 158,569(151,826-166,682) | 6,034.43(5,777.81-6,343.16) | 0.05(0.01 to 0.08) | 0.08(0.04 to 0.11) | 0.1(0.02 to 0.18) | -0.05(-0.09 to 0) |
| Italy | 318,562(304,934-332,299) | 3,451.46(3,303.8-3,600.29) | 256,680(245,785-268,610) | 3,377.42(3,234.06-3,534.4) | -0.08(-0.12 to -0.05) | 0.06(-0.03 to 0.14) | 0.06(-0.02 to 0.14) | -0.29(-0.32 to -0.27) |
| Luxembourg | 3,660(3,493-3,835) | 5,539.83(5,286.65-5,804.56) | 5,546(5,301-5,827) | 5,478.60(5,236.21-5,755.72) | -0.05(-0.08 to -0.02) | -0.07(-0.13 to -0.02) | -0.23(-0.25 to -0.2) | 0.1(0.05 to 0.16) |
| Malta | 4,374(4,172-4,586) | 4,997.99(4,767.13-5,239.41) | 3,271(3,123-3,415) | 5,109.79(4,878.15-5,334.95) | 0.07(0.03 to 0.1) | -0.29(-0.36 to -0.22) | 0.08(0.02 to 0.15) | 0.33(0.28 to 0.37) |
| Netherlands | 163,517(156,931-172,036) | 6,000.02(5,758.36-6,312.62) | 157,966(150,960-165,804) | 5,889.83(5,628.61-6,182.06) | -0.08(-0.11 to -0.04) | -0.1(-0.13 to -0.06) | -0.1(-0.2 to -0.01) | -0.01(-0.05 to 0.02) |
| Norway | 52,408(50,231-55,012) | 6,564.57(6,291.92-6,890.76) | 58,479(55,892-61,568) | 6,330.32(6,050.21-6,664.7) | -0.13(-0.18 to -0.07) | -0.05(-0.14 to 0.04) | -0.48(-0.53 to -0.42) | 0.15(0.03 to 0.28) |
| Portugal | 107,395(101,991-114,045) | 5,075.62(4,820.22-5,389.91) | 71,514(67,980-75,290) | 5,249.67(4,990.23-5,526.86) | 0.11(0.09 to 0.12) | 0.29(0.28 to 0.3) | 0.05(0.02 to 0.09) | 0.01(-0.01 to 0.04) |
| Spain | 383,954(365,443-404,761) | 4,899.85(4,663.62-5,165.39) | 328,716(312,687-345,668) | 5,072.17(4,824.84-5,333.73) | 0.11(0.07 to 0.15) | 0.39(0.34 to 0.44) | 0.61(0.56 to 0.66) | -0.55(-0.61 to -0.49) |
| Sweden | 96,439(91,871-101,563) | 6,244.26(5,948.5-6,576.03) | 110,578(105,456-116,130) | 6,073.28(5,791.93-6,378.22) | -0.1(-0.17 to -0.04) | -0.66(-0.75 to -0.58) | 0.6(0.44 to 0.77) | -0.16(-0.22 to -0.11) |
| Switzerland | 73,466(70,061-77,328) | 6,357.16(6,062.51-6,691.31) | 84,236(80,274-88,998) | 6,321.64(6,024.33-6,679.04) | -0.03(-0.06 to 0.01) | -0.36(-0.41 to -0.3) | 0.11(0.05 to 0.18) | 0.13(0.06 to 0.19) |
| United Kingdom | 643,575(616,405-672,161) | 5,893.29(5,644.49-6,155.05) | 657,621(630,885-686,617) | 5,581.08(5,354.18-5,827.16) | -0.19(-0.24 to -0.13) | -0.58(-0.65 to -0.51) | 0.35(0.31 to 0.39) | -0.37(-0.5 to -0.24) |
| Argentina | 1,040,618(979,050-1,103,172) | 10,267.33(9,659.86-10,884.52) | 1,039,628(978,757-1,109,765) | 10,209.08(9,611.33-10,897.82) | -0.02(-0.05 to 0) | 0.01(0.01 to 0.02) | 0.01(0.01 to 0.02) | -0.12(-0.18 to -0.05) |
| Chile | 220,807(210,019-233,307) | 5,559.22(5,287.60-5,873.93) | 197,709(186,565-208,448) | 5,413.94(5,108.79-5,708.03) | -0.09(-0.13 to -0.05) | -0.21(-0.29 to -0.14) | -0.01(-0.09 to 0.06) | -0.07(-0.13 to -0.01) |
| Uruguay | 61,323(58,054-64,858) | 7,491.38(7,092.02-7,923.21) | 48,977(46,439-51,995) | 7,426.16(7,041.36-7,883.82) | -0.03(-0.05 to -0.02) | 0.03(0.01 to 0.05) | -0.12(-0.13 to -0.11) | 0(-0.03 to 0.04) |
| Canada | 213,395(199,498-226,139) | 3,710.13(3,468.51-3,931.69) | 227,853(210,817-242,804) | 3,691.83(3,415.80-3,934.07) | -0.02(-0.03 to 0) | -0.04(-0.06 to -0.03) | 0.02(-0.01 to 0.05) | -0.03(-0.05 to -0.01) |
| United States of America | 2,464,037(2,295,606-2,637,761) | 4,407.12(4,105.87-4,717.84) | 2,616,717(2,427,207-2,785,582) | 4,402.7(4,083.85-4,686.82) | 0(-0.03 to 0.04) | -1.64(-1.67 to -1.61) | 0.52(0.46 to 0.58) | 0.84(0.78 to 0.89) |
| Antigua and Barbuda | 856(805-911) | 4,707.03(4,426.42-5,006.51) | 801(750-856) | 4,738.13(4,439.08-5,061.79) | 0.02(0.01 to 0.03) | 0.08(0.07 to 0.09) | -0.11(-0.12 to -0.11) | 0.1(0.08 to 0.12) |
| Bahamas | 3,780(3,549-4,007) | 4,686.8(4,399.46-4,968.02) | 3,753(3,501-3,996) | 4,622.59(4,311.97-4,921.8) | -0.04(-0.06 to -0.03) | 0.03(0.01 to 0.05) | 0.09(0.06 to 0.11) | -0.17(-0.2 to -0.14) |
| Barbados | 2,937(2,757-3,114) | 4,709.39(4,420.71-4,993.65) | 2,234(2,097-2,371) | 4,745.09(4,452.19-5,034.02) | 0.02(0.01 to 0.04) | 0.09(0.07 to 0.12) | 0.04(0.03 to 0.05) | -0.04(-0.08 to 0) |
| Belize | 3,885(3,657-4,110) | 4,746.07(4,466.84-5,020.27) | 5,787(5,420-6,182) | 4,700.29(4,402.74-5,020.9) | -0.03(-0.04 to -0.02) | -0.05(-0.06 to -0.04) | 0.02(0.01 to 0.04) | -0.05(-0.07 to -0.04) |
| Cuba | 118,180(111,456-125,300) | 4,719.6(4,451.07-5,003.93) | 84,156(79,079-90,070) | 4,735.8(4,450.07-5,068.63) | 0.01(-0.01 to 0.03) | -0.15(-0.19 to -0.1) | 0.08(0.04 to 0.12) | 0.12(0.08 to 0.15) |
| Dominica | 1,174(1,105-1,254) | 4,730.92(4,454.9-5,053.44) | 642(600-682) | 4,689.52(4,386.78-4,987.7) | -0.03(-0.04 to -0.02) | -0.01(-0.02 to -0.01) | -0.02(-0.03 to 0) | -0.04(-0.05 to -0.04) |
| Dominican Republic | 127,345(120,096-135,685) | 4,724.44(4,455.51-5,033.85) | 140,047(131,447-148,587) | 4,766.01(4,473.36-5,056.65) | 0.03(0.02 to 0.04) | 0.07(0.05 to 0.08) | -0.1(-0.1 to -0.09) | 0.12(0.11 to 0.13) |
| Grenada | 1,595(1,504-1,702) | 4,775.68(4,501.99-5,094.67) | 1,041(980-1,108) | 4,767.34(4,489.89-5,077.96) | -0.01(-0.02 to 0) | -0.04(-0.06 to -0.03) | -0.03(-0.05 to -0.01) | 0.04(0.02 to 0.07) |
| Guyana | 13,877(13,034-14,762) | 4,721.08(4,434.35-5,022.35) | 10,181(9,558-10,776) | 4,771.28(4,478.96-5,049.97) | 0.04(0.02 to 0.05) | 0.17(0.14 to 0.19) | -0.31(-0.34 to -0.27) | 0.23(0.21 to 0.25) |
| Haiti | 129,937(122,345-137,961) | 4,789.28(4,509.44-5,085.03) | 210,629(197,881-223,521) | 4,838.97(4,546.08-5,135.14) | 0.03(0.01 to 0.05) | -0.09(-0.12 to -0.06) | 0.11(0.08 to 0.15) | 0.06(0.04 to 0.07) |
| Jamaica | 39,275(36,878-41,777) | 4,702.41(4,415.47-5,001.94) | 27,355(25,728-29,202) | 4,684.61(4,406.04-5,001) | -0.01(-0.03 to 0) | 0.03(0.02 to 0.04) | -0.13(-0.16 to -0.11) | 0.06(0.03 to 0.09) |
| Saint Lucia | 2,435(2,290-2,595) | 4,723.37(4,442.98-5,034.93) | 1,399(1,305-1,490) | 4,713.38(4,394.07-5,018.64) | -0.01(-0.01 to 0) | -0.02(-0.02 to -0.01) | -0.11(-0.11 to -0.1) | 0.09(0.08 to 0.1) |
| Saint Vincent and the Grenadines | 1,920(1,799-2,054) | 4,673.96(4,377.99-4,998.86) | 1,181(1,110-1,254) | 4,731.64(4,450.1-5,026.27) | 0.04(0.03 to 0.05) | 0.12(0.09 to 0.14) | -0.04(-0.05 to -0.02) | 0.04(0.02 to 0.06) |
| Suriname | 6,193(5,803-6,607) | 4,754.39(4,454.92-5,072.17) | 6,859(6,433-7,315) | 4,787.5(4,489.82-5,105.8) | 0.02(0.02 to 0.03) | -0.05(-0.06 to -0.04) | 0(-0.01 to 0.01) | 0.1(0.09 to 0.11) |
| Trinidad and Tobago | 19,217(17,981-20,453) | 4,729.53(4,425.44-5,033.71) | 12,817(11,947-13,659) | 4,704.92(4,385.44-5,013.89) | -0.02(-0.03 to -0.01) | -0.32(-0.35 to -0.29) | 0.32(0.29 to 0.34) | -0.1(-0.11 to -0.08) |
| Bolivia (Plurinational State of) | 130,573(122,844-138,777) | 4,861.55(4,573.77-5,166.98) | 170,288(160,851-181,348) | 4,884.19(4,613.5-5,201.4) | 0.01(0.01 to 0.02) | 0(-0.01 to 0) | -0.07(-0.08 to -0.06) | 0.1(0.09 to 0.12) |
| Ecuador | 184,279(174,022-196,781) | 4,767.54(4,502.16-5,090.97) | 247,102(232,056-262,743) | 4,872.96(4,576.25-5,181.4) | 0.07(0.05 to 0.09) | 0.09(0.06 to 0.12) | 0.1(0.06 to 0.15) | 0.01(0 to 0.03) |
| Peru | 401,419(378,243-428,661) | 4,835.85(4,556.65-5,164.03) | 465,417(437,241-493,856) | 4,879.91(4,584.49-5,178.1) | 0.03(0.02 to 0.03) | 0(-0.01 to 0) | -0.07(-0.07 to -0.06) | 0.14(0.13 to 0.15) |
| Colombia | 418,796(397,251-442,637) | 3,590.8(3,406.07-3,795.22) | 380,821(360,547-403,382) | 3,588.24(3,397.21-3,800.82) | 0(-0.01 to 0) | 0.05(0.04 to 0.06) | -0.12(-0.13 to -0.12) | 0.07(0.07 to 0.08) |
| Costa Rica | 40,685(38,405-43,127) | 3,619.06(3,416.25-3,836.23) | 36,533(34,428-38,711) | 3,591.22(3,384.3-3,805.33) | -0.02(-0.03 to -0.02) | -0.15(-0.16 to -0.13) | -0.06(-0.07 to -0.04) | 0.11(0.1 to 0.12) |
| El Salvador | 77,207(73,133-81,668) | 3,577.56(3,388.79-3,784.27) | 65,549(61,885-69,763) | 3,604.01(3,402.51-3,835.69) | 0.02(0 to 0.05) | 0.2(0.16 to 0.25) | -0.29(-0.33 to -0.25) | 0.13(0.1 to 0.16) |
| Guatemala | 146,811(138,679-155,262) | 3,614.95(3,414.71-3,823.04) | 177,798(167,826-188,545) | 3,603.5(3,401.39-3,821.32) | -0.01(-0.02 to 0) | 0.04(0.01 to 0.06) | -0.07(-0.08 to -0.06) | 0.02(-0.01 to 0.04) |
| Honduras | 79,982(75,766-84,692) | 3,620.03(3,429.2-3,833.22) | 117,451(111,267-124,468) | 3,583.91(3,395.22-3,798.04) | -0.03(-0.04 to -0.03) | -0.05(-0.05 to -0.04) | -0.05(-0.06 to -0.05) | 0(-0.02 to 0.01) |
| Mexico | 1,230,618(1,167,872-1,299,056) | 3,682.69(3,494.92-3,887.5) | 1,124,924(1,068,672-1,186,271) | 3,508.05(3,332.63-3,699.36) | -0.16(-0.17 to -0.14) | 0.36(0.34 to 0.38) | -0.55(-0.59 to -0.52) | -0.25(-0.27 to -0.23) |
| Nicaragua | 65,888(62,693-69,922) | 3,617.9(3,442.45-3,839.38) | 71,433(67,623-75,641) | 3,606.96(3,414.56-3,819.46) | -0.01(-0.02 to 0) | -0.05(-0.06 to -0.05) | -0.06(-0.09 to -0.03) | 0.07(0.04 to 0.09) |
| Panama | 29,946(28,234-31,749) | 3,591.05(3,385.81-3,807.24) | 41,634(39,293-44,149) | 3,609.8(3,406.81-3,827.89) | 0.02(0.01 to 0.02) | 0.02(0.01 to 0.03) | -0.07(-0.08 to -0.05) | 0.09(0.07 to 0.1) |
| Venezuela (Bolivarian Republic of) | 254,756(241,572-270,435) | 3,591.18(3,405.33-3,812.2) | 236,973(224,762-250,799) | 3,577.53(3,393.18-3,786.26) | -0.01(-0.03 to 0.01) | 0.05(0.03 to 0.06) | -0.08(-0.09 to -0.07) | 0.01(-0.04 to 0.06) |
| Brazil | 2,223,010(2,101,768-2,344,200) | 4,279.59(4,046.19-4,512.9) | 2,092,684(1,982,898-2,209,063) | 4,342.99(4,115.15-4,584.51) | 0.06(0 to 0.12) | -0.01(-0.05 to 0.02) | -0.37(-0.48 to -0.25) | 0.49(0.36 to 0.63) |
| Paraguay | 72,643(68,685-77,550) | 4,350.97(4,113.88-4,644.86) | 86,214(81,121-91,402) | 4,294.12(4,040.46-4,552.54) | -0.04(-0.05 to -0.04) | -0.18(-0.19 to -0.17) | -0.01(-0.02 to 0) | 0.03(0.02 to 0.05) |
| Algeria | 926,267(878,015-975,756) | 8,636.81(8,186.9-9,098.26) | 1,158,873(1,098,337-1,217,756) | 8,712.22(8,257.12-9,154.89) | 0.03(0.02 to 0.04) | -0.03(-0.04 to -0.02) | 0.09(0.08 to 0.09) | 0.01(-0.01 to 0.03) |
| Bahrain | 14,193(13,440-14,962) | 8,694.26(8,233.06-9,165.37) | 25,845(24,398-27,271) | 8,710.51(8,222.78-9,190.94) | 0(-0.01 to 0.02) | 0.03(0.01 to 0.05) | 0.01(-0.01 to 0.04) | -0.02(-0.04 to -0.01) |
| Egypt | 2,432,516(2,297,541-2,556,675) | 10,966.3(10,357.81-11,526.04) | 3,989,226(3,789,644-4,204,597) | 10,824.06(10,282.53-11,408.43) | -0.04(-0.06 to -0.03) | 0.02(0.01 to 0.03) | -0.21(-0.24 to -0.18) | 0.03(0 to 0.05) |
| Iran (Islamic Republic of) | 1,760,640(1,681,950-1,837,368) | 6,935.88(6,625.88-7,238.14) | 1,398,689(1,336,995-1,463,918) | 6,931.27(6,625.54-7,254.51) | 0(-0.02 to 0.01) | -0.24(-0.26 to -0.22) | 0.15(0.12 to 0.17) | 0.06(0.04 to 0.08) |
| Iraq | 607,677(576,630-646,366) | 7,377.89(7,000.94-7,847.61) | 995,681(938,527-1,059,878) | 7,396.29(6,971.72-7,873.16) | 0.01(0 to 0.02) | 0.1(0.08 to 0.13) | -0.01(-0.01 to 0) | -0.07(-0.09 to -0.06) |
| Jordan | 135,330(127,513-143,212) | 8,285.22(7,806.64-8,767.76) | 300,576(281,675-321,752) | 8,273.4(7,753.15-8,856.28) | 0(-0.01 to 0.01) | 0.06(0.03 to 0.08) | 0.03(0.02 to 0.03) | -0.07(-0.08 to -0.06) |
| Kuwait | 48,231(45,709-50,890) | 8,700.55(8,245.51-9,180.13) | 73,554(69,549-77,718) | 8,699.62(8,225.90-9,192.04) | 0(-0.01 to 0.01) | 0(-0.02 to 0.02) | 0.03(0.01 to 0.05) | -0.05(-0.08 to -0.03) |
| Lebanon | 90,150(85,615-94,617) | 8,619.52(8,185.94-9,046.64) | 110,797(104,841-116,857) | 8,669.21(8,203.15-9,143.32) | 0.02(0.01 to 0.02) | 0.02(0.01 to 0.03) | 0.05(0.05 to 0.06) | -0.03(-0.04 to -0.02) |
| Libya | 156,866(149,189-165,533) | 8,662.16(8,238.2-9,140.72) | 128,687(121,708-135,757) | 8,627.33(8,159.44-9,101.3) | -0.01(-0.02 to -0.01) | 0(-0.02 to 0.02) | 0.04(0.04 to 0.05) | -0.08(-0.09 to -0.06) |
| Morocco | 843,724(800,363-887,305) | 8,621.97(8,178.86-9,067.31) | 848,631(803,078-896,451) | 8,666.7(8,201.49-9,155.06) | 0.02(0.01 to 0.02) | 0.02(0.01 to 0.02) | 0.08(0.07 to 0.08) | -0.03(-0.04 to -0.02) |
| Palestine | 83,628(79,443-87,800) | 8,636.64(8,204.49-9,067.58) | 161,683(153,160-170,232) | 8,659.28(8,202.79-9,117.11) | 0.01(0.01 to 0.01) | 0.04(0.03 to 0.05) | 0.03(0.02 to 0.03) | -0.04(-0.04 to -0.03) |
| Oman | 72,889(69,356-76,731) | 8,673.46(8,252.98-9,130.62) | 106,937(101,800-112,310) | 8,743.74(8,323.69-9,183.09) | 0.02(0.01 to 0.04) | 0(-0.03 to 0.03) | 0.07(0.05 to 0.09) | 0(-0.02 to 0.03) |
| Qatar | 10,939(10,275-11,569) | 8,748.56(8,217.10-9,252.2) | 43,599(41,240-46,200) | 8,827.55(8,349.87-9,354.15) | 0.03(0.01 to 0.05) | -0.11(-0.12 to -0.09) | 0.14(0.11 to 0.17) | 0.04(0 to 0.09) |
| Saudi Arabia | 568,850(537,640-597,340) | 8,680.25(8,204.01-9,114.99) | 657,489(624,124-698,019) | 8,690.89(8,249.86-9,226.63) | 0(-0.01 to 0.01) | 0.02(0.01 to 0.04) | -0.03(-0.05 to -0.02) | 0(-0.01 to 0.02) |
| Syrian Arab Republic | 510,957(483,418-538,613) | 8,627.97(8,162.95-9,094.96) | 314,024(296,018-333,031) | 8,572.12(8,080.60-9,090.97) | -0.02(-0.03 to -0.01) | 0.02(0.01 to 0.03) | 0.04(0.03 to 0.04) | -0.12(-0.14 to -0.1) |
| Tunisia | 267,460(254,263-282,384) | 8,612.51(8,187.56-9,093.1) | 240,111(227,655-253,885) | 8,681.67(8,231.30-9,179.7) | 0.03(0.02 to 0.03) | -0.01(-0.02 to 0) | 0.08(0.07 to 0.08) | 0(-0.02 to 0.02) |
| Turkey | 2,447,924(2,294,899-2,608,123) | 11,947.68(11,200.8-12,729.56) | 2,219,924(2,074,985-2,376,744) | 11,986.36(11,203.77-12,833.1) | 0.01(0.01 to 0.02) | 0(-0.01 to 0) | 0.02(0.02 to 0.03) | 0.01(0 to 0.02) |
| United Arab Emirates | 51,418(48,573-54,072) | 8,724.03(8,241.28-9,174.25) | 117,057(110,703-124,411) | 8,743.41(8,268.82-9,292.69) | 0.01(-0.01 to 0.02) | -0.02(-0.04 to 0.01) | 0.02(-0.02 to 0.06) | 0(-0.02 to 0.02) |
| Yemen | 611,101(580,956-642,453) | 8,613.9(8,188.98-9,055.82) | 1,193,628(1,130,703-1,259,173) | 8,656.66(8,200.31-9,132.02) | 0.02(0.01 to 0.03) | 0.03(0.01 to 0.04) | 0.1(0.09 to 0.11) | -0.07(-0.07 to -0.06) |
| Afghanistan | 370,306(352,260-390,603) | 8,595.64(8,176.74-9,066.77) | 1,233,532(1,169,666-1,294,885) | 8,686.46(8,236.72-9,118.51) | 0.03(0.03 to 0.04) | 0.12(0.12 to 0.13) | -0.03(-0.03 to -0.02) | 0.01(0.01 to 0.01) |
| Bangladesh | 7,879,559(7,388,803-8,411,111) | 16,109.84(15,106.48-17,196.6) | 7,732,571(7,221,437-8,295,026) | 16,895.89(15,779.05-18,124.87) | 0.15(0.14 to 0.17) | 0.06(0.04 to 0.07) | 0.24(0.21 to 0.27) | 0.15(0.14 to 0.17) |
| Bhutan | 41,959(39,461-44,678) | 16,002.86(15,050.32-17,040.06) | 31,737(29,780-33,895) | 16,956.79(15,910.76-18,109.76) | 0.18(0.16 to 0.2) | 0.02(-0.01 to 0.06) | 0.2(0.17 to 0.22) | 0.28(0.26 to 0.3) |
| India | 52,744,206(49,941,010-55,783,929) | 16,153.39(15,294.89-17,084.33) | 60,501,150(57,437,737-64,321,489) | 16,512.36(15,676.28-17,555.04) | 0.07(0.06 to 0.08) | 0.08(0.05 to 0.11) | -0.02(-0.03 to 0) | 0.15(0.14 to 0.15) |
| Nepal | 1,375,937(1,286,678-1,470,398) | 16,331.24(15,271.81-17,452.43) | 1,608,376(1,505,005-1,728,121) | 17,430.77(16,310.49-18,728.51) | 0.21(0.2 to 0.22) | 0.06(0.05 to 0.08) | 0.24(0.2 to 0.27) | 0.31(0.3 to 0.32) |
| Pakistan | 7,927,841(7,504,179-8,369,179) | 16,099.74(15,239.37-16,996) | 14,672,226(13,933,829-15,504,904) | 17,172.13(16,307.92-18,146.68) | 0.21(0.19 to 0.22) | 0.14(0.12 to 0.15) | 0.24(0.2 to 0.27) | 0.23(0.2 to 0.26) |
| Angola | 1,243,751(1,152,831-1,330,883) | 26,379.74(24,451.34-28,227.8) | 4,036,667(3,776,213-4,307,122) | 26,477.81(24,769.41-28,251.81) | 0.01(0 to 0.02) | 0(-0.01 to 0.02) | 0.12(0.1 to 0.13) | -0.08(-0.1 to -0.05) |
| Central African Republic | 321,418(299,053-344,433) | 26,288.1(24,458.89-28,170.48) | 600,649(560,492-642,248) | 26,300.15(24,541.83-28,121.61) | 0(-0.01 to 0.02) | -0.02(-0.03 to -0.01) | 0.06(0.05 to 0.06) | -0.03(-0.06 to 0) |
| Congo | 274,870(257,120-294,718) | 26,104.24(24,418.53-27,989.22) | 503,595(469,132-542,142) | 26,102.82(24,316.52-28,100.85) | 0(-0.01 to 0.01) | 0.02(0.01 to 0.04) | 0.13(0.12 to 0.15) | -0.13(-0.16 to -0.11) |
| Democratic Republic of the Congo | 4,660,593(4,344,444-4,980,248) | 26,325.43(24,539.66-28,131.01) | 9,966,681(9,288,108-10,654,511) | 26,228.85(24,443.08-28,038.98) | -0.01(-0.02 to 0) | -0.04(-0.05 to -0.04) | 0.07(0.06 to 0.08) | -0.06(-0.08 to -0.05) |
| Equatorial Guinea | 51,801(48,368-55,167) | 26,306.06(24,562.61-28,015.44) | 153,100(142,697-164,157) | 26,170.98(24,392.67-28,061.04) | -0.02(-0.02 to -0.01) | -0.01(-0.01 to 0) | 0.04(0.04 to 0.05) | -0.08(-0.09 to -0.07) |
| Gabon | 106,618(99,559-113,470) | 26,163.62(24,431.46-27,845.06) | 167,363(155,151-180,626) | 26,185.67(24,274.97-28,260.81) | 0(0 to 0.01) | 0.02(0.02 to 0.03) | 0.05(0.04 to 0.06) | -0.06(-0.08 to -0.04) |
| Burundi | 718,567(676,791-761,490) | 27,414.44(25,820.61-29,052) | 1,604,618(1,508,713-1,709,819) | 27,409.88(25,771.64-29,206.92) | 0(-0.01 to 0.01) | -0.09(-0.1 to -0.09) | 0.04(0.03 to 0.04) | 0.05(0.02 to 0.08) |
| Comoros | 58,323(55,138-61,974) | 27,421.38(25,923.97-29,138.13) | 65,823(62,002-70,402) | 27,408.47(25,817.73-29,315.38) | 0(-0.01 to 0) | -0.05(-0.05 to -0.05) | -0.02(-0.02 to -0.02) | 0.06(0.05 to 0.07) |
| Djibouti | 47,687(44,916-50,638) | 27,388.3(25,797.24-29,083.43) | 113,811(106,852-121,279) | 27,545.81(25,861.46-29,353.43) | 0.02(0 to 0.03) | -0.06(-0.07 to -0.05) | 0.02(0.01 to 0.03) | 0.07(0.04 to 0.11) |
| Eritrea | 435,427(410,186-461,236) | 27,352.95(25,767.33-28,974.24) | 693,369(652,226-739,793) | 27,464.22(25,834.52-29,303.05) | 0.01(0.01 to 0.02) | -0.04(-0.05 to -0.03) | -0.02(-0.03 to -0.02) | 0.1(0.08 to 0.11) |
| Ethiopia | 7,585,567(7,282,541-7,940,921) | 31,134.45(29,890.7-32,592.98) | 14,137,095(13,543,766-14,814,309) | 31,875.89(30,538.07-33,402.85) | 0.08(0.07 to 0.08) | 0.05(0.04 to 0.07) | 0.14(0.13 to 0.14) | 0.04(0.03 to 0.05) |
| Kenya | 3,063,414(2,931,084-3,211,056) | 27,425(26,240.32-28,746.75) | 5,116,300(4,889,198-5,367,317) | 27,410.84(26,194.13-28,755.68) | 0(-0.01 to 0.01) | -0.13(-0.14 to -0.12) | 0.08(0.06 to 0.1) | 0.03(0.01 to 0.06) |
| Madagascar | 1,494,878(1,412,878-1,590,938) | 27,398.97(25,896.01-29,159.59) | 3,216,032(3,031,965-3,432,852) | 27,408.92(25,840.19-29,256.78) | 0(0 to 0.01) | -0.03(-0.04 to -0.03) | -0.02(-0.03 to -0.02) | 0.05(0.04 to 0.06) |
| Malawi | 1,249,703(1,183,271-1,324,135) | 27,468.3(26,008.13-29,104.31) | 2,217,499(2,086,753-2,352,629) | 27,296.11(25,686.71-28,959.49) | -0.02(-0.03 to -0.01) | -0.05(-0.06 to -0.03) | -0.03(-0.03 to -0.02) | 0.01(-0.01 to 0.03) |
| Mauritius | 4,764(4,501-5,082) | 1,443.39(1,363.76-1,539.69) | 3,072(2,898-3,273) | 1,481.16(1,397.40-1,577.85) | 0.08(0.07 to 0.1) | 0.12(0.1 to 0.14) | 0.03(0.02 to 0.03) | 0.1(0.07 to 0.14) |
| Mozambique | 1,693,448(1,586,991-1,797,250) | 27,295.39(25,579.48-28,968.49) | 3,922,354(3,678,306-4,185,227) | 27,494.41(25,783.71-29,337.06) | 0.02(0.02 to 0.03) | 0.01(-0.01 to 0.03) | -0.01(-0.01 to 0) | 0.07(0.05 to 0.09) |
| Rwanda | 927,790(873,605-985,402) | 27,345(25,747.98-29,043) | 1,362,541(1,278,608-1,450,629) | 27,412.98(25,724.33-29,185.21) | 0.01(-0.01 to 0.02) | -0.05(-0.07 to -0.04) | 0.01(0 to 0.02) | 0.06(0.02 to 0.1) |
| Seychelles | 344(325-364) | 1,448.04(1,368.31-1,532.91) | 348(328-367) | 1,486.48(1,402.52-1,569.37) | 0.08(0.07 to 0.09) | 0.06(0.04 to 0.07) | 0.2(0.19 to 0.21) | 0(-0.02 to 0.02) |
| Somalia | 1,065,376(1,008,076-1,133,877) | 27,347.83(25,876.94-29,106.2) | 2,845,211(2,675,537-3,029,587) | 27,544.7(25,902.08-29,329.67) | 0.02(0.01 to 0.03) | -0.03(-0.04 to -0.01) | -0.01(-0.02 to 0) | 0.1(0.08 to 0.12) |
| United Republic of Tanzania | 3,312,268(3,103,181-3,506,754) | 27,429.61(25,698.11-29,040.19) | 6,738,142(6,336,843-7,164,185) | 27,612.04(25,967.57-29,357.91) | 0.02(0.02 to 0.02) | -0.04(-0.05 to -0.04) | 0.02(0.01 to 0.02) | 0.08(0.07 to 0.09) |
| Uganda | 2,315,997(2,184,265-2,454,624) | 27,507.13(25,942.55-29,153.61) | 5,457,805(5,137,399-5,782,806) | 27,515.05(25,899.75-29,153.52) | 0(0 to 0.01) | -0.06(-0.07 to -0.05) | -0.02(-0.03 to -0.02) | 0.07(0.06 to 0.09) |
| Zambia | 1,030,456(972,853-1,095,626) | 27,445.62(25,911.4-29,181.38) | 2,277,401(2,141,768-2,420,014) | 27,532.92(25,893.16-29,257.05) | 0.01(0.01 to 0.01) | -0.04(-0.04 to -0.03) | -0.01(-0.01 to 0) | 0.07(0.06 to 0.08) |
| Botswana | 148,741(139,997-158,519) | 25,191.41(23,710.49-26,847.32) | 176,377(166,242-188,301) | 25,258.8(23,807.49-26,966.5) | 0.01(0 to 0.02) | -0.07(-0.08 to -0.07) | 0.05(0.04 to 0.06) | 0.05(0.02 to 0.07) |
| Lesotho | 171,698(161,719-183,496) | 25,153.83(23,691.94-26,882.31) | 158,245(148,754-168,787) | 25,098.88(23,593.42-26,770.84) | -0.01(-0.02 to 0) | -0.04(-0.04 to -0.03) | -0.02(-0.05 to 0) | 0.04(0.02 to 0.06) |
| Namibia | 152,359(143,935-162,308) | 25,362.27(23,959.96-27,018.46) | 208,575(195,878-221,782) | 25,268.74(23,730.58-26,868.75) | -0.01(-0.04 to 0.01) | -0.12(-0.13 to -0.11) | 0.05(0.02 to 0.07) | 0.01(-0.05 to 0.07) |
| South Africa | 3,447,534(3,265,526-3,638,133) | 25,324.36(23,987.4-26,724.44) | 3,827,465(3,629,982-4,054,242) | 25,172.12(23,873.33-26,663.56) | -0.02(-0.03 to 0) | -0.11(-0.12 to -0.1) | 0.12(0.11 to 0.13) | -0.06(-0.1 to -0.02) |
| Eswatini | 97,848(92,315-104,272) | 25,366.34(23,931.99-27,031.64) | 104,522(98,091-110,824) | 25,330.4(23,771.85-26,857.53) | 0(-0.01 to 0) | -0.09(-0.09 to -0.08) | 0.03(0.03 to 0.04) | 0.03(0.02 to 0.05) |
| Zimbabwe | 1,216,928(1,148,743-1,295,172) | 25,266.81(23,851.09-26,891.37) | 1,594,757(1,501,069-1,696,101) | 25,338.35(23,849.78-26,948.55) | 0.01(0 to 0.02) | -0.02(-0.03 to -0.01) | 0.05(0.05 to 0.06) | -0.01(-0.04 to 0.01) |
| Benin | 592,797(558,091-632,956) | 24,477.76(23,044.65-26,135.98) | 1,494,922(1,399,446-1,595,887) | 24,585.93(23,015.71-26,246.44) | 0.02(0.01 to 0.03) | -0.11(-0.12 to -0.1) | 0.11(0.1 to 0.12) | 0.03(0.01 to 0.05) |
| Burkina Faso | 1,151,509(1,087,557-1,224,471) | 24,401.24(23,046.06-25,947.36) | 2,567,003(2,416,107-2,727,552) | 24,748.96(23,294.14-26,296.84) | 0.05(0.04 to 0.06) | -0.06(-0.07 to -0.05) | 0.09(0.09 to 0.1) | 0.08(0.07 to 0.1) |
| Cameroon | 1,198,218(1,127,872-1,280,786) | 24,543.39(23,102.46-26,234.64) | 3,279,121(3,078,891-3,503,179) | 24,349.46(22,862.63-26,013.23) | -0.02(-0.04 to 0) | -0.16(-0.18 to -0.14) | 0.12(0.11 to 0.14) | -0.04(-0.08 to 0) |
| Cabo Verde | 38,142(35,989-40,840) | 24,243.7(22,875.23-25,959.02) | 34,246(32,081-36,714) | 23,914.31(22,402.7-25,637.72) | -0.04(-0.07 to -0.02) | -0.27(-0.31 to -0.23) | 0.14(0.09 to 0.19) | -0.03(-0.07 to 0.01) |
| Chad | 720,338(675,685-766,012) | 24,615.47(23,089.59-26,176.23) | 2,227,460(2,104,311-2,370,820) | 24,708.65(23,342.6-26,298.91) | 0.02(0 to 0.03) | -0.09(-0.11 to -0.08) | 0.07(0.05 to 0.08) | 0.05(0.01 to 0.09) |
| Coted'Ivoire | 1,398,638(1,321,434-1,484,039) | 24,523.31(23,169.62-26,020.69) | 2,832,396(2,657,407-3,024,451) | 24,476.74(22,964.54-26,136.43) | 0(-0.02 to 0.01) | -0.15(-0.16 to -0.14) | 0.12(0.11 to 0.14) | -0.01(-0.04 to 0.02) |
| Gambia | 112,851(106,211-120,345) | 24,465.2(23,025.59-26,089.8) | 241,316(226,357-257,202) | 24,290.71(22,784.91-25,889.79) | -0.02(-0.03 to -0.01) | -0.08(-0.09 to -0.07) | 0.09(0.07 to 0.11) | -0.07(-0.09 to -0.04) |
| Ghana | 1,634,791(1,537,635-1,747,054) | 24,339.62(22,893.11-26,011.05) | 3,140,581(2,946,358-3,338,944) | 24,377.56(22,869.97-25,917.28) | 0.01(0 to 0.02) | -0.14(-0.15 to -0.12) | 0.1(0.08 to 0.12) | 0.04(0.01 to 0.06) |
| Guinea | 677,613(641,419-718,986) | 24,623.99(23,308.74-26,127.47) | 1,476,927(1,387,559-1,578,419) | 24,429.17(22,950.98-26,107.89) | -0.02(-0.04 to -0.01) | -0.18(-0.19 to -0.17) | 0.09(0.07 to 0.12) | 0.01(-0.02 to 0.03) |
| Guinea-Bissau | 117,258(110,458-124,742) | 24,307.38(22,897.62-25,858.79) | 219,104(206,015-233,704) | 24,395.14(22,937.74-26,020.72) | 0.02(0.01 to 0.03) | -0.1(-0.11 to -0.09) | 0.16(0.14 to 0.18) | -0.03(-0.05 to -0.01) |
| Liberia | 276,959(261,107-293,978) | 24,505.83(23,103.20-26,011.67) | 529,238(496,339-564,194) | 24,211.08(22,706.06-25,810.24) | -0.03(-0.08 to 0.01) | -0.1(-0.23 to 0.03) | 0.1(0.08 to 0.11) | -0.11(-0.16 to -0.06) |
| Mali | 1,015,710(955,772-1,075,511) | 24,594.6(23,143.24-26,042.62) | 2,859,619(2,692,636-3,041,779) | 24,703.45(23,260.93-26,277.08) | 0.02(0 to 0.03) | -0.07(-0.09 to -0.06) | 0.06(0.06 to 0.07) | 0.04(0.01 to 0.07) |
| Mauritania | 225,637(212,252-239,152) | 24,410.05(22,962.07-25,872.19) | 450,098(421,524-482,414) | 24,290.07(22,748.04-26,034.05) | -0.01(-0.03 to 0.01) | -0.11(-0.12 to -0.1) | 0.1(0.09 to 0.12) | -0.05(-0.09 to 0) |
| Niger | 998,403(945,514-1,059,551) | 24,573.35(23,271.61-26,078.36) | 3,150,490(2,960,499-3,339,770) | 24,682.64(23,194.14-26,165.56) | 0.02(0 to 0.03) | -0.01(-0.04 to 0.02) | 0.02(0.01 to 0.03) | 0.04(0 to 0.07) |
| Nigeria | 9,776,013(9,301,959-10,300,155) | 24,986.9(23,775.25-26,326.58) | 25,179,326(23,952,779-26,552,044) | 24,785.47(23,578.11-26,136.72) | -0.02(-0.03 to -0.01) | -0.04(-0.06 to -0.03) | 0.04(0.04 to 0.05) | -0.08(-0.1 to -0.05) |
| Sao Tome and Principe | 13,652(12,809-14,521) | 24,092.46(22,605.12-25,626.23) | 18,676(17,537-20,055) | 23,995.19(22,531.97-25,766.52) | -0.01(-0.02 to 0) | -0.05(-0.07 to -0.03) | 0.12(0.12 to 0.13) | -0.12(-0.14 to -0.09) |
| Senegal | 892,225(836,954-951,329) | 24,435.93(22,922.19-26,054.63) | 1,549,492(1,451,347-1,659,698) | 24,360.9(22,817.89-26,093.55) | -0.01(-0.02 to 0.01) | -0.12(-0.15 to -0.1) | 0.11(0.09 to 0.13) | -0.03(-0.06 to 0) |
| Sierra Leone | 447,911(423,892-477,808) | 24,710.6(23,385.49-26,359.95) | 876,523(824,262-933,660) | 24,509.86(23,048.51-26,107.55) | -0.03(-0.06 to -0.01) | -0.21(-0.24 to -0.17) | 0.06(-0.01 to 0.13) | 0.03(0.01 to 0.05) |
| Togo | 427,880(402,133-455,962) | 24,276.41(22,815.6-25,869.64) | 802,574(750,171-857,966) | 24,253.96(22,670.33-25,927.93) | 0(-0.01 to 0.01) | -0.08(-0.09 to -0.07) | 0.12(0.11 to 0.13) | -0.06(-0.08 to -0.03) |
| American Samoa | 579(546-618) | 3,042.4(2,871.04-3,249.14) | 429(400-461) | 3,024.23(2,820.61-3,249.6) | -0.02(-0.03 to -0.02) | -0.02(-0.03 to -0.01) | -0.01(-0.01 to 0) | -0.04(-0.05 to -0.03) |
| Bermuda | 564(532-601) | 4,733.22(4,465.73-5,037.75) | 396(371-425) | 4,691.1(4,397.39-5,036.7) | -0.03(-0.04 to -0.02) | 0.01(0 to 0.02) | 0.04(0.03 to 0.04) | -0.12(-0.14 to -0.11) |
| Cook Islands | 196(183-210) | 2,972.07(2,778.77-3,187.35) | 115(108-123) | 3,040.48(2,844.14-3,248.63) | 0.08(0.06 to 0.09) | 0.12(0.09 to 0.16) | 0.02(-0.02 to 0.05) | 0.09(0.07 to 0.1) |
| Greenland | 535(500-565) | 3,759.05(3,516.2-3,974.96) | 438(408-465) | 3,726.17(3,474.41-3,952.77) | -0.03(-0.05 to -0.01) | -0.11(-0.12 to -0.09) | -0.07(-0.09 to -0.05) | 0.07(0.03 to 0.12) |
| Guam | 1,265(1,189-1,349) | 3,032.11(2,851.41-3,234.89) | 1,133(1,064-1,216) | 3,097.44(2,909.76-3,323.41) | 0.07(0.06 to 0.08) | 0.03(0.01 to 0.06) | 0.05(0.04 to 0.06) | 0.12(0.1 to 0.14) |
| Monaco | 209(199-220) | 5,927.18(5,640.87-6,228.01) | 297(282-311) | 5,955.74(5,656.07-6,250.3) | 0.01(-0.03 to 0.05) | -0.09(-0.15 to -0.03) | -0.08(-0.16 to 0) | 0.2(0.14 to 0.26) |
| Nauru | 128(120-137) | 3,020.86(2,831.97-3,251.13) | 123(116-131) | 3,091.92(2,908.48-3,299.44) | 0.08(0.07 to 0.08) | 0.09(0.08 to 0.09) | 0.1(0.1 to 0.11) | 0.04(0.02 to 0.07) |
| Niue | 24(22-26) | 2,956.4(2,754.71-3,167.75) | 12(11-13) | 3,056.86(2,847.59-3,275.44) | 0.11(0.1 to 0.12) | 0.08(0.08 to 0.09) | 0.18(0.17 to 0.19) | 0.07(0.05 to 0.1) |
| Northern Mariana Islands | 371(349-395) | 3,054.15(2,872.74-3,248.72) | 343(320-368) | 3,045.95(2,841.75-3,264.98) | 0(-0.06 to 0.06) | 0.18(0.02 to 0.35) | -0.33(-0.42 to -0.24) | 0.06(0.04 to 0.09) |
| Palau | 135(127-145) | 2,967.56(2,778.9-3,178.76) | 99(92-106) | 3,032.1(2,832.92-3,259.69) | 0.07(0.05 to 0.09) | 0.13(0.07 to 0.18) | 0.11(0.09 to 0.14) | -0.03(-0.06 to 0) |
| Puerto Rico | 46,412(43,528-49,521) | 4,660.88(4,371.26-4,973.15) | 20,368(19,065-21,767) | 4,583.26(4,290.07-4,898.12) | -0.05(-0.06 to -0.05) | 0.11(0.11 to 0.12) | -0.06(-0.07 to -0.06) | -0.19(-0.2 to -0.17) |
| Saint Kitts and Nevis | 667(624-712) | 4,720.38(4,421.44-5,042.51) | 469(439-498) | 4,763.12(4,463.13-5,054.18) | 0.03(0.02 to 0.03) | -0.02(-0.03 to -0.01) | -0.03(-0.04 to -0.01) | 0.13(0.12 to 0.14) |
| San Marino | 233(222-244) | 5,685.96(5,416.17-5,966.67) | 249(237-262) | 5,659.48(5,394.66-5,948.89) | -0.03(-0.05 to -0.01) | 0.48(0.43 to 0.53) | -0.11(-0.14 to -0.07) | -0.38(-0.39 to -0.36) |
| Tokelau | 18(17-19) | 2,962.1(2,773.2-3,160.8) | 12(11-13) | 2,995.71(2,788.90-3,224.47) | 0.04(0.01 to 0.07) | -0.06(-0.12 to 0) | 0.15(0.08 to 0.21) | 0.02(-0.01 to 0.05) |
| Tuvalu | 106(101-113) | 3,057.45(2,891.83-3,260.47) | 115(107-122) | 3,072.34(2,877.61-3,281.86) | 0.02(0.01 to 0.02) | -0.1(-0.11 to -0.1) | 0.02(0.01 to 0.03) | 0.11(0.1 to 0.12) |
| United States Virgin Islands | 1,502(1,413-1,596) | 4,702.29(4,423.73-4,996.67) | 627(584-668) | 4,685.8(4,363.74-4,987.63) | -0.01(-0.02 to -0.01) | 0.06(0.05 to 0.06) | 0(0 to 0.01) | -0.08(-0.09 to -0.08) |
| South Sudan | 722,244(681,878-768,499) | 27,522.92(25,984.68-29,285.6) | 1,180,389(1,113,668-1,256,495) | 27,483.18(25,929.7-29,255.16) | -0.01(-0.02 to 0.01) | -0.04(-0.05 to -0.04) | 0(-0.01 to 0.01) | 0.02(-0.01 to 0.05) |
| Sudan | 764,927(726,782-808,025) | 8,602(8,173.03-9,086.65) | 1,436,016(1,359,861-1,516,717) | 8,656.29(8,197.22-9,142.75) | 0.02(0.01 to 0.03) | 0.05(0.04 to 0.06) | 0.05(0.04 to 0.05) | -0.03(-0.05 to -0.02) |

**sTable4 The incident cases and incidence rate of fungal skin diseases and their AAPCs from 1990 to 2021 at the global, regional and national levels**

| *Rate per 100,000* | 1990 | | 2021 | | 1990-2021 | 1990-1999 | 2000-2009 | 2010-2021 |
| --- | --- | --- | --- | --- | --- | --- | --- | --- |
| *AAPC(95% CI)* | Incident cases | Incidence rate | Incident cases | Incidence rate | AAPC | AAPC | AAPC | AAPC |
| Fungal skin diseases | 290,930,380(239,071,081-352,548,040) | 16,728.3(13,746.42-20,271.27) | 381,786,200(314,958,733-466,426,730) | 18,976.77(15,655.09-23,183.84) | 0.4(0.37 to 0.44) | 0.58(0.57 to 0.59) | 0.58(0.57 to 0.59) | 0.09(-0.01 to 0.19) |
| Male | 159,907,962(130,920,107-193,934,122) | 17,896.97(14,652.6-21,705.19) | 209,448,145(172,195,584-256,622,411) | 20,174.83(16,586.52-24,718.83) | 0.39(0.36 to 0.43) | 0.59(0.55 to 0.64) | 0.55(0.53 to 0.57) | 0.04(-0.04 to 0.12) |
| Female | 131,022,418(108,524,285-158,701,381) | 15,493.52(12,833.09-18,766.58) | 172,338,056(142,387,890-209,386,164) | 17,699.38(14,623.45-21,504.27) | 0.4(0.36 to 0.44) | 0.5(0.49 to 0.52) | 0.5(0.49 to 0.52) | 0.2(0.09 to 0.31) |
| **Age groups** |  |  |  |  |  |  |  |  |
| 0-4 years | 100,776,145(78,636,317-132,312,846) | 16,255.84(12,684.55-21,342.92) | 120,927,681(93,928,342-159,813,892) | 18,373.27(14,271.10-24,281.49) | 0.4 (0.36 to 0.45) | 0.98(0.93 to 1.04) | 0.19(0.14 to 0.23) | 0.1(0.03 to 0.18) |
| 5-9 years | 104,386,958(76,959,419-135,889,840) | 17,888.85(13,188.58-23,287.52) | 140,377,507(10,390,2413-18,256,6892) | 20,431.81(15,122.9-26,572.44) | 0.42 (0.38-0.46) | 0.61(0.57 to 0.66) | 0.71(0.68 to 0.73) | -0.04(-0.14 to 0.06) |
| 10-14 years | 85,767,278(65,217,780-107,916,317) | 16,010.84(12,174.71-20,145.57) | 120,481,012(91,065,535-152,565,032) | 18,072.98(13,660.46-22,885.81) | 0.35 (0.28-0.42) | 0.14(0.08 to 0.2) | 0.72(0.62 to 0.83) | 0.18(0.01 to 0.35) |
| **SDI region** |  |  |  |  |  |  |  |  |
| High SDI | 15,712,256(13,159,272-18,789,147) | 8,456.19(7,082.2-10,112.14) | 13,711,783(11,475,514-16,442,377) | 7,947.18(6,651.06-9,529.8) | -0.2 (-0.21 to -0.19) | -0.36(-0.37 to -0.35) | -0.37(-0.38 to -0.37) | 0.11(0.1 to 0.13) |
| High-middle SDI | 25,366,856(21,150,216-29,988,674) | 9,270.73(7,729.69-10,959.85) | 20,252,588(16,771,878-24,170,845) | 8,771.52(7,264-10,468.54) | -0.18 (-0.21 to -0.16) | 0.09(0.04 to 0.14) | -0.36(-0.42 to -0.3) | -0.24(-0.28 to -0.21) |
| Middle SDI | 78,818,537(64,804,171-93,783,294) | 13,655(11,227.06-16,247.58) | 78,605,130(64,733,901-92,515,847) | 13,866.74(11,419.71-16,320.73) | 0.05 (0.03 to 0.06) | 0.18(0.16 to 0.2) | 0.21(0.19 to 0.22) | -0.24(-0.27 to -0.2) |
| Low-middle SDI | 92,359,413(73,678,337-115,720,771) | 19,563.02(15,606.11-24,511.29) | 113,465,931(91,875,636-139,737,811) | 19,568.45(15,844.96-24,099.32) | 0 (-0.04 to 0.03) | 0.08(0.06 to 0.1) | -0.03(-0.11 to 0.05) | -0.09(-0.15 to -0.02) |
| Low SDI | 78,470,696(63,929,690-98,544,396) | 34,279.63(27,927.45-43,048.76) | 155,521,239(126,494,732-193,606,659) | 33,792.39(27,485.38-42,067.77) | -0.06 (-0.12 to 0) | 0.19(0.09 to 0.29) | -0.26(-0.35 to -0.18) | -0.18(-0.31 to -0.05) |
| **GBD region** |  |  |  |  |  |  |  |  |
| Andean Latin America | 2,977,332(2,440,974-3,559,980) | 20,046.63(16,435.29-23,969.65) | 3,458,961(2,825,915-4,153,996) | 19,115.78(15,617.28-22,956.85) | -0.16(-0.17 to -0.15) | -0.25(-0.26 to -0.23) | -0.1(-0.12 to -0.08) | -0.13(-0.15 to -0.12) |
| Australasia | 545,581(448,611-669,762) | 11,896.77(9,782.27-14,604.62) | 668,994(542,931-826,807) | 11,672.95(9,473.34-14,426.55) | -0.06(-0.07 to -0.05) | -0.1(-0.11 to -0.08) | -0.18(-0.19 to -0.16) | 0.06(0.04 to 0.09) |
| Caribbean | 1,742,451(1,439,856-2,091,226) | 15,268.02(12,616.57-18,324.12) | 1,744,301(1,446,949-2,103,626) | 15,161.11(12,576.59-18,284.29) | -0.02(-0.03 to -0.02) | -0.09(-0.1 to -0.08) | 0(0 to 0) | 0.01(0.01 to 0.02) |
| Central Asia | 2,051,551(1,676,149-2,527,964) | 82,09.09(6,706.95-10,115.41) | 2,273,940(1,852,744-2,808,893) | 8,216.35(6,694.46-10,149.28) | 0(-0.01 to 0.01) | 0.28(0.26 to 0.3) | -0.21(-0.24 to -0.19) | -0.04(-0.06 to -0.03) |
| Central Europe | 2,498,786(2,008,742-3,068,244) | 84,75.17(6,813.08-10,406.61) | 1,491,457(1,199,111-1,832,534) | 8,425.73(6,774.18-1,352.59) | -0.02(-0.03 to -0.01) | 0.1(0.09 to 0.11) | -0.22(-0.24 to -0.2) | 0.07(0.06 to 0.08) |
| Central Latin America | 7,446,156(6,164,398-8,966,301) | 11,565.73(9,574.84-13,926.89) | 7,310,526(6,032,433-8,810,312) | 11,515.37(9,502.15-13,877.79) | -0.01(-0.04 to 0.01) | -0.37(-0.4 to -0.35) | -0.12(-0.13 to -0.11) | 0.39(0.32 to 0.45) |
| Central Sub-Saharan Africa | 9,591,878(7,368,179-12,818,633) | 37,914.62(29,124.82-50,669.29) | 20,265,949(15,272,253-26,873,404) | 34,535.43(26,025.61-45,795.26) | -0.29(-0.48 to -0.09) | 0.22(0.11 to 0.33) | -0.07(-0.46 to 0.31) | -1.04(-1.47 to -0.6) |
| East Asia | 29,379,353(24,148,751-35,190,626) | 89,07.32(7,321.49-10,669.2) | 20,982,431(17,139,055-24,854,998) | 7,848.24(6,410.67-9,296.73) | -0.41(-0.44 to -0.38) | -0.07(-0.09 to -0.05) | -0.93(-0.99 to -0.86) | -0.23(-0.28 to -0.19) |
| Eastern Europe | 4,427,802(3,543,096-5,476,238) | 86,04.03(6,884.88-10,641.33) | 3,082,018(2,437,120-3,829,940) | 8,695.45(6,875.97-10,805.6) | 0.03(0.02 to 0.05) | 0.38(0.37 to 0.4) | -0.5(-0.52 to -0.49) | 0.22(0.2 to 0.24) |
| Eastern Sub-Saharan Africa | 40,991,522(33,591,932-51,092,810) | 45,259.13(37,089.17-56,412.05) | 76,399,283(62,322,062-94,967,536) | 42,817.15(34,927.72-53,223.53) | -0.2(-0.27 to -0.14) | 0.08(0 to 0.17) | -0.34(-0.38 to -0.29) | -0.3(-0.46 to -0.15) |
| High-income Asia Pacific | 3,522,929(2,877,407-4,296,608) | 10,008.45(8,174.56-12,206.43) | 2,144,764(1,741,132-2,644,243) | 9,563.93(7,764.06-11,791.21) | -0.14(-0.15 to -0.13) | -0.37(-0.38 to -0.35) | -0.11(-0.12 to -0.1) | 0.02(0 to 0.04) |
| High-income North America | 2,529,522(2,213,746-2,844,825) | 4,101.2(3,589.22-4,612.41) | 2,683,972(2,359,119-3,027,359) | 4,090.24(3,595.18-4,613.54) | 0(-0.01 to 0.01) | 0.02(0 to 0.05) | -0.17(-0.2 to -0.14) | 0.11(0.1 to 0.12) |
| North Africa and Middle East | 7,284,612(6,100,042-8,501,185) | 5,185.32(4,342.12-6,051.29) | 7,855,547(6,562,992-9,254,080) | 4,285.11(3,580.03-5,047.99) | -0.63(-0.66 to -0.59) | -0.93(-1.01 to -0.85) | -0.38(-0.41 to -0.35) | -0.61(-0.66 to -0.56) |
| Oceania | 309,185(255,268-368,952) | 11,537.34(9,525.39-13,767.57) | 581,509(479,686-693,895) | 11,445.08(9,441.02-13,657.02) | -0.03(-0.04 to -0.02) | -0.03(-0.05 to -0.02) | 0.03(0 to 0.06) | -0.07(-0.08 to -0.07) |
| South Asia | 88,134,224(68,812,228-114,359,029) | 20,337.33(15,878.70-26,388.81) | 93,311,287(73,660,276-117,752,645) | 18,403.81(14,528.04-23,224.39) | -0.33(-0.35 to -0.31) | -0.2(-0.22 to -0.17) | -0.4(-0.42 to -0.38) | -0.38(-0.41 to -0.35) |
| Southeast Asia | 28,978,701(24,210,857-34,463,416) | 16,971.67(14,179.33-20,183.84) | 28,855,913(23,960,782-34,317,817) | 16,713.19(13,877.95-19,876.69) | -0.05(-0.06 to -0.05) | -0.04(-0.04 to -0.03) | -0.06(-0.08 to -0.04) | -0.06(-0.08 to -0.04) |
| Southern Latin America | 1,483,568(1,217,533-1,799,440) | 9,939.2(8,156.89-12,055.39) | 1,392,345(1,134,219-1,724,121) | 9,605.34(7,824.61-11,894.15) | -0.11(-0.12 to -0.09) | -0.24(-0.26 to -0.23) | -0.17(-0.19 to -0.16) | 0.07(0.03 to 0.1) |
| Southern Sub-Saharan Africa | 4,566,798(3,706,695-5,552,965) | 22,073.21(17,915.98-26,839.76) | 5,274,654(4,283,639-6,430,205) | 21,917.59(17,799.66-26,719.21) | -0.05(-0.11 to 0.01) | 0.31(0.24 to 0.38) | 0(-0.06 to 0.05) | -0.39(-0.54 to -0.24) |
| Tropical Latin America | 8,638,474(7,047,577-10,486,375) | 16,112.35(13,145.04-19,559.03) | 7,734,694(6,359,358-9,361,778) | 15,409.89(12,669.8-18,651.54) | -0.15(-0.15 to -0.14) | -0.23(-0.24 to -0.22) | -0.07(-0.08 to -0.06) | -0.13(-0.14 to -0.12) |
| Western Europe | 9,078,137(7,531,167-10,999,391) | 12,782.82(10,604.55-15,488.11) | 8,491,173(6,967,924-10,357,723) | 12,465.33(10,229.15-15,205.48) | -0.08(-0.09 to -0.07) | -0.1(-0.12 to -0.09) | -0.25(-0.26 to -0.25) | 0.08(0.07 to 0.1) |
| Western Sub-Saharan Africa | 34,751,818(28,240,364-43,465,421) | 39,544.71(32,135.21-49,460.07) | 85,782,482(69,859,257-105,911,711) | 39,942.72(32,528.42-49,315.45) | 0.02(-0.03 to 0.07) | 0.43(0.35 to 0.51) | -0.29(-0.37 to -0.21) | -0.1(-0.2 to 0) |
| **204 countries and territories** |  |  |  |  |  |  |  |  |
| China | 28,485,205(23,394,631-34,147,485) | 8,947.02(7,348.1-10,725.51) | 20,402,323(16,650,948-24,186,192) | 7,858.18(6,413.3-9,315.58) | -0.42(-0.45 to -0.39) | -0.07(-0.09 to -0.04) | -0.95(-1.02 to -0.88) | -0.19(-0.24 to -0.14) |
| Democratic People's Republic of Korea | 490,705(401,752-590,581) | 8,248.16(6,752.98-9,926.97) | 378,422(309,680-452,566) | 7,927.21(6,487.19-9,480.37) | -0.13(-0.16 to -0.11) | 0.02(-0.02 to 0.06) | -0.28(-0.31 to -0.26) | -0.14(-0.18 to -0.1) |
| Taiwan (Province of China) | 403,443(330,254-482,207) | 7,324.51(5,995.77-8,754.47) | 201,686(164,709-239,854) | 6,844.37(5,589.52-8,139.65) | -0.22(-0.24 to -0.2) | -0.4(-0.45 to -0.36) | -0.06(-0.08 to -0.04) | -0.22(-0.26 to -0.18) |
| Cambodia | 751,907(622,521-900,408) | 16,132.09(13,356.13-19,318.15) | 809,828(665,055-968,554) | 15,827.6(12,998.1-18,929.78) | -0.06(-0.09 to -0.04) | 0.24(0.22 to 0.26) | -0.25(-0.3 to -0.21) | -0.11(-0.15 to -0.06) |
| Indonesia | 12,109,816(9,925,601-14,469,052) | 17,877.63(14,653.09-21,360.55) | 11,775,779(9,652,536-14,091,778) | 17,500.47(14,345.03-20,942.37) | -0.07(-0.08 to -0.06) | -0.06(-0.09 to -0.04) | -0.09(-0.1 to -0.09) | -0.07(-0.1 to -0.04) |
| Lao People's Democratic Republic | 304,160(250,452-363,943) | 16,502.55(13,588.55-19,746.15) | 367,836(302,081-439,895) | 16,018.18(13,154.75-19,156.14) | -0.1(-0.1 to -0.09) | 0(0 to 0.01) | -0.04(-0.05 to -0.04) | -0.24(-0.25 to -0.23) |
| Malaysia | 1,043,587(859,494-1,252,661) | 15,876.86(13,076.12-19,057.66) | 1,186,701(976,078-1,427,772) | 15,586.51(12,820.12-18,752.8) | -0.06(-0.08 to -0.05) | -0.04(-0.05 to -0.03) | -0.02(-0.05 to 0) | -0.11(-0.14 to -0.08) |
| Maldives | 16,290(13,434-19,608) | 15,507.99(12,789.06-18,667.2) | 15,217(12,489-18,324) | 15,190.89(12,467.57-18,293.34) | -0.07(-0.09 to -0.04) | 0.26(0.24 to 0.28) | -0.43(-0.49 to -0.38) | 0.11(0.06 to 0.15) |
| Myanmar | 2,440,342(2,002,395-2,929,461) | 16,515.2(13,551.36-19,825.36) | 2,500,411(2,064,074-2,996,440) | 16,013.12(13,218.74-19,189.79) | -0.11(-0.12 to -0.1) | 0.01(0 to 0.02) | -0.15(-0.16 to -0.15) | -0.15(-0.18 to -0.12) |
| Philippines | 4,292,109(3,540,690-5,134,345) | 17,022.49(14,042.36-20,362.79) | 5,836,191(4,819,295-6,991,153) | 17,166.02(14,175.02-20,563.11) | 0.02(0.01 to 0.03) | 0(-0.02 to 0.01) | 0.06(0.05 to 0.08) | 0.02(-0.01 to 0.04) |
| Sri Lanka | 1,024,744(843,398-1,198,504) | 18,520.44(15,242.93-21,660.83) | 918,042(756,641-1,072,230) | 17,986.73(14,824.49-21,007.67) | -0.1(-0.11 to -0.08) | -0.14(-0.16 to -0.12) | -0.3(-0.31 to -0.28) | 0.16(0.14 to 0.18) |
| Thailand | 2,687,204(2,198,775-3,211,441) | 15,939.69(13,042.48-19,049.31) | 1,511,769(1,235,346-1,815,791) | 15,479.45(12,649.07-18,592.42) | -0.1(-0.11 to -0.08) | -0.18(-0.19 to -0.17) | -0.05(-0.07 to -0.03) | -0.03(-0.04 to -0.02) |
| Timor-Leste | 52,484(43,323-62,891) | 15,778.51(13,024.29-18,907.06) | 83,479(68,735-99,786) | 16,033.91(13,202.03-19,166.11) | 0.05(0.03 to 0.07) | 0.11(0.09 to 0.14) | 0.01(-0.05 to 0.07) | -0.03(-0.06 to -0.01) |
| Viet Nam | 4,157,899(3,425,747-5,001,091) | 15,682.81(12,921.27-18,863.17) | 3,774,156(3,089,990-4,542,093) | 15,241.33(12,478.44-18,342.53) | -0.09(-0.11 to -0.07) | 0.14(0.14 to 0.15) | -0.3(-0.35 to -0.26) | 0.02(-0.02 to 0.07) |
| Fiji | 33,041(27,007-39,480) | 11,740.16(9,596.08-14,028.08) | 31,779(25,942-37,756) | 11,660.98(9,519.02-13,854.27) | -0.03(-0.04 to -0.02) | 0.05(0.02 to 0.08) | -0.09(-0.11 to -0.07) | -0.01(-0.03 to 0.01) |
| Kiribati | 3,641(2,965-4,391) | 12,327.82(10,038.69-14,867.02) | 5,142(4,199-6,165) | 12,237.26(9,994.15-14,672.74) | -0.03(-0.06 to -0.01) | 0.02(-0.02 to 0.05) | -0.1(-0.14 to -0.06) | -0.01(-0.06 to 0.03) |
| Marshall Islands | 2,634(2,140-3,180) | 12,005.39(9,754.81-14,493.74) | 2,069(1,684-2,486) | 11,853.21(9,647.16-14,238.45) | -0.05(-0.07 to -0.03) | 0.1(0.05 to 0.14) | -0.18(-0.21 to -0.16) | -0.06(-0.11 to -0.02) |
| Micronesia (Federated States of) | 5,584(4,536-6,737) | 12,158.01(9,876.74-14,669.04) | 3,597(2,952-4,281) | 11,756.08(9,646.08-13,988.23) | -0.11(-0.12 to -0.1) | -0.03(-0.05 to -0.02) | -0.18(-0.2 to -0.17) | -0.11(-0.14 to -0.08) |
| Papua New Guinea | 195,047(160,979-232,807) | 11,472.92(9,469.00-13,693.99) | 447,387(369,216-534,885) | 11,421.55(9,425.88-13,655.32) | -0.02(-0.03 to -0.01) | -0.01(-0.02 to 0.01) | 0.05(0.02 to 0.08) | -0.08(-0.09 to -0.08) |
| Samoa | 8,313(6,813-9,867) | 11,666.02(9,561.91-13,847.01) | 8,961(7,374-10,720) | 11,208.67(9,224.3-13,409.75) | -0.13(-0.15 to -0.12) | -0.25(-0.27 to -0.23) | -0.08(-0.1 to -0.06) | -0.12(-0.16 to -0.09) |
| Solomon Islands | 18,217(15,076-21,751) | 11,699.44(9,682.66-13,969.15) | 29,914(24,772-35,400) | 11,503.31(9,525.93-13,613.14) | -0.06(-0.07 to -0.04) | -0.12(-0.15 to -0.1) | 0.02(-0.01 to 0.04) | -0.06(-0.08 to -0.04) |
| Tonga | 4,744(3,933-5,644) | 11,348.76(9,408.90-13,501.25) | 4,329(3,560-5,164) | 11,094.83(9,123.73-13,232.47) | -0.08(-0.09 to -0.06) | -0.06(-0.08 to -0.04) | -0.08(-0.09 to -0.07) | -0.1(-0.12 to -0.08) |
| Vanuatu | 7,877(6,484-9,428) | 11,568.36(9,522.26-13,846) | 13,525(11,186-16,194) | 11,604.9(9,597.99-13,895.51) | 0.01(0 to 0.02) | 0.02(0 to 0.04) | 0.02(0.01 to 0.03) | -0.01(-0.03 to 0) |
| Armenia | 85,802(69,958-105,973) | 8,224.88(6,706.1-10,158.4) | 49,415(39,812-61,313) | 8,341.88(6,720.91-10,350.54) | 0.05(0.02 to 0.07) | 0.4(0.39 to 0.42) | -0.33(-0.39 to -0.27) | 0.18(0.13 to 0.22) |
| Azerbaijan | 200,048(163,345-246,884) | 8,242.82(6,730.48-10,172.65) | 197,806(159,336-245,970) | 8,379.53(6,749.84-10,419.89) | 0.05(0.04 to 0.07) | 0.32(0.3 to 0.34) | -0.2(-0.22 to -0.18) | 0.18(0.15 to 0.22) |
| Georgia | 113,794(92,436-140,564) | 8,313.96(6,753.51-10,269.84) | 61,058(49,367-75,836) | 8,296.64(6,708.13-10,304.73) | 0(-0.03 to 0.02) | 0.28(0.26 to 0.3) | -0.25(-0.27 to -0.23) | 0.08(0.01 to 0.14) |
| Kazakhstan | 429,455(350,091-528,899) | 8,264.73(6,737.41-10,178.51) | 445,485(362,931-550,191) | 8,209.47(6,688.15-10,139) | -0.02(-0.04 to 0) | 0.34(0.31 to 0.37) | -0.47(-0.51 to -0.42) | 0.14(0.12 to 0.16) |
| Kyrgyzstan | 137,648(112,443-169,599) | 8,205.14(6,702.68-10,109.74) | 187,298(152,196-232,080) | 8,235.09(6,691.71-10,204.05) | 0.01(0 to 0.03) | 0.25(0.23 to 0.27) | -0.23(-0.25 to -0.2) | 0.14(0.11 to 0.17) |
| Mongolia | 74,301(60,735-91,374) | 8,255.66(6,748.3-10,152.54) | 89,142(72,359-110,725) | 8,203.69(6,659.23-10,189.99) | -0.02(-0.03 to -0.01) | 0.3(0.28 to 0.33) | -0.35(-0.38 to -0.33) | 0.12(0.09 to 0.15) |
| Tajikistan | 188,971(154,963-232,796) | 8,138.13(6,673.54-10,025.48) | 293,598(239,580-362,498) | 8,191.07(6,684.02-10,113.32) | 0.02(0.01 to 0.03) | 0.23(0.21 to 0.25) | -0.09(-0.11 to -0.08) | -0.01(-0.04 to 0.02) |
| Turkmenistan | 122,963(100,550-151,387) | 8,192.84(6,699.48-10,086.68) | 125,677(102,280-155,215) | 8,246.5(6,711.29-10,184.71) | 0.02(0.01 to 0.03) | 0.16(0.15 to 0.17) | -0.03(-0.04 to -0.02) | -0.03(-0.05 to -0.01) |
| Uzbekistan | 698,569(571,499-861,125) | 8,165.01(6,679.8-10,065) | 824,462(673,313-1,017,601) | 8,170.22(6,672.37-10,084.18) | 0(-0.01 to 0.01) | 0.28(0.27 to 0.3) | -0.11(-0.13 to -0.09) | -0.09(-0.12 to -0.06) |
| Albania | 91,906(74,903-113,507) | 8,226.1(6,704.25-10,159.5) | 36,828(29,720-45,539) | 8,300.56(6,698.45-10,263.83) | 0.03(0.02 to 0.04) | 0.17(0.16 to 0.18) | 0.13(0.1 to 0.15) | -0.16(-0.17 to -0.15) |
| Bosnia and Herzegovina | 91,248(73,719-112,860) | 8,328.62(6,728.64-10,301.25) | 40,901(32,951-50,696) | 8,339.54(6,718.48-10,336.68) | 0(-0.01 to 0.02) | 0.15(0.11 to 0.19) | -0.03(-0.05 to -0.01) | -0.04(-0.05 to -0.02) |
| Bulgaria | 145,601(117,600-180,087) | 8,386.08(6,773.32-10,372.36) | 81,420(65,731-100,836) | 8,341.66(6,734.3-10,330.83) | -0.02(-0.03 to -0.01) | 0.16(0.14 to 0.18) | -0.41(-0.42 to -0.39) | 0.16(0.15 to 0.17) |
| Croatia | 82,539(66,510-102,303) | 8,362.44(6,738.46-10,364.85) | 49,822(40,198-61,724) | 8,343.46(6,731.73-10,336.58) | -0.01(-0.01 to 0) | -0.03(-0.04 to -0.01) | -0.02(-0.04 to -0.01) | 0.07(0.06 to 0.08) |
| Czechia | 185,799(149,661-228,728) | 8,431.26(6,791.4-10,379.32) | 142,188(114,946-175,565) | 8,284.33(6,697.16-10,229.03) | -0.06(-0.07 to -0.04) | 0.03(0.01 to 0.05) | -0.45(-0.48 to -0.42) | 0.19(0.18 to 0.2) |
| Hungary | 180,169(145,052-221,657) | 8,454.37(6,806.53-10,401.19) | 115,011(93,012-142,008) | 8,283.02(6,698.71-10,227.35) | -0.07(-0.07 to -0.06) | -0.09(-0.11 to -0.07) | -0.14(-0.15 to -0.13) | -0.01(-0.01 to -0.01) |
| North Macedonia | 43,867(35,472-54,243) | 8,327.47(6,733.87-10,297.21) | 27,306(21,984-33,866) | 8,335.56(6,710.92-10,338.04) | 0(0 to 0.01) | 0.08(0.07 to 0.09) | -0.01(-0.02 to 0) | -0.01(-0.02 to 0.01) |
| Montenegro | 13,422(10,849-16,613) | 8,305.62(6,713.16-10,280.07) | 9,236(7,460-11,412) | 8,289.5(6,696.06-10,243.19) | -0.01(-0.01 to 0) | 0.03(0.03 to 0.04) | -0.01(-0.02 to 0) | -0.03(-0.04 to -0.02) |
| Poland | 830,434(658,512-1,031,045) | 8,671.3(6,876.11-10,766.06) | 506,851(402,144-627,197) | 8,611.54(6,832.54-10,656.26) | -0.02(-0.03 to -0.01) | 0.14(0.13 to 0.14) | -0.25(-0.26 to -0.25) | 0.11(0.09 to 0.13) |
| Romania | 467,208(378,122-577,024) | 8,390.87(6,790.92-10,363.13) | 251,048(202,738-310,861) | 8,340.45(6,735.47-10,327.6) | -0.02(-0.03 to -0.01) | 0.15(0.13 to 0.16) | -0.3(-0.35 to -0.26) | 0.06(0.05 to 0.06) |
| Serbia | 180,992(146,167-223,814) | 8,345.1(6,739.43-10,319.56) | 112,389(90,279-138,594) | 8,464.15(6,799-10,437.67) | 0.05(0.04 to 0.05) | 0.04(0.03 to 0.06) | -0.06(-0.07 to -0.06) | 0.16(0.14 to 0.17) |
| Slovakia | 110,934(89,561-137,343) | 8,368.1(6,755.9-10,360.18) | 70,820(57,379-87,387) | 8,268.01(6,698.81-10,202.19) | -0.04(-0.05 to -0.02) | 0.13(0.12 to 0.14) | -0.23(-0.27 to -0.2) | 0.05(0.02 to 0.08) |
| Slovenia | 34,708(27,973-43,074) | 8,393.22(6,764.46-10,416.07) | 25,928(20,884-32,113) | 8,303.18(6,687.83-10,283.95) | -0.04(-0.05 to -0.02) | 0.05(0.04 to 0.07) | -0.28(-0.31 to -0.25) | 0.18(0.17 to 0.2) |
| Belarus | 199,741(161,909-247,462) | 8,309.16(6,735.35-10,294.31) | 131,940(106,032-164,755) | 8,360.31(6,718.66-10,439.59) | 0.02(0 to 0.04) | 0.38(0.36 to 0.39) | -0.42(-0.45 to -0.4) | 0.23(0.17 to 0.28) |
| Estonia | 28,928(23,520-35,732) | 8,286.31(6,737.19-10,235.25) | 18,008(14,545-22,245) | 8,332.06(6,729.79-10,292.16) | 0.02(-0.01 to 0.05) | 0.38(0.34 to 0.42) | -0.51(-0.57 to -0.45) | 0.21(0.2 to 0.22) |
| Latvia | 46,962(38,232-58,044) | 8,254.05(6,719.65-10,201.82) | 24,757(19,961-30,720) | 8,334.13(6,719.55-10,341.47) | 0.03(0.01 to 0.05) | 0.47(0.43 to 0.52) | -0.48(-0.51 to -0.45) | 0.13(0.1 to 0.16) |
| Lithuania | 68,727(55,908-84,860) | 8,273.37(6,730.23-10,215.51) | 33,903(27,379-42,048) | 8,313.94(6,713.94-10,311.27) | 0.01(0 to 0.03) | 0.3(0.27 to 0.34) | -0.17(-0.19 to -0.15) | 0(-0.02 to 0.02) |
| Republic of Moldova | 102,353(83,228-126,475) | 8,281.49(6,734.1-10,233.26) | 43,853(35,302-54,510) | 8,395.95(6,758.76-10,436.36) | 0.05(0.02 to 0.07) | 0.39(0.37 to 0.41) | -0.28(-0.33 to -0.23) | 0.13(0.11 to 0.16) |
| Russian Federation | 2,997,675(2,395,960-3,708,364) | 8,639.2(6,905.07-10,687.38) | 2,268,485(1,792,409-2,824,476) | 8,699.06(6,873.43-10,831.14) | 0.02(0 to 0.04) | 0.42(0.39 to 0.45) | -0.58(-0.6 to -0.57) | 0.32(0.28 to 0.35) |
| Ukraine | 983,417(784,929-1,214,683) | 8,645.5(6,900.53-10,678.63) | 561,070(441,281-697,178) | 8,842.44(6,954.57-10,987.49) | 0.07(0.05 to 0.1) | 0.35(0.31 to 0.38) | -0.47(-0.51 to -0.42) | 0.46(0.44 to 0.48) |
| Brunei Darussalam | 9,028(7,421-10,968) | 9,966.44(8,193.33-12,108.48) | 9,105(7,451-11,210) | 9,625.21(7,876.68-11,850.02) | -0.11(-0.12 to -0.1) | -0.24(-0.27 to -0.22) | -0.03(-0.04 to -0.03) | -0.09(-0.1 to -0.08) |
| Japan | 2,312,649(1,877,172-2,823,225) | 10,016.23(8,130.15-12,227.57) | 1,489,904(1,206,670-1,834,994) | 9,646.91(7,813.01-11,881.32) | -0.12(-0.13 to -0.11) | -0.3(-0.31 to -0.28) | -0.12(-0.13 to -0.11) | 0.05(0.04 to 0.06) |
| Republic of Korea | 1,138,447(932,085-1,395,456) | 10,012.23(8,197.35-12,272.53) | 571,695(464,050-712,946) | 9,411.41(7,639.33-11,736.72) | -0.2(-0.21 to -0.18) | -0.51(-0.55 to -0.47) | -0.13(-0.14 to -0.12) | 0.08(0.06 to 0.09) |
| Singapore | 62,804(51,677-77,273) | 9,671.51(7,957.96-11,899.69) | 74,060(60,222-91,432) | 9,119.64(7,415.67-11,258.8) | -0.19(-0.21 to -0.17) | -0.29(-0.31 to -0.27) | -0.12(-0.17 to -0.07) | -0.11(-0.16 to -0.06) |
| Australia | 447,180(364,247-548,624) | 11,811.95(9,621.31-14,491.52) | 550,496(442,830-681,329) | 11,590.79(9,323.86-14,345.49) | -0.06(-0.07 to -0.05) | -0.1(-0.11 to -0.09) | -0.17(-0.18 to -0.16) | 0.11(0.08 to 0.13) |
| New Zealand | 98,401(80,888-119,429) | 12,298.08(10,109.32-14,926.18) | 118,498(96,356-145,264) | 12,070.42(9,815.01-14,796.89) | -0.06(-0.08 to -0.04) | -0.08(-0.13 to -0.04) | -0.18(-0.2 to -0.17) | 0.07(0.05 to 0.09) |
| Andorra | 1,248(1,010-1,537) | 13,131.25(10,629.77-16,175.96) | 1,308(1,037-1,619) | 12,862.75(10,200.49-15,928.4) | -0.06(-0.1 to -0.03) | -0.41(-0.46 to -0.35) | 0(-0.01 to 0.01) | 0.18(0.07 to 0.3) |
| Austria | 174,704(142,192-215,884) | 12,956.82(10,545.57-16,010.97) | 162,006(131,238-199,997) | 12,490.57(10,118.43-15,419.73) | -0.12(-0.13 to -0.11) | -0.15(-0.16 to -0.13) | -0.15(-0.15 to -0.14) | -0.05(-0.07 to -0.03) |
| Belgium | 233,614(190,090-287,621) | 12,934.55(10,524.72-15,924.75) | 240,957(194,257-298,106) | 12,601.66(10,159.33-15,590.42) | -0.08(-0.09 to -0.07) | -0.15(-0.17 to -0.13) | -0.2(-0.21 to -0.19) | 0.12(0.11 to 0.13) |
| Cyprus | 25,933(21,103-31,803) | 13,101.83(10,661.74-16,067.42) | 27,236(22,070-33,545) | 12,453.79(10,091.27-15,338.41) | -0.16(-0.17 to -0.16) | -0.05(-0.06 to -0.04) | -0.4(-0.42 to -0.38) | -0.02(-0.03 to -0.01) |
| Denmark | 115,403(94,399-142,060) | 13,064.29(10,686.51-16,082) | 120,093(97,419-147,541) | 12,586.18(10,209.89-15,462.86) | -0.12(-0.13 to -0.11) | -0.27(-0.31 to -0.23) | -0.09(-0.1 to -0.07) | -0.04(-0.07 to -0.02) |
| Finland | 125,935(102,295-154,340) | 13,051.08(10,601.14-15,994.75) | 107,396(86,337-132,510) | 12,678.17(10,192.21-15,642.99) | -0.09(-0.1 to -0.08) | -0.19(-0.2 to -0.18) | -0.25(-0.25 to -0.24) | 0.18(0.16 to 0.2) |
| France | 1,523,068(1,237,458-1,868,009) | 13,001.9(10,563.75-15,946.54) | 1,462,859(1,175,317-1,812,052) | 12,602.56(10,125.38-15,610.87) | -0.1(-0.11 to -0.09) | -0.18(-0.19 to -0.18) | -0.21(-0.23 to -0.2) | 0.09(0.07 to 0.11) |
| Germany | 1,688,967(1,374,766-2,067,308) | 13,046.4(10,619.36-15,968.89) | 1,497,535(1,215,598-1,849,552) | 12,516.36(10,159.93-15,458.5) | -0.13(-0.15 to -0.11) | -0.16(-0.21 to -0.12) | -0.14(-0.16 to -0.13) | -0.09(-0.12 to -0.06) |
| Greece | 264,008(213,318-325,849) | 13,046.61(10,541.66-16,102.65) | 176,463(141,577-217,522) | 12,651.91(10,150.69-15,595.77) | -0.1(-0.11 to -0.09) | -0.22(-0.22 to -0.22) | -0.27(-0.29 to -0.24) | 0.14(0.12 to 0.15) |
| Iceland | 8,103(6,615-9,937) | 12,773.12(10,427.64-15,665.2) | 8,406(6,783-10,364) | 12,446.32(10,042.93-15,345.92) | -0.08(-0.1 to -0.07) | -0.17(-0.19 to -0.14) | -0.16(-0.17 to -0.15) | 0.09(0.06 to 0.11) |
| Ireland | 129,055(104,965-158,781) | 13,135.26(10,683.33-16,160.78) | 125,746(100,879-155,634) | 12,609.98(10,116.25-15,607.17) | -0.13(-0.14 to -0.12) | -0.26(-0.27 to -0.25) | -0.36(-0.38 to -0.35) | 0.22(0.21 to 0.23) |
| Israel | 244,961(198,987-298,942) | 15,978.59(12,979.71-19,499.69) | 403,402(325,227-495,334) | 15,351.68(12,376.66-18,850.17) | -0.13(-0.13 to -0.12) | -0.24(-0.25 to -0.23) | -0.18(-0.18 to -0.17) | 0.04(0.03 to 0.04) |
| Italy | 1,257,162(1,026,310-1,539,562) | 13,620.73(11,119.56-16,680.39) | 1,003,682(810,194-1,239,110) | 13,206.53(10,660.6-16,304.32) | -0.1(-0.11 to -0.08) | -0.32(-0.35 to -0.29) | -0.2(-0.21 to -0.2) | 0.19(0.18 to 0.2) |
| Luxembourg | 8,577(6,995-10,520) | 12,981.38(10,586.32-15,922.27) | 12,684(10,263-15,629) | 12,530.08(10,138.72-15,439.6) | -0.11(-0.12 to -0.1) | -0.25(-0.27 to -0.23) | -0.08(-0.08 to -0.07) | -0.01(-0.03 to 0.02) |
| Malta | 11,378(9,249-14,037) | 12,999.76(10,567.33-16,038) | 7,965(6,448-9,827) | 12,442.15(10,072.96-15,351.62) | -0.14(-0.16 to -0.12) | -0.08(-0.1 to -0.07) | -0.2(-0.24 to -0.15) | -0.08(-0.13 to -0.03) |
| Netherlands | 349,512(285,286-427,528) | 12,824.88(10,468.19-15,687.57) | 335,441(270,445-413,969) | 12,507.02(10,083.64-15,434.99) | -0.08(-0.09 to -0.07) | -0.12(-0.14 to -0.1) | -0.1(-0.12 to -0.09) | -0.03(-0.05 to -0.02) |
| Norway | 107,679(88,014-131,134) | 13,487.67(11,024.47-16,425.68) | 121,766(98,897-150,197) | 13,181.07(10,705.52-16,258.7) | -0.07(-0.09 to -0.05) | -0.2(-0.25 to -0.15) | -0.12(-0.14 to -0.1) | 0.12(0.09 to 0.15) |
| Portugal | 281,648(227,138-344,904) | 13,311.05(10,734.85-16,300.62) | 171,497(137,724-211,089) | 12,589.17(10,109.97-15,495.58) | -0.18(-0.19 to -0.17) | -0.41(-0.43 to -0.38) | -0.15(-0.17 to -0.13) | -0.05(-0.06 to -0.03) |
| Spain | 704,596(611,467-817,214) | 8,991.75(7,803.28-10,428.93) | 543,452(464,660-636,342) | 8,385.6(7,169.81-9,818.91) | -0.23(-0.23 to -0.22) | -0.43(-0.43 to -0.42) | -0.32(-0.33 to -0.32) | -0.02(-0.04 to -0.01) |
| Sweden | 204,986(168,366-250,423) | 13,272.51(10,901.40-16,214.43) | 238,207(193,981-293,776) | 13,083.02(10,653.98-16,135.04) | -0.04(-0.06 to -0.03) | 0.14(0.11 to 0.17) | -0.38(-0.41 to -0.35) | 0.13(0.12 to 0.14) |
| Switzerland | 148,570(121,228-182,492) | 12,856(10,490.1-15,791.39) | 166,246(134,624-205,011) | 12,476.26(10,103.16-15,385.44) | -0.09(-0.11 to -0.08) | -0.1(-0.14 to -0.05) | -0.16(-0.17 to -0.15) | 0.01(-0.02 to 0.03) |
| United Kingdom | 1,460,582(1,194,045-1,782,416) | 13,374.71(10,934-16,321.79) | 1,548,166(1,264,185-1,900,625) | 13,138.93(10,728.86-16,130.17) | -0.05(-0.07 to -0.04) | -0.04(-0.06 to -0.02) | -0.28(-0.29 to -0.27) | 0.19(0.15 to 0.22) |
| Argentina | 1,012,100(830,425-1,229,493) | 9,985.95(8,193.44-12,130.88) | 983,682(801,012-1,218,748) | 9,659.69(7,865.88-11,968.03) | -0.11(-0.12 to -0.09) | -0.28(-0.31 to -0.25) | -0.15(-0.16 to -0.14) | 0.13(0.09 to 0.17) |
| Chile | 390,802(321,142-475,227) | 9,839.13(8,085.34-11,964.69) | 345,715(281,167-427,912) | 9,466.85(7,699.31-11,717.68) | -0.12(-0.13 to -0.11) | -0.16(-0.18 to -0.14) | -0.25(-0.26 to -0.24) | 0.09(0.07 to 0.11) |
| Uruguay | 80,596(66,159-98,556) | 9,845.9(8,082.18-12,039.97) | 62,872(51,241-77,778) | 9,532.99(7,769.38-11,793.09) | -0.1(-0.11 to -0.09) | -0.26(-0.3 to -0.23) | -0.06(-0.06 to -0.05) | 0.03(0.01 to 0.04) |
| Canada | 252,390(208,901-301,492) | 4,388.1(3,631.99-5,241.79) | 262,783(215,518-317,094) | 4,257.79(3,491.96-5,137.78) | -0.09(-0.11 to -0.08) | -0.15(-0.17 to -0.13) | -0.26(-0.29 to -0.23) | 0.11(0.11 to 0.12) |
| United States of America | 2,276,374(1,996,246-2,554,265) | 4,071.47(3,570.44-4,568.5) | 2,420,604(2,130,230-2,719,762) | 4,072.74(3,584.17-4,576.08) | 0(-0.01 to 0.01) | 0.04(0.03 to 0.06) | -0.16(-0.18 to -0.14) | 0.1(0.07 to 0.12) |
| Antigua and Barbuda | 2,725(2,260-3,291) | 14,978.78(12,424.61-18,091.67) | 2,493(2,074-3,010) | 14,748.58(12,271.94-17,807.45) | -0.05(-0.06 to -0.04) | -0.11(-0.14 to -0.08) | 0(0 to 0.01) | -0.07(-0.08 to -0.05) |
| Bahamas | 12,486(10,318-15,069) | 15,479.94(12,792.09-18,682) | 12,440(10,297-15,064) | 15,323.43(12,683.54-18,555.32) | -0.03(-0.05 to -0.01) | -0.1(-0.16 to -0.04) | -0.09(-0.1 to -0.08) | 0.04(0.01 to 0.06) |
| Barbados | 9,419(7,822-11,400) | 15,103.37(12,542.40-18,280.55) | 6,959(5,801-8,424) | 14,777.85(12,319.13-17,889.8) | -0.07(-0.08 to -0.06) | -0.2(-0.23 to -0.18) | -0.04(-0.06 to -0.03) | 0.01(0 to 0.03) |
| Belize | 12,242(10,130-14,760) | 14,954.44(12,374.73-18,030.65) | 18,417(15,290-22,352) | 14,958.97(12,419.53-18,154.84) | 0(-0.02 to 0.02) | 0.11(0.09 to 0.14) | -0.14(-0.19 to -0.09) | -0.01(-0.03 to 0.01) |
| Cuba | 365,161(302,852-440,520) | 14,582.94(12,094.57-17,592.44) | 254,529(211,487-310,649) | 14,323.39(11,901.21-17,481.5) | -0.06(-0.07 to -0.05) | -0.04(-0.06 to -0.01) | -0.08(-0.1 to -0.05) | -0.03(-0.05 to -0.02) |
| Dominica | 3,800(3,135-4,569) | 15,312.37(12,633.23-18,414.12) | 2,128(1,756-2,582) | 15,553.52(12,835.23-18,871.04) | 0.05(0.04 to 0.06) | -0.02(-0.04 to 0) | 0.04(0.03 to 0.05) | 0.11(0.09 to 0.13) |
| Dominican Republic | 411,199(340,460-494,112) | 15,255.26(12,630.87-18,331.29) | 438,069(362,502-528,296) | 14,908.18(12,336.50-17,978.73) | -0.08(-0.09 to -0.06) | -0.26(-0.28 to -0.25) | 0.03(0 to 0.06) | -0.08(-0.1 to -0.05) |
| Grenada | 5,064(4,185-6,140) | 15,157.11(12,526.19-18,379.44) | 3,274(2,717-3,952) | 14,996.02(12,448.21-18,104.5) | -0.04(-0.04 to -0.03) | -0.13(-0.14 to -0.12) | 0.02(0.01 to 0.03) | -0.03(-0.05 to -0.01) |
| Guyana | 45,967(38,148-55,088) | 15,638.43(12,978.33-18,741.74) | 32,982(27,197-39,656) | 15,456.41(12,745.32-18,584.11) | -0.04(-0.05 to -0.03) | -0.05(-0.08 to -0.03) | 0.1(0.08 to 0.12) | -0.16(-0.19 to -0.14) |
| Haiti | 437,374(358,874-522,776) | 16,120.93(13,227.52-19,268.68) | 685,832(564,792-825,511) | 15,756.22(12,975.45-18,965.19) | -0.08(-0.09 to -0.08) | -0.12(-0.13 to -0.11) | -0.09(-0.1 to -0.09) | -0.04(-0.05 to -0.03) |
| Jamaica | 124,284(103,236-150,625) | 14,880.62(12,360.49-18,034.5) | 86,914(72,198-105,238) | 14,884.35(12,364.16-18,022.38) | 0(-0.01 to 0.01) | -0.03(-0.05 to -0.01) | -0.01(-0.03 to 0.02) | 0.02(0 to 0.05) |
| Saint Lucia | 7,843(6,484-9,475) | 15,215.84(12,579.34-18,382.82) | 4,434(3,684-5,378) | 14,935.35(12,407.83-18,114.45) | -0.06(-0.07 to -0.05) | -0.15(-0.18 to -0.12) | -0.02(-0.05 to 0) | -0.03(-0.05 to -0.01) |
| Saint Vincent and the Grenadines | 6,256(5,170-7,572) | 15,225.57(12,582.87-18,429.94) | 3,773(3,135-4,571) | 15,121.53(12,565.65-18,319.07) | -0.03(-0.04 to -0.01) | -0.08(-0.09 to -0.06) | -0.05(-0.08 to -0.03) | 0.05(0.03 to 0.07) |
| Suriname | 20,277(16,829-24,601) | 15,565.67(12,918.61-18,884.81) | 21,936(18,095-26,604) | 15,311.37(12,630.34-18,569.21) | -0.06(-0.07 to -0.04) | -0.07(-0.1 to -0.04) | -0.05(-0.07 to -0.03) | -0.02(-0.04 to -0.01) |
| Trinidad and Tobago | 63,033(51,543-76,346) | 15,513.11(12,685.40-18,789.78) | 41,230(34,045-49,963) | 15,134.67(12,497.36-18,340.47) | -0.09(-0.1 to -0.07) | 0.04(0 to 0.08) | -0.33(-0.34 to -0.31) | 0.07(0.05 to 0.09) |
| Bolivia (Plurinational State of) | 538,715(441,633-644,531) | 20,057.59(16,443.00-23,997.37) | 673,500(551,115-808,419) | 19,317.26(15,807.03-23,187.02) | -0.13(-0.14 to -0.12) | -0.09(-0.1 to -0.09) | -0.16(-0.18 to -0.15) | -0.09(-0.11 to -0.07) |
| Ecuador | 761,049(623,881-914,630) | 19,689.31(16,140.62-23,662.67) | 960,672(786,090-1,158,483) | 18,944.87(15,502.05-22,845.8) | -0.13(-0.14 to -0.11) | -0.39(-0.4 to -0.37) | 0.02(0 to 0.04) | -0.03(-0.05 to -0.01) |
| Peru | 1,677,569(1,369,733-2,011,553) | 20,209.47(16,501.01-24,232.94) | 1,824,790(1,484,503-2,205,820) | 19,132.99(15,565.08-23,128.12) | -0.18(-0.19 to -0.17) | -0.22(-0.23 to -0.21) | -0.14(-0.16 to -0.13) | -0.16(-0.18 to -0.14) |
| Colombia | 1,331,642(1,089,524-1,609,692) | 11,417.65(9,341.7-13,801.69) | 1,134,434(931,402-1,383,804) | 10,689.07(8,776.03-13,038.73) | -0.22(-0.22 to -0.21) | -0.39(-0.42 to -0.37) | -0.12(-0.14 to -0.11) | -0.16(-0.17 to -0.16) |
| Costa Rica | 122,244(100,320-148,095) | 10,873.97(8,923.73-13,173.49) | 108,211(88,825-132,468) | 10,637.14(8,731.50-13,021.54) | -0.07(-0.08 to -0.06) | -0.02(-0.03 to -0.01) | -0.16(-0.17 to -0.15) | -0.02(-0.03 to 0) |
| El Salvador | 249,571(204,940-301,936) | 11,564.46(9,496.38-13,990.92) | 196,669(162,181-240,086) | 10,813.15(8,916.95-13,200.29) | -0.22(-0.23 to -0.21) | -0.49(-0.5 to -0.48) | -0.13(-0.14 to -0.11) | -0.09(-0.1 to -0.08) |
| Guatemala | 483,745(397,659-584,267) | 11,911.3(9,791.59-14,386.45) | 557,307(455,394-676,476) | 11,295.19(9,229.67-13,710.43) | -0.17(-0.19 to -0.16) | -0.38(-0.39 to -0.36) | -0.13(-0.16 to -0.11) | -0.05(-0.08 to -0.02) |
| Honduras | 260,529(213,543-313,883) | 11,791.69(9,665.08-14,206.53) | 374,630(305,774-455,108) | 11,431.53(9,330.44-13,887.22) | -0.1(-0.11 to -0.09) | -0.19(-0.2 to -0.18) | -0.09(-0.12 to -0.05) | -0.06(-0.08 to -0.04) |
| Mexico | 3,882,241(3,190,095-4,633,689) | 11,617.82(9,546.54-13,866.58) | 3,874,374(3,160,545-4,678,774) | 12,082.14(9,856.08-14,590.64) | 0.13(0.1 to 0.15) | -0.45(-0.47 to -0.43) | -0.09(-0.1 to -0.08) | 1.02(0.95 to 1.1) |
| Nicaragua | 205,552(167,538-250,308) | 11,286.73(9,199.45-13,744.27) | 215,105(176,435-261,455) | 10,861.61(8,908.97-13,202.01) | -0.13(-0.14 to -0.12) | -0.04(-0.06 to -0.03) | -0.24(-0.25 to -0.23) | -0.07(-0.09 to -0.05) |
| Panama | 94,317(77,516-114,389) | 11,310.3(9,295.6-13,717.24) | 124,564(102,127-151,760) | 10,800.08(8,854.70-13,158.05) | -0.15(-0.16 to -0.14) | -0.31(-0.34 to -0.29) | -0.07(-0.08 to -0.06) | -0.1(-0.1 to -0.1) |
| Venezuela (Bolivarian Republic of) | 816,315(673,103-988,882) | 11,507.22(9,488.42-13,939.81) | 725,231(594,263-878,285) | 10,948.66(8,971.46-13,259.27) | -0.16(-0.17 to -0.14) | -0.33(-0.36 to -0.29) | -0.26(-0.28 to -0.23) | 0.07(0.06 to 0.09) |
| Brazil | 8,388,051(6,835,009-10,188,803) | 16,148.13(13,158.32-19,614.82) | 7,437,098(6,114,486-9,006,679) | 15,434.36(12,689.52-18,691.75) | -0.15(-0.15 to -0.14) | -0.23(-0.24 to -0.22) | -0.07(-0.07 to -0.06) | -0.13(-0.14 to -0.12) |
| Paraguay | 250,423(206,248-301,968) | 14,999.16(12,353.25-18,086.41) | 297,597(247,090-361,756) | 14,822.59(12,306.98-18,018.22) | -0.04(-0.04 to -0.04) | 0(0 to 0) | -0.05(-0.06 to -0.05) | -0.05(-0.06 to -0.04) |
| Algeria | 527,358(425,218-634,353) | 4,917.26(3,964.87-5,914.91) | 562,754(459,674-676,842) | 4,230.69(3,455.75-5,088.39) | -0.49(-0.5 to -0.48) | -0.67(-0.68 to -0.67) | -0.26(-0.28 to -0.24) | -0.6(-0.64 to -0.57) |
| Bahrain | 7,952(6,483-9,538) | 4,870.88(3,971.57-5,842.84) | 12,176(9,853-14,765) | 4,103.55(3,320.60-4,976.34) | -0.55(-0.57 to -0.54) | -0.61(-0.65 to -0.57) | -0.55(-0.56 to -0.54) | -0.53(-0.56 to -0.5) |
| Egypt | 1,434,728(1,171,611-1,759,405) | 6,468.06(5,281.87-7,931.77) | 1,560,240(1,339,772-1,814,051) | 4,233.44(3,635.23-4,922.1) | -1.37(-1.5 to -1.25) | -2.71(-3.04 to -2.38) | -0.26(-0.33 to -0.19) | -1.56(-1.76 to -1.35) |
| Iran (Islamic Republic of) | 1,280,401(1,037,068-1,528,407) | 5,044.02(4,085.43-6,021.02) | 879,614(723,250-1,061,459) | 4,358.97(3,584.10-5,260.11) | -0.47(-0.49 to -0.45) | -0.72(-0.75 to -0.69) | -0.12(-0.14 to -0.11) | -0.65(-0.7 to -0.59) |
| Iraq | 335,538(278,069-397,177) | 4,073.81(3,376.07-4,822.17) | 501,134(407,636-600,093) | 3,722.61(3,028.07-4,457.72) | -0.29(-0.3 to -0.29) | -0.13(-0.14 to -0.12) | -0.42(-0.43 to -0.42) | -0.28(-0.29 to -0.26) |
| Jordan | 79,109(64,162-94,825) | 4,843.26(3,928.14-5,805.43) | 150,108(121,296-178,775) | 4,131.76(3,338.70-4,920.82) | -0.51(-0.53 to -0.49) | -0.51(-0.54 to -0.48) | -0.64(-0.65 to -0.62) | -0.34(-0.39 to -0.29) |
| Kuwait | 24,905(20,314-29,703) | 4,492.76(3,664.44-5,358.26) | 33,934(27,565-41,058) | 4,013.54(3,260.3-4,856.17) | -0.36(-0.38 to -0.34) | -0.31(-0.34 to -0.28) | -0.25(-0.29 to -0.21) | -0.44(-0.47 to -0.41) |
| Lebanon | 49,115(40,222-58,747) | 4,696.01(3,845.75-5,617.03) | 51,614(41,955-62,417) | 4,038.48(3,282.68-4,883.75) | -0.49(-0.5 to -0.48) | -0.69(-0.7 to -0.68) | -0.37(-0.38 to -0.37) | -0.42(-0.46 to -0.38) |
| Libya | 85,353(69,919-102,218) | 4,713.19(3,860.9-5,644.49) | 63,044(50,924-76,107) | 4,226.54(3,414.03-5,102.3) | -0.35(-0.36 to -0.34) | -0.31(-0.32 to -0.29) | -0.41(-0.42 to -0.4) | -0.39(-0.41 to -0.36) |
| Morocco | 491,458(397,501-587,335) | 5,022.18(4,062.03-6,001.94) | 426,544(347,214-514,054) | 4,356.11(3,545.95-5,249.81) | -0.46(-0.47 to -0.45) | -0.5(-0.51 to -0.48) | -0.36(-0.37 to -0.36) | -0.52(-0.54 to -0.5) |
| Palestine | 45,578(37,252-54,424) | 4,707.04(3,847.25-5,620.62) | 77,659(63,194-93,913) | 4,159.19(3,384.46-5,029.71) | -0.4(-0.41 to -0.39) | -0.46(-0.47 to -0.45) | -0.24(-0.25 to -0.24) | -0.49(-0.5 to -0.47) |
| Oman | 40,102(32,616-48,549) | 4,771.98(3,881.18-5,777.02) | 50,689(40,847-61,043) | 4,144.58(3,339.89-4,991.23) | -0.46(-0.47 to -0.44) | -0.7(-0.71 to -0.69) | -0.07(-0.11 to -0.03) | -0.65(-0.7 to -0.61) |
| Qatar | 5,989(4,912-7,217) | 4,789.79(3,928.34-5,771.45) | 19,973(16,371-23,884) | 4,043.95(3,314.65-4,835.87) | -0.55(-0.55 to -0.54) | -0.43(-0.44 to -0.42) | -0.54(-0.56 to -0.52) | -0.67(-0.69 to -0.66) |
| Saudi Arabia | 331,156(266,977-399,900) | 5,053.21(4,073.89-6,102.2) | 320,452(259,330-385,734) | 4,235.83(3,427.90-5,098.75) | -0.57(-0.58 to -0.56) | -0.63(-0.64 to -0.62) | -0.56(-0.57 to -0.55) | -0.56(-0.57 to -0.54) |
| Syrian Arab Republic | 284,957(231,656-341,685) | 4,811.76(3,911.72-5,769.66) | 151,522(123,958-181,118) | 4,136.20(3,383.75-4,944.11) | -0.49(-0.5 to -0.47) | -0.63(-0.65 to -0.61) | -0.54(-0.57 to -0.52) | -0.37(-0.41 to -0.33) |
| Tunisia | 144,504(118,158-172,850) | 4,653.18(3,804.83-5,565.95) | 114,232(92,392-138,381) | 4,130.29(3,340.63-5,003.42) | -0.38(-0.39 to -0.37) | -0.47(-0.48 to -0.45) | -0.26(-0.27 to -0.25) | -0.45(-0.48 to -0.42) |
| Turkey | 1,038,920(872,147-1,226,066) | 5,070.7(4,256.72-5,984.11) | 777,873(638,263-937,846) | 4,200.08(3,446.27-5,063.85) | -0.61(-0.66 to -0.55) | -0.72(-0.75 to -0.69) | -0.93(-0.98 to -0.88) | -0.27(-0.45 to -0.08) |
| United Arab Emirates | 30,304(24,433-36,857) | 5,141.68(4,145.49-6,253.39) | 58,671(47,798-70,921) | 4,382.38(3,570.24-5,297.36) | -0.51(-0.54 to -0.48) | -0.47(-0.51 to -0.43) | -0.3(-0.32 to -0.29) | -0.81(-0.9 to -0.72) |
| Yemen | 357,413(288,321-429,333) | 5,037.98(4,064.09-6,051.75) | 623,330(507,706-745,019) | 4,520.64(3,682.08-5,403.18) | -0.35(-0.36 to -0.33) | -0.38(-0.39 to -0.36) | -0.28(-0.3 to -0.26) | -0.36(-0.4 to -0.32) |
| Afghanistan | 232,085(188,594-279,962) | 5,387.21(4,377.69-6,498.55) | 671,222(549,010-810,179) | 4,726.7(3,866.1-5,705.24) | -0.42(-0.44 to -0.4) | -0.08(-0.12 to -0.03) | -0.61(-0.63 to -0.58) | -0.5(-0.55 to -0.46) |
| Bangladesh | 8,581,009(6,778,595-11,024,963) | 17,543.96(13,858.91-22,540.65) | 6,956,448(5,538,829-8,683,847) | 15,200.04(12,102.5-18,974.46) | -0.47(-0.48 to -0.45) | -0.42(-0.43 to -0.42) | -0.54(-0.58 to -0.49) | -0.36(-0.4 to -0.31) |
| Bhutan | 44,657(35,597-56,657) | 17,032.14(13,576.53-21,608.59) | 28,513(22,961-35,261) | 15,234.12(12,267.63-18,839.48) | -0.36(-0.38 to -0.35) | -0.2(-0.22 to -0.18) | -0.53(-0.55 to -0.51) | -0.37(-0.41 to -0.33) |
| India | 68,925,714(53,437,423-89,581,315) | 21,109.12(16,365.69-27,435.09) | 69,469,952(54,563,173-87,567,178) | 18,960.19(14,891.73-23,899.4) | -0.35(-0.37 to -0.33) | -0.23(-0.25 to -0.21) | -0.41(-0.42 to -0.4) | -0.38(-0.43 to -0.32) |
| Nepal | 1,457,799(1,144,994-1,879,985) | 17,302.88(13,590.15-22,313.89) | 1,438,720(1,155,440-1,810,422) | 15,592.12(12,522.08-19,620.44) | -0.34(-0.37 to -0.31) | -0.56(-0.63 to -0.49) | -0.3(-0.36 to -0.23) | -0.18(-0.2 to -0.16) |
| Pakistan | 9,125,045(7,202,285-11,673,784) | 18,531(14,626.29-23,706.94) | 15,417,654(12,252,472-19,679,988) | 18,044.57(14,340.09-23,033.13) | -0.1(-0.11 to -0.08) | 0.21(0.18 to 0.24) | -0.28(-0.3 to -0.25) | -0.18(-0.21 to -0.14) |
| Angola | 1,803,366(1,382,689-2,420,434) | 38,249.07(29,326.59-51,336.98) | 5,303,462(4,042,629-7,100,223) | 34,787.13(26,516.92-46,572.67) | -0.29(-0.42 to -0.17) | 0.33(0.25 to 0.41) | -0.13(-0.35 to 0.08) | -1.13(-1.45 to -0.81) |
| Central African Republic | 479,074(367,343-630,664) | 39,182.43(30,044.19-51,580.66) | 838,962(652,074-1,106,227) | 36,735.01(28,551.88-48,437.55) | -0.18(-0.44 to 0.09) | 0.36(0.22 to 0.51) | 0.01(-0.5 to 0.53) | -0.97(-1.6 to -0.32) |
| Congo | 408,838(315,660-544,877) | 38,827.18(29,978.07-51,746.72) | 671,532(510,427-889,781) | 34,807.5(26,456.95-46,120.03) | -0.32(-0.58 to -0.06) | 0.29(0.15 to 0.43) | -0.22(-0.72 to 0.28) | -1.07(-1.7 to -0.44) |
| Democratic Republic of the Congo | 6,670,738(5,064,187-8,983,864) | 37,679.77(28,605.14-50,745.5) | 13,049,290(9,763,697-17,426,326) | 34,341.21(25,694.67-45,860.06) | -0.29(-0.48 to -0.1) | 0.18(0.08 to 0.29) | -0.07(-0.45 to 0.31) | -1.04(-1.5 to -0.58) |
| Equatorial Guinea | 76,297(58,679-102,077) | 38,746.03(29,798.95-51,837.85) | 196,086(148,987-260,537) | 33,519.03(25,467.94-44,536.31) | -0.46(-0.63 to -0.29) | 0.1(0 to 0.19) | -0.56(-0.89 to -0.22) | -0.98(-1.39 to -0.57) |
| Gabon | 153,566(120,702-202,049) | 37,684.5(29,619.94-49,582.23) | 206,616(167,381-270,233) | 32,327.18(26,188.51-42,280.74) | -0.48(-0.75 to -0.21) | 0.4(-0.02 to 0.82) | -0.21(-0.68 to 0.26) | -2.06(-2.6 to -1.52) |
| Burundi | 1,041,401(826,806-1,350,855) | 39,731.06(31,543.91-51,537.18) | 2,218,921(1,751,926-2,838,864) | 37,903.33(29,926.18-48,493.12) | -0.17(-0.2 to -0.14) | -0.19(-0.22 to -0.16) | -0.13(-0.16 to -0.09) | -0.25(-0.32 to -0.18) |
| Comoros | 82,732(66,347-105,690) | 38,897.63(31,193.8-49,691.42) | 90,949(71,988-115,590) | 37,871.13(29,975.8-48,131.43) | -0.1(-0.14 to -0.06) | 0.21(0.15 to 0.27) | -0.39(-0.44 to -0.33) | -0.2(-0.32 to -0.08) |
| Djibouti | 66,505(53,252-85,037) | 38,196.56(30,584.44-48,839.9) | 154,017(122,859-197,145) | 37,276.97(29,735.79-47,715.22) | -0.12(-0.18 to -0.05) | 0.15(0.04 to 0.26) | 0.07(-0.07 to 0.21) | -0.53(-0.6 to -0.46) |
| Eritrea | 638,184(507,376-829,451) | 40,089.89(31,872.7-52,105.04) | 993,813(789,419-1,283,134) | 39,364.72(31,268.71-50,824.69) | -0.09(-0.13 to -0.05) | -0.02(-0.08 to 0.03) | 0.06(0.01 to 0.12) | -0.37(-0.48 to -0.26) |
| Ethiopia | 16,533,658(13,339,180-20,846,596) | 67,861.29(54,749.77-85,563.46) | 26,404,188(21,409,185-32,948,142) | 59,535.35(48,272.77-74,290.46) | -0.42(-0.46 to -0.39) | -0.14(-0.18 to -0.1) | -0.68(-0.73 to -0.62) | -0.5(-0.57 to -0.42) |
| Kenya | 3,778,980(2,959,801-4,875,682) | 33,831.05(26,497.4-43,649.2) | 7,001,451(5,453,044-8,878,346) | 37,510.63(29,214.97-47,566.2) | 0.3(0.16 to 0.44) | 0.05(-0.05 to 0.16) | 0.65(0.44 to 0.87) | 0.39(0.11 to 0.68) |
| Madagascar | 1,720,433(1,362,971-2,130,774) | 31,533.05(24,981.30-39,054.01) | 3,551,658(2,840,155-4,408,568) | 30,269.32(24,205.47-37,572.41) | -0.15(-0.19 to -0.1) | -0.24(-0.31 to -0.18) | -0.06(-0.09 to -0.03) | -0.2(-0.32 to -0.07) |
| Malawi | 1,763,130(1,405,020-2,266,317) | 38,753.36(30,882.16-49,813.34) | 3,045,892(2,428,773-3,910,128) | 37,493.15(29,896.77-48,131.4) | -0.13(-0.17 to -0.09) | -0.05(-0.09 to -0.01) | 0.01(-0.03 to 0.05) | -0.34(-0.46 to -0.23) |
| Mauritius | 52,435(43,083-62,795) | 15,886.66(13,053.32-19,025.75) | 32,611(26,738-39,150) | 15,723.19(12,891.71-18,876.3) | -0.04(-0.07 to -0.01) | -0.34(-0.39 to -0.28) | 0.2(0.14 to 0.26) | -0.08(-0.11 to -0.04) |
| Mozambique | 2,225,127(1,760,026-2,772,197) | 35,865.1(28,368.51-44,682.91) | 5,115,164(4,094,145-6,312,494) | 35,855.61(28,698.6-44,248.5) | -0.01(-0.08 to 0.05) | -0.1(-0.18 to -0.02) | 0.19(0.12 to 0.27) | -0.2(-0.38 to -0.02) |
| Rwanda | 1,674,161(1,330,376-2,116,700) | 49,342.99(39,210.51-62,386.04) | 2,191,084(1,668,066-2,805,753) | 44,082.45(33,559.83-56,448.98) | -0.41(-0.49 to -0.33) | -0.24(-0.32 to -0.15) | -0.62(-0.74 to -0.5) | -0.26(-0.45 to -0.07) |
| Seychelles | 3,804(3,132-4,544) | 16,030.92(13,200.71-19,149.1) | 3,646(2,990-4,386) | 15,575.57(12,775.06-18,737.74) | -0.09(-0.11 to -0.08) | -0.04(-0.06 to -0.02) | -0.29(-0.31 to -0.26) | 0.06(0.05 to 0.08) |
| Somalia | 1,504,712(1,194,302-1,935,178) | 38,625.41(30,657.3-49,675.32) | 3,905,285(3,121,246-4,955,557) | 37,807.37(30,217.03-47,975.13) | -0.09(-0.12 to -0.05) | -0.06(-0.11 to -0.02) | 0.05(0.01 to 0.09) | -0.29(-0.38 to -0.2) |
| United Republic of Tanzania | 4,332,475(3,424,145-5,571,128) | 35,878.16(28,356.08-46,135.72) | 9,572,359(7,587,884-12,264,692) | 39,226.3(31,094.18-50,259.14) | 0.21(0.08 to 0.33) | 1.17(0.92 to 1.43) | -0.02(-0.27 to 0.23) | -0.36(-0.48 to -0.24) |
| Uganda | 3,113,329(2,485,473-3,985,087) | 36,977.06(29,520-47,330.94) | 7,358,120(5,799,754-9,578,145) | 37,095.33(29,238.96-48,287.4) | -0.02(-0.08 to 0.04) | 0.22(0.15 to 0.29) | -0.06(-0.1 to -0.03) | -0.26(-0.43 to -0.1) |
| Zambia | 1,478,468(1,178,592-1,898,956) | 39,378.17(31,391.13-50,577.63) | 3,152,632(2,495,988-4,009,915) | 38,114.12(30,175.55-48,478.35) | -0.13(-0.18 to -0.07) | 0.15(0.11 to 0.2) | -0.03(-0.1 to 0.04) | -0.48(-0.63 to -0.32) |
| Botswana | 128,820(103,963-158,910) | 21,817.51(17,607.54-26,913.64) | 150,710(121,645-184,077) | 21,583.09(17,420.67-26,361.57) | -0.1(-0.17 to -0.03) | 0.4(0.25 to 0.56) | -0.11(-0.23 to 0) | -0.54(-0.67 to -0.41) |
| Lesotho | 147,177(118,227-181,452) | 21,561.56(17,320.3-26,582.89) | 143,548(115,488-175,952) | 22,767.77(18,317.28-27,907.22) | 0.11(0.02 to 0.2) | 0.29(0.23 to 0.36) | 0.49(0.28 to 0.69) | -0.41(-0.51 to -0.31) |
| Namibia | 132,261(107,226-163,878) | 22,016.71(17,849.31-27,279.72) | 173,490(141,486-210,505) | 21,018.23(17,140.97-25,502.59) | -0.16(-0.23 to -0.1) | 0.09(0.01 to 0.16) | -0.13(-0.17 to -0.09) | -0.46(-0.65 to -0.28) |
| South Africa | 3,039,469(2,462,349-3,696,418) | 22,326.86(18,087.54-27,152.58) | 3,335,217(2,700,054-4,039,119) | 21,934.74(17,757.47-26,564.1) | -0.08(-0.11 to -0.05) | 0.44(0.42 to 0.47) | -0.19(-0.25 to -0.14) | -0.5(-0.57 to -0.44) |
| Eswatini | 83,241(67,903-102,674) | 21,579.51(17,603.2-26,617.34) | 90,995(73,499-111,853) | 22,052.30(17,812.07-27,107.06) | 0.03(-0.03 to 0.1) | 0.25(0.23 to 0.27) | 0.25(0.23 to 0.27) | -0.39(-0.6 to -0.18) |
| Zimbabwe | 1,035,830(832,633-1,268,949) | 21,506.71(17,287.77-26,346.9) | 1,380,694(1,114,088-1,698,094) | 21,937.2(17,701.22-26,980.21) | 0.01(-0.05 to 0.07) | -0.02(-0.11 to 0.07) | 0.42(0.27 to 0.57) | -0.27(-0.34 to -0.19) |
| Benin | 868,313(676,361-1,125,291) | 35,854.35(27,928.26-46,465.48) | 2,086,333(1,635,909-2,704,455) | 34,312.45(26,904.65-44,478.27) | -0.16(-0.17 to -0.14) | 0.04(0.03 to 0.05) | -0.43(-0.45 to -0.41) | -0.21(-0.25 to -0.18) |
| Burkina Faso | 1,626,550(1,277,500-2,061,181) | 34,467.69(27,071.09-43,677.8) | 3,542,854(2,793,845-4,510,854) | 34,157.31(26,935.98-43,489.99) | -0.05(-0.07 to -0.03) | 0.04(0.02 to 0.06) | -0.29(-0.34 to -0.24) | -0.01(-0.07 to 0.05) |
| Cameroon | 1,520,004(1,193,761-1,943,041) | 31,134.6(24,452.09-39,799.78) | 3,975,661(3,110,803-4,839,014) | 29,521.7(23,099.6-35,932.61) | -0.18(-0.23 to -0.13) | 0.18(0.09 to 0.26) | -0.44(-0.52 to -0.36) | -0.43(-0.54 to -0.31) |
| Cabo Verde | 51,196(40,195-65,795) | 32,541.64(25,548.63-41,820.84) | 44,651(34,959-55,593) | 31,180.19(24,412.18-38,821.75) | -0.16(-0.24 to -0.09) | 0.19(0.06 to 0.32) | -0.48(-0.64 to -0.33) | -0.18(-0.28 to -0.07) |
| Chad | 1,021,695(806,322-1,312,654) | 34,913.47(27,553.72-44,856.15) | 3,114,972(2,445,237-4,003,912) | 34,553.6(27,124.4-44,414.4) | -0.05(-0.09 to -0.02) | 0.37(0.34 to 0.39) | -0.25(-0.32 to -0.19) | -0.37(-0.47 to -0.27) |
| Coted'Ivoire | 1,969,040(1,509,320-2,465,628) | 34,524.56(26,463.97-43,231.58) | 4,156,853(3,229,398-5,247,561) | 35,922.32(27,907.52-45,347.9) | 0.15(0.09 to 0.2) | 0.6(0.52 to 0.69) | -0.73(-0.8 to -0.66) | 0.62(0.48 to 0.76) |
| Gambia | 158,824(124,838-203,468) | 34,431.84(27,063.95-44,110.3) | 335,805(265,557-421,516) | 33,801.94(26,730.85-42,429.57) | -0.09(-0.15 to -0.03) | 0.16(0.09 to 0.23) | -0.29(-0.46 to -0.13) | -0.26(-0.33 to -0.19) |
| Ghana | 1,899,432(1,378,063-2,460,568) | 28,279.74(20,517.32-36,634.21) | 3,490,360(2,567,345-4,443,042) | 27,092.59(19,928.03-34,487.42) | -0.15(-0.26 to -0.05) | -0.29(-0.46 to -0.13) | -0.23(-0.38 to -0.07) | -0.17(-0.43 to 0.08) |
| Guinea | 986,905(781,234-1,277,045) | 35,863.44(28,389.52-46,406.95) | 2,133,216(1,668,455-2,705,732) | 35,284.54(27,597.15-44,754.25) | -0.07(-0.12 to -0.01) | 0.16(0.08 to 0.25) | -0.34(-0.42 to -0.26) | -0.17(-0.31 to -0.03) |
| Guinea-Bissau | 180,071(140,461-230,001) | 37,328.15(29,117.25-47,678.56) | 327,305(255,317-425,962) | 36,442.18(28,427.12-47,426.67) | -0.13(-0.22 to -0.05) | 0.22(0.11 to 0.32) | -0.28(-0.46 to -0.1) | -0.36(-0.45 to -0.26) |
| Liberia | 406,611(318,128-525,153) | 35,977.6(28,148.53-46,466.4) | 733,008(580,090-916,023) | 33,532.97(26,537.43-41,905.37) | -0.29(-0.38 to -0.2) | -0.27(-0.4 to -0.14) | -0.43(-0.61 to -0.25) | -0.3(-0.42 to -0.17) |
| Mali | 1,982,412(1,616,127-2,526,725) | 48,002.5(39,133.20-61,182.6) | 5,584,190(4,466,900-7,072,214) | 48,240.26(38,588.31-61,094.89) | -0.03(-0.11 to 0.05) | 0.15(0 to 0.3) | -0.1(-0.2 to 0) | -0.24(-0.36 to -0.13) |
| Mauritania | 336,954(267,004-429,928) | 36,452.72(28,885.24-46,510.91) | 612,776(473,725-770,900) | 33,069.14(25,565.10-41,602.5) | -0.33(-0.38 to -0.28) | -0.23(-0.27 to -0.19) | -0.34(-0.41 to -0.27) | -0.44(-0.58 to -0.31) |
| Niger | 1,673,933(1,305,648-2,089,744) | 41,199.94(32,135.48-51,434.16) | 4,913,013(3,899,497-5,945,849) | 38,491.19(30,550.76-46,582.98) | -0.24(-0.27 to -0.21) | -0.07(-0.09 to -0.04) | -0.7(-0.75 to -0.64) | -0.07(-0.15 to 0.01) |
| Nigeria | 17,504,736(14,147,728-22,015,757) | 44,741.05(36,160.74-56,270.95) | 46,154,593(36,989,898-57,589,700) | 45,432.64(36,411.3-56,688.88) | 0.04(-0.01 to 0.09) | 0.63(0.56 to 0.71) | -0.31(-0.38 to -0.24) | -0.28(-0.41 to -0.16) |
| Sao Tome and Principe | 19,785(15,534-25,418) | 34,915.14(27,413.09-44,854.96) | 25,738(20,054-32,131) | 33,069.37(25,765.83-41,282.19) | -0.22(-0.3 to -0.14) | -0.03(-0.12 to 0.07) | -0.37(-0.58 to -0.16) | -0.48(-0.59 to -0.38) |
| Senegal | 1,282,899(1,013,541-1,643,998) | 35,135.55(27,758.49-45,025.22) | 2,172,144(1,699,210-2,760,348) | 34,150.15(26,714.74-43,397.82) | -0.12(-0.19 to -0.05) | -0.06(-0.16 to 0.03) | -0.18(-0.33 to -0.03) | -0.2(-0.28 to -0.12) |
| Sierra Leone | 637,211(503,313-819,141) | 35,153.98(27,767.06-45,190.79) | 1,232,674(967,181-1,578,762) | 34,468.74(27,044.87-44,146.27) | -0.09(-0.17 to -0.01) | 0.03(-0.04 to 0.11) | -0.06(-0.15 to 0.04) | -0.27(-0.48 to -0.07) |
| Togo | 624,101(486,905-802,705) | 35,409.24(27,625.23-45,542.62) | 1,145,337(896,660-1,444,915) | 34,612.34(27,097.27-43,665.65) | -0.09(-0.12 to -0.07) | 0.22(0.2 to 0.24) | -0.38(-0.43 to -0.33) | -0.23(-0.3 to -0.16) |
| American Samoa | 2,163(1,782-2,576) | 11,372(9,367.21-13,542.56) | 1,653(1,357-1,965) | 11,656.88(9,568.87-13,856.25) | 0.08(0.06 to 0.1) | -0.05(-0.07 to -0.04) | 0.16(0.13 to 0.19) | 0.14(0.1 to 0.18) |
| Bermuda | 1,744(1,439-2,107) | 14,632.93(12,071.33-17,676.57) | 1,194(991-1,453) | 14,155.38(11,748.02-17,217.22) | -0.11(-0.11 to -0.1) | -0.29(-0.31 to -0.28) | -0.14(-0.15 to -0.12) | 0.05(0.04 to 0.06) |
| Cook Islands | 742(606-886) | 11,244.69(9,191.60-13,428.71) | 409(337-487) | 10,795.1(8,899.27-12,846.57) | -0.13(-0.14 to -0.12) | -0.29(-0.3 to -0.28) | -0.05(-0.07 to -0.04) | 0.04(0.02 to 0.06) |
| Greenland | 700(574-827) | 4,923.31(4,037.89-5,815.78) | 544(449-650) | 4,625.73(3,818.14-5,526.69) | -0.2(-0.22 to -0.18) | -0.22(-0.26 to -0.17) | -0.36(-0.38 to -0.34) | -0.07(-0.12 to -0.01) |
| Guam | 4,517(3,740-5,362) | 10,829.98(8,966.83-12,857.1) | 4,015(3,316-4,773) | 10,977.2(9,064.95-13,047.84) | 0.05(0.03 to 0.07) | -0.15(-0.21 to -0.08) | 0.22(0.19 to 0.24) | 0.04(0.03 to 0.04) |
| Monaco | 454(370-559) | 12,870.44(10,496.1-15,856.1) | 624(505-771) | 12,534.59(10,136.82-15,477.09) | -0.09(-0.09 to -0.08) | -0.12(-0.14 to -0.1) | -0.06(-0.07 to -0.06) | -0.07(-0.09 to -0.06) |
| Nauru | 498(410-597) | 11,798.41(9,709.44-14,150.04) | 463(378-553) | 11,645.83(9,509.43-13,911.06) | -0.04(-0.05 to -0.04) | 0.15(0.14 to 0.16) | -0.05(-0.07 to -0.04) | -0.23(-0.24 to -0.21) |
| Niue | 94(76-113) | 11,595.82(9,364.92-14,023.49) | 44(36-52) | 11,348.13(9,323.75-13,392.95) | -0.07(-0.09 to -0.05) | -0.02(-0.06 to 0.03) | -0.27(-0.29 to -0.24) | -0.01(-0.03 to 0.01) |
| Northern Mariana Islands | 1,339(1,109-1,590) | 11,010.9(9,118.37-13,073.32) | 1,266(1,042-1,508) | 11,243.09(9,253.54-13,388.21) | 0.07(-0.05 to 0.19) | -0.41(-0.69 to -0.13) | 0.55(0.5 to 0.6) | 0.01(-0.19 to 0.22) |
| Palau | 530(436-631) | 11,632.98(9,565.04-13,842.36) | 373(306-444) | 11,454.52(9,372.21-13,607.85) | -0.05(-0.07 to -0.03) | -0.24(-0.29 to -0.19) | -0.01(-0.06 to 0.03) | 0.11(0.1 to 0.12) |
| Puerto Rico | 147,509(122,737-178,567) | 14,813.58(12,325.80-17,932.56) | 65,177(53,101-79,430) | 14,666.57(11,949.26-17,873.99) | -0.03(-0.05 to -0.02) | -0.17(-0.19 to -0.16) | -0.11(-0.12 to -0.09) | 0.18(0.14 to 0.21) |
| Saint Kitts and Nevis | 2,233(1,839-2,704) | 15,815.69(13,025.88-19,147.66) | 1,484(1,231-1,798) | 15,069.62(12,502.7-18,264.76) | -0.15(-0.16 to -0.15) | -0.3(-0.32 to -0.28) | -0.12(-0.13 to -0.1) | -0.1(-0.1 to -0.09) |
| San Marino | 533(429-656) | 13,017.99(10,479.21-16,013.38) | 561(448-695) | 12,763.63(10,184.35-15,793.73) | -0.06(-0.07 to -0.05) | -0.38(-0.39 to -0.36) | -0.02(-0.04 to -0.01) | 0.14(0.12 to 0.15) |
| Tokelau | 70(57-84) | 11,662.37(9,432.01-13,999.62) | 45(36-53) | 11,406.96(9,279.74-13,627.9) | -0.08(-0.12 to -0.04) | -0.07(-0.16 to 0.01) | -0.12(-0.19 to -0.04) | -0.13(-0.17 to -0.1) |
| Tuvalu | 411(340-491) | 11,829.6(9,790.9-14,134.96) | 429(351-511) | 11,515.38(9,409.46-13,712.26) | -0.09(-0.09 to -0.08) | -0.08(-0.09 to -0.07) | -0.07(-0.09 to -0.06) | -0.15(-0.16 to -0.15) |
| United States Virgin Islands | 4,834(4,016-5,807) | 15,130.69(12,570.39-18,176.18) | 2,006(1,669-2,442) | 14,983.60(12,464.92-18,238.54) | -0.03(-0.04 to -0.03) | -0.13(-0.15 to -0.12) | -0.01(-0.02 to -0.01) | 0.04(0.02 to 0.05) |
| South Sudan | 1,008,931(801,869-1,275,866) | 38,447.87(30,557.21-48,620.08) | 1,577,238(1,251,262-1,994,053) | 36,723.07(29,133.32-46,427.83) | -0.17(-0.19 to -0.15) | -0.07(-0.09 to -0.05) | 0(-0.02 to 0.02) | -0.47(-0.53 to -0.4) |
| Sudan | 453,701(366,564-546,156) | 5,102.1(4,122.19-6,141.8) | 741,434(603,770-884,334) | 4,469.35(3,639.52-5,330.76) | -0.43(-0.44 to -0.42) | -0.51(-0.53 to -0.48) | -0.27(-0.28 to -0.26) | -0.53(-0.56 to -0.51) |

**sTable5 The incident cases and incidence rate of viral skin diseases and their AAPCs from 1990 to 2021 at the global, regional and national levels**

| *Rate per 100,000* | 1990 | | 2021 | | 1990-2021 | 1990-1999 | 2000-2009 | 2010-2021 |
| --- | --- | --- | --- | --- | --- | --- | --- | --- |
| *AAPC(95% CI)* | Incident cases | Incidence rate | Incident cases | Incidence rate | AAPC | AAPC | AAPC | AAPC |
| Viral skin diseases | 37,742,170(35,868,260-39,735,703) | 2,170.15(2,062.4-2,284.78) | 44,470,228(42,254,333-46,938,624) | 2,210.4(2,100.26-2,333.09) | 0.06(0.05 to 0.07) | -0.09(-0.11 to -0.07) | 0.11(0.09 to 0.13) | 0.15(0.14 to 0.16) |
| Male | 19,193,666(18,233,763-20,193,548) | 2,148.16(2,040.73-2,260.07) | 22,550,156(21,440,669-23,744,825) | 2,172.12(2,065.25-2,287.19) | 0.04(0.03 to 0.04) | -0.11(-0.12 to -0.09) | 0.08(0.07 to 0.09) | 0.12(0.11 to 0.14) |
| Female | 18,548,504(17,642,189-19,543,552) | 2,193.38(2,086.2-2,311.04) | 21,920,072(20,838,086-23,134,062) | 2,251.22(2,140.1-2,375.9) | 0.08(0.07 to 0.1) | -0.08(-0.1 to -0.06) | 0.15(0.13 to 0.17) | 0.17(0.14 to 0.19) |
| **Age groups** |  |  |  |  |  |  |  |  |
| 0-4 years | 14,662,641(13,856,426-15,515,370) | 2,365.18(2,235.13-2,502.73) | 16,255,029(15,349,130-17,231,438) | 2469.73(2332.09-2618.08) | 0.14 (0.12 to 0.16) | 0.05(0.03 to 0.07) | 0.03(0.02 to 0.04) | 0.3(0.26 to 0.34) |
| 5-9 years | 16,148,535(15,113,522-17,185,020) | 2,767.38(2,590.01-2,945.01) | 19,489,684(18,234,986-20,799,099) | 2,836.71(2,654.08-3,027.29) | 0.08 (0.07-0.08) | 0.11(0.1 to 0.11) | 0.04(0.04 to 0.05) | 0.09(0.09 to 0.1) |
| 10-14 years | 6,930,993(6,459,512-7,479,452) | 1293.86(1205.85-1396.25) | 8,725,514(8,139,838-9,429,367) | 1308.89(1221.03-1414.47) | 0.04 (0.03-0.04) | 0.05(0.05 to 0.06) | -0.02(-0.03 to -0.01) | 0.06(0.05 to 0.08) |
| **SDI region** |  |  |  |  |  |  |  |  |
| High SDI | 5,484,309(5,264,139-5,719,944) | 2,951.6(2,833.11-3,078.42) | 5,172,118(4,963,384-5,380,899) | 2,997.69(2,876.72-3,118.7) | 0.04 (0.02 to 0.06) | 0.01(-0.02 to 0.04) | -0.07(-0.08 to -0.06) | 0.17(0.13 to 0.22) |
| High-middle SDI | 6,086,697(5,767,959-6,426,629) | 2,224.48(2,107.99-2,348.72) | 5,428,890(5,141,985-5,747,686) | 2,351.29(2,227.02-2,489.36) | 0.18 (0.16 to 0.2) | -0.14(-0.18 to -0.11) | 0.44(0.41 to 0.47) | 0.24(0.21 to 0.28) |
| Middle SDI | 11,966,093(11,349,810-12,653,995) | 2,073.08(1,966.31-2,192.25) | 12,258,168(11,621,246-12,998,128) | 2,162.46(2,050.11-2,293) | 0.14 (0.11 to 0.16) | -0.04(-0.09 to 0.01) | 0.21(0.16 to 0.26) | 0.24(0.22 to 0.26) |
| Low-middle SDI | 9,428,166(8,932,431-9,944,641) | 1,997.02(1,892.01-2,106.42) | 11,762,324(11,158,956-12,447,971) | 2,028.54(1,924.48-2,146.79) | 0.05 (0.04 to 0.06) | -0.02(-0.03 to -0.01) | 0.05(0.04 to 0.06) | 0.1(0.07 to 0.12) |
| Low SDI | 4,746,574(4,520,909-4,994,350) | 2,073.52(1,974.94-2,181.76) | 9,816,889(9,368,950-10,333,758) | 2,133.06(2,035.73-2,245.37) | 0.09 (0.09 to 0.1) | -0.02(-0.04 to -0.01) | 0.16(0.15 to 0.16) | 0.13(0.12 to 0.13) |
| **GBD region** |  |  |  |  |  |  |  |  |
| Andean Latin America | 293,878(277,209-313,092) | 1,978.71(1,866.47-2,108.08) | 369,255(348,126-391,866) | 2,040.67(1,923.9-2,165.63) | 0.1 (0.07 to 0.13) | 0.12(0.1 to 0.15) | -0.03(-0.07 to 0.01) | 0.19(0.15 to 0.23) |
| Australasia | 148,925(141,177-156,406) | 3,247.41(3,078.46-3,410.54) | 189,165(179,077-199,684) | 3,300.65(3,124.63-3,484.19) | 0.06 (0.05 to 0.07) | 0.08(0.07 to 0.09) | -0.13(-0.15 to -0.11) | 0.19(0.17 to 0.21) |
| Caribbean | 216,824(204,712-230,010) | 1,899.9(1,793.77-2,015.43) | 219,034(207,034-232,662) | 1,903.8(1,799.53-2,022.25) | 0 (-0.01 to 0.02) | -0.05(-0.07 to -0.02) | 0.01(-0.02 to 0.04) | 0.07(0.04 to 0.1) |
| Central Asia | 508,645(480,688-539,112) | 2,035.3(1,923.43-2,157.21) | 560,310(529,826-593,062) | 2,024.55(1,914.44-2,142.89) | -0.02 (-0.05 to 0.01) | -0.31(-0.34 to -0.29) | -0.17(-0.21 to -0.12) | 0.32(0.26 to 0.39) |
| Central Europe | 608,966(577,480-642,803) | 2,065.44(1,958.65-2,180.2) | 370,488(351,984-392,442) | 2,093.01(1,988.47-2,217.03) | 0.04 (0.02 to 0.05) | -0.09(-0.11 to -0.07) | 0.16(0.14 to 0.18) | 0.01(-0.03 to 0.04) |
| Central Latin America | 1,157,923(1,099,869-1,226,251) | 1,798.54(1,708.37-1,904.67) | 1,172,523(1,108,804-1,245,989) | 1,846.93(1,746.57-1,962.65) | 0.08 (0.07 to 0.1) | 0.17(0.15 to 0.19) | -0.05(-0.07 to -0.04) | 0.13(0.1 to 0.15) |
| Central Sub-Saharan Africa | 449,073(427,013-479,239) | 1,775.09(1,687.89-1,894.33) | 1,106,080(1,053,058-1,177,557) | 1,884.88(1,794.53-2,006.69) | 0.19 (0.18 to 0.21) | -0.05(-0.07 to -0.04) | 0.25(0.23 to 0.26) | 0.35(0.33 to 0.38) |
| East Asia | 7,382,538(7,002,884-7,775,946) | 2,238.26(2,123.16-2,357.53) | 6,584,058(6,239,595-6,980,136) | 2,462.69(2,333.85-2,610.84) | 0.3(0.26 to 0.34) | -0.15(-0.2 to -0.09) | 0.68(0.62 to 0.73) | 0.38(0.32 to 0.44) |
| Eastern Europe | 1,093,306(1,034,730-1,155,817) | 2,124.49(2,010.67-2,245.96) | 743,805(704,193-787,198) | 2,098.53(1,986.78-2,220.96) | -0.04 (-0.08 to 0) | -0.96(-1.03 to -0.89) | 0.66(0.59 to 0.73) | 0.09(0.04 to 0.14) |
| Eastern Sub-Saharan Africa | 2,253,584(2,151,021-2,377,100) | 2,488.2(2,374.96-2,624.58) | 4,689,954(4,474,035-4,939,246) | 2,628.43(2,507.43-2,768.15) | 0.18 (0.17 to 0.18) | 0.08(0.08 to 0.09) | 0.25(0.24 to 0.26) | 0.19(0.18 to 0.2) |
| High-income Asia Pacific | 1,144,797(1,084,808-1,203,895) | 3,252.31(3,081.88-3,420.2) | 746,462(709,917-786,765) | 3,328.62(3,165.67-3,508.34) | 0.08 (0.05 to 0.11) | 0.1(0.06 to 0.13) | -0.13(-0.21 to -0.06) | 0.25(0.21 to 0.3) |
| High-income North America | 2,052,497(1,989,102-2,114,036) | 3,327.78(3,225-3,427.56) | 2,205,004(2,132,290-2,273,809) | 3,360.32(3,249.5-3,465.17) | 0.01 (-0.02 to 0.05) | -0.03(-0.08 to 0.02) | -0.12(-0.15 to -0.1) | 0.19(0.12 to 0.27) |
| North Africa and Middle East | 2,663,145(2,525,098-2,833,995) | 1,895.67(1,797.41-2,017.29) | 3,642,268(3,444,364-3,883,784) | 1,986.81(1,878.87-2,118.56) | 0.15 (0.13 to 0.16) | 0.03(0.02 to 0.04) | 0.19(0.17 to 0.21) | 0.21(0.17 to 0.24) |
| Oceania | 55,766(52,623-58,995) | 2,080.93(1,963.66-2,201.42) | 106,891(100,934-113,445) | 2,103.79(1,986.55-2,232.78) | 0.03 (0.02 to 0.05) | 0.12(0.11 to 0.12) | -0.14(-0.18 to -0.1) | 0.11(0.1 to 0.13) |
| South Asia | 8,889,313(8,393,576-9,383,627) | 2,051.24(1,936.85-2,165.31) | 10,111,827(9,558,861-10,677,778) | 1,994.36(1,885.34-2,105.98) | -0.08 (-0.09 to -0.07) | -0.16(-0.19 to -0.13) | -0.05(-0.06 to -0.04) | -0.05(-0.06 to -0.04) |
| Southeast Asia | 3,452,673(3,277,628-3,639,805) | 2,022.09(1,919.58-2,131.69) | 3,648,740(3,465,494-3,861,068) | 2,113.33(2,007.19-2,236.31) | 0.14 (0.13 to 0.15) | 0.23(0.2 to 0.26) | 0.12(0.1 to 0.13) | 0.09(0.07 to 0.12) |
| Southern Latin America | 469,542(444,163-495,874) | 3,145.71(2,975.68-3,322.12) | 470,683(445,395-497,084) | 3,247.09(3,072.64-3,429.22) | 0.11 (0.1 to 0.12) | 0.08(0.06 to 0.09) | -0.12(-0.13 to -0.1) | 0.37(0.34 to 0.39) |
| Southern Sub-Saharan Africa | 433,536(410,938-459,085) | 2,095.46(1,986.23-2,218.95) | 524,133(497,687-554,848) | 2,177.91(2,068.03-2,305.54) | 0.13 (0.11 to 0.14) | 0.04(0.02 to 0.07) | 0.18(0.17 to 0.19) | 0.14(0.1 to 0.19) |
| Tropical Latin America | 1,007,683(956,011-1,062,795) | 1,879.52(1,783.14-1,982.31) | 976,793(926,156-1,038,886) | 1,946.07(1,845.2-2,069.78) | 0.11 (0.1 to 0.13) | -0.03(-0.05 to -0.01) | 0.01(-0.01 to 0.04) | 0.33(0.3 to 0.36) |
| Western Europe | 1,834,437(1,744,181-1,933,036) | 2,583.05(2,455.96-2,721.88) | 1,764,848(1,676,067-1,862,638) | 2,590.86(2,460.52-2,734.41) | 0.01 (-0.01 to 0.03) | -0.02(-0.05 to 0) | -0.11(-0.13 to -0.09) | 0.17(0.11 to 0.23) |
| Western Sub-Saharan Africa | 1,625,119(1,546,132-1,716,001) | 1,849.25(1,759.37-1,952.67) | 4,267,908(4,063,441-4,522,290) | 1,987.26(1,892.06-2,105.7) | 0.23 (0.23 to 0.24) | 0.07(0.06 to 0.08) | 0.33(0.32 to 0.35) | 0.27(0.26 to 0.28) |
| **204 countries and territories** |  |  |  |  |  |  |  |  |
| China | 7,109,157(6,744,577-7,487,779) | 2,232.94(2,118.43-2,351.86) | 6,394,175(6,060,506-6,780,201) | 2,462.79(2,334.27-2,611.47) | 0.31(0.27 to 0.35) | -0.17(-0.22 to -0.11) | 0.71(0.65 to 0.77) | 0.39(0.33 to 0.45) |
| Democratic People's Republic of Korea | 136,056(128,965-144,731) | 2,286.95(2,167.75-2,432.76) | 110,016(104,458-116,287) | 2,304.62(2,188.19-2,436) | 0.02(0 to 0.05) | 0.14(0.12 to 0.17) | -0.08(-0.14 to -0.02) | 0.01(-0.02 to 0.04) |
| Taiwan (Province of China) | 137,326(129,422-145,377) | 2,493.14(2,349.66-2,639.33) | 79,867(75,337-84,982) | 2,710.36(2,556.63-2,883.94) | 0.27(0.23 to 0.31) | 0.62(0.54 to 0.71) | -0.21(-0.26 to -0.17) | 0.46(0.39 to 0.54) |
| Cambodia | 88,993(83,952-94,127) | 1,909.33(1,801.18-2,019.48) | 102,965(97,229-109,250) | 2,012.39(1,900.28-2,135.22) | 0.17(0.15 to 0.18) | 0.03(0.02 to 0.05) | 0.19(0.14 to 0.23) | 0.25(0.22 to 0.28) |
| Indonesia | 1,369,937(1,299,690-1,447,956) | 2,022.43(1,918.72-2,137.61) | 1,430,546(1,356,511-1,516,870) | 2,125.99(2,015.98-2,254.28) | 0.16(0.15 to 0.17) | 0.29(0.27 to 0.3) | 0.16(0.15 to 0.18) | 0.05(0.02 to 0.07) |
| Lao People's Democratic Republic | 34,969(32,986-37,022) | 1,897.3(1,789.67-2,008.67) | 46,776(43,951-49,581) | 2,036.95(1,913.97-2,159.13) | 0.23(0.22 to 0.24) | 0.2(0.19 to 0.2) | 0.19(0.18 to 0.21) | 0.3(0.29 to 0.31) |
| Malaysia | 140,930(132,928-148,829) | 2,144.07(2,022.33-2,264.25) | 167,757(158,299-177,896) | 2,203.38(2,079.15-2,336.54) | 0.09(0.07 to 0.1) | 0.24(0.23 to 0.26) | -0.03(-0.06 to 0) | 0.03(0.01 to 0.05) |
| Maldives | 2,084(1,972-2,203) | 1,983.61(1,877.27-2,097.3) | 2,133(2,004-2,270) | 2,129.73(2,001.12-2,265.93) | 0.22(0.19 to 0.26) | 0.19(0.17 to 0.22) | 0.19(0.11 to 0.26) | 0.26(0.19 to 0.33) |
| Myanmar | 279,650(264,488-296,527) | 1,892.55(1,789.95-2,006.77) | 317,930(299,655-337,709) | 2,036.09(1,919.06-2,162.76) | 0.23(0.22 to 0.25) | 0.15(0.11 to 0.19) | 0.37(0.36 to 0.38) | 0.18(0.16 to 0.21) |
| Philippines | 521,136(495,229-550,818) | 2,066.82(1,964.08-2,184.54) | 710,194(674,500-750,442) | 2,088.9(1,983.92-2,207.28) | 0.04(0.03 to 0.05) | 0.15(0.14 to 0.16) | -0.09(-0.1 to -0.07) | 0.04(0.02 to 0.07) |
| Sri Lanka | 115,665(109,077-123,142) | 2,090.43(1,971.37-2,225.58) | 110,658(103,851-118,036) | 2,168.07(2,034.72-2,312.62) | 0.12(0.09 to 0.14) | 0.26(0.21 to 0.31) | 0.22(0.18 to 0.26) | -0.09(-0.13 to -0.05) |
| Thailand | 344,829(325,902-364,756) | 2,045.42(1,933.16-2,163.62) | 206,853(195,114-219,639) | 2,118.03(1,997.84-2,248.95) | 0.11(0.09 to 0.13) | 0.43(0.39 to 0.48) | -0.21(-0.24 to -0.18) | 0.07(0.04 to 0.09) |
| Timor-Leste | 6,374(6,019-6,709) | 1,916.13(1,809.47-2,016.85) | 10,585(9,977-11,235) | 2,033.07(1,916.38-2,157.9) | 0.18(0.17 to 0.2) | 0.29(0.28 to 0.31) | 0.43(0.41 to 0.46) | -0.09(-0.12 to -0.05) |
| Viet Nam | 535,783(505,089-567,990) | 2,020.87(1,905.1-2,142.35) | 532,273(501,064-563,587) | 2,149.5(2,023.48-2,275.96) | 0.19(0.16 to 0.23) | 0.04(0.01 to 0.06) | 0.22(0.14 to 0.31) | 0.31(0.26 to 0.35) |
| Fiji | 6,287(5,920-6,687) | 2,233.73(2,103.52-2,376.16) | 6,184(5,803-6,582) | 2,269.11(2,129.41-2,415.24) | 0.05(0.03 to 0.07) | 0.02(-0.01 to 0.04) | 0.02(0 to 0.04) | 0.07(0.04 to 0.1) |
| Kiribati | 637(597-676) | 2,155.47(2,020.59-2,288.19) | 905(855-962) | 2,153.89(2,034.04-2,289.39) | -0.01(-0.02 to 0.01) | -0.02(-0.04 to -0.01) | -0.01(-0.02 to 0) | 0.01(-0.02 to 0.04) |
| Marshall Islands | 475(449-505) | 2,165.51(2,046.54-2,300.76) | 376(354-400) | 2,154.98(2,025.66-2,290.45) | -0.02(-0.03 to 0) | -0.1(-0.15 to -0.05) | 0.14(0.12 to 0.17) | -0.1(-0.11 to -0.09) |
| Micronesia (Federated States of) | 997(939-1,055) | 2,171.65(2,044.05-2,298) | 663(626-705) | 2,167.29(2,046.55-2,302.5) | -0.01(-0.02 to 0.01) | 0.04(0 to 0.08) | 0(-0.02 to 0.03) | -0.06(-0.07 to -0.05) |
| Papua New Guinea | 34,577(32,572-36,585) | 2,033.84(1,915.95-2,151.95) | 81,583(76,868-86,919) | 2,082.76(1,962.39-2,218.99) | 0.08(0.06 to 0.09) | 0.17(0.17 to 0.18) | -0.14(-0.18 to -0.11) | 0.18(0.17 to 0.2) |
| Samoa | 1,579(1,490-1,682) | 2,215.91(2,091.16-2,361.18) | 1,808(1,703-1,925) | 2,262.07(2,130.68-2,407.93) | 0.07(0.06 to 0.08) | 0.29(0.28 to 0.3) | -0.02(-0.03 to -0.01) | -0.03(-0.05 to -0.01) |
| Solomon Islands | 3,130(2,953-3,314) | 2,010.24(1,896.75-2,128.11) | 5,390(5,074-5,738) | 2,072.64(1,951.36-2,206.64) | 0.1(0.09 to 0.11) | 0.19(0.18 to 0.21) | 0.1(0.09 to 0.11) | 0.01(-0.01 to 0.02) |
| Tonga | 924(872-982) | 2,209.59(2,086.03-2,349.1) | 886(836-944) | 2,270.18(2,141.38-2,418.94) | 0.08(0.08 to 0.09) | 0.16(0.15 to 0.17) | 0.04(0.03 to 0.05) | 0.07(0.05 to 0.08) |
| Vanuatu | 1,431(1,340-1,518) | 2,101.6(1,968.58-2,229.35) | 2,500(2,357-2,658) | 2,144.75(2,021.99-2,280.59) | 0.06(0.05 to 0.07) | 0.12(0.09 to 0.14) | 0.05(0.04 to 0.06) | 0.02(0 to 0.04) |
| Armenia | 21,163(19,876-22,444) | 2,028.69(1,905.3-2,151.49) | 12,025(11,274-12,750) | 2,030.05(1,903.28-2,152.38) | 0(-0.05 to 0.05) | -0.49(-0.55 to -0.44) | 0.22(0.1 to 0.35) | 0.2(0.12 to 0.28) |
| Azerbaijan | 50,093(47,050-53,227) | 2,064.06(1,938.67-2,193.17) | 47,859(44,982-50,875) | 2,027.41(1,905.54-2,155.2) | -0.06(-0.11 to -0.02) | -0.36(-0.4 to -0.32) | -0.22(-0.32 to -0.12) | 0.29(0.2 to 0.38) |
| Georgia | 29,017(27,227-30,844) | 2,120.02(1,989.23-2,253.53) | 15,272(14,386-16,217) | 2,075.14(1,954.86-2,203.61) | -0.07(-0.1 to -0.04) | -0.56(-0.6 to -0.52) | -0.03(-0.06 to 0) | 0.31(0.25 to 0.37) |
| Kazakhstan | 107,813(100,865-114,707) | 2,074.83(1,941.11-2,207.5) | 112,238(105,631-119,398) | 2,068.33(1,946.6-2,200.29) | -0.02(-0.06 to 0.02) | -0.35(-0.39 to -0.31) | 0.09(-0.01 to 0.18) | 0.14(0.08 to 0.2) |
| Kyrgyzstan | 34,280(32,301-36,461) | 2,043.45(1,925.44-2,173.43) | 45,604(42,928-48,590) | 2,005.12(1,887.45-2,136.41) | -0.07(-0.11 to -0.03) | -0.32(-0.36 to -0.29) | -0.4(-0.46 to -0.34) | 0.4(0.31 to 0.49) |
| Mongolia | 17,574(16,553-18,649) | 1,952.67(1,839.16-2,072.14) | 22,019(20,720-23,370) | 2,026.44(1,906.86-2,150.71) | 0.13(0.09 to 0.17) | -0.21(-0.31 to -0.11) | -0.06(-0.09 to -0.02) | 0.64(0.6 to 0.69) |
| Tajikistan | 46,194(43,352-49,023) | 1,989.37(1,866.98-2,111.2) | 69,799(65,652-74,201) | 1,947.31(1,831.64-2,070.14) | -0.07(-0.1 to -0.05) | -0.47(-0.51 to -0.42) | -0.17(-0.22 to -0.11) | 0.4(0.35 to 0.44) |
| Turkmenistan | 30,780(28,893-32,744) | 2,050.82(1,925.12-2,181.69) | 30,994(29,176-32,980) | 2,033.71(1,914.4-2,164.07) | -0.03(-0.04 to -0.02) | -0.21(-0.23 to -0.2) | -0.11(-0.14 to -0.08) | 0.22(0.2 to 0.23) |
| Uzbekistan | 171,730(162,096-182,621) | 2,007.21(1,894.61-2,134.51) | 204,501(192,535-216,368) | 2,026.55(1,907.98-2,144.15) | 0.03(0.01 to 0.05) | -0.17(-0.21 to -0.13) | -0.23(-0.28 to -0.17) | 0.39(0.37 to 0.41) |
| Albania | 22,894(21,556-24,303) | 2,049.12(1,929.34-2,175.24) | 9,040(8,527-9,559) | 2,037.43(1,921.78-2,154.55) | -0.02(-0.04 to 0.01) | -0.14(-0.16 to -0.13) | -0.44(-0.49 to -0.39) | 0.44(0.4 to 0.48) |
| Bosnia and Herzegovina | 21,750(20,508-23,146) | 1,985.23(1,871.83-2,112.63) | 10,130(9,569-10,748) | 2,065.37(1,951.18-2,191.37) | 0.13(0.09 to 0.17) | 0.13(0.06 to 0.19) | -0.2(-0.29 to -0.11) | 0.32(0.27 to 0.36) |
| Bulgaria | 35,714(33,520-37,861) | 2,057.02(1,930.61-2,180.67) | 20,355(19,140-21,610) | 2,085.41(1,960.93-2,213.98) | 0.03(-0.03 to 0.09) | -0.32(-0.41 to -0.23) | 0.64(0.48 to 0.81) | -0.1(-0.16 to -0.05) |
| Croatia | 20,878(19,588-22,285) | 2,115.24(1,984.57-2,257.83) | 12,538(11,825-13,291) | 2,099.63(1,980.32-2,225.76) | -0.03(-0.07 to 0.01) | -0.12(-0.16 to -0.07) | -0.29(-0.39 to -0.18) | 0.12(0.08 to 0.16) |
| Czechia | 45,329(42,518-48,027) | 2,056.97(1,929.38-2,179.39) | 36,734(34,633-38,991) | 2,140.27(2,017.82-2,271.73) | 0.12(0.05 to 0.18) | 0.39(0.31 to 0.46) | 0.3(0.25 to 0.35) | -0.27(-0.44 to -0.11) |
| Hungary | 42,839(40,383-45,556) | 2,010.21(1,894.96-2,137.72) | 29,412(27,548-31,173) | 2,118.23(1,983.98-2,245.09) | 0.16(0.1 to 0.22) | 0.63(0.45 to 0.8) | -0.01(-0.08 to 0.05) | -0.05(-0.09 to -0.01) |
| North Macedonia | 10,914(10,283-11,594) | 2,071.85(1,952.06-2,200.89) | 6,855(6,455-7,271) | 2,092.71(1,970.42-2,219.45) | 0.03(0.01 to 0.06) | -0.11(-0.17 to -0.06) | -0.18(-0.2 to -0.17) | 0.34(0.29 to 0.39) |
| Montenegro | 3,460(3,250-3,673) | 2,141.02(2,011.3-2,272.81) | 2,354(2,214-2,509) | 2,112.43(1,986.95-2,251.76) | -0.04(-0.06 to -0.02) | -0.29(-0.31 to -0.27) | -0.04(-0.07 to 0) | 0.14(0.1 to 0.17) |
| Poland | 197,186(187,198-208,741) | 2,059(1,954.7-2,179.65) | 122,666(116,300-130,000) | 2,084.12(1,975.98-2,208.73) | 0.04(0.01 to 0.07) | -0.25(-0.34 to -0.16) | 0.06(0.03 to 0.09) | 0.18(0.15 to 0.21) |
| Romania | 116,765(110,320-123,725) | 2,097.05(1,981.3-2,222.05) | 63,032(59,469-66,948) | 2,094.08(1,975.7-2,224.17) | 0(-0.04 to 0.03) | -0.37(-0.44 to -0.3) | 0.64(0.57 to 0.71) | -0.15(-0.18 to -0.11) |
| Serbia | 44,899(41,946-47,627) | 2,070.17(1,934.01-2,195.98) | 27,018(25,309-28,725) | 2,034.74(1,906.02-2,163.31) | -0.07(-0.13 to -0.01) | 0.05(-0.07 to 0.17) | 0.14(0.01 to 0.28) | -0.38(-0.43 to -0.34) |
| Slovakia | 27,710(26,143-29,296) | 2,090.22(1,972.06-2,209.87) | 18,196(17,164-19,360) | 2,124.37(2,003.89-2,260.19) | 0.06(0.02 to 0.1) | 0.05(0 to 0.09) | 0(-0.11 to 0.11) | 0.07(0.03 to 0.11) |
| Slovenia | 8,890(8,385-9,451) | 2,149.82(2,027.76-2,285.41) | 6,766(6,363-7,188) | 2,166.72(2,037.78-2,301.97) | 0.04(-0.01 to 0.08) | -0.12(-0.14 to -0.09) | 0.09(0 to 0.19) | 0.06(-0.05 to 0.16) |
| Belarus | 50,615(47,669-53,651) | 2,105.58(1,983-2,231.85) | 33,446(31,329-35,624) | 2,119.26(1,985.15-2,257.28) | 0.02(-0.02 to 0.06) | -0.77(-0.83 to -0.71) | 0.55(0.44 to 0.66) | 0.26(0.21 to 0.32) |
| Estonia | 7,390(6,942-7,867) | 2,116.77(1,988.4-2,253.49) | 4,580(4,302-4,861) | 2,119.14(1,990.5-2,249.11) | -0.02(-0.1 to 0.06) | -0.45(-0.53 to -0.36) | 0.59(0.42 to 0.77) | -0.18(-0.33 to -0.03) |
| Latvia | 12,216(11,463-13,008) | 2,147.04(2,014.7-2,286.37) | 6,375(5,998-6,771) | 2,145.96(2,019.08-2,279.42) | -0.02(-0.08 to 0.05) | -0.64(-0.71 to -0.56) | 0.73(0.57 to 0.89) | -0.17(-0.25 to -0.09) |
| Lithuania | 17,542(16,410-18,607) | 2,111.68(1,975.41-2,239.96) | 8,900(8,364-9,427) | 2,182.38(2,051.11-2,311.78) | 0.1(0.07 to 0.14) | -0.25(-0.32 to -0.18) | -0.11(-0.19 to -0.02) | 0.56(0.52 to 0.59) |
| Republic of Moldova | 25,423(23,951-26,973) | 2,057.05(1,937.92-2,182.4) | 10,554(9,909-11,184) | 2,020.59(1,897.08-2,141.31) | -0.06(-0.09 to -0.03) | -0.67(-0.72 to -0.61) | 0.17(0.11 to 0.22) | 0.23(0.17 to 0.29) |
| Russian Federation | 740,813(702,189-784,128) | 2,135(2,023.69-2,259.83) | 552,157(522,375-584,215) | 2,117.38(2,003.18-2,240.31) | -0.03(-0.11 to 0.04) | -1.03(-1.17 to -0.89) | 0.88(0.79 to 0.97) | 0.09(0 to 0.18) |
| Ukraine | 239,308(225,341-253,402) | 2,103.82(1,981.04-2,227.73) | 127,794(120,322-135,848) | 2,014.03(1,896.27-2,140.95) | -0.14(-0.21 to -0.07) | -0.7(-0.82 to -0.58) | 0.32(0.23 to 0.42) | -0.12(-0.28 to 0.03) |
| Brunei Darussalam | 2,925(2,765-3,092) | 3,228.71(3,052.96-3,413.29) | 3,092(2,911-3,263) | 3,268.42(3,077.46-3,449.61) | 0.04(0.02 to 0.06) | 0.07(0.06 to 0.08) | -0.07(-0.09 to -0.06) | 0.13(0.08 to 0.19) |
| Japan | 756,667(717,324-799,370) | 3,277.17(3,106.77-3,462.12) | 512,433(485,894-541,538) | 3,317.93(3,146.1-3,506.38) | 0.04(0.02 to 0.06) | -0.04(-0.06 to -0.02) | 0(-0.02 to 0.03) | 0.14(0.1 to 0.18) |
| Republic of Korea | 364,819(344,652-386,165) | 3,208.45(3,031.09-3,396.18) | 203,332(192,555-215,297) | 3,347.31(3,169.91-3,544.28) | 0.14(0.08 to 0.2) | 0.34(0.24 to 0.44) | -0.38(-0.49 to -0.28) | 0.47(0.4 to 0.55) |
| Singapore | 20,387(19,287-21,556) | 3,139.45(2,970.16-3,319.54) | 27,604(26,172-29,131) | 3,399.18(3,222.83-3,587.1) | 0.27(0.21 to 0.33) | 0.56(0.45 to 0.67) | -0.2(-0.32 to -0.09) | 0.57(0.5 to 0.64) |
| Australia | 123,036(116,558-129,239) | 3,249.9(3,078.79-3,413.75) | 156,698(148,129-165,388) | 3,299.31(3,118.88-3,482.27) | 0.06(0.03 to 0.08) | 0.05(0.04 to 0.07) | -0.12(-0.15 to -0.09) | 0.19(0.14 to 0.24) |
| New Zealand | 25,889(24,514-27,311) | 3,235.65(3,063.74-3,413.38) | 32,467(30,683-34,276) | 3,307.14(3,125.41-3,491.44) | 0.07(0.05 to 0.09) | 0.14(0.12 to 0.16) | -0.12(-0.16 to -0.08) | 0.19(0.15 to 0.23) |
| Andorra | 214(203-227) | 2,255.55(2,132.28-2,389.9) | 225(212-238) | 2,208.41(2,086.19-2,344.22) | -0.06(-0.21 to 0.09) | 0.52(0.4 to 0.64) | -0.09(-0.27 to 0.09) | -0.48(-0.82 to -0.13) |
| Austria | 30,782(29,030-32,643) | 2,282.96(2,152.97-2,420.95) | 29,856(28,275-31,580) | 2,301.85(2,179.98-2,434.79) | 0.03(-0.01 to 0.07) | 0.05(-0.03 to 0.13) | -0.21(-0.27 to -0.16) | 0.27(0.2 to 0.33) |
| Belgium | 40,532(38,323-42,884) | 2,244.14(2,121.81-2,374.35) | 43,645(41,151-46,330) | 2,282.56(2,152.12-2,422.99) | 0.06(0.03 to 0.1) | 0.11(0.07 to 0.14) | 0.01(-0.02 to 0.03) | 0.09(0.01 to 0.18) |
| Cyprus | 4,330(4,104-4,582) | 2,187.38(2,073.26-2,314.78) | 5,033(4,761-5,331) | 2,301.38(2,177.03-2,437.82) | 0.17(0.14 to 0.19) | 0.15(0.1 to 0.2) | 0.01(-0.02 to 0.03) | 0.32(0.27 to 0.37) |
| Denmark | 21,374(20,233-22,610) | 2,419.69(2,290.51-2,559.58) | 23,721(22,351-25,226) | 2,486.05(2,342.46-2,643.82) | 0.09(0.05 to 0.14) | 0.79(0.67 to 0.9) | -0.4(-0.44 to -0.36) | -0.01(-0.08 to 0.05) |
| Finland | 24,617(23,258-26,109) | 2,551.13(2,410.33-2,705.8) | 21,375(20,203-22,685) | 2,523.32(2,385.00-2,677.94) | -0.04(-0.07 to 0) | 0.07(-0.03 to 0.18) | -0.06(-0.1 to -0.01) | -0.11(-0.15 to -0.08) |
| France | 266,103(251,481-281,100) | 2,271.62(2,146.81-2,399.65) | 261,754(247,610-277,423) | 2,255.01(2,133.17-2,390) | -0.02(-0.05 to 0.01) | -0.21(-0.23 to -0.18) | 0.16(0.08 to 0.24) | -0.01(-0.04 to 0.03) |
| Germany | 549,385(517,452-586,119) | 4,243.72(3,997.05-4,527.47) | 509,360(478,395-547,214) | 4,257.22(3,998.43-4,573.6) | 0.01(-0.02 to 0.04) | -0.38(-0.44 to -0.32) | 0.11(0.05 to 0.16) | 0.31(0.27 to 0.36) |
| Greece | 43,625(41,105-46,034) | 2,155.83(2,031.31-2,274.86) | 30,480(28,807-32,338) | 2,185.33(2,065.39-2,318.55) | 0.05(0.02 to 0.08) | 0.07(0 to 0.14) | 0.26(0.23 to 0.29) | -0.18(-0.24 to -0.12) |
| Iceland | 1,440(1,364-1,521) | 2,269.22(2,149.79-2,397.8) | 1,531(1,446-1,618) | 2,267.44(2,141.26-2,395.65) | -0.01(-0.1 to 0.07) | 0.27(0.08 to 0.45) | -0.23(-0.38 to -0.08) | -0.06(-0.14 to 0.03) |
| Ireland | 21,824(20,578-23,184) | 2,221.22(2,094.41-2,359.65) | 22,782(21,468-24,163) | 2,284.63(2,152.86-2,423.09) | 0.09(0.01 to 0.16) | -0.1(-0.24 to 0.04) | 0.37(0.21 to 0.53) | -0.07(-0.15 to 0.01) |
| Israel | 24,305(22,916-25,892) | 1,585.4(1,494.78-1,688.89) | 42,652(39,669-45,449) | 1,623.14(1,509.62-1,729.59) | 0.07(0.05 to 0.1) | 0(-0.02 to 0.03) | 0.02(-0.03 to 0.07) | 0.21(0.16 to 0.26) |
| Italy | 197,724(187,663-208,236) | 2,142.25(2,033.24-2,256.14) | 167,905(159,192-177,075) | 2,209.31(2,094.68-2,329.97) | 0.1(0.07 to 0.13) | 0.35(0.32 to 0.38) | 0.13(0.08 to 0.18) | -0.11(-0.16 to -0.06) |
| Luxembourg | 1,519(1,435-1,608) | 2,298.47(2,172.42-2,433.44) | 2,341(2,205-2,476) | 2,312.94(2,178.58-2,446.06) | 0.01(-0.02 to 0.04) | 0.13(0.09 to 0.17) | -0.25(-0.31 to -0.2) | 0.16(0.11 to 0.2) |
| Malta | 1,915(1,810-2,021) | 2,187.62(2,068.52-2,309.28) | 1,456(1,372-1,547) | 2,273.82(2,142.85-2,416.48) | 0.12(0.1 to 0.15) | -0.03(-0.04 to -0.01) | -0.22(-0.25 to -0.18) | 0.53(0.48 to 0.57) |
| Netherlands | 54,974(52,018-58,373) | 2,017.21(1,908.74-2,141.92) | 53,723(50,760-57,095) | 2,003.07(1,892.62-2,128.81) | -0.01(-0.04 to 0.01) | 0.06(0.02 to 0.09) | 0(-0.04 to 0.03) | -0.02(-0.08 to 0.05) |
| Norway | 17,588(16,633-18,622) | 2,203.11(2,083.4-2,332.58) | 20,571(19,520-21,757) | 2,226.81(2,113.08-2,355.14) | 0.04(0 to 0.07) | 0.32(0.26 to 0.39) | -0.31(-0.33 to -0.29) | 0.09(0.03 to 0.16) |
| Portugal | 43,379(41,052-45,839) | 2,050.13(1,940.16-2,166.41) | 29,415(27,776-31,140) | 2,159.29(2,039.00-2,285.92) | 0.17(0.14 to 0.19) | 0.36(0.32 to 0.39) | 0.19(0.17 to 0.21) | 0.01(-0.03 to 0.06) |
| Spain | 183,511(173,142-193,629) | 2,341.89(2,209.57-2,471.01) | 159,297(149,808-168,569) | 2,457.99(2,311.59-2,601.06) | 0.16(0.14 to 0.18) | 0.35(0.3 to 0.4) | 0.41(0.4 to 0.43) | -0.24(-0.29 to -0.2) |
| Sweden | 38,679(36,621-40,881) | 2,504.41(2,371.15-2,646.99) | 46,038(43,817-48,688) | 2,528.54(2,406.57-2,674.09) | 0.03(-0.01 to 0.06) | 0.04(-0.01 to 0.09) | 0.14(0.1 to 0.18) | -0.03(-0.12 to 0.05) |
| Switzerland | 27,397(25,810-29,024) | 2,370.7(2,233.34-2,511.53) | 31,641(29,781-33,726) | 2,374.55(2,234.98-2,531.06) | 0.01(-0.01 to 0.03) | -0.11(-0.13 to -0.09) | -0.2(-0.23 to -0.17) | 0.28(0.24 to 0.33) |
| United Kingdom | 237,540(225,718-250,451) | 2,175.19(2,066.93-2,293.41) | 258,279(245,354-272,844) | 2,191.95(2,082.26-2,315.57) | 0.03(0.01 to 0.04) | -0.06(-0.08 to -0.05) | -0.13(-0.14 to -0.11) | 0.21(0.18 to 0.23) |
| Argentina | 319,404(301,414-336,919) | 3,151.43(2,973.92-3,324.23) | 330,547(312,631-349,087) | 3,245.95(3,070.03-3,428.02) | 0.1(0.04 to 0.16) | 0.06(0.01 to 0.11) | -0.1(-0.19 to -0.02) | 0.36(0.23 to 0.48) |
| Chile | 124,572(118,062-132,108) | 3,136.33(2,972.43-3,326.06) | 118,856(112,263-125,766) | 3,254.67(3,074.15-3,443.91) | 0.12(0.09 to 0.14) | 0.15(0.12 to 0.18) | -0.21(-0.26 to -0.16) | 0.37(0.35 to 0.39) |
| Uruguay | 25,542(24,231-27,006) | 3,120.35(2,960.09-3,299.13) | 21,255(20,080-22,475) | 3,222.81(3,044.67-3,407.76) | 0.1(0.05 to 0.15) | 0.07(0.01 to 0.13) | -0.11(-0.13 to -0.08) | 0.32(0.2 to 0.44) |
| Canada | 191,587(182,412-202,114) | 3,330.96(3,171.45-3,513.98) | 208,774(197,796-220,269) | 3,382.7(3,204.83-3,568.95) | 0.04(-0.01 to 0.08) | 0.13(0.05 to 0.21) | -0.22(-0.32 to -0.12) | 0.19(0.16 to 0.23) |
| United States of America | 1,860,389(1,801,934-1,918,464) | 3,327.45(3,222.90-3,431.32) | 1,995,798(1,931,211-2,058,694) | 3,357.99(3,249.33-3,463.81) | 0.01(-0.03 to 0.05) | -0.04(-0.1 to 0.01) | -0.1(-0.13 to -0.08) | 0.19(0.11 to 0.27) |
| Antigua and Barbuda | 365(344-390) | 2,004.29(1,890.79-2,145.12) | 339(320-362) | 2,007.38(1,890.43-2,143.72) | 0(-0.02 to 0.01) | 0.15(0.13 to 0.16) | -0.13(-0.16 to -0.09) | -0.02(-0.04 to 0) |
| Bahamas | 1,676(1,564-1,790) | 2,077.87(1,938.77-2,219.59) | 1,612(1,516-1,718) | 1,985.91(1,867.50-2,115.78) | -0.16(-0.22 to -0.11) | 0.18(0.02 to 0.33) | 0.01(-0.07 to 0.08) | -0.59(-0.63 to -0.56) |
| Barbados | 1,255(1,184-1,338) | 2,011.74(1,899.28-2,145.86) | 931(878-991) | 1,976.82(1,864.04-2,104.3) | -0.06(-0.1 to -0.02) | 0.18(0.14 to 0.22) | 0.15(0.04 to 0.26) | -0.37(-0.41 to -0.32) |
| Belize | 1,544(1,451-1,647) | 1,885.76(1,772.31-2,011.39) | 2,345(2,208-2,489) | 1,904.38(1,793.56-2,021.69) | 0.03(0 to 0.06) | 0.14(0.11 to 0.17) | 0.18(0.15 to 0.22) | -0.15(-0.19 to -0.11) |
| Cuba | 49,825(46,688-53,171) | 1,989.8(1,864.53-2,123.42) | 35,232(33,119-37,694) | 1,982.64(1,863.76-2,121.18) | -0.02(-0.08 to 0.04) | -0.4(-0.51 to -0.3) | 0.15(0.06 to 0.24) | 0.32(0.23 to 0.42) |
| Dominica | 486(456-517) | 1,958.59(1,837.75-2,084.59) | 268(253-287) | 1,961.58(1,847.84-2,095.72) | 0(-0.04 to 0.04) | 0.3(0.21 to 0.39) | 0.03(-0.05 to 0.1) | -0.23(-0.29 to -0.17) |
| Dominican Republic | 49,931(46,919-52,954) | 1,852.4(1,740.67-1,964.58) | 57,006(53,417-60,552) | 1,940(1,817.85-2,060.69) | 0.15(0.14 to 0.16) | 0.21(0.2 to 0.21) | 0.03(0.02 to 0.04) | 0.23(0.21 to 0.26) |
| Grenada | 640(600-682) | 1,915.55(1,795.4-2,041.01) | 427(401-456) | 1,955.47(1,839.19-2,089.55) | 0.06(0.05 to 0.08) | 0.09(0.06 to 0.13) | 0.09(0.06 to 0.11) | 0.01(-0.02 to 0.04) |
| Guyana | 5,461(5,126-5,824) | 1,857.82(1,744.01-1,981.43) | 4,207(3,948-4,491) | 1,971.62(1,850.34-2,104.6) | 0.21(0.14 to 0.27) | 0.7(0.58 to 0.82) | -0.56(-0.66 to -0.45) | 0.49(0.45 to 0.53) |
| Haiti | 47,907(45,085-50,810) | 1,765.77(1,661.75-1,872.78) | 78,859(74,372-84,164) | 1,811.69(1,708.6-1,933.58) | 0.08(0.05 to 0.11) | -0.04(-0.1 to 0.01) | 0.25(0.22 to 0.28) | 0.06(0.03 to 0.09) |
| Jamaica | 16,308(15,361-17,461) | 1,952.56(1,839.18-2,090.66) | 11,376(10,660-12,109) | 1,948.22(1,825.65-2,073.72) | -0.01(-0.04 to 0.02) | 0.25(0.23 to 0.27) | -0.33(-0.4 to -0.26) | 0.07(0.04 to 0.1) |
| Saint Lucia | 987(929-1,054) | 1,915.61(1,802.25-2,043.93) | 578(542-614) | 1,946.02(1,825.92-2,068.24) | 0.05(0.03 to 0.06) | 0.22(0.2 to 0.25) | -0.18(-0.19 to -0.16) | 0.1(0.07 to 0.13) |
| Saint Vincent and the Grenadines | 772(725-821) | 1,879.3(1,764.3-1,999.35) | 483(456-514) | 1,936.89(1,827.78-2,058.95) | 0.1(0.06 to 0.13) | 0.22(0.17 to 0.27) | -0.11(-0.14 to -0.07) | 0.13(0.05 to 0.22) |
| Suriname | 2,524(2,377-2,675) | 1,937.73(1,824.34-2,053.59) | 2,774(2,605-2,960) | 1,936.44(1,818.12-2,066.03) | 0(-0.02 to 0.01) | -0.15(-0.17 to -0.12) | 0.01(-0.02 to 0.04) | 0.09(0.06 to 0.12) |
| Trinidad and Tobago | 8,437(7,914-8,983) | 2,076.41(1,947.72-2,210.89) | 5,604(5,253-6,002) | 2,057.14(1,928.33-2,203.31) | -0.04(-0.09 to 0.01) | -0.7(-0.75 to -0.66) | 0.62(0.57 to 0.66) | -0.1(-0.21 to 0.01) |
| Bolivia (Plurinational State of) | 51,689(48,595-55,343) | 1,924.48(1,809.31-2,060.54) | 69,342(64,997-73,874) | 1,988.86(1,864.26-2,118.86) | 0.1(0.08 to 0.12) | 0.17(0.14 to 0.19) | -0.07(-0.09 to -0.05) | 0.22(0.18 to 0.26) |
| Ecuador | 76,781(72,190-81,970) | 1,986.42(1,867.64-2,120.66) | 103,180(96,749-110,171) | 2,034.75(1,907.93-2,172.62) | 0.08(0.05 to 0.1) | 0.2(0.13 to 0.27) | -0.1(-0.12 to -0.07) | 0.14(0.11 to 0.18) |
| Peru | 165,409(155,892-176,220) | 1,992.66(1,878.02-2,122.9) | 196,734(185,058-209,740) | 2,062.76(1,940.34-2,199.14) | 0.11(0.1 to 0.12) | 0.07(0.05 to 0.09) | 0.01(0 to 0.03) | 0.23(0.2 to 0.26) |
| Colombia | 212,290(200,201-226,414) | 1,820.2(1,716.55-1,941.3) | 197,412(185,987-211,000) | 1,860.09(1,752.44-1,988.12) | 0.06(0.04 to 0.09) | 0.17(0.12 to 0.22) | -0.19(-0.23 to -0.15) | 0.21(0.19 to 0.23) |
| Costa Rica | 21,354(20,079-22,790) | 1,899.52(1,786.06-2,027.25) | 19,437(18,253-20,769) | 1,910.66(1,794.27-2,041.62) | 0.02(0 to 0.04) | -0.2(-0.23 to -0.17) | -0.04(-0.1 to 0.01) | 0.25(0.23 to 0.27) |
| El Salvador | 37,271(35,063-39,607) | 1,727.06(1,624.71-1,835.29) | 33,511(31,538-35,724) | 1,842.48(1,734.03-1,964.18) | 0.21(0.17 to 0.24) | 0.68(0.61 to 0.76) | -0.45(-0.51 to -0.38) | 0.35(0.3 to 0.39) |
| Guatemala | 68,944(65,061-73,595) | 1,697.6(1,602.01-1,812.13) | 88,471(83,461-94,518) | 1,793.08(1,691.54-1,915.64) | 0.18(0.14 to 0.23) | 0.38(0.33 to 0.42) | 0.12(0.04 to 0.19) | 0.12(0.05 to 0.19) |
| Honduras | 37,876(35,800-40,351) | 1,714.31(1,620.32-1,826.3) | 57,954(54,440-61,800) | 1,768.41(1,661.19-1,885.77) | 0.1(0.09 to 0.11) | 0.13(0.11 to 0.15) | 0.07(0.04 to 0.1) | 0.11(0.09 to 0.12) |
| Mexico | 602,010(571,252-640,061) | 1,801.55(1,709.50-1,915.42) | 596,081(563,689-633,252) | 1,858.86(1,757.86-1,974.78) | 0.1(0.07 to 0.12) | 0.14(0.09 to 0.18) | 0.03(0 to 0.06) | 0.13(0.09 to 0.17) |
| Nicaragua | 31,436(29,670-33,547) | 1,726.16(1,629.19-1,842.03) | 35,443(33,310-37,804) | 1,789.67(1,681.97-1,908.89) | 0.12(0.1 to 0.14) | 0.14(0.12 to 0.15) | 0.01(-0.03 to 0.04) | 0.19(0.16 to 0.22) |
| Panama | 15,687(14,804-16,788) | 1,881.1(1,775.32-2,013.23) | 22,312(20,901-23,856) | 1,934.51(1,812.22-2,068.36) | 0.09(0.07 to 0.11) | 0.09(0.06 to 0.12) | -0.11(-0.14 to -0.08) | 0.27(0.23 to 0.31) |
| Venezuela (Bolivarian Republic of) | 131,055(123,399-139,733) | 1,847.42(1,739.5-1,969.75) | 121,902(114,358-130,718) | 1,840.33(1,726.44-1,973.43) | 0(-0.05 to 0.04) | 0.23(0.14 to 0.32) | -0.18(-0.3 to -0.06) | -0.02(-0.06 to 0.03) |
| Brazil | 975,537(925,331-1,029,382) | 1,878.04(1,781.39-1,981.7) | 937,422(889,271-997,034) | 1,945.45(1,845.53-2,069.16) | 0.12(0.1 to 0.13) | -0.03(-0.05 to -0.02) | 0.01(-0.01 to 0.04) | 0.34(0.31 to 0.37) |
| Paraguay | 32,146(30,274-34,255) | 1,925.39(1,813.26-2,051.71) | 39,371(36,963-41,943) | 1,960.96(1,841.05-2,089.09) | 0.06(0.05 to 0.07) | 0.01(-0.01 to 0.03) | 0.04(0.03 to 0.05) | 0.13(0.11 to 0.14) |
| Algeria | 210,502(197,119-224,116) | 1,962.79(1,838.00-2,089.73) | 272,954(255,439-291,484) | 2,052.03(1,920.35-2,191.33) | 0.15(0.1 to 0.2) | -0.05(-0.08 to -0.03) | 0.04(-0.1 to 0.18) | 0.36(0.28 to 0.45) |
| Bahrain | 3,379(3,185-3,618) | 2,069.89(1,951.14-2,215.97) | 6,207(5,829-6,641) | 2,091.80(1,964.53-2,238.26) | 0.04(-0.02 to 0.09) | 0.18(0.05 to 0.31) | -0.05(-0.08 to -0.02) | 0(-0.11 to 0.11) |
| Egypt | 420,732(394,233-450,378) | 1,896.75(1,777.29-2,030.4) | 750,271(701,427-802,540) | 2,035.73(1,903.21-2,177.55) | 0.23(0.21 to 0.25) | 0.11(0.08 to 0.14) | 0.18(0.15 to 0.21) | 0.41(0.38 to 0.44) |
| Iran (Islamic Republic of) | 497,227(471,514-526,362) | 1,958.78(1,857.49-2,073.55) | 412,144(390,271-437,017) | 2,042.4(1,934.01-2,165.66) | 0.13(0.1 to 0.17) | -0.48(-0.5 to -0.46) | 0.45(0.39 to 0.51) | 0.33(0.27 to 0.38) |
| Iraq | 157,015(147,357-167,507) | 1,906.33(1,789.08-2,033.72) | 270,567(253,800-288,614) | 2,009.87(1,885.33-2,143.93) | 0.17(0.15 to 0.18) | 0.16(0.13 to 0.19) | 0.18(0.17 to 0.2) | 0.15(0.12 to 0.17) |
| Jordan | 32,708(30,751-35,067) | 2,002.46(1,882.67-2,146.88) | 72,956(68,751-77,973) | 2,008.12(1,892.38-2,146.21) | 0(-0.03 to 0.04) | 0.17(0.12 to 0.23) | 0.21(0.17 to 0.25) | -0.34(-0.41 to -0.27) |
| Kuwait | 12,134(11,412-12,959) | 2,188.91(2,058.61-2,337.76) | 18,282(17,048-19,486) | 2,162.25(2,016.31-2,304.68) | -0.04(-0.09 to 0) | -0.17(-0.3 to -0.05) | -0.05(-0.11 to 0) | -0.04(-0.07 to -0.01) |
| Lebanon | 20,372(19,216-21,647) | 1,947.85(1,837.29-2,069.71) | 25,959(24,364-27,649) | 2,031.12(1,906.32-2,163.39) | 0.13(0.1 to 0.16) | 0.07(0.05 to 0.09) | 0.13(0.08 to 0.19) | 0.14(0.09 to 0.19) |
| Libya | 35,123(32,891-37,390) | 1,939.5(1,816.24-2,064.66) | 29,567(27,784-31,680) | 1,982.22(1,862.68-2,123.89) | 0.06(0.04 to 0.09) | 0.47(0.39 to 0.55) | 0.27(0.24 to 0.29) | -0.48(-0.51 to -0.44) |
| Morocco | 180,980(170,022-193,653) | 1,849.42(1,737.45-1,978.92) | 188,688(177,037-201,121) | 1,926.98(1,808.02-2,053.96) | 0.13(0.12 to 0.14) | 0.07(0.06 to 0.09) | 0.15(0.14 to 0.17) | 0.16(0.14 to 0.18) |
| Palestine | 17,843(16,837-19,014) | 1,842.69(1,738.84-1,963.66) | 36,209(33,888-38,715) | 1,939.27(1,814.95-2,073.47) | 0.17(0.15 to 0.18) | 0.3(0.27 to 0.33) | 0.06(0.05 to 0.07) | 0.14(0.12 to 0.16) |
| Oman | 16,560(15,547-17,706) | 1,970.54(1,850.05-2,106.93) | 26,588(24,986-28,364) | 2,173.96(2,043-2,319.19) | 0.32(0.3 to 0.35) | 0.26(0.24 to 0.28) | 0.32(0.26 to 0.38) | 0.38(0.34 to 0.41) |
| Qatar | 2,633(2,473-2,822) | 2,106.02(1,977.51-2,256.98) | 10,900(10,286-11,673) | 2,206.83(2,082.63-2,363.51) | 0.16(0.12 to 0.2) | 0.41(0.33 to 0.5) | 0.03(-0.03 to 0.1) | 0.08(0.02 to 0.14) |
| Saudi Arabia | 132,439(124,032-141,852) | 2,020.93(1,892.64-2,164.56) | 161,719(151,681-172,723) | 2,137.65(2,004.97-2,283.1) | 0.18(0.15 to 0.2) | 0.28(0.27 to 0.29) | -0.06(-0.1 to -0.02) | 0.28(0.22 to 0.33) |
| Syrian Arab Republic | 111,393(104,974-118,883) | 1,880.96(1,772.59-2,007.45) | 67,963(63,937-72,236) | 1,855.23(1,745.35-1,971.88) | -0.05(-0.09 to -0.02) | 0.31(0.27 to 0.34) | 0.3(0.27 to 0.33) | -0.71(-0.79 to -0.62) |
| Tunisia | 59,854(56,339-63,810) | 1,927.38(1,814.19-2,054.77) | 56,186(52,601-60,032) | 2,031.52(1,901.9-2,170.58) | 0.17(0.15 to 0.18) | 0.1(0.08 to 0.12) | 0.25(0.23 to 0.26) | 0.15(0.12 to 0.18) |
| Turkey | 390,439(366,821-415,144) | 1,905.63(1,790.36-2,026.21) | 377,678(354,130-405,441) | 2,039.25(1,912.12-2,189.16) | 0.22(0.21 to 0.24) | 0.24(0.23 to 0.25) | 0.15(0.12 to 0.18) | 0.28(0.26 to 0.29) |
| United Arab Emirates | 12,842(12,032-13,787) | 2,178.82(2,041.35-2,339.18) | 30,016(28,106-31,995) | 2,242.04(2,099.34-2,389.85) | 0.1(0.02 to 0.17) | 0.19(0.05 to 0.33) | -0.05(-0.12 to 0.02) | 0.2(0.07 to 0.32) |
| Yemen | 122,137(115,195-130,770) | 1,721.6(1,623.75-1,843.3) | 253,893(239,249-272,706) | 1,841.33(1,735.13-1,977.77) | 0.22(0.2 to 0.23) | 0.18(0.17 to 0.19) | 0.62(0.6 to 0.63) | -0.1(-0.14 to -0.06) |
| Afghanistan | 71,447(67,521-75,874) | 1,658.44(1,567.33-1,761.21) | 253,365(237,904-269,630) | 1,784.18(1,675.3-1,898.72) | 0.22(0.18 to 0.26) | 0.52(0.47 to 0.57) | -0.1(-0.21 to 0) | 0.28(0.25 to 0.31) |
| Bangladesh | 995,911(937,638-1,058,926) | 2,036.15(1,917.01-2,164.99) | 907,369(853,252-966,405) | 1,982.63(1,864.38-2,111.62) | -0.09(-0.1 to -0.07) | -0.06(-0.09 to -0.03) | -0.04(-0.05 to -0.03) | -0.14(-0.16 to -0.13) |
| Bhutan | 5,078(4,773-5,404) | 1,936.72(1,820.32-2,060.88) | 3,645(3,433-3,859) | 1,947.25(1,834.32-2,061.57) | 0.02(-0.01 to 0.05) | -0.05(-0.11 to 0) | 0.06(0 to 0.13) | 0.03(0.01 to 0.05) |
| India | 6,737,656(6,358,724-7,127,249) | 2,063.47(1,947.42-2,182.78) | 7,333,201(6,929,548-7,743,700) | 2,001.42(1,891.26-2,113.46) | -0.1(-0.11 to -0.08) | -0.21(-0.23 to -0.19) | -0.04(-0.04 to -0.04) | -0.07(-0.1 to -0.04) |
| Nepal | 167,696(158,486-177,928) | 1,990.41(1,881.09-2,111.86) | 182,752(172,447-195,622) | 1,980.58(1,868.89-2,120.05) | -0.01(-0.03 to 0) | 0.05(0.04 to 0.07) | -0.1(-0.12 to -0.09) | 0.03(0 to 0.06) |
| Pakistan | 982,972(930,149-1,034,935) | 1,996.21(1,888.93-2,101.73) | 1,684,860(1,594,758-1,784,189) | 1,971.93(1,866.48-2,088.18) | -0.03(-0.05 to -0.01) | 0.04(-0.02 to 0.09) | -0.15(-0.19 to -0.12) | 0(-0.04 to 0.03) |
| Angola | 81,915(77,350-87,360) | 1,737.4(1,640.58-1,852.89) | 298,531(279,147-319,855) | 1,958.16(1,831.03-2,098.03) | 0.39(0.37 to 0.41) | 0.28(0.25 to 0.31) | 0.53(0.51 to 0.55) | 0.34(0.31 to 0.37) |
| Central African Republic | 21,030(19,859-22,396) | 1,720.02(1,624.24-1,831.72) | 40,354(38,356-43,042) | 1,766.94(1,679.47-1,884.67) | 0.09(0.08 to 0.09) | 0.07(0.07 to 0.08) | 0.13(0.11 to 0.14) | 0.06(0.06 to 0.07) |
| Congo | 19,468(18,388-20,802) | 1,848.87(1,746.33-1,975.59) | 38,493(36,145-41,256) | 1,995.21(1,873.49-2,138.44) | 0.25(0.23 to 0.26) | 0.19(0.19 to 0.2) | 0.41(0.39 to 0.43) | 0.13(0.1 to 0.17) |
| Democratic Republic of the Congo | 315,529(299,268-337,571) | 1,782.27(1,690.42-1,906.77) | 703,312(666,149-746,605) | 1,850.87(1,753.07-1,964.8) | 0.13(0.1 to 0.15) | -0.2(-0.24 to -0.16) | 0.13(0.05 to 0.21) | 0.39(0.36 to 0.41) |
| Equatorial Guinea | 3,415(3,239-3,639) | 1,734.14(1,644.72-1,847.76) | 12,106(11,325-12,892) | 2,069.38(1,935.95-2,203.82) | 0.58(0.55 to 0.6) | 0.58(0.52 to 0.63) | 0.93(0.92 to 0.95) | 0.24(0.21 to 0.28) |
| Gabon | 7,717(7,268-8,184) | 1,893.67(1,783.55-2,008.26) | 13,285(12,501-14,161) | 2,078.51(1,955.97-2,215.62) | 0.3(0.3 to 0.31) | 0.41(0.4 to 0.41) | 0.27(0.27 to 0.27) | 0.24(0.23 to 0.25) |
| Burundi | 64,577(61,296-68,345) | 2,463.71(2,338.53-2,607.48) | 147,909(140,102-155,939) | 2,526.57(2,393.21-2,663.73) | 0.08(0.06 to 0.11) | -0.01(-0.05 to 0.03) | 0.09(0.03 to 0.15) | 0.15(0.12 to 0.18) |
| Comoros | 5,341(5,037-5,641) | 2,511.07(2,368.43-2,652.4) | 6,445(6,085-6,813) | 2,683.73(2,533.81-2,837.07) | 0.22(0.21 to 0.22) | 0.31(0.3 to 0.32) | 0.17(0.16 to 0.17) | 0.18(0.17 to 0.19) |
| Djibouti | 4,316(4,091-4,558) | 2,479(2,349.67-2,618.03) | 10,992(10,442-11,603) | 2,660.46(2,527.25-2,808.24) | 0.24(0.2 to 0.27) | 0.11(0.06 to 0.16) | 0.26(0.18 to 0.33) | 0.36(0.31 to 0.41) |
| Eritrea | 39,029(37,054-41,410) | 2,451.78(2,327.68-2,601.32) | 66,491(63,270-70,683) | 2,633.68(2,506.11-2,799.75) | 0.24(0.21 to 0.27) | 0.34(0.24 to 0.43) | 0.14(0.11 to 0.17) | 0.23(0.22 to 0.24) |
| Ethiopia | 625,614(596,149-657,726) | 2,567.79(2,446.85-2,699.59) | 1,198,442(1,147,373-1,254,150) | 2,702.21(2,587.06-2,827.82) | 0.17(0.16 to 0.18) | 0.01(-0.01 to 0.04) | 0.25(0.24 to 0.26) | 0.2(0.18 to 0.21) |
| Kenya | 284,727(271,116-299,727) | 2,549(2,427.14-2,683.28) | 502,145(477,956-531,053) | 2,690.27(2,560.67-2,845.14) | 0.17(0.16 to 0.18) | 0.05(0.04 to 0.06) | 0.27(0.26 to 0.29) | 0.18(0.16 to 0.2) |
| Madagascar | 136,375(129,048-143,585) | 2,499.55(2,365.26-2,631.7) | 306,952(290,580-324,632) | 2,616.02(2,476.5-2,766.7) | 0.15(0.15 to 0.15) | 0.07(0.06 to 0.07) | 0.15(0.15 to 0.15) | 0.22(0.21 to 0.23) |
| Malawi | 110,679(105,039-117,099) | 2,432.72(2,308.75-2,573.82) | 210,656(200,045-222,459) | 2,593.05(2,462.44-2,738.34) | 0.21(0.18 to 0.24) | 0.05(-0.02 to 0.12) | 0.44(0.41 to 0.47) | 0.14(0.11 to 0.17) |
| Mauritius | 6,819(6,434-7,252) | 2,066.14(1,949.28-2,197.1) | 4,462(4,209-4,733) | 2,151.57(2,029.52-2,282.22) | 0.13(0.11 to 0.16) | 0.61(0.55 to 0.68) | 0(-0.04 to 0.03) | -0.07(-0.1 to -0.04) |
| Mozambique | 147,788(140,649-156,104) | 2,382.08(2,267.02-2,516.12) | 366,572(347,554-386,565) | 2,569.55(2,436.25-2,709.69) | 0.25(0.24 to 0.25) | 0.18(0.16 to 0.19) | 0.29(0.28 to 0.3) | 0.24(0.22 to 0.26) |
| Rwanda | 85,384(81,004-90,255) | 2,516.56(2,387.46-2,660.12) | 131,654(124,898-139,396) | 2,648.76(2,512.83-2,804.51) | 0.16(0.11 to 0.21) | -0.19(-0.26 to -0.12) | 0.47(0.36 to 0.58) | 0.19(0.15 to 0.23) |
| Seychelles | 509(480-541) | 2,146.26(2,024.63-2,279.75) | 517(488-547) | 2,208.92(2,082.8-2,338.22) | 0.09(0.08 to 0.11) | 0.22(0.18 to 0.27) | 0.03(0.01 to 0.04) | 0.04(0.01 to 0.07) |
| Somalia | 89,491(85,139-94,696) | 2,297.2(2,185.48-2,430.82) | 240,736(228,595-253,759) | 2,330.59(2,213.05-2,456.66) | 0.04(0.02 to 0.06) | -0.07(-0.1 to -0.05) | 0.14(0.11 to 0.17) | 0.03(-0.01 to 0.08) |
| United Republic of Tanzania | 299,536(283,949-315,783) | 2,480.52(2,351.44-2,615.06) | 644,801(612,528-682,834) | 2,642.31(2,510.06-2,798.17) | 0.21(0.2 to 0.21) | 0.13(0.12 to 0.14) | 0.23(0.23 to 0.24) | 0.25(0.23 to 0.27) |
| Uganda | 201,391(191,347-213,279) | 2,391.92(2,272.64-2,533.11) | 521,032(492,948-550,975) | 2,626.74(2,485.17-2,777.69) | 0.31(0.29 to 0.32) | 0.22(0.2 to 0.23) | 0.41(0.39 to 0.43) | 0.29(0.27 to 0.31) |
| Zambia | 93,698(88,756-99,281) | 2,495.59(2,363.95-2,644.29) | 224,838(212,237-237,942) | 2,718.21(2,565.88-2,876.62) | 0.28(0.27 to 0.29) | 0.18(0.18 to 0.18) | 0.27(0.25 to 0.3) | 0.35(0.33 to 0.37) |
| Botswana | 12,057(11,367-12,797) | 2,042.09(1,925.23-2,167.32) | 15,355(14,426-16,335) | 2,199.04(2,065.91-2,339.38) | 0.24(0.23 to 0.25) | 0.22(0.22 to 0.23) | 0.33(0.32 to 0.35) | 0.16(0.15 to 0.18) |
| Lesotho | 13,419(12,655-14,333) | 1,965.84(1,854.01-2,099.82) | 13,131(12,415-13,961) | 2,082.62(1,969.16-2,214.34) | 0.19(0.18 to 0.2) | 0.2(0.19 to 0.21) | 0.22(0.2 to 0.23) | 0.16(0.14 to 0.17) |
| Namibia | 12,368(11,644-13,200) | 2,058.89(1,938.32-2,197.25) | 17,957(16,841-19,179) | 2,175.44(2,040.23-2,323.54) | 0.18(0.16 to 0.2) | 0.25(0.2 to 0.3) | 0.11(0.1 to 0.12) | 0.19(0.15 to 0.22) |
| South Africa | 290,200(275,198-307,966) | 2,131.71(2,021.51-2,262.21) | 338,293(320,723-358,315) | 2,224.86(2,109.3-2,356.53) | 0.14(0.12 to 0.16) | 0.01(-0.02 to 0.03) | 0.25(0.24 to 0.27) | 0.13(0.08 to 0.18) |
| Eswatini | 7,839(7,396-8,295) | 2,032.32(1,917.32-2,150.33) | 8,907(8,392-9,439) | 2,158.45(2,033.83-2,287.48) | 0.19(0.19 to 0.2) | 0.15(0.13 to 0.17) | 0.19(0.19 to 0.2) | 0.22(0.2 to 0.24) |
| Zimbabwe | 97,652(91,921-103,546) | 2,027.54(1,908.53-2,149.9) | 130,490(122,921-138,491) | 2,073.3(1,953.03-2,200.42) | 0.08(0.06 to 0.09) | 0.04(0.02 to 0.07) | -0.05(-0.08 to -0.02) | 0.23(0.2 to 0.26) |
| Benin | 44,687(42,057-47,759) | 1,845.21(1,736.6-1,972.07) | 118,589(112,210-126,508) | 1,950.35(1,845.44-2,080.58) | 0.18(0.17 to 0.19) | 0.09(0.08 to 0.1) | 0.14(0.14 to 0.15) | 0.29(0.27 to 0.3) |
| Burkina Faso | 82,482(78,274-87,392) | 1,747.85(1,658.68-1,851.89) | 193,780(183,011-206,419) | 1,868.27(1,764.45-1,990.13) | 0.22(0.21 to 0.23) | 0.1(0.09 to 0.12) | 0.33(0.31 to 0.34) | 0.23(0.22 to 0.24) |
| Cameroon | 93,420(88,121-99,333) | 1,913.54(1,805.01-2,034.66) | 276,079(260,009-295,281) | 2,050.05(1,930.72-2,192.64) | 0.22(0.21 to 0.24) | 0.05(0.02 to 0.07) | 0.29(0.27 to 0.32) | 0.31(0.29 to 0.34) |
| Cabo Verde | 2,993(2,820-3,206) | 1,902.34(1,792.36-2,037.73) | 2,924(2,760-3,119) | 2,042(1,927.65-2,177.72) | 0.22(0.19 to 0.25) | 0.03(0.01 to 0.05) | 0.29(0.23 to 0.35) | 0.29(0.24 to 0.35) |
| Chad | 50,686(48,090-53,678) | 1,732.04(1,643.35-1,834.28) | 165,299(157,424-175,782) | 1,833.62(1,746.26-1,949.91) | 0.18(0.18 to 0.19) | 0.12(0.12 to 0.12) | 0.29(0.28 to 0.29) | 0.16(0.15 to 0.17) |
| Coted'Ivoire | 107,008(101,044-113,859) | 1,876.24(1,771.68-1,996.37) | 231,361(218,739-245,968) | 1,999.36(1,890.29-2,125.58) | 0.2(0.2 to 0.21) | 0.01(0 to 0.02) | 0.26(0.25 to 0.27) | 0.32(0.31 to 0.33) |
| Gambia | 8,507(8,060-9,055) | 1,844.35(1,747.37-1,963.15) | 19,530(18,428-20,800) | 1,965.85(1,855-2,093.75) | 0.21(0.2 to 0.21) | 0.04(0.04 to 0.05) | 0.38(0.37 to 0.39) | 0.18(0.16 to 0.19) |
| Ghana | 131,317(123,984-139,890) | 1,955.12(1,845.94-2,082.75) | 274,994(258,498-292,730) | 2,134.54(2,006.49-2,272.21) | 0.29(0.27 to 0.3) | 0.18(0.15 to 0.2) | 0.17(0.14 to 0.2) | 0.47(0.45 to 0.5) |
| Guinea | 49,612(46,866-52,895) | 1,802.88(1,703.08-1,922.18) | 116,267(109,869-123,575) | 1,923.11(1,817.29-2,044) | 0.21(0.2 to 0.22) | 0.14(0.13 to 0.15) | 0.17(0.14 to 0.2) | 0.31(0.29 to 0.33) |
| Guinea-Bissau | 8,772(8,294-9,325) | 1,818.35(1,719.37-1,933.09) | 17,500(16,580-18,605) | 1,948.47(1,846.02-2,071.47) | 0.22(0.21 to 0.23) | 0.09(0.08 to 0.09) | 0.26(0.24 to 0.29) | 0.29(0.28 to 0.31) |
| Liberia | 20,853(19,785-22,177) | 1,845.08(1,750.62-1,962.27) | 42,113(39,928-44,727) | 1,926.54(1,826.62-2,046.12) | 0.13(0.06 to 0.2) | -0.39(-0.52 to -0.26) | 0.43(0.34 to 0.53) | 0.18(0.12 to 0.23) |
| Mali | 71,959(68,060-76,546) | 1,742.43(1,648.01-1,853.49) | 217,088(205,510-231,577) | 1,875.36(1,775.35-2,000.53) | 0.24(0.23 to 0.25) | 0.14(0.12 to 0.16) | 0.27(0.26 to 0.27) | 0.29(0.28 to 0.3) |
| Mauritania | 17,719(16,700-18,836) | 1,916.93(1,806.64-2,037.68) | 38,310(36,188-40,752) | 2,067.45(1,952.96-2,199.23) | 0.25(0.24 to 0.25) | 0.22(0.21 to 0.24) | 0.27(0.26 to 0.28) | 0.24(0.22 to 0.25) |
| Niger | 68,925(65,334-73,029) | 1,696.42(1,608.04-1,797.44) | 223,055(211,782-236,241) | 1,747.53(1,659.22-1,850.84) | 0.1(0.09 to 0.11) | 0(-0.01 to 0.02) | 0.18(0.16 to 0.21) | 0.09(0.07 to 0.1) |
| Nigeria | 731,503(695,396-772,842) | 1,869.68(1,777.39-1,975.34) | 2,069,240(1,968,057-2,187,339) | 2,036.87(1,937.28-2,153.12) | 0.28(0.27 to 0.29) | 0.07(0.06 to 0.08) | 0.45(0.43 to 0.47) | 0.29(0.27 to 0.3) |
| Sao Tome and Principe | 1,070(1,010-1,141) | 1,887.89(1,781.70-2,013.21) | 1,579(1,491-1,684) | 2,028.21(1,915.29-2,164.21) | 0.24(0.22 to 0.26) | -0.05(-0.09 to -0.02) | 0.66(0.63 to 0.68) | 0.13(0.1 to 0.16) |
| Senegal | 67,446(63,761-71,756) | 1,847.2(1,746.25-1,965.23) | 125,454(118,620-133,541) | 1,972.37(1,864.93-2,099.51) | 0.21(0.2 to 0.22) | 0.09(0.08 to 0.1) | 0.29(0.27 to 0.32) | 0.24(0.22 to 0.26) |
| Sierra Leone | 33,061(31,504-35,037) | 1,823.92(1,738.04-1,932.94) | 68,881(65,236-73,307) | 1,926.1(1,824.17-2,049.85) | 0.18(0.16 to 0.2) | -0.08(-0.12 to -0.04) | 0.28(0.26 to 0.31) | 0.33(0.32 to 0.35) |
| Togo | 33,046(31,095-35,326) | 1,874.9(1,764.20-2,004.28) | 65,816(62,196-70,064) | 1,988.96(1,879.58-2,117.35) | 0.19(0.18 to 0.2) | 0.1(0.09 to 0.12) | 0.18(0.15 to 0.2) | 0.27(0.26 to 0.28) |
| American Samoa | 443(417-471) | 2,326.55(2,193.82-2,476.23) | 320(301-342) | 2,258.61(2,120.11-2,408.11) | -0.1(-0.12 to -0.07) | 0.18(0.14 to 0.22) | -0.22(-0.24 to -0.21) | -0.22(-0.27 to -0.17) |
| Bermuda | 250(236-266) | 2,097.66(1,980.92-2,231.02) | 175(164-186) | 2,068.46(1,939.55-2,208.56) | -0.06(-0.09 to -0.03) | 0.02(-0.04 to 0.07) | 0.14(0.06 to 0.22) | -0.29(-0.32 to -0.26) |
| Cook Islands | 153(144-162) | 2,314.33(2,184.57-2,457.05) | 87(82-92) | 2,296.5(2,156.15-2,439.44) | -0.03(-0.08 to 0.02) | 0.17(0.07 to 0.28) | -0.19(-0.28 to -0.11) | -0.18(-0.23 to -0.14) |
| Greenland | 473(450-500) | 3,327.95(3,160.81-3,512.75) | 398(378-420) | 3,384(3,217.97-3,572.91) | 0.06(-0.02 to 0.14) | 0.1(0.01 to 0.19) | -0.26(-0.37 to -0.16) | 0.33(0.15 to 0.51) |
| Guam | 986(930-1,048) | 2,364.64(2,229.51-2,513.01) | 866(815-920) | 2,367.88(2,229.22-2,515.2) | 0(-0.02 to 0.03) | 0.3(0.27 to 0.34) | -0.35(-0.43 to -0.27) | -0.01(-0.03 to 0.01) |
| Monaco | 81(76-86) | 2,305.2(2,167.65-2,438.35) | 117(111-124) | 2,359.09(2,224.83-2,491.99) | 0.08(0.03 to 0.14) | 0.1(0.05 to 0.15) | -0.15(-0.2 to -0.1) | 0.32(0.19 to 0.45) |
| Nauru | 95(89-101) | 2,251.81(2,119.97-2,386.84) | 89(84-95) | 2,247.55(2,113.64-2,392.03) | -0.01(-0.03 to 0.01) | -0.24(-0.27 to -0.21) | 0.1(0.06 to 0.13) | 0.08(0.07 to 0.1) |
| Niue | 19(17-20) | 2,305.04(2,165.43-2,453.3) | 9(8-9) | 2,276.28(2,143.71-2,421.77) | -0.04(-0.08 to -0.01) | -0.2(-0.28 to -0.12) | 0.42(0.37 to 0.47) | -0.23(-0.27 to -0.19) |
| Northern Mariana Islands | 285(269-303) | 2,342.34(2,211.02-2,491.22) | 261(245-278) | 2,315.25(2,173.50-2,466.99) | -0.05(-0.13 to 0.03) | 0.19(0.06 to 0.32) | 0.19(0.06 to 0.32) | -0.24(-0.39 to -0.08) |
| Palau | 104(98-110) | 2,281.97(2,150.22-2,424.11) | 74(70-79) | 2,278.58(2,145.15-2,420.42) | 0(-0.02 to 0.02) | 0.35(0.3 to 0.39) | -0.2(-0.22 to -0.19) | -0.13(-0.16 to -0.09) |
| Puerto Rico | 20,175(19,040-21,494) | 2,026.03(1,912.09-2,158.53) | 8,929(8,396-9,488) | 2,009.28(1,889.44-2,135.1) | -0.02(-0.05 to 0.01) | 0.31(0.29 to 0.34) | -0.14(-0.18 to -0.1) | -0.22(-0.3 to -0.14) |
| Saint Kitts and Nevis | 283(267-301) | 2,002.97(1,892.79-2,130.53) | 200(189-213) | 2,030.93(1,914.89-2,167.47) | 0.04(0.03 to 0.06) | 0.07(0.04 to 0.1) | 0.03(-0.01 to 0.06) | 0.1(0.07 to 0.12) |
| San Marino | 91(86-96) | 2,213.71(2,086.84-2,339.09) | 98(92-104) | 2,226.84(2,100.77-2,362.96) | 0.03(0 to 0.06) | 0.5(0.49 to 0.52) | -0.04(-0.12 to 0.04) | -0.3(-0.35 to -0.25) |
| Tokelau | 13(13-14) | 2,212.6(2,084.6-2,356.87) | 9(8-9) | 2,214.86(2,074.63-2,367.91) | 0.06(-0.28 to 0.4) | 0.21(-0.37 to 0.78) | 0.18(-0.36 to 0.73) | 0.08(-0.19 to 0.36) |
| Tuvalu | 75(71-80) | 2,169.74(2,039.37-2,304.38) | 82(78-87) | 2,198.79(2,080.03-2,343.52) | 0.04(0.03 to 0.05) | 0.22(0.21 to 0.24) | -0.01(-0.02 to 0.01) | -0.06(-0.07 to -0.05) |
| United States Virgin Islands | 658(620-702) | 2,059.87(1,939.81-2,197.76) | 277(261-294) | 2,066.66(1,946.49-2,198.31) | 0.01(-0.01 to 0.03) | 0.17(0.12 to 0.22) | 0.06(0.05 to 0.08) | -0.17(-0.2 to -0.13) |
| South Sudan | 64,027(60,983-67,843) | 2,439.91(2,323.93-2,585.31) | 106,205(100,752-111,818) | 2,472.8(2,345.84-2,603.47) | 0.04(0.03 to 0.05) | 0.12(0.11 to 0.13) | 0.16(0.14 to 0.17) | -0.13(-0.15 to -0.11) |
| Sudan | 153,929(145,850-163,781) | 1,731.01(1,640.15-1,841.81) | 316,759(297,928-338,750) | 1,909.42(1,795.91-2,041.98) | 0.31(0.3 to 0.33) | 0.38(0.36 to 0.4) | 0.35(0.33 to 0.36) | 0.23(0.21 to 0.25) |

**sTable6 Number of DALYs and DALYs rate of bacterial skin diseases and their AAPCs from 1990 to 2021 at the global, regional and national levels**

| *Rate per 100,000* | 1990 | | 2021 | | 1990-2021 | 1990-1999 | 2000-2009 | 2010-2021 |
| --- | --- | --- | --- | --- | --- | --- | --- | --- |
| *AAPC(95% CI)* | Number of DALYs | DALYs rate | Number of DALYs | DALYs rate | AAPC | AAPC | AAPC | AAPC |
| Bacterial skin diseases | 778,691(618,954-977,771) | 44.77(35.59-56.22) | 440,459(280,002-600,523) | 21.89(13.92-29.85) | -2.28(-2.5 to -2.05) | -0.85(-0.96 to -0.75) | -1.77(-1.86 to -1.69) | -3.9(-4.5 to -3.31) |
| Male | 373,295(299,859-465,207) | 41.78(33.56-52.07) | 228,601(138,924-318,831) | 22.02(13.38-30.71) | -2.05(-2.28 to -1.82) | -1.62(-1.83 to -1.41) | -1.27(-1.51 to -1.03) | -3.08(-3.52 to -2.63) |
| Female | 405,395(290,141-520,207) | 47.94(34.31-61.51) | 211,858(130,172-299,332) | 21.76(13.37-30.74) | -2.55(-2.79 to -2.31) | -0.32(-0.44 to -0.21) | -2.26(-2.53 to -1.98) | -4.65(-5.25 to -4.05) |
| **Age groups** |  |  |  |  |  |  |  |  |
| 0-4 years | 679,948(531,077-863,957) | 109.68(85.67-139.36) | 355,187(210,278-495,585) | 53.97(31.95-75.3) | -2.3 (-2.48 to -2.12) | -0.44(-0.5 to -0.37) | -2.48(-2.64 to -2.32) | -3.7(-4.16 to -3.23) |
| 5-9 years | 51,227(37,181-69,799) | 8.78(6.37-11.96) | 39,686(25,859-61,640) | 5.78(3.76-8.97) | -1.34 (-1.68 to -1) | 0.68(0.51 to 0.86) | -1.29(-2.28 to -0.28) | -3(-3.41 to -2.6) |
| 10-14 years | 47,516(35,753-63,120) | 8.87(6.67-11.78) | 45,586(32,196-66,835) | 6.84(4.83-10.03) | -0.81 (-1.22 to -0.4) | 0.51(0.29 to 0.73) | -0.55(-1.66 to 0.57) | -2.05(-2.68 to -1.41) |
| **SDI region** |  |  |  |  |  |  |  |  |
| High SDI | 11,307(8,861-14,876) | 6.09(4.77-8.01) | 9,058(6,734-12,429) | 5.25(3.9-7.2) | -0.48 (-0.69 to -0.27) | -1.37(-1.57 to -1.17) | 0.24(0.04 to 0.43) | -0.41(-0.88 to 0.06) |
| High-middle SDI | 49,291(42,737-57,658) | 18.01(15.62-21.07) | 11,596(8,945-15,684) | 5.02(3.87-6.79) | -4.05 (-4.26 to -3.84) | -4.5(-4.94 to -4.05) | -5.07(-5.25 to -4.88) | -2.78(-3.14 to -2.43) |
| Middle SDI | 175,194(150,329-205,409) | 30.35(26.04-35.59) | 71,507(52,164-93,030) | 12.61(9.2-16.41) | -2.86 (-3.43 to -2.28) | -2.61(-3.01 to -2.2) | -2.33(-2.68 to -1.98) | -3.74(-5.14 to -2.31) |
| Low-middle SDI | 350,134(267,070-450,243) | 74.16(56.57-95.37) | 174,529(105,840-252,346) | 30.1(18.25-43.52) | -2.92 (-3.26 to -2.58) | -0.75(-0.98 to -0.53) | -2.67(-3.05 to -2.29) | -4.95(-5.81 to -4.08) |
| Low SDI | 192,338(135,831-261,612) | 84.02(59.34-114.28) | 173,330(103,733-241,418) | 37.66(22.54-52.46) | -2.57 (-2.72 to -2.42) | -1.46(-1.58 to -1.34) | -2.57(-2.87 to -2.26) | -3.51(-3.8 to -3.23) |
| **GBD region** |  |  |  |  |  |  |  |  |
| Andean Latin America | 1,636(995-2,370) | 11.02(6.7-15.96) | 1,762(1,347-2,292) | 9.74(7.45-12.67) | -0.2(-1.1 to 0.72) | 6.66(4.86 to 8.49) | -10.66(-11.37 to -9.94) | 2.4(0.87 to 3.96) |
| Australasia | 295(187-466) | 6.44(4.08-10.17) | 377(242-598) | 6.58(4.23-10.44) | 0.12(-0.08 to 0.33) | 0.36(0.07 to 0.65) | 0.87(0.71 to 1.03) | -0.71(-1.18 to -0.23) |
| Caribbean | 3,562(1,527-6,406) | 31.21(13.38-56.14) | 4,724(2,359-8,299) | 41.06(20.51-72.13) | 0.92(0.72 to 1.12) | 1.55(0.97 to 2.12) | 1.18(1.02 to 1.33) | 0.16(-0.05 to 0.37) |
| Central Asia | 1,157(778-1,724) | 4.63(3.11-6.9) | 2,141(1,641-2,844) | 7.74(5.93-10.28) | 1.45(0.02 to 2.9) | -1.61(-2.9 to -0.3) | 2.51(1.04 to 4) | 2.91(-0.58 to 6.53) |
| Central Europe | 2,998(2,511-3,649) | 10.17(8.52-12.38) | 1,259(1,030-1,585) | 7.11(5.82-8.95) | -1.18(-2.32 to -0.02) | -8.57(-10.95 to -6.13) | 1.43(0.62 to 2.24) | 2.04(0.13 to 3.99) |
| Central Latin America | 11,414(10,284-12,785) | 17.73(15.97-19.86) | 7,730(6,175-9,765) | 12.18(9.73-15.38) | -1.23(-2.46 to 0.01) | -5.05(-6.04 to -4.06) | 1.06(-0.25 to 2.38) | 0.28(-2.47 to 3.1) |
| Central Sub-Saharan Africa | 31,026(20,331-43,373) | 122.64(80.36-171.44) | 27,856(11,502-52,449) | 47.47(19.6-89.38) | -3.02(-3.2 to -2.85) | -1.21(-1.27 to -1.15) | -1.86(-2.19 to -1.52) | -5.62(-5.98 to -5.26) |
| East Asia | 108,464(91,021-125,957) | 32.88(27.6-38.19) | 6,516(4,441-9,345) | 2.44(1.66-3.5) | -7.9(-8.62 to -7.17) | -6.9(-8.16 to -5.62) | -10.83(-11.66 to -10) | -5.82(-6.53 to -5.11) |
| Eastern Europe | 3,784(2,958-5,135) | 7.35(5.75-9.98) | 2,448(1,889-3,374) | 6.91(5.33-9.52) | -0.19(-0.94 to 0.57) | 0.19(-2.19 to 2.64) | 0.92(0.67 to 1.17) | -1.59(-2.3 to -0.87) |
| Eastern Sub-Saharan Africa | 117,724(70,090-168,839) | 129.98(77.39-186.42) | 102,908(59,634-150,889) | 57.67(33.42-84.56) | -2.59(-2.7 to -2.48) | -2.41(-2.72 to -2.1) | -2.02(-2.15 to -1.88) | -3.22(-3.35 to -3.1) |
| High-income Asia Pacific | 1,254(916-1,802) | 3.56(2.6-5.12) | 927(699-1,262) | 4.13(3.12-5.63) | 0.49(0.16 to 0.82) | 0.72(0.31 to 1.13) | 0.58(0.28 to 0.87) | 0.18(-0.59 to 0.95) |
| High-income North America | 4,301(3,193-5,788) | 6.97(5.18-9.38) | 4,759(3,611-6,309) | 7.25(5.50-9.61) | 0.2(-0.11 to 0.5) | -1.2(-1.75 to -0.65) | 1.42(1.12 to 1.71) | 0.09(-0.35 to 0.53) |
| North Africa and Middle East | 9,357(6,130-14,027) | 6.66(4.36-9.98) | 8,389(5,221-13,558) | 4.58(2.85-7.4) | -1.21(-1.29 to -1.13) | -1.12(-1.27 to -0.96) | -0.4(-0.44 to -0.37) | -2.06(-2.24 to -1.87) |
| Oceania | 1,479(856-2,283) | 55.19(31.94-85.19) | 2,685(1,588-4,148) | 52.85(31.26-81.63) | -0.14(-0.37 to 0.1) | -0.03(-0.36 to 0.31) | 0.83(0.12 to 1.54) | -1.08(-1.17 to -1) |
| South Asia | 390,667(292,675-498,931) | 90.15(67.54-115.13) | 167,348(100,055-251,413) | 33.01(19.73-49.59) | -3.28(-3.59 to -2.96) | -0.82(-0.97 to -0.67) | -3.36(-3.62 to -3.1) | -5.21(-6.04 to -4.38) |
| Southeast Asia | 50,032(35,122-67,666) | 29.3(20.57-39.63) | 29,373(21,405-36,225) | 17.01(12.4-20.98) | -1.72(-1.99 to -1.44) | -2.28(-2.55 to -2) | -0.28(-0.39 to -0.16) | -2.51(-3.22 to -1.79) |
| Southern Latin America | 823(590-1,161) | 5.52(3.95-7.78) | 1,383(1,146-1,778) | 9.54(7.9-12.27) | 1.74(0.78 to 2.72) | 3.72(0.98 to 6.53) | 1.12(-0.03 to 2.3) | 0.66(-0.28 to 1.61) |
| Southern Sub-Saharan Africa | 6,681(4,065-9,008) | 32.29(19.65-43.54) | 5,450(3,244-8,400) | 22.65(13.48-34.9) | -1.14(-1.41 to -0.87) | 0.72(-0.15 to 1.6) | -1.03(-1.24 to -0.83) | -2.66(-2.8 to -2.52) |
| Tropical Latin America | 12,998(10,878-15,776) | 24.24(20.29-29.42) | 11,044(9,086-13,264) | 22(18.1-26.43) | -0.17(-1.35 to 1.02) | 3.62(2.99 to 4.25) | -1.84(-4.65 to 1.05) | -2.15(-4.3 to 0.04) |
| Western Europe | 2,201(1,405-3,499) | 3.1(1.98-4.93) | 2,308(1,521-3,550) | 3.39(2.23-5.21) | 0.28(0.07 to 0.49) | 0.45(0.26 to 0.65) | 0.8(0.48 to 1.13) | -0.34(-0.78 to 0.1) |
| Western Sub-Saharan Africa | 16,836(12,581-22,780) | 19.16(14.32-25.92) | 49,069(31,273-69,049) | 22.85(14.56-32.15) | 0.59(0.42 to 0.75) | 4.51(4.17 to 4.84) | 0.39(0.17 to 0.61) | -2.77(-2.95 to -2.6) |
| **204 countries and territories** |  |  |  |  |  |  |  |  |
| China | 106,930(89,509-124,297) | 33.59(28.11-39.04) | 6,118(4,154-8,782) | 2.36(1.6-3.38) | -8.34(-8.92 to -7.75) | -7.16(-7.46 to -6.85) | -11.07(-11.71 to-10.44) | -6.64(-8.09 to -5.18) |
| Democratic People's Republic of Korea | 684(233-1,169) | 11.49(3.92-19.65) | 185(90-316) | 3.88(1.88-6.63) | -3.47(-3.61 to -3.32) | -3.83(-3.99 to -3.66) | -2.94(-3.33 to -2.54) | -3.44(-3.62 to -3.25) |
| Taiwan (Province of China) | 850(761-974) | 15.43(13.81-17.69) | 213(170-275) | 7.23(5.76-9.34) | -2.71(-3.11 to -2.31) | -1(-2.16 to 0.17) | -3.4(-3.69 to -3.11) | -3.4(-3.69 to -3.11) |
| Cambodia | 1,780(871-3,284) | 38.19(18.69-70.45) | 689(390-1,016) | 13.47(7.63-19.85) | -3.31(-3.42 to -3.19) | -3.05(-3.24 to -2.86) | -3.82(-3.95 to -3.69) | -3(-3.23 to -2.78) |
| Indonesia | 24,609(16,227-33,929) | 36.33(23.96-50.09) | 13,008(8,812-17,523) | 19.33(13.1-26.04) | -2.01(-2.23 to -1.79) | -2.58(-2.95 to -2.21) | -0.52(-0.7 to -0.33) | -2.86(-3.31 to -2.41) |
| Lao People's Democratic Republic | 771(279-1,657) | 41.85(15.16-89.92) | 389(197-644) | 16.95(8.58-28.06) | -2.87(-3.02 to -2.72) | -2.09(-2.31 to -1.88) | -3.55(-3.9 to -3.2) | -3.04(-3.26 to -2.83) |
| Malaysia | 517(301-766) | 7.86(4.58-11.66) | 597(373-830) | 7.84(4.9-10.91) | -0.06(-1.26 to 1.16) | 0(-1.06 to 1.07) | 4.03(0.66 to 7.52) | -3.51(-5.11 to -1.88) |
| Maldives | 23(11-41) | 22.1(10.86-39.37) | 10(6-16) | 10.21(6.05-15.9) | -2.52(-3.14 to -1.89) | -7.04(-7.82 to -6.26) | 3.17(1.9 to 4.45) | -3.76(-4.85 to -2.66) |
| Myanmar | 5,573(2,433-10,707) | 37.72(16.47-72.46) | 2,732(1,319-4,334) | 17.5(8.45-27.75) | -2.42(-2.59 to -2.26) | -0.56(-0.8 to -0.32) | -3.46(-3.82 to -3.1) | -2.91(-3.06 to -2.76) |
| Philippines | 11,081(8,050-14,720) | 43.95(31.92-58.38) | 9,056(7,182-11,228) | 26.64(21.12-33.03) | -1.6(-2.12 to -1.08) | -2.94(-3.45 to -2.43) | -0.15(-1.48 to 1.19) | -2.13(-2.6 to -1.66) |
| Sri Lanka | 174(128-226) | 3.14(2.3-4.09) | 125(89-170) | 2.44(1.74-3.33) | -0.66(-1.33 to 0.01) | -0.2(-0.98 to 0.58) | 3.06(1.09 to 5.06) | -4.2(-4.87 to -3.53) |
| Thailand | 2,785(1,901-3,985) | 16.52(11.27-23.64) | 1,562(1,252-1,985) | 15.99(12.82-20.33) | -0.08(-0.61 to 0.44) | -2.37(-3.12 to -1.62) | 0.87(-0.55 to 2.3) | 1.16(0.59 to 1.73) |
| Timor-Leste | 129(53-233) | 38.88(15.92-70.08) | 92(45-143) | 17.66(8.68-27.54) | -2.56(-2.73 to -2.4) | -2.56(-2.87 to -2.26) | -4.72(-5.12 to -4.32) | -0.91(-1.07 to -0.76) |
| Viet Nam | 2,509(1,721-3,398) | 9.46(6.49-12.82) | 1,051(685-1,583) | 4.24(2.77-6.39) | -2.55(-2.74 to -2.36) | -4.19(-4.33 to -4.05) | 0.22(-0.32 to 0.77) | -3.57(-3.71 to -3.43) |
| Fiji | 46(33-67) | 16.34(11.69-23.64) | 50(36-69) | 18.25(13.29-25.45) | 0.55(0.25 to 0.86) | 1.96(1.53 to 2.4) | 1.04(0.3 to 1.77) | -1.44(-1.82 to -1.06) |
| Kiribati | 48(28-72) | 163.05(96.37-244.29) | 32(23-44) | 76.21(53.55-105.02) | -2.42(-2.66 to -2.19) | -3.13(-3.48 to -2.77) | -1.09(-1.49 to -0.69) | -3.19(-3.6 to -2.77) |
| Marshall Islands | 5(4-7) | 24.41(16.75-33.88) | 3(2-5) | 18.84(12.68-26.45) | -0.82(-1.01 to -0.63) | 0.57(0.29 to 0.85) | -0.1(-0.35 to 0.15) | -2.6(-2.95 to -2.25) |
| Micronesia (Federated States of) | 13(8-20) | 28.42(17.59-43.39) | 4(3-7) | 14.13(9.46-21.48) | -2.28(-2.5 to -2.06) | -2.92(-3.04 to -2.8) | -1.59(-2.26 to -0.92) | -2.29(-2.52 to -2.07) |
| Papua New Guinea | 1,147(587-1,896) | 67.48(34.54-111.54) | 2,347(1,312-3,744) | 59.92(33.5-95.59) | -0.38(-0.64 to -0.12) | -0.32(-0.72 to 0.09) | 0.56(-0.13 to 1.24) | -1.32(-1.41 to -1.24) |
| Samoa | 13(8-20) | 18.92(11.6-28.41) | 11(7-18) | 14.24(9.25-22.03) | -0.88(-1.14 to -0.61) | -1.16(-1.48 to -0.84) | -0.46(-0.75 to -0.16) | -1.15(-1.43 to -0.88) |
| Solomon Islands | 63(39-94) | 40.25(24.97-60.59) | 69(40-123) | 26.65(15.35-47.33) | -1.26(-1.75 to -0.78) | -0.46(-2.09 to 1.19) | -1.59(-1.73 to -1.46) | -1.59(-1.73 to -1.46) |
| Tonga | 8(6-10) | 18.62(13.45-25.09) | 6(4-9) | 14.67(9.59-23.14) | -0.83(-1.11 to -0.55) | 0.15(-0.33 to 0.64) | -0.22(-0.95 to 0.5) | -2.29(-2.49 to -2.08) |
| Vanuatu | 23(15-35) | 33.49(21.34-50.71) | 26(16-42) | 22.45(13.83-36.23) | -1.23(-1.81 to -0.64) | 0.3(-0.64 to 1.25) | -0.63(-1.07 to -0.18) | -3.02(-4.13 to -1.89) |
| Armenia | 42(29-65) | 4.02(2.81-6.22) | 29(21-42) | 4.83(3.49-7.06) | 0.89(-1.34 to 3.18) | -0.14(-5.6 to 5.64) | 0.67(-0.95 to 2.33) | 1.13(-1.72 to 4.06) |
| Azerbaijan | 94(56-155) | 3.89(2.32-6.37) | 74(43-126) | 3.14(1.82-5.35) | -0.7(-0.82 to -0.58) | -0.66(-0.8 to -0.52) | 0.89(0.55 to 1.23) | -2.14(-2.22 to -2.05) |
| Georgia | 57(40-80) | 4.15(2.9-5.88) | 44(33-56) | 5.92(4.53-7.65) | 0.93(-0.84 to 2.74) | -3.99(-6.43 to -1.49) | 4.12(0.51 to 7.86) | 0.29(-1.92 to 2.54) |
| Kazakhstan | 192(127-304) | 3.70(2.44-5.84) | 651(411-1,038) | 11.99(7.57-19.13) | 4.08(2.59 to 5.58) | 1.44(-2.22 to 5.23) | 1.27(0.25 to 2.31) | 8.19(6.21 to 10.21) |
| Kyrgyzstan | 116(86-165) | 6.92(5.13-9.86) | 213(171-274) | 9.37(7.5-12.03) | 1.17(-1.73 to 4.17) | -0.22(-7.99 to 8.2) | 1.66(-0.3 to 3.66) | 1.74(-2.4 to 6.06) |
| Mongolia | 257(121-467) | 28.56(13.47-51.86) | 242(165-337) | 22.28(15.23-31.05) | -0.81(-1.24 to -0.38) | -3.37(-4.12 to -2.61) | 4.16(3.68 to 4.64) | -2.83(-3.72 to -1.94) |
| Tajikistan | 75(44-124) | 3.22(1.89-5.35) | 93(51-163) | 2.6(1.42-4.55) | -0.67(-0.77 to -0.57) | 0.32(0.19 to 0.45) | -1.7(-1.86 to -1.55) | -0.53(-0.74 to -0.33) |
| Turkmenistan | 71(48-106) | 4.73(3.17-7.07) | 260(206-336) | 17.06(13.49-22.03) | 4.49(2.44 to 6.59) | 0.42(-4.77 to 5.88) | 5.3(3.71 to 6.92) | 7.02(3.87 to 10.26) |
| Uzbekistan | 253(156-410) | 2.96(1.83-4.79) | 536(380-758) | 5.31(3.76-7.51) | 1.8(0.41 to 3.22) | -0.03(-3.59 to 3.66) | 2.96(2.49 to 3.43) | 2.16(-0.31 to 4.7) |
| Albania | 277(191-369) | 24.76(17.14-33.06) | 19(10-33) | 4.26(2.15-7.44) | -5.56(-5.89 to -5.23) | -8.93(-9.86 to -7.99) | -4.21(-4.56 to -3.85) | -4.03(-4.33 to -3.73) |
| Bosnia and Herzegovina | 37(20-60) | 3.34(1.81-5.5) | 17(11-26) | 3.5(2.29-5.2) | 0.25(-0.15 to 0.66) | -0.51(-1.43 to 0.42) | 2.38(2.1 to 2.67) | -0.81(-1.24 to -0.38) |
| Bulgaria | 151(128-183) | 8.68(7.4-10.56) | 82(67-105) | 8.42(6.83-10.75) | -0.21(-0.82 to 0.4) | -5.94(-6.77 to -5.11) | 3.97(3.07 to 4.87) | 1.18(0.16 to 2.21) |
| Croatia | 35(26-48) | 3.52(2.64-4.88) | 20(15-29) | 3.4(2.47-4.85) | -0.17(-0.85 to 0.52) | -4.79(-6.34 to -3.22) | 2.17(0.99 to 3.37) | 1.44(0.78 to 2.11) |
| Czechia | 271(236-315) | 12.29(10.73-14.27) | 160(128-203) | 9.3(7.43-11.86) | -0.96(-1.9 to 0) | -10.28(-12.12 to -8.39) | 4.21(3.83 to 4.59) | 2.06(0.04 to 4.14) |
| Hungary | 248(216-284) | 11.62(10.15-13.32) | 147(119-190) | 10.59(8.54-13.68) | -0.15(-1.75 to 1.47) | -8.61(-10.95 to -6.21) | 4.14(2.29 to 6.03) | 4.37(0.62 to 8.25) |
| North Macedonia | 19(12-29) | 3.58(2.35-5.49) | 7(4-13) | 2.23(1.25-3.89) | -1.52(-1.71 to -1.32) | -4.13(-4.55 to -3.71) | 0.81(0.4 to 1.22) | -1.2(-1.39 to -1) |
| Montenegro | 3(2-6) | 2.02(1.07-3.68) | 2(1-4) | 2.04(1.06-3.69) | 0.03(0 to 0.06) | -0.07(-0.15 to 0.02) | 0.13(0.08 to 0.19) | 0.01(-0.02 to 0.04) |
| Poland | 696(612-828) | 7.27(6.39-8.64) | 315(253-403) | 5.35(4.3-6.85) | -0.95(-1.9 to 0.01) | -11.26(-13.86 to -8.59) | 0.33(-0.66 to 1.32) | 6.16(5.24 to 7.08) |
| Romania | 909(604-1,374) | 16.33(10.84-24.68) | 337(275-422) | 11.18(9.13-14.02) | -1.01(-2.02 to 0.01) | -8.41(-10.5 to -6.27) | 1.65(0.67 to 2.63) | 2.25(0.56 to 3.95) |
| Serbia | 161(97-248) | 7.41(4.47-11.44) | 65(44-93) | 4.92(3.29-7.01) | -1.24(-2.03 to -0.44) | -2.26(-3.52 to -0.99) | 0(-1.96 to 1.99) | -1.1(-2.02 to -0.17) |
| Slovakia | 114(92-145) | 8.62(6.94-10.92) | 51(35-72) | 6(4.03-8.45) | -1.15(-1.61 to -0.69) | -4.48(-5.47 to -3.48) | 3.9(3.47 to 4.34) | -2.64(-3.34 to -1.93) |
| Slovenia | 31(26-38) | 7.4(6.17-9.22) | 18(14-23) | 5.67(4.51-7.39) | -0.9(-1.96 to 0.17) | -8.76(-10.37 to -7.13) | 3.93(1.92 to 5.99) | 1.25(-0.31 to 2.84) |
| Belarus | 95(56-161) | 3.95(2.33-6.71) | 60(34-101) | 3.79(2.17-6.43) | -0.15(-0.45 to 0.15) | -0.45(-1.11 to 0.23) | 0.39(-0.27 to 1.06) | -0.32(-0.6 to -0.04) |
| Estonia | 34(27-45) | 9.65(7.61-12.8) | 11(8-17) | 5.29(3.65-7.99) | -1.95(-2.67 to -1.23) | -4.56(-5.03 to -4.09) | -0.18(-1.69 to 1.35) | -1.15(-2.29 to 0) |
| Latvia | 64(53-82) | 11.34(9.23-14.34) | 19(14-26) | 6.29(4.68-8.86) | -1.86(-2.68 to -1.03) | -5.03(-6.45 to -3.59) | 1.28(0.37 to 2.2) | -2.07(-3.65 to -0.48) |
| Lithuania | 70(54-94) | 8.44(6.56-11.35) | 24(17-36) | 5.98(4.27-8.86) | -1.2(-1.82 to -0.58) | -3.75(-5.45 to -2.02) | 0.91(0.61 to 1.21) | -1.18(-2.04 to -0.3) |
| Republic of Moldova | 126(93-166) | 10.18(7.54-13.43) | 48(36-63) | 9.14(6.84-12.14) | -0.3(-0.76 to 0.16) | -2.59(-3.59 to -1.57) | 0.45(-0.22 to 1.12) | 0.94(0.3 to 1.58) |
| Russian Federation | 3,003(2,439-3,922) | 8.65(7.03-11.3) | 1,962(1,544-2,655) | 7.52(5.92-10.18) | -0.45(-1.33 to 0.45) | 0.71(-2.09 to 3.59) | 0.51(0.21 to 0.8) | -2.32(-3.22 to -1.4) |
| Ukraine | 392(206-699) | 3.45(1.81-6.14) | 325(222-489) | 5.11(3.49-7.71) | 1.3(0.57 to 2.03) | 0.09(-1.15 to 1.34) | 2.02(1.48 to 2.57) | 1.57(-0.08 to 3.24) |
| Brunei Darussalam | 5(3-9) | 5.99(3.32-9.39) | 10(7-14) | 10.12(7.06-14.34) | 1.74(1.57 to 1.91) | 3.61(3.45 to 3.77) | 2.03(1.7 to 2.36) | -0.28(-0.62 to 0.06) |
| Japan | 810(586-1,159) | 3.51(2.54-5.02) | 672(514-903) | 4.35(3.33-5.84) | 0.64(0.18 to 1.1) | 0.31(0.09 to 0.53) | 1.01(0.61 to 1.41) | 0.54(-0.64 to 1.74) |
| Republic of Korea | 382(259-587) | 3.36(2.28-5.16) | 184(122-277) | 3.02(2-4.56) | -0.32(-0.49 to -0.15) | 1.03(0.85 to 1.21) | -0.58(-0.82 to -0.33) | -1.26(-1.66 to -0.87) |
| Singapore | 56(48-68) | 8.66(7.38-10.41) | 62(50-78) | 7.58(6.21-9.6) | -0.18(-1.38 to 1.04) | -0.64(-2.75 to 1.52) | 0.2(-1.02 to 1.43) | -0.11(-2.61 to 2.46) |
| Australia | 244(153-389) | 6.46(4.05-10.27) | 319(208-504) | 6.71(4.38-10.61) | 0.24(0 to 0.48) | 0.85(0.74 to 0.96) | 0.85(0.74 to 0.96) | -0.76(-1.39 to -0.13) |
| New Zealand | 51(32-80) | 6.37(4.01-10.01) | 58(35-94) | 5.95(3.61-9.56) | -0.24(-0.35 to -0.13) | -1.21(-1.45 to -0.98) | 0.7(0.56 to 0.84) | -0.37(-0.56 to -0.17) |
| Andorra | 0(0-0) | 2.18(1.13-4.07) | 0(0-0) | 2.15(1.11-3.94) | -0.08(-0.13 to -0.04) | 0.48(0.39 to 0.57) | 0(-0.05 to 0.05) | -0.61(-0.67 to -0.56) |
| Austria | 37(20-65) | 2.75(1.48-4.79) | 37(20-64) | 2.85(1.56-4.97) | 0.11(0.05 to 0.17) | 0.03(-0.05 to 0.11) | 0.09(0.07 to 0.12) | 0.22(0.07 to 0.38) |
| Belgium | 58(39-90) | 3.22(2.15-4.96) | 70(48-101) | 3.67(2.53-5.28) | 0.32(-0.05 to 0.7) | 0.52(0.42 to 0.61) | 0.52(0.42 to 0.61) | -0.03(-1.06 to 1.01) |
| Cyprus | 6(3-10) | 2.89(1.57-5.17) | 6(3-11) | 2.74(1.42-4.97) | -0.17(-0.32 to -0.01) | -0.37(-0.55 to -0.2) | 0.97(0.8 to 1.14) | -1.06(-1.4 to -0.71) |
| Denmark | 29(17-51) | 3.29(1.90-5.79) | 32(18-55) | 3.35(1.92-5.73) | 0.04(-0.1 to 0.19) | 0.37(0.2 to 0.54) | -0.49(-0.79 to -0.19) | 0.26(0 to 0.52) |
| Finland | 25(13-47) | 2.59(1.36-4.9) | 22(12-41) | 2.61(1.38-4.86) | 0.02(-0.09 to 0.12) | 0.13(-0.18 to 0.43) | 0.27(0.16 to 0.37) | -0.29(-0.38 to -0.2) |
| France | 354(226-583) | 3.03(1.93-4.98) | 387(261-596) | 3.33(2.24-5.13) | 0.29(0.11 to 0.47) | 0.83(0.57 to 1.09) | 0.45(0.3 to 0.6) | -0.43(-0.78 to -0.07) |
| Germany | 474(278-807) | 3.66(2.15-6.23) | 468(281-774) | 3.91(2.35-6.47) | 0.2(0.16 to 0.24) | -0.18(-0.21 to -0.14) | 0.29(0.23 to 0.34) | 0.41(0.33 to 0.5) |
| Greece | 36(17-67) | 1.78(0.82-3.32) | 26(13-51) | 1.88(0.94-3.62) | 0.17(0.13 to 0.21) | 0.29(0.18 to 0.39) | 0.5(0.46 to 0.54) | -0.26(-0.32 to -0.2) |
| Iceland | 2(1-3) | 2.76(1.66-4.52) | 2(1-3) | 2.92(1.79-4.69) | 0.19(0.09 to 0.28) | 0.35(0.26 to 0.43) | 0.12(0.01 to 0.22) | 0.08(-0.07 to 0.23) |
| Ireland | 27(16-44) | 2.71(1.66-4.47) | 27(17-46) | 2.73(1.65-4.57) | 0.03(-0.18 to 0.24) | 1.41(1 to 1.84) | -0.08(-0.32 to 0.16) | -1.15(-1.57 to -0.71) |
| Israel | 92(74-122) | 5.97(4.8-7.96) | 138(106-186) | 5.24(4.04-7.1) | -0.47(-0.78 to -0.15) | 0.13(-0.3 to 0.57) | -0.45(-0.69 to -0.2) | -1.13(-1.85 to -0.42) |
| Italy | 227(161-337) | 2.45(1.74-3.65) | 196(141-282) | 2.57(1.86-3.71) | 0.15(-0.17 to 0.47) | -0.31(-0.85 to 0.24) | 1.04(0.79 to 1.3) | -0.24(-0.93 to 0.46) |
| Luxembourg | 2(1-3) | 2.4(1.34-4.23) | 2(1-4) | 2.39(1.39-4.05) | -0.08(-0.28 to 0.12) | 0.13(-0.13 to 0.4) | -0.61(-0.78 to -0.43) | 0.27(-0.21 to 0.75) |
| Malta | 3(2-4) | 3.29(2.31-5.12) | 4(3-5) | 5.62(4.32-7.48) | 1.72(1.18 to 2.26) | 1.1(0.24 to 1.97) | 2.53(1.94 to 3.13) | 1.8(0.83 to 2.78) |
| Netherlands | 93(62-147) | 3.4(2.27-5.4) | 104(74-151) | 3.88(2.78-5.64) | 0.44(0.12 to 0.75) | 1.04(0.78 to 1.3) | -0.48(-0.93 to -0.03) | 0.65(-0.03 to 1.33) |
| Norway | 17(8-32) | 2.12(1.03-4.01) | 20(10-37) | 2.13(1.05-4.03) | 0(-0.09 to 0.08) | 0.13(0.05 to 0.21) | -0.18(-0.4 to 0.04) | 0(-0.13 to 0.14) |
| Portugal | 53(33-93) | 2.52(1.54-4.39) | 38(24-60) | 2.78(1.73-4.44) | 0.32(0.05 to 0.59) | 1.25(1.13 to 1.36) | -0.52(-0.96 to -0.08) | 0.18(-0.45 to 0.81) |
| Spain | 238(154-366) | 3.04(1.97-4.67) | 222(148-336) | 3.42(2.29-5.18) | 0.39(0.2 to 0.59) | 1.31(1.17 to 1.44) | 0.78(0.37 to 1.2) | -0.55(-0.92 to -0.19) |
| Sweden | 48(30-75) | 3.08(1.96-4.89) | 51(32-85) | 2.8(1.74-4.66) | -0.32(-0.56 to -0.08) | -1.86(-2.21 to -1.51) | 0.87(0.56 to 1.19) | -0.29(-0.72 to 0.15) |
| Switzerland | 31(18-54) | 2.73(1.59-4.64) | 37(21-62) | 2.74(1.61-4.68) | 0.02(-0.1 to 0.14) | -0.02(-0.06 to 0.03) | -0.02(-0.06 to 0.03) | 0.08(-0.25 to 0.41) |
| United Kingdom | 348(220-547) | 3.18(2.01-5.01) | 419(282-630) | 3.55(2.39-5.35) | 0.44(0.13 to 0.75) | 0.47(-0.28 to 1.24) | 2.35(1.9 to 2.8) | -1.22(-1.5 to -0.94) |
| Argentina | 619(438-878) | 6.11(4.32-8.66) | 1,115(925-1,442) | 10.95(9.08-14.16) | 1.83(0.8 to 2.86) | 3.39(1.85 to 4.94) | 1.41(-1.04 to 3.92) | 0.64(-0.63 to 1.92) |
| Chile | 161(114-237) | 4.06(2.87-5.98) | 215(170-283) | 5.89(4.66-7.76) | 1.22(0.48 to 1.96) | 3.17(1.01 to 5.38) | 0.27(-0.14 to 0.67) | 0.59(-0.45 to 1.65) |
| Uruguay | 42(31-62) | 5.19(3.84-7.54) | 53(41-69) | 7.97(6.29-10.49) | 1.42(0.52 to 2.32) | 4.18(3.69 to 4.67) | 0.17(-2.45 to 2.86) | -0.03(-1.06 to 1) |
| Canada | 305(209-428) | 5.3(3.63-7.44) | 327(219-469) | 5.3(3.54-7.59) | 0.05(-0.15 to 0.25) | -0.09(-0.37 to 0.19) | 0.71(0.51 to 0.9) | -0.51(-0.95 to -0.07) |
| United States of America | 3,995(2,978-5,328) | 7.14(5.33-9.53) | 4,431(3,374-5,888) | 7.46(5.68-9.91) | 0.2(-0.14 to 0.54) | -1.3(-1.85 to -0.75) | 1.48(1.21 to 1.76) | 0.11(-0.52 to 0.74) |
| Antigua and Barbuda | 2(1-2) | 10.56(8.21-13.16) | 7(5-8) | 38.63(31.98-46.33) | 4.38(2.59 to 6.19) | 15.89(9.94 to 22.16) | -0.62(-1.56 to 0.33) | 0.63(-1.4 to 2.7) |
| Bahamas | 9(7-11) | 11.07(8.74-14.05) | 20(15-26) | 24.3(18.3-32.21) | 2.44(1.85 to 3.02) | 11.78(9.86 to 13.74) | -1.16(-1.51 to -0.81) | -1.16(-1.51 to -0.81) |
| Barbados | 21(17-26) | 33.42(26.93-41.54) | 32(23-44) | 67.68(48.58-92.6) | 2.29(0.3 to 4.31) | 11.23(4.1 to 18.84) | -1.16(-1.68 to -0.64) | -1.16(-1.68 to -0.64) |
| Belize | 7(5-11) | 8.93(5.84-13.08) | 18(15-23) | 14.99(12.32-18.56) | 1.93(1 to 2.87) | 13.05(11.63 to 14.48) | -3.42(-4.49 to -2.33) | -1.27(-3.21 to 0.72) |
| Cuba | 140(105-187) | 5.6(4.2-7.45) | 137(113-172) | 7.71(6.35-9.67) | 1.18(0.27 to 2.1) | 6.31(4.5 to 8.15) | -1.87(-2.58 to -1.16) | 0.2(-1.76 to 2.19) |
| Dominica | 6(3-8) | 23.38(12.74-34.14) | 7(5-11) | 54.55(34.11-81.57) | 2.78(2.4 to 3.17) | 7.83(7.49 to 8.17) | 1.87(1.13 to 2.6) | -0.78(-1.44 to -0.12) |
| Dominican Republic | 139(98-200) | 5.15(3.62-7.42) | 100(63-156) | 3.4(2.13-5.31) | -1.37(-1.74 to -1) | 3(2.74 to 3.26) | -3.02(-3.55 to -2.49) | -3.49(-4.3 to -2.68) |
| Grenada | 6(3-9) | 16.67(9.36-27.35) | 12(10-15) | 56.32(45.85-68.3) | 4.33(2.36 to 6.33) | 18(10.82 to 25.64) | -1.16(-1.69 to -0.62) | -0.44(-1.87 to 1.01) |
| Guyana | 15(11-20) | 5.11(3.81-6.87) | 33(25-43) | 15.31(11.68-20.1) | 3.7(2.58 to 4.83) | 13.35(9.68 to 17.13) | -0.61(-1.06 to -0.16) | 0.1(-1.11 to 1.33) |
| Haiti | 2,748(849-5,461) | 101.28(31.29-201.3) | 3,822(1,582-7,168) | 87.81(36.35-164.68) | -0.45(-0.6 to -0.29) | -1.99(-2.4 to -1.59) | 1.23(1.11 to 1.35) | -0.62(-0.8 to -0.44) |
| Jamaica | 129(99-163) | 15.45(11.9-19.51) | 157(121-211) | 26.93(20.66-36.13) | 2.15(1.3 to 3) | 9.85(6.91 to 12.87) | -1.83(-2.29 to -1.36) | 0.12(-0.22 to 0.46) |
| Saint Lucia | 8(6-10) | 15.24(11.64-20.3) | 12(9-15) | 38.86(29.95-50.82) | 3.6(2.91 to 4.3) | 13.62(11.29 to 15.99) | -0.24(-0.76 to 0.29) | -0.24(-0.76 to 0.29) |
| Saint Vincent and the Grenadines | 3(3-4) | 8.32(6.71-10.35) | 11(8-13) | 42.51(33.84-53.85) | 5.45(3.78 to 7.14) | 24.6(19.92 to 29.45) | -2.98(-4.07 to -1.87) | 0.51(-1.85 to 2.93) |
| Suriname | 68(40-104) | 52.53(30.77-79.62) | 84(49-130) | 58.73(34.11-91.05) | 0.33(-0.19 to 0.84) | 6.65(5.96 to 7.34) | -0.58(-1.08 to -0.08) | -3.74(-4.87 to -2.59) |
| Trinidad and Tobago | 45(38-56) | 11.19(9.37-13.84) | 50(39-65) | 18.27(14.26-23.81) | 1.72(0.85 to 2.61) | 11.56(8.35 to 14.88) | -2.05(-2.27 to -1.83) | -2.05(-2.27 to -1.83) |
| Bolivia (Plurinational State of) | 365(176-592) | 13.6(6.56-22.02) | 301(208-411) | 8.64(5.96-11.78) | -1.44(-1.53 to -1.36) | -0.65(-0.73 to -0.56) | -1.46(-1.7 to -1.22) | -2.12(-2.22 to -2.02) |
| Ecuador | 108(74-163) | 2.79(1.91-4.22) | 720(560-933) | 14.19(11.05-18.4) | 5.29(1.75 to 8.96) | 14.43(7.16 to 22.19) | -14.58(-16.76 to-12.34) | 11.41(5.52 to 17.63) |
| Peru | 1,163(671-1,732) | 14.01(8.09-20.87) | 741(505-1,085) | 7.77(5.3-11.37) | -1.82(-2.43 to -1.21) | 6.79(5.29 to 8.32) | -12.29(-12.79 to-11.79) | -0.34(-1.42 to 0.76) |
| Colombia | 2,710(2,349-3,138) | 23.24(20.14-26.9) | 1,922(1,431-2,536) | 18.11(13.48-23.9) | -0.73(-2.23 to 0.8) | -3.12(-4.5 to -1.71) | -0.43(-3.8 to 3.06) | 0.32(-2.52 to 3.23) |
| Costa Rica | 71(59-86) | 6.32(5.29-7.67) | 70(58-86) | 6.87(5.75-8.44) | 0.25(-0.75 to 1.25) | -1.97(-4.2 to 0.31) | 0.22(-0.56 to 1.01) | 2.17(0.37 to 4) |
| El Salvador | 373(246-522) | 17.27(11.41-24.19) | 137(98-195) | 7.51(5.38-10.7) | -2.75(-3.32 to -2.17) | -2.78(-3.43 to -2.13) | -2.35(-3.46 to -1.22) | -2.92(-4.03 to -1.8) |
| Guatemala | 695(470-1,036) | 17.1(11.58-25.5) | 986(767-1,289) | 19.99(15.54-26.12) | 0.52(-0.77 to 1.83) | 0.45(-2.38 to 3.37) | -0.92(-1.19 to -0.65) | 2.04(-0.75 to 4.92) |
| Honduras | 128(66-196) | 5.78(2.97-8.86) | 105(60-175) | 3.21(1.83-5.35) | -1.91(-2.02 to -1.8) | -2.77(-2.99 to -2.55) | -1.56(-1.64 to -1.49) | -1.4(-1.6 to -1.21) |
| Mexico | 6,864(6,229-7,704) | 20.54(18.64-23.05) | 3,962(3,214-4,958) | 12.36(10.02-15.46) | -1.23(-1.9 to -0.55) | -6.96(-7.4 to -6.52) | 2.29(0.25 to 4.37) | 1.13(0.33 to 1.94) |
| Nicaragua | 253(155-365) | 13.88(8.48-20.02) | 142(100-205) | 7.15(5.07-10.33) | -2.15(-2.41 to -1.9) | -1.42(-1.64 to -1.19) | -1.35(-1.6 to -1.11) | -3.35(-3.98 to -2.71) |
| Panama | 93(72-116) | 11.2(8.65-13.87) | 186(148-233) | 16.12(12.8-20.18) | 1.26(0.67 to 1.85) | 0.9(0.58 to 1.22) | 1.56(-0.01 to 3.16) | 1.33(0.42 to 2.24) |
| Venezuela (Bolivarian Republic of) | 227(169-318) | 3.2(2.38-4.48) | 220(162-301) | 3.33(2.45-4.55) | 0.15(-0.81 to 1.13) | 0.13(-2.39 to 2.72) | -0.11(-1.48 to 1.28) | 0.76(-0.26 to 1.78) |
| Brazil | 12,820(10,685-15,617) | 24.68(20.57-30.06) | 10,857(8,944-13,033) | 22.53(18.56-27.05) | -0.08(-1.15 to 1) | 3.7(3.07 to 4.34) | -1.86(-4.68 to 1.05) | -1.99(-3.62 to -0.34) |
| Paraguay | 179(102-262) | 10.7(6.12-15.72) | 187(129-270) | 9.33(6.42-13.43) | -0.44(-1.03 to 0.16) | -0.5(-0.99 to -0.02) | 0.79(-0.52 to 2.12) | -1.41(-2.6 to -0.2) |
| Algeria | 663(401-1,050) | 6.18(3.74-9.79) | 541(322-895) | 4.07(2.42-6.73) | -1.33(-1.4 to -1.26) | -2.17(-2.23 to -2.1) | -0.35(-0.45 to -0.25) | -1.57(-1.71 to -1.42) |
| Bahrain | 65(47-84) | 39.81(29.06-51.29) | 44(35-55) | 14.78(11.87-18.59) | -3.22(-3.85 to -2.59) | -3.61(-4.09 to -3.13) | -3.27(-5.2 to -1.3) | -3.15(-3.71 to -2.58) |
| Egypt | 1,083(593-1,868) | 4.88(2.67-8.42) | 1,804(1,062-3,124) | 4.89(2.88-8.48) | 0.03(-0.22 to 0.28) | -0.15(-0.51 to 0.21) | 2.04(1.42 to 2.67) | -1.67(-1.99 to -1.35) |
| Iran (Islamic Republic of) | 1,534(993-2,302) | 6.04(3.91-9.07) | 732(430-1,179) | 3.63(2.13-5.84) | -1.55(-1.86 to -1.25) | -2.17(-2.51 to -1.82) | 2.59(2.18 to 3) | -5.09(-5.72 to -4.45) |
| Iraq | 276(157-474) | 3.35(1.91-5.75) | 385(211-662) | 2.86(1.57-4.92) | -0.49(-0.56 to -0.43) | -0.4(-0.48 to -0.32) | 0.88(0.73 to 1.03) | -1.72(-1.83 to -1.61) |
| Jordan | 98(67-147) | 6.02(4.1-9.01) | 138(83-229) | 3.79(2.28-6.3) | -1.53(-1.7 to -1.36) | -0.37(-0.55 to -0.19) | -1.63(-2.03 to -1.22) | -2.36(-2.64 to -2.08) |
| Kuwait | 17(9-31) | 3.12(1.63-5.68) | 31(18-54) | 3.67(2.12-6.35) | 0.51(-0.33 to 1.35) | 2.37(0.24 to 4.54) | -0.91(-1.21 to -0.6) | 0.42(-1.15 to 2.02) |
| Lebanon | 75(45-117) | 7.2(4.3-11.17) | 58(37-97) | 4.57(2.91-7.59) | -1.46(-1.54 to -1.38) | -1.48(-1.54 to -1.42) | -1.68(-1.81 to -1.54) | -1.37(-1.55 to -1.19) |
| Libya | 98(61-155) | 5.39(3.38-8.55) | 58(34-97) | 3.92(2.3-6.5) | -1.03(-1.2 to -0.85) | -1.96(-2.43 to -1.48) | -0.63(-0.77 to -0.49) | -0.67(-0.9 to -0.43) |
| Morocco | 834(451-1,369) | 8.52(4.61-13.99) | 431(243-716) | 4.4(2.48-7.31) | -2.1(-2.22 to -1.98) | -1.56(-1.65 to -1.47) | -2.03(-2.14 to -1.93) | -2.56(-2.85 to -2.26) |
| Palestine | 42(25-69) | 4.35(2.53-7.16) | 68(39-119) | 3.65(2.08-6.4) | -0.54(-0.75 to -0.34) | -0.93(-1.08 to -0.78) | 0.61(0.38 to 0.83) | -1.34(-1.84 to -0.84) |
| Oman | 122(76-179) | 14.5(9.07-21.3) | 86(63-124) | 7.04(5.15-10.18) | -2.18(-2.5 to -1.86) | -5.89(-6.27 to -5.52) | 1.28(0.46 to 2.11) | -2.36(-2.83 to -1.88) |
| Qatar | 9(6-13) | 6.82(4.82-10.02) | 28(19-42) | 5.69(3.87-8.59) | -0.57(-0.81 to -0.33) | 0.12(-0.25 to 0.49) | 0.49(0.07 to 0.92) | -2.44(-2.88 to -1.99) |
| Saudi Arabia | 1,006(525-1,654) | 15.35(8.01-25.24) | 263(141-479) | 3.47(1.87-6.33) | -4.72(-4.88 to -4.56) | -3.31(-3.81 to -2.81) | -6.77(-6.9 to -6.64) | -3.78(-3.96 to -3.6) |
| Syrian Arab Republic | 171(84-332) | 2.89(1.42-5.6) | 102(48-195) | 2.78(1.3-5.33) | -0.14(-0.2 to -0.08) | 0.01(-0.11 to 0.14) | -0.1(-0.19 to -0.02) | -0.28(-0.36 to -0.21) |
| Tunisia | 190(115-301) | 6.13(3.7-9.71) | 105(58-188) | 3.8(2.09-6.78) | -1.53(-1.58 to -1.48) | -2.89(-2.99 to -2.8) | -0.66(-0.72 to -0.61) | -1.26(-1.34 to -1.18) |
| Turkey | 825(423-1,471) | 4.03(2.07-7.18) | 674(344-1,265) | 3.64(1.86-6.83) | -0.3(-0.42 to -0.18) | -0.55(-0.61 to -0.49) | 0.64(0.42 to 0.85) | -0.89(-1.13 to -0.65) |
| United Arab Emirates | 26(15-44) | 4.42(2.53-7.38) | 49(27-85) | 3.63(2.05-6.35) | -0.67(-0.86 to -0.49) | -1.37(-1.56 to -1.18) | 0.69(0.36 to 1.02) | -1.54(-1.92 to -1.16) |
| Yemen | 704(348-1,151) | 9.92(4.9-16.22) | 734(423-1,144) | 5.33(3.07-8.3) | -2(-2.1 to -1.9) | -0.95(-1.04 to -0.87) | -2.4(-2.6 to -2.2) | -2.43(-2.57 to -2.29) |
| Afghanistan | 569(265-1,066) | 13.21(6.14-24.75) | 1,076(579-1,731) | 7.58(4.08-12.19) | -1.81(-2.12 to -1.49) | -1.55(-1.74 to -1.37) | -2.55(-3.06 to -2.03) | -1.25(-1.94 to -0.55) |
| Bangladesh | 14,956(7,224-23,731) | 30.58(14.77-48.52) | 4,653(2,715-7,434) | 10.17(5.93-16.24) | -3.57(-4.01 to -3.14) | -4.49(-5.2 to -3.78) | -3.99(-4.2 to -3.77) | -2.1(-3.12 to -1.08) |
| Bhutan | 179(95-272) | 68.15(36.17-103.66) | 52(26-88) | 27.61(14.01-46.81) | -3.04(-3.44 to -2.65) | -3.45(-3.69 to -3.21) | -3.45(-3.69 to -3.21) | -2.3(-3.31 to -1.29) |
| India | 358,133(265,039-463,254) | 109.68(81.17-141.88) | 147,733(85,501-229,415) | 40.32(23.34-62.61) | -3.25(-3.57 to -2.94) | -0.51(-0.65 to -0.36) | -3.38(-3.64 to -3.13) | -5.39(-6.21 to -4.55) |
| Nepal | 494(271-911) | 5.86(3.22-10.81) | 2,004(1,191-3,007) | 21.72(12.91-32.59) | 4.32(4.03 to 4.61) | -0.77(-0.96 to -0.58) | 3.62(3.08 to 4.15) | 9(8.46 to 9.56) |
| Pakistan | 16,906(10,903-25,358) | 34.33(22.14-51.5) | 12,906(8,240-18,821) | 15.11(9.64-22.03) | -2.66(-2.98 to -2.34) | -4.09(-4.52 to -3.66) | -2.14(-2.55 to -1.73) | -1.97(-2.71 to -1.22) |
| Angola | 9,383(5,781-13,531) | 199.01(122.61-286.99) | 10,712(4,759-20,611) | 70.26(31.21-135.19) | -3.37(-3.61 to -3.13) | -1.19(-1.33 to -1.05) | -0.83(-1.1 to -0.56) | -7.28(-7.77 to -6.79) |
| Central African Republic | 1,836(1,126-2,726) | 150.13(92.07-222.99) | 1,856(832-3,631) | 81.26(36.41-158.97) | -1.95(-2.15 to -1.75) | -1.7(-1.95 to -1.46) | -1.86(-2.48 to -1.24) | -2.19(-2.28 to -2.1) |
| Congo | 952(490-1,532) | 90.39(46.5-145.46) | 637(215-1,413) | 33.03(11.16-73.27) | -3.28(-3.54 to -3.01) | -0.91(-1.2 to -0.61) | -2.36(-2.99 to -1.73) | -5.83(-6.21 to -5.44) |
| Democratic Republic of the Congo | 18,357(10,076-29,565) | 103.69(56.91-167.00) | 14,275(5,162-29,120) | 37.57(13.58-76.63) | -3.23(-3.36 to -3.1) | -1.47(-1.61 to -1.32) | -2.9(-3.25 to -2.55) | -5.21(-5.36 to -5.07) |
| Equatorial Guinea | 236(145-345) | 119.62(73.66-175.15) | 195(80-427) | 33.29(13.72-73) | -3.94(-4.47 to -3.41) | -2.3(-2.87 to -1.72) | -5.12(-6.34 to -3.89) | -4.29(-5.19 to -3.38) |
| Gabon | 264(118-481) | 64.73(28.94-118.09) | 182(68-436) | 28.42(10.57-68.18) | -2.64(-2.93 to -2.35) | -2.6(-2.77 to -2.42) | -0.52(-0.97 to -0.06) | -4.19(-4.81 to -3.57) |
| Burundi | 3,132(1,763-4,753) | 119.47(67.26-181.33) | 2,935(1,079-5,126) | 50.13(18.44-87.56) | -2.83(-3.06 to -2.6) | -2.86(-3.08 to -2.63) | 0.73(0.25 to 1.22) | -5.74(-6.1 to -5.37) |
| Comoros | 252(140-380) | 118.28(65.93-178.73) | 131(64-226) | 54.56(26.61-94.15) | -2.57(-3.08 to -2.06) | -2.45(-2.65 to -2.24) | -1.49(-2.49 to -0.48) | -3.27(-3.79 to -2.74) |
| Djibouti | 168(107-243) | 96.75(61.7-139.68) | 187(96-330) | 45.27(23.22-79.97) | -2.34(-2.68 to -1.99) | -0.75(-1.32 to -0.16) | -1.04(-1.58 to -0.5) | -4.51(-5.16 to -3.85) |
| Eritrea | 1,628(987-2,417) | 102.28(61.98-151.85) | 1,614(785-2,829) | 63.91(31.08-112.07) | -1.47(-1.74 to -1.19) | -2(-2.6 to -1.39) | -1.13(-1.32 to -0.93) | -1.3(-1.79 to -0.8) |
| Ethiopia | 31,586(12,222-52,754) | 129.64(50.17-216.53) | 23,222(15,259-34,570) | 52.36(34.41-77.95) | -2.9(-3.21 to -2.59) | -2.97(-3.44 to -2.49) | -4.02(-4.34 to -3.71) | -1.72(-2.39 to -1.05) |
| Kenya | 8,858(6,362-11,654) | 79.3(56.95-104.33) | 6,471(3,817-10,835) | 34.67(20.45-58.05) | -2.54(-2.66 to -2.41) | -2.69(-2.91 to -2.47) | -1.03(-1.18 to -0.87) | -3.91(-4.15 to -3.66) |
| Madagascar | 6,911(4,002-10,362) | 126.67(73.35-189.93) | 6,792(3,411-11,957) | 57.88(29.07-101.9) | -2.49(-2.69 to -2.28) | -2.24(-2.49 to -1.98) | -1.54(-2.01 to -1.06) | -3.44(-3.76 to -3.13) |
| Malawi | 9,251(4,390-15,214) | 203.33(96.5-334.41) | 5,233(2,519-8,475) | 64.42(31-104.33) | -3.63(-4.11 to -3.16) | -2.87(-3.16 to -2.57) | -4.08(-4.85 to -3.31) | -4.27(-5.23 to -3.3) |
| Mauritius | 7(6-9) | 2.11(1.72-2.68) | 20(16-23) | 9.41(7.88-10.9) | 5(2.18 to 7.9) | 7.77(3.46 to 12.26) | 6.01(0.67 to 11.62) | 2.65(-2.6 to 8.18) |
| Mozambique | 7,504(4,259-11,244) | 120.95(68.65-181.23) | 7,255(3,997-12,184) | 50.86(28.02-85.41) | -2.68(-3.01 to -2.34) | -0.52(-0.82 to -0.23) | -2.35(-3.23 to -1.45) | -4.59(-4.95 to -4.23) |
| Rwanda | 3,914(2,171-6,198) | 115.36(63.98-182.66) | 2,495(1,185-4,606) | 50.19(23.85-92.66) | -2.56(-3.29 to -1.82) | -1.71(-3.57 to 0.2) | -2.74(-4.33 to -1.12) | -3.28(-3.65 to -2.91) |
| Seychelles | 1(1-1) | 2.99(2.15-3.86) | 1(1-1) | 4.23(3.28-5.62) | 1.06(0.39 to 1.73) | 0.42(-0.26 to 1.1) | 5.55(3.84 to 7.29) | -1.99(-3.02 to -0.94) |
| Somalia | 5,135(2,630-8,110) | 131.8(67.52-208.17) | 7,579(4,300-12,973) | 73.38(41.63-125.6) | -1.84(-2.23 to -1.45) | -1.17(-1.36 to -0.97) | -0.91(-1.9 to 0.09) | -3.27(-3.77 to -2.77) |
| United Republic of Tanzania | 18,602(10,159-29,150) | 154.04(84.13-241.4) | 17,049(8,427-27,797) | 69.86(34.53-113.91) | -2.54(-2.73 to -2.35) | -2.56(-2.78 to -2.34) | -1.07(-1.41 to -0.73) | -3.7(-4.09 to -3.32) |
| Uganda | 10,101(5,687-15,712) | 119.97(67.54-186.61) | 10,578(4,443-18,699) | 53.33(22.4-94.27) | -2.61(-3.41 to -1.81) | -2.65(-2.81 to -2.5) | -2.65(-2.81 to -2.5) | -2.71(-4.91 to -0.47) |
| Zambia | 6,528(3,578-9,714) | 173.88(95.30-258.72) | 6,467(2,390-13,459) | 78.18(28.89-162.71) | -2.51(-2.9 to -2.11) | -1.28(-1.81 to -0.75) | 0.19(-0.76 to 1.14) | -5.58(-6.2 to -4.96) |
| Botswana | 98(49-174) | 16.54(8.37-29.47) | 103(53-168) | 14.72(7.59-24) | -0.38(-0.45 to -0.31) | 1(0.81 to 1.2) | -0.89(-0.93 to -0.85) | -1.17(-1.2 to -1.13) |
| Lesotho | 144(74-239) | 21.14(10.84-35.07) | 128(61-221) | 20.33(9.75-35.08) | -0.12(-0.27 to 0.03) | 0.53(0.36 to 0.71) | -0.22(-0.5 to 0.05) | -0.6(-0.79 to -0.42) |
| Namibia | 110(57-178) | 18.36(9.48-29.55) | 127(67-221) | 15.37(8.06-26.81) | -0.56(-0.74 to -0.38) | 0.08(-0.42 to 0.58) | -0.22(-0.48 to 0.04) | -1.58(-1.67 to -1.48) |
| South Africa | 3,749(2,647-4,758) | 27.54(19.45-34.95) | 2,674(1,915-3,810) | 17.59(12.59-25.06) | -1.4(-1.74 to -1.05) | 1.61(1.43 to 1.78) | -1.91(-2.67 to -1.13) | -3.41(-4.1 to -2.71) |
| Eswatini | 91(50-146) | 23.51(12.90-37.78) | 88(46-152) | 21.24(11.24-36.85) | -0.33(-0.43 to -0.22) | 1.9(1.66 to 2.14) | -0.14(-0.29 to 0) | -2.36(-2.53 to -2.2) |
| Zimbabwe | 2,490(991-4,288) | 51.69(20.57-89.04) | 2,330(914-4,280) | 37.02(14.53-68.01) | -1.22(-1.62 to -0.82) | -0.16(-1.12 to 0.81) | 0.37(-0.38 to 1.12) | -2.85(-3.22 to -2.48) |
| Benin | 326(217-495) | 13.44(8.96-20.45) | 1,222(679-1,973) | 20.09(11.17-32.45) | 1.32(1.06 to 1.59) | 5.86(5.25 to 6.47) | 0.46(0.21 to 0.72) | -2.18(-2.37 to -2) |
| Burkina Faso | 3,293(2,378-4,572) | 69.79(50.4-96.88) | 5,752(2,897-11,089) | 55.46(27.93-106.91) | -0.7(-0.99 to -0.42) | 0.22(-0.09 to 0.53) | -1.36(-1.63 to -1.09) | -1(-1.67 to -0.33) |
| Cameroon | 580(375-894) | 11.88(7.68-18.32) | 2,220(1,337-3,452) | 16.48(9.93-25.63) | 1.09(0.95 to 1.23) | 5.21(4.91 to 5.51) | 0.9(0.76 to 1.05) | -2.68(-2.84 to -2.52) |
| Cabo Verde | 11(6-20) | 7.02(3.56-13.03) | 13(7-22) | 8.76(4.66-15.35) | 0.75(0.58 to 0.91) | 3.58(3.16 to 4.01) | 0.21(0.05 to 0.37) | -1.55(-1.73 to -1.38) |
| Chad | 395(256-607) | 13.5(8.75-20.75) | 2,117(1,337-3,153) | 23.48(14.83-34.98) | 1.84(1.53 to 2.14) | 6.14(5.2 to 7.08) | 0.35(0.17 to 0.54) | -0.88(-0.93 to -0.84) |
| C??te d'Ivoire | 699(440-1,078) | 12.25(7.72-18.91) | 1,985(1,194-2,932) | 17.15(10.32-25.33) | 1.11(0.93 to 1.29) | 5.96(5.58 to 6.34) | -0.23(-0.37 to -0.09) | -2(-2.35 to -1.64) |
| Gambia | 50(32-78) | 10.84(6.85-16.84) | 135(74-222) | 13.6(7.45-22.38) | 0.8(0.57 to 1.02) | 5.52(5.11 to 5.93) | -0.64(-1.15 to -0.13) | -1.96(-2.18 to -1.74) |
| Ghana | 3,056(1,988-4,367) | 45.5(29.59-65.01) | 6,347(2,886-10,984) | 49.27(22.4-85.26) | 0.24(-0.16 to 0.64) | -0.7(-0.93 to -0.47) | 7.41(6.57 to 8.26) | -3.93(-4.56 to -3.3) |
| Guinea | 490(309-739) | 17.81(11.24-26.84) | 1,426(871-2,123) | 23.59(14.41-35.11) | 0.92(0.73 to 1.12) | 6.76(6.14 to 7.39) | -0.46(-0.66 to -0.26) | -2.89(-3.06 to -2.72) |
| Guinea-Bissau | 68(47-102) | 14.06(9.65-21.06) | 161(94-255) | 17.95(10.46-28.35) | 0.85(0.44 to 1.27) | 6.66(5.71 to 7.63) | 0.12(-0.41 to 0.66) | -3.59(-3.79 to -3.39) |
| Liberia | 172(111-254) | 15.2(9.83-22.48) | 368(215-584) | 16.84(9.85-26.7) | 0.3(-0.05 to 0.65) | 5.87(4.86 to 6.89) | -2.28(-2.6 to -1.95) | -2.76(-3.05 to -2.46) |
| Mali | 620(392-947) | 15.02(9.49-22.93) | 2,576(1,601-3,718) | 22.25(13.83-32.12) | 1.32(1.09 to 1.55) | 6.87(6.54 to 7.2) | -0.41(-0.92 to 0.11) | -1.99(-2.33 to -1.64) |
| Mauritania | 103(67-167) | 11.19(7.21-18.04) | 257(152-404) | 13.86(8.2-21.78) | 0.69(0.5 to 0.88) | 5.81(5.39 to 6.23) | -0.24(-0.64 to 0.17) | -2.96(-3.07 to -2.85) |
| Niger | 608(396-882) | 14.97(9.74-21.71) | 2,687(1,531-4,468) | 21.05(11.99-35) | 1.11(0.82 to 1.41) | 7.16(6.75 to 7.57) | -2.15(-2.78 to -1.52) | -1.11(-1.57 to -0.64) |
| Nigeria | 5,395(3,834-7,740) | 13.79(9.8-19.78) | 19,440(13,387-26,532) | 19.14(13.18-26.12) | 1.09(0.9 to 1.28) | 7.3(6.73 to 7.86) | -0.23(-0.37 to -0.09) | -3.25(-3.44 to -3.05) |
| Sao Tome and Principe | 7(4-12) | 13.12(7.09-21.32) | 6(3-11) | 7.98(4-14.66) | -1.58(-1.71 to -1.45) | -0.22(-0.44 to 0) | -1.92(-2.03 to -1.82) | -2.29(-2.55 to -2.02) |
| Senegal | 459(306-689) | 12.58(8.38-18.87) | 1,027(578-1,669) | 16.15(9.08-26.23) | 0.84(0.42 to 1.25) | 6.14(5.48 to 6.81) | -0.71(-1.01 to -0.4) | -2.69(-3.48 to -1.89) |
| Sierra Leone | 299(185-444) | 16.51(10.22-24.5) | 834(533-1,237) | 23.31(14.91-34.6) | 1.14(0.96 to 1.33) | 6.74(6.45 to 7.03) | -0.85(-1.3 to -0.4) | -2.12(-2.36 to -1.88) |
| Togo | 204(131-326) | 11.56(7.45-18.47) | 496(285-797) | 15(8.61-24.08) | 0.87(0.52 to 1.21) | 5.36(4.5 to 6.24) | -0.02(-0.7 to 0.67) | -2.44(-2.68 to -2.2) |
| American Samoa | 7(5-10) | 38.9(27.33-54.64) | 5(3-8) | 36.49(22.14-59.31) | 0.12(-0.65 to 0.9) | -2.55(-3.76 to -1.33) | 0.97(0.08 to 1.87) | 1.23(-0.03 to 2.5) |
| Bermuda | 0(0-0) | 4.1(2.66-6.05) | 1(1-1) | 9.69(6.63-14.19) | 2.95(1.95 to 3.95) | 6.88(5.58 to 8.19) | 1.56(0.65 to 2.48) | 1.6(-0.16 to 3.4) |
| Cook Islands | 0(0-0) | 5.18(3.65-6.88) | 0(0-0) | 5.18(2.84-9.44) | 0.44(-0.42 to 1.31) | -1.25(-1.74 to -0.75) | -2.26(-2.5 to -2.02) | 4.57(2.13 to 7.06) |
| Greenland | 1(1-1) | 7.98(5.87-10.53) | 1(1-1) | 6.69(4.52-9.62) | -0.51(-0.95 to -0.06) | -1.59(-2.55 to -0.61) | -0.06(-0.49 to 0.37) | -0.16(-0.55 to 0.22) |
| Guam | 6(4-7) | 13.22(10.04-17.37) | 7(5-10) | 17.94(12.6-26.07) | 0.9(0.05 to 1.76) | -1.39(-1.97 to -0.8) | 5.61(4.58 to 6.66) | -1.57(-3.15 to 0.04) |
| Monaco | 0(0-0) | 2.34(1.25-4.33) | 0(0-0) | 2.40(1.28-4.32) | 0.1(0.06 to 0.13) | -0.04(-0.07 to -0.01) | 0.13(0.1 to 0.16) | 0.19(0.1 to 0.29) |
| Nauru | 1(1-2) | 27.75(18.66-37.95) | 1(1-1) | 24.81(16.79-35.3) | -0.36(-0.55 to -0.16) | 2.13(1.86 to 2.4) | 0.53(0.03 to 1.03) | -3.31(-3.55 to -3.06) |
| Niue | 0(0-0) | 18(12.55-25.7) | 0(0-0) | 74.93(57.25-99.94) | 5.43(4.8 to 6.07) | 0.39(0.22 to 0.57) | 0.39(0.22 to 0.57) | 15.26(13.38 to17.17) |
| Northern Mariana Islands | 1(1-2) | 10.19(7.52-13.57) | 1(1-1) | 9.52(7.04-12.6) | -0.51(-1.73 to 0.73) | -0.07(-2.39 to 2.31) | -1.77(-4.34 to 0.87) | -0.67(-2.14 to 0.82) |
| Palau | 0(0-0) | 4.51(3.28-6.11) | 0(0-0) | 2.97(2.09-4.2) | -1.38(-1.58 to -1.17) | -1.86(-2.14 to -1.57) | 0.24(-0.31 to 0.8) | -2.23(-2.4 to -2.06) |
| Puerto Rico | 89(75-107) | 8.93(7.53-10.73) | 56(46-70) | 12.59(10.43-15.72) | 1.32(0.3 to 2.35) | 12.08(9.03 to 15.21) | -2.3(-3.19 to -1.39) | -3.22(-4.45 to -1.97) |
| Saint Kitts and Nevis | 2(2-3) | 17.22(13.37-21.94) | 5(4-7) | 51.9(39.53-67.47) | 3.73(2.51 to 4.96) | 11.58(8.67 to 14.57) | -1.09(-2.02 to -0.15) | 2.68(0.55 to 4.85) |
| San Marino | 0(0-0) | 2.2(1.17-3.99) | 0(0-0) | 2.21(1.16-4.04) | 0.01(-0.02 to 0.04) | 0.67(0.62 to 0.72) | 0.07(0.02 to 0.11) | -0.58(-0.62 to -0.54) |
| Tokelau | 0(0-0) | 23.67(16.36-35.89) | 0(0-0) | 77.18(50.03-123.3) | 5.33(4.5 to 6.17) | -1.91(-2.08 to -1.73) | -1.91(-2.08 to -1.73) | 19.89(17.28 to22.56) |
| Tuvalu | 2(1-3) | 57.27(25.8-99.66) | 1(1-1) | 19.73(13.71-27.8) | -3.38(-3.59 to -3.18) | -5.73(-6.07 to -5.39) | -2.54(-3.1 to -1.97) | -2.18(-2.35 to -2.01) |
| United States Virgin Islands | 3(2-5) | 9.7(6.06-14.44) | 1(1-2) | 10.07(5.09-18.1) | 0.36(-0.97 to 1.71) | 5.74(4.99 to 6.49) | -0.74(-3.94 to 2.57) | -3.39(-5.71 to -1.01) |
| South Sudan | 4,071(1,797-6,538) | 155.13(68.49-249.16) | 4,812(2,869-7,328) | 112.03(66.81-170.61) | -1.09(-1.36 to -0.82) | -2.11(-2.41 to -1.81) | -0.41(-0.84 to 0.03) | -0.45(-0.83 to -0.08) |
| Sudan | 945(474-1,579) | 10.62(5.33-17.76) | 973(526-1,591) | 5.87(3.17-9.59) | -1.89(-1.96 to -1.81) | -1.14(-1.19 to -1.09) | -1.29(-1.34 to -1.24) | -2.99(-3.16 to -2.82) |

**sTable7 Number of DALYs and DALYs rate of fungal skin diseases and their AAPCs from 1990 to 2021 at the global, regional and national levels**

| *Rate per 100,000* | 1990 | | 2021 | | 1990-2021 | 1990-1999 | 2000-2009 | 2010-2021 |
| --- | --- | --- | --- | --- | --- | --- | --- | --- |
| *AAPC(95% CI)* | Number of DALYs | DALYs rate | Number of DALYs | DALYs rate | AAPC | AAPC | AAPC | AAPC |
| Fungal skin diseases | 683,592(269,007-1,499,209) | 39.31(15.47-86.2) | 924,335(365,560-2,019,319) | 45.94(18.17-100.37) | 0.5(0.46 to 0.54) | 0.79(0.78 to 0.81) | 0.64(0.62 to 0.66) | 0.13(0.04 to 0.23) |
| Male | 384,257(150,773-843,001) | 43.01(16.87-94.35) | 525,263(208,085-1,145,032) | 50.6(20.04-110.29) | 0.52(0.49 to 0.54) | 0.69(0.68 to 0.71) | 0.6(0.59 to 0.62) | 0.14(0.03 to 0.25) |
| Female | 299,334(118,556-657,726) | 35.4(14.02-77.78) | 399,072(158,028-873,435) | 40.99(16.23-89.7) | 0.46(0.42 to 0.5) | 0.81(0.78 to 0.83) | 0.68(0.66 to 0.69) | 0.13(0.07 to 0.18) |
| **Age groups** |  |  |  |  |  |  |  |  |
| 0-4 years | 212,453(82,231-455,985) | 34.27(13.26-73.55) | 260,874(101,892-560,076) | 39.64(15.48-85.1) | 0.47(0.42 to 0.52) | 1.16(1.12 to 1.21) | 0.21(0.12 to 0.31) | 0.11(0 to 0.21) |
| 5-9 years | 265,871(99,198-596,240) | 45.56(17-102.18) | 364,101(136,766-810,147) | 52.99(19.91-117.92) | 0.48(0.43 to 0.53) | 0.83(0.76 to 0.9) | 0.77(0.66 to 0.89) | -0.11(-0.16 to -0.07) |
| 10-14 years | 205,268(78,019-461,197) | 38.32(14.56-86.1) | 299,360(114,858-668,601) | 44.91(17.23-100.29) | 0.49(0.43 to 0.55) | 0.29(0.15 to 0.44) | 0.99(0.9 to 1.07) | 0.26(0.18 to 0.35) |
| **SDI region** |  |  |  |  |  |  |  |  |
| High SDI | 29,524(11,616-65,567) | 15.89(6.25-35.29) | 25,638(10,120-56,909) | 14.86(5.87-32.98) | -0.21(-0.22 to -0.21) | -0.39(-0.41 to -0.38) | -0.42(-0.43 to -0.41) | 0.1(0.09 to 0.12) |
| High-middle SDI | 48,018(19,067-106,138) | 17.55(6.97-38.79) | 38,393(15,041-84,943) | 16.63(6.51-36.79) | -0.17(-0.21 to -0.13) | 0.19(0.15 to 0.22) | -0.38(-0.46 to -0.3) | -0.28(-0.33 to -0.23) |
| Middle SDI | 162,449(64,367-357,209) | 28.14(11.15-61.89) | 163,503(64,540-364,511) | 28.84(11.39-64.3) | 0.07(0.06 to 0.09) | 0.27(0.26 to 0.28) | 0.22(0.21 to 0.24) | -0.23(-0.26 to -0.2) |
| Low-middle SDI | 208,714(82,417-461,923) | 44.21(17.46-97.84) | 259,373(102,716-573,191) | 44.73(17.71-98.85) | 0.03(0.02 to 0.05) | 0.17(0.13 to 0.21) | -0.02(-0.03 to 0) | -0.04(-0.06 to -0.03) |
| Low SDI | 234,455(92,456-513,572) | 102.42(40.39-224.35) | 436,916(174,659-945,607) | 94.94(37.95-205.47) | -0.26(-0.28 to -0.23) | 0.05(0.02 to 0.08) | -0.49(-0.51 to -0.47) | -0.29(-0.35 to -0.23) |
| **GBD region** |  |  |  |  |  |  |  |  |
| Andean Latin America | 6,577(2,606-14,489) | 44.28(17.55-97.55) | 7,681(3,063-16,874) | 42.45(16.93-93.25) | -0.14(-0.16 to -0.13) | -0.2(-0.22 to -0.18) | -0.09(-0.11 to -0.07) | -0.14(-0.16 to -0.12) |
| Australasia | 1,019(410-2,224) | 22.22(8.95-48.49) | 1,251(506-2,736) | 21.83(8.82-47.74) | -0.06(-0.08 to -0.04) | -0.08(-0.1 to -0.06) | -0.23(-0.27 to -0.18) | 0.09(0.07 to 0.11) |
| Caribbean | 3,390(1,350-7,283) | 29.7(11.83-63.81) | 3,423(1,360-7,372) | 29.76(11.82-64.08) | 0(-0.02 to 0.03) | -0.05(-0.14 to 0.04) | 0.01(-0.01 to 0.03) | 0.04(0.02 to 0.06) |
| Central Asia | 3,731(1,453-8,160) | 14.93(5.81-32.65) | 4,149(1,604-9,171) | 14.99(5.8-33.14) | 0.01(-0.02 to 0.04) | 0.38(0.33 to 0.43) | -0.3(-0.34 to -0.25) | -0.03(-0.09 to 0.03) |
| Central Europe | 4,599(1,808-10,159) | 15.6(6.13-34.46) | 2,744(1,082-6,043) | 15.5(6.11-34.14) | -0.02(-0.03 to -0.01) | 0.13(0.13 to 0.14) | -0.28(-0.29 to -0.27) | 0.1(0.09 to 0.11) |
| Central Latin America | 13,860(5,443-30,079) | 21.53(8.45-46.72) | 13,654(5,382-30,263) | 21.51(8.48-47.67) | 0(-0.04 to 0.03) | -0.4(-0.44 to -0.36) | -0.14(-0.15 to -0.13) | 0.46(0.36 to 0.56) |
| Central Sub-Saharan Africa | 35,405(13,333-78,441) | 139.95(52.7-310.06) | 67,282(25,994-152,998) | 114.66(44.3-260.73) | -0.65(-0.68 to -0.62) | -0.31(-0.33 to -0.29) | -0.75(-0.76 to -0.73) | -0.87(-0.94 to -0.8) |
| East Asia | 54,895(21,296-121,277) | 16.64(6.46-36.77) | 38,811(15,203-85,384) | 14.52(5.69-31.94) | -0.45(-0.49 to -0.4) | -0.06(-0.09 to -0.02) | -1.07(-1.2 to -0.94) | -0.24(-0.28 to -0.2) |
| Eastern Europe | 8,187(3,241-18,206) | 15.91(6.3-35.38) | 5,723(2,262-12,692) | 16.15(6.38-35.81) | 0.05(0.02 to 0.07) | 0.47(0.44 to 0.5) | -0.7(-0.72 to -0.68) | 0.33(0.28 to 0.38) |
| Eastern Sub-Saharan Africa | 129,905(51,564-280,612) | 143.43(56.93-309.83) | 219,441(87,356-470,620) | 122.98(48.96-263.75) | -0.51(-0.56 to -0.46) | -0.06(-0.2 to 0.09) | -0.63(-0.66 to -0.6) | -0.77(-0.8 to -0.74) |
| High-income Asia Pacific | 6,599(2,609-14,638) | 18.75(7.41-41.59) | 3,966(1,577-8,837) | 17.69(7.03-39.4) | -0.18(-0.2 to -0.17) | -0.49(-0.5 to -0.47) | -0.15(-0.18 to -0.13) | 0.04(0.01 to 0.08) |
| High-income North America | 4,550(1,823-9,784) | 7.38(2.96-15.86) | 4,798(1,935-10,331) | 7.31(2.95-15.74) | -0.02(-0.04 to -0.01) | -0.03(-0.07 to 0) | -0.18(-0.21 to -0.16) | 0.13(0.12 to 0.14) |
| North Africa and Middle East | 14,049(5,554-30,010) | 10(3.95-21.36) | 14,496(5,780-32,167) | 7.91(3.15-17.55) | -0.77(-0.82 to -0.72) | -1.29(-1.39 to -1.19) | -0.52(-0.55 to -0.48) | -0.62(-0.71 to -0.53) |
| Oceania | 580(227-1,274) | 21.64(8.46-47.55) | 1,097(435-2,393) | 21.59(8.56-47.1) | -0.01(-0.03 to 0.01) | -0.01(-0.03 to 0.01) | 0.06(0 to 0.11) | -0.06(-0.07 to -0.05) |
| South Asia | 194,796(73,942-431,986) | 44.95(17.06-99.68) | 200,086(77,415-438,485) | 39.46(15.27-86.48) | -0.43(-0.45 to -0.41) | -0.2(-0.24 to -0.17) | -0.59(-0.62 to -0.57) | -0.5(-0.53 to -0.46) |
| Southeast Asia | 60,995(24,166-134,246) | 35.72(14.15-78.62) | 60,919(24,122-133,695) | 35.28(13.97-77.44) | -0.04(-0.05 to -0.04) | 0(-0.01 to 0.01) | -0.08(-0.09 to -0.07) | -0.04(-0.05 to -0.03) |
| Southern Latin America | 2,759(1,074-6,141) | 18.48(7.19-41.14) | 2,576(1,008-5,703) | 17.77(6.96-39.35) | -0.13(-0.15 to -0.11) | -0.29(-0.33 to -0.25) | -0.24(-0.28 to -0.2) | 0.1(0.08 to 0.12) |
| Southern Sub-Saharan Africa | 9,300(3,680-20,139) | 44.95(17.79-97.34) | 10,766(4,247-23,114) | 44.73(17.65-96.05) | -0.04(-0.07 to -0.01) | 0.37(0.34 to 0.4) | -0.04(-0.07 to 0) | -0.42(-0.5 to -0.34) |
| Tropical Latin America | 16,746(6,664-36,562) | 31.23(12.43-68.19) | 14,885(5,827-32,608) | 29.65(11.61-64.97) | -0.17(-0.18 to -0.16) | -0.28(-0.3 to -0.26) | -0.08(-0.11 to -0.06) | -0.15(-0.16 to -0.14) |
| Western Europe | 17,436(6,830-38,432) | 24.55(9.62-54.12) | 16,306(6,410-35,700) | 23.94(9.41-52.41) | -0.08(-0.09 to -0.07) | -0.1(-0.11 to -0.08) | -0.3(-0.31 to -0.29) | 0.12(0.11 to 0.13) |
| Western Sub-Saharan Africa | 94,216(38,000-205,027) | 107.21(43.24-233.3) | 230,280(93,305-498,360) | 107.23(43.45-232.05) | -0.01(-0.04 to 0.01) | 0.52(0.48 to 0.56) | -0.09(-0.11 to -0.07) | -0.09(-0.15 to -0.02) |
| **204 countries and territories** |  |  |  |  |  |  |  |  |
| China | 53,305(20,677-117,711) | 16.74(6.49-36.97) | 37,761(14,784-83,036) | 14.54(5.69-31.98) | -0.46(-0.51 to -0.42) | -0.06(-0.09 to -0.02) | -1.1(-1.23 to -0.97) | -0.25(-0.29 to -0.21) |
| Democratic People's Republic of Korea | 886(346-1,933) | 14.9(5.82-32.49) | 696(272-1,559) | 14.59(5.71-32.66) | -0.08(-0.12 to -0.03) | 0.17(0.11 to 0.23) | -0.27(-0.39 to -0.15) | -0.12(-0.17 to -0.06) |
| Taiwan (Province of China) | 704(269-1,492) | 12.78(4.89-27.08) | 353(138-741) | 11.97(4.70-25.14) | -0.22(-0.24 to -0.2) | -0.47(-0.5 to -0.44) | -0.05(-0.09 to -0.02) | -0.15(-0.2 to -0.11) |
| Cambodia | 1,583(632-3,469) | 33.96(13.56-74.42) | 1,722(679-3,791) | 33.65(13.28-74.09) | -0.03(-0.07 to 0.02) | 0.43(0.4 to 0.46) | -0.29(-0.41 to -0.17) | -0.14(-0.21 to -0.07) |
| Indonesia | 25,299(10,026-56,115) | 37.35(14.80-82.84) | 24,687(9,745-54,646) | 36.69(14.48-81.21) | -0.06(-0.07 to -0.05) | -0.06(-0.07 to -0.04) | -0.09(-0.1 to -0.08) | -0.04(-0.06 to -0.02) |
| Lao People's Democratic Republic | 647(257-1,398) | 35.12(13.94-75.85) | 783(314-1,707) | 34.1(13.66-74.34) | -0.1(-0.11 to -0.08) | 0.06(0.03 to 0.08) | -0.04(-0.05 to -0.02) | -0.28(-0.32 to -0.24) |
| Malaysia | 2,203(869-4,807) | 33.52(13.22-73.14) | 2,519(991-5,488) | 33.09(13.02-72.08) | -0.04(-0.07 to -0.01) | 0.01(-0.01 to 0.02) | -0.01(-0.1 to 0.07) | -0.1(-0.16 to -0.05) |
| Maldives | 34(13-74) | 32.28(12.84-70.46) | 32(13-71) | 32.08(12.7-71.19) | -0.02(-0.04 to 0.01) | 0.5(0.48 to 0.52) | -0.54(-0.59 to -0.49) | 0.04(-0.02 to 0.09) |
| Myanmar | 5,192(2,059-11,286) | 35.14(13.93-76.38) | 5,327(2,119-11,666) | 34.12(13.57-74.71) | -0.1(-0.12 to -0.08) | 0.05(-0.01 to 0.1) | -0.16(-0.18 to -0.15) | -0.16(-0.19 to -0.12) |
| Philippines | 8,789(3,520-19,451) | 34.86(13.96-77.14) | 12,083(4,824-26,720) | 35.54(14.19-78.59) | 0.06(0.05 to 0.07) | 0.03(0.02 to 0.05) | 0.1(0.08 to 0.12) | 0.04(0.02 to 0.06) |
| Sri Lanka | 2,478(971-5,483) | 44.79(17.56-99.1) | 2,239(877-4,976) | 43.87(17.19-97.49) | -0.07(-0.1 to -0.04) | -0.14(-0.17 to -0.1) | -0.34(-0.42 to -0.26) | 0.25(0.22 to 0.27) |
| Thailand | 5,708(2,266-12,652) | 33.86(13.44-75.05) | 3,218(1,269-7,126) | 32.95(12.99-72.97) | -0.09(-0.11 to -0.07) | -0.19(-0.24 to -0.14) | -0.01(-0.05 to 0.02) | -0.06(-0.08 to -0.03) |
| Timor-Leste | 110(44-238) | 32.93(13.17-71.47) | 178(71-384) | 34.1(13.57-73.81) | 0.11(0.09 to 0.14) | 0.21(0.18 to 0.23) | 0.09(0.08 to 0.1) | 0.03(-0.03 to 0.09) |
| Viet Nam | 8,746(3,506-18,987) | 32.99(13.22-71.62) | 7,969(3,166-17,237) | 32.18(12.79-69.61) | -0.08(-0.12 to -0.03) | 0.27(0.25 to 0.28) | -0.4(-0.5 to -0.3) | -0.05(-0.15 to 0.04) |
| Fiji | 63(25-137) | 22.23(8.78-48.56) | 61(24-133) | 22.27(8.74-48.76) | 0(-0.03 to 0.02) | 0.08(0.05 to 0.1) | -0.1(-0.12 to -0.09) | 0.03(-0.04 to 0.09) |
| Kiribati | 7(3-16) | 24.06(9.67-52.81) | 10(4-22) | 24.02(9.3-52.32) | -0.02(-0.05 to 0.01) | 0.01(-0.01 to 0.02) | -0.1(-0.16 to -0.05) | 0.05(-0.01 to 0.11) |
| Marshall Islands | 5(2-11) | 23.01(9.09-50.42) | 4(2-9) | 22.75(9.11-49.95) | -0.04(-0.06 to -0.02) | 0.2(0.15 to 0.24) | -0.21(-0.23 to -0.19) | -0.08(-0.11 to -0.05) |
| Micronesia (Federated States of) | 11(4-24) | 23.45(9.2-51.96) | 7(3-15) | 22.43(8.77-49.31) | -0.14(-0.16 to -0.12) | -0.02(-0.05 to 0) | -0.23(-0.25 to -0.2) | -0.16(-0.17 to -0.14) |
| Papua New Guinea | 364(142-801) | 21.43(8.36-47.14) | 843(333-1,831) | 21.51(8.51-46.74) | 0(-0.01 to 0.02) | 0.02(0 to 0.05) | 0.1(0.06 to 0.14) | -0.08(-0.09 to -0.07) |
| Samoa | 16(6-35) | 22(8.68-49.33) | 17(7-37) | 20.99(8.35-45.89) | -0.16(-0.19 to -0.14) | -0.32(-0.35 to -0.29) | -0.09(-0.12 to -0.07) | -0.1(-0.17 to -0.04) |
| Solomon Islands | 34(14-76) | 22.08(8.68-48.69) | 57(22-126) | 21.78(8.63-48.43) | -0.05(-0.06 to -0.04) | -0.15(-0.17 to -0.14) | 0.06(0.03 to 0.09) | -0.05(-0.08 to -0.03) |
| Tonga | 9(3-20) | 21.18(8.32-47.16) | 8(3-18) | 20.7(8.17-45.51) | -0.08(-0.1 to -0.06) | -0.08(-0.08 to -0.07) | -0.08(-0.08 to -0.07) | -0.09(-0.15 to -0.04) |
| Vanuatu | 15(6-32) | 21.74(8.68-47.64) | 26(10-58) | 22.08(8.73-49.38) | 0.05(0.03 to 0.07) | 0.09(0.06 to 0.13) | 0.07(0.05 to 0.1) | 0(-0.03 to 0.03) |
| Armenia | 156(61-336) | 15(5.82-32.23) | 91(35-198) | 15.29(5.89-33.39) | 0.06(0.04 to 0.09) | 0.52(0.5 to 0.55) | -0.43(-0.48 to -0.38) | 0.16(0.11 to 0.21) |
| Azerbaijan | 365(141-802) | 15.03(5.82-33.05) | 363(143-799) | 15.39(6.06-33.84) | 0.08(0.02 to 0.13) | 0.41(0.34 to 0.49) | -0.28(-0.39 to -0.18) | 0.12(0.03 to 0.21) |
| Georgia | 208(81-454) | 15.21(5.89-33.2) | 112(43-243) | 15.21(5.85-33.03) | 0.01(-0.03 to 0.04) | 0.37(0.33 to 0.41) | -0.35(-0.43 to -0.27) | 0.04(-0.01 to 0.09) |
| Kazakhstan | 783(302-1,704) | 15.06(5.81-32.8) | 813(319-1,775) | 14.99(5.88-32.71) | -0.01(-0.04 to 0.02) | 0.46(0.41 to 0.5) | -0.66(-0.74 to -0.59) | 0.12(0.08 to 0.15) |
| Kyrgyzstan | 251(97-548) | 14.94(5.80-32.65) | 342(131-761) | 15.05(5.76-33.47) | 0.03(-0.01 to 0.07) | 0.35(0.33 to 0.38) | -0.33(-0.4 to -0.25) | 0.08(-0.01 to 0.18) |
| Mongolia | 135(53-297) | 15.02(5.85-33.04) | 163(64-359) | 14.99(5.86-33.07) | 0(-0.04 to 0.03) | 0.42(0.38 to 0.46) | -0.46(-0.53 to -0.39) | 0.07(0.01 to 0.12) |
| Tajikistan | 343(135-752) | 14.76(5.83-32.4) | 535(206-1,185) | 14.93(5.74-33.07) | 0.04(0.01 to 0.07) | 0.32(0.3 to 0.34) | -0.14(-0.19 to -0.08) | -0.03(-0.08 to 0.03) |
| Turkmenistan | 223(88-491) | 14.88(5.84-32.71) | 229(89-505) | 15.06(5.87-33.13) | 0.04(0.02 to 0.05) | 0.24(0.2 to 0.28) | -0.06(-0.1 to -0.03) | -0.05(-0.07 to -0.03) |
| Uzbekistan | 1,268(496-2,793) | 14.82(5.8-32.64) | 1,500(580-3,329) | 14.87(5.75-32.98) | 0.01(-0.01 to 0.03) | 0.4(0.38 to 0.42) | -0.16(-0.23 to -0.1) | -0.17(-0.2 to -0.14) |
| Albania | 168(66-369) | 15.02(5.87-33.02) | 68(26-148) | 15.24(5.85-33.36) | 0.04(0.02 to 0.07) | 0.22(0.16 to 0.27) | 0.15(0.11 to 0.2) | -0.2(-0.24 to -0.16) |
| Bosnia and Herzegovina | 167(65-369) | 15.27(5.94-33.71) | 75(29-166) | 15.31(5.9-33.87) | 0(-0.03 to 0.04) | 0.2(0.11 to 0.29) | -0.05(-0.1 to 0) | -0.1(-0.14 to -0.05) |
| Bulgaria | 267(103-595) | 15.38(5.95-34.27) | 149(57-326) | 15.31(5.85-33.36) | -0.01(-0.03 to 0.01) | 0.22(0.19 to 0.25) | -0.52(-0.55 to -0.48) | 0.21(0.2 to 0.22) |
| Croatia | 152(58-329) | 15.38(5.88-33.34) | 92(35-201) | 15.33(5.93-33.74) | -0.01(-0.02 to 0) | -0.04(-0.08 to -0.01) | -0.05(-0.07 to -0.04) | 0.05(0.04 to 0.06) |
| Czechia | 342(133-751) | 15.5(6.03-34.09) | 260(103-567) | 15.18(6-33.05) | -0.07(-0.11 to -0.02) | 0.07(0.01 to 0.14) | -0.51(-0.62 to -0.41) | 0.23(0.21 to 0.25) |
| Hungary | 331(129-724) | 15.52(6.04-33.97) | 211(84-463) | 15.19(6.02-33.36) | -0.07(-0.09 to -0.04) | -0.07(-0.12 to -0.01) | -0.17(-0.22 to -0.12) | 0(-0.02 to 0.01) |
| North Macedonia | 80(31-175) | 15.24(5.94-33.2) | 50(20-110) | 15.31(6-33.47) | 0.01(-0.01 to 0.04) | 0.13(0.12 to 0.15) | -0.02(-0.07 to 0.03) | -0.03(-0.07 to 0) |
| Montenegro | 25(10-54) | 15.23(5.92-33.4) | 17(7-37) | 15.19(5.87-33.2) | -0.01(-0.02 to 0.01) | 0.02(0 to 0.05) | 0(-0.03 to 0.03) | -0.03(-0.05 to -0.01) |
| Poland | 1,539(610-3,418) | 16.07(6.36-35.69) | 937(372-2,102) | 15.92(6.32-35.72) | -0.03(-0.04 to -0.02) | 0.17(0.16 to 0.18) | -0.34(-0.36 to -0.33) | 0.11(0.1 to 0.13) |
| Romania | 856(330-1,851) | 15.38(5.93-33.24) | 461(175-1,001) | 15.3(5.82-33.27) | -0.02(-0.04 to 0.01) | 0.19(0.14 to 0.24) | -0.38(-0.41 to -0.35) | 0.08(0.04 to 0.12) |
| Serbia | 332(130-732) | 15.3(5.99-33.74) | 207(79-457) | 15.59(5.96-34.44) | 0.07(0.03 to 0.1) | 0.08(0.01 to 0.15) | -0.1(-0.13 to -0.07) | 0.18(0.13 to 0.23) |
| Slovakia | 204(79-450) | 15.35(5.99-33.93) | 129(50-283) | 15.11(5.85-33.03) | -0.05(-0.08 to -0.02) | 0.16(0.09 to 0.23) | -0.3(-0.37 to -0.23) | 0.01(-0.01 to 0.02) |
| Slovenia | 64(25-139) | 15.42(5.93-33.52) | 48(19-104) | 15.22(5.98-33.3) | -0.04(-0.08 to 0) | 0.05(-0.02 to 0.13) | -0.34(-0.38 to -0.3) | 0.17(0.09 to 0.25) |
| Belarus | 367(143-796) | 15.26(5.94-33.09) | 243(95-534) | 15.38(6-33.84) | 0.03(-0.01 to 0.08) | 0.46(0.43 to 0.49) | -0.56(-0.62 to -0.5) | 0.22(0.12 to 0.32) |
| Estonia | 53(20-116) | 15.16(5.76-33.12) | 33(13-73) | 15.27(5.92-33.63) | 0.02(-0.02 to 0.06) | 0.47(0.45 to 0.5) | -0.62(-0.67 to -0.56) | 0.23(0.15 to 0.3) |
| Latvia | 86(33-188) | 15.1(5.88-32.98) | 45(18-99) | 15.29(5.94-33.47) | 0.05(0 to 0.09) | 0.62(0.57 to 0.66) | -0.58(-0.71 to -0.46) | 0.11(0.07 to 0.15) |
| Lithuania | 126(49-274) | 15.15(5.85-33.04) | 62(24-135) | 15.26(5.87-33.15) | 0.02(0 to 0.05) | 0.39(0.32 to 0.45) | -0.25(-0.29 to -0.21) | 0(-0.04 to 0.03) |
| Republic of Moldova | 187(72-410) | 15.15(5.79-33.2) | 81(31-177) | 15.44(5.93-33.89) | 0.06(0.03 to 0.1) | 0.5(0.44 to 0.55) | -0.39(-0.44 to -0.33) | 0.11(0.06 to 0.16) |
| Russian Federation | 5,547(2,192-12,337) | 15.98(6.32-35.55) | 4,213(1,662-9,352) | 16.15(6.37-35.86) | 0.03(0.01 to 0.06) | 0.48(0.44 to 0.52) | -0.72(-0.75 to -0.7) | 0.31(0.27 to 0.36) |
| Ukraine | 1,821(721-4,076) | 16.01(6.33-35.83) | 1,047(419-2,335) | 16.5(6.6-36.79) | 0.1(0.06 to 0.14) | 0.42(0.37 to 0.47) | -0.65(-0.74 to -0.56) | 0.47(0.43 to 0.51) |
| Brunei Darussalam | 17(7-38) | 18.98(7.68-42.41) | 17(7-38) | 18.16(7.14-40.17) | -0.14(-0.15 to -0.12) | -0.32(-0.37 to -0.27) | -0.02(-0.05 to 0) | -0.1(-0.11 to -0.09) |
| Japan | 4,317(1,714-9,607) | 18.7(7.42-41.61) | 2,765(1,105-6,156) | 17.9(7.16-39.86) | -0.14(-0.15 to -0.13) | -0.37(-0.38 to -0.35) | -0.12(-0.14 to -0.1) | 0.05(0.04 to 0.07) |
| Republic of Korea | 2,149(845-4,735) | 18.9(7.43-41.64) | 1,049(408-2,325) | 17.27(6.72-38.28) | -0.29(-0.32 to -0.25) | -0.72(-0.8 to -0.64) | -0.24(-0.26 to -0.22) | 0.04(-0.03 to 0.1) |
| Singapore | 116(46-251) | 17.87(7.05-38.65) | 135(52-295) | 16.59(6.42-36.26) | -0.24(-0.27 to -0.21) | -0.38(-0.41 to -0.35) | -0.17(-0.2 to -0.14) | -0.17(-0.24 to -0.11) |
| Australia | 834(337-1,836) | 22.03(8.91-48.5) | 1,028(419-2,256) | 21.65(8.82-47.5) | -0.06(-0.08 to -0.04) | -0.1(-0.12 to -0.07) | -0.21(-0.26 to -0.17) | 0.1(0.08 to 0.12) |
| New Zealand | 185(74-409) | 23.09(9.29-51.06) | 223(89-497) | 22.7(9.07-50.64) | -0.06(-0.09 to -0.02) | -0.05(-0.13 to 0.02) | -0.26(-0.36 to -0.17) | 0.08(0.06 to 0.09) |
| Andorra | 2(1-5) | 25.53(10.07-56.01) | 3(1-6) | 24.98(9.77-54.2) | -0.07(-0.1 to -0.04) | -0.48(-0.55 to -0.42) | 0.03(0.01 to 0.04) | 0.18(0.12 to 0.24) |
| Austria | 339(133-741) | 25.12(9.86-54.96) | 312(122-685) | 24.04(9.44-52.79) | -0.14(-0.16 to -0.12) | -0.17(-0.23 to -0.11) | -0.19(-0.2 to -0.17) | -0.09(-0.12 to -0.06) |
| Belgium | 452(176-972) | 25.02(9.76-53.8) | 466(182-1,008) | 24.37(9.51-52.7) | -0.09(-0.1 to -0.07) | -0.15(-0.19 to -0.12) | -0.27(-0.3 to -0.25) | 0.12(0.09 to 0.15) |
| Cyprus | 51(20-110) | 25.52(10.05-55.41) | 52(21-113) | 23.98(9.63-51.56) | -0.2(-0.23 to -0.17) | -0.02(-0.06 to 0.02) | -0.56(-0.62 to -0.49) | -0.04(-0.08 to -0.01) |
| Denmark | 224(87-485) | 25.39(9.88-54.94) | 232(92-506) | 24.3(9.67-53.02) | -0.13(-0.15 to -0.11) | -0.27(-0.31 to -0.24) | -0.13(-0.16 to -0.11) | -0.04(-0.08 to 0) |
| Finland | 245(96-528) | 25.37(9.93-54.67) | 208(81-452) | 24.51(9.57-53.31) | -0.11(-0.14 to -0.08) | -0.2(-0.24 to -0.17) | -0.33(-0.35 to -0.31) | 0.17(0.1 to 0.24) |
| France | 2,958(1,173-6,429) | 25.25(10.01-54.88) | 2,825(1,118-6,197) | 24.33(9.63-53.39) | -0.11(-0.14 to -0.09) | -0.22(-0.25 to -0.19) | -0.26(-0.3 to -0.21) | 0.11(0.07 to 0.16) |
| Germany | 3,285(1,294-7,113) | 25.37(9.99-54.94) | 2,886(1,138-6,200) | 24.12(9.51-51.82) | -0.16(-0.18 to -0.15) | -0.21(-0.24 to -0.18) | -0.15(-0.16 to -0.14) | -0.14(-0.16 to -0.11) |
| Greece | 512(200-1,106) | 25.29(9.9-54.66) | 341(135-738) | 24.42(9.65-52.93) | -0.11(-0.15 to -0.07) | -0.26(-0.27 to -0.24) | -0.31(-0.41 to -0.21) | 0.2(0.16 to 0.24) |
| Iceland | 16(6-34) | 24.58(9.67-53.31) | 16(6-35) | 23.92(9.35-51.71) | -0.09(-0.11 to -0.06) | -0.18(-0.22 to -0.13) | -0.19(-0.26 to -0.12) | 0.1(0.06 to 0.13) |
| Ireland | 251(98-551) | 25.57(9.95-56.09) | 243(95-523) | 24.32(9.51-52.49) | -0.16(-0.18 to -0.14) | -0.33(-0.36 to -0.31) | -0.45(-0.47 to -0.43) | 0.24(0.21 to 0.28) |
| Israel | 540(214-1,162) | 35.22(13.93-75.79) | 885(354-1,926) | 33.69(13.46-73.31) | -0.14(-0.16 to -0.13) | -0.28(-0.31 to -0.25) | -0.2(-0.21 to -0.19) | 0.03(0.01 to 0.05) |
| Italy | 2,415(938-5,395) | 26.17(10.16-58.45) | 1,923(749-4,282) | 25.31(9.86-56.34) | -0.11(-0.12 to -0.1) | -0.37(-0.38 to -0.35) | -0.24(-0.26 to -0.22) | 0.21(0.19 to 0.23) |
| Luxembourg | 17(7-36) | 25.22(9.87-54.59) | 24(10-53) | 24.17(9.47-52.81) | -0.13(-0.16 to -0.1) | -0.31(-0.41 to -0.22) | -0.1(-0.13 to -0.07) | -0.02(-0.04 to 0) |
| Malta | 22(9-48) | 25.21(9.84-55.32) | 15(6-33) | 23.92(9.32-52.04) | -0.17(-0.2 to -0.14) | -0.11(-0.13 to -0.09) | -0.24(-0.31 to -0.18) | -0.16(-0.2 to -0.11) |
| Netherlands | 674(269-1,463) | 24.73(9.86-53.7) | 646(257-1,399) | 24.1(9.59-52.16) | -0.08(-0.1 to -0.07) | -0.11(-0.14 to -0.08) | -0.11(-0.13 to -0.08) | -0.04(-0.06 to -0.02) |
| Norway | 206(80-461) | 25.81(9.99-57.75) | 233(91-519) | 25.23(9.81-56.14) | -0.07(-0.1 to -0.04) | -0.2(-0.28 to -0.12) | -0.15(-0.17 to -0.13) | 0.11(0.06 to 0.17) |
| Portugal | 550(214-1,202) | 26.01(10.11-56.80) | 330(128-715) | 24.25(9.42-52.45) | -0.22(-0.24 to -0.21) | -0.5(-0.52 to -0.49) | -0.19(-0.22 to -0.15) | -0.03(-0.07 to 0.01) |
| Spain | 1,184(474-2,466) | 15.11(6.05-31.47) | 902(358-1,826) | 13.92(5.53-28.18) | -0.27(-0.29 to -0.24) | -0.54(-0.57 to -0.51) | -0.37(-0.41 to -0.32) | 0.02(-0.04 to 0.08) |
| Sweden | 390(154-868) | 25.26(9.97-56.19) | 455(177-1,020) | 25(9.74-56.03) | -0.03(-0.06 to 0) | 0.23(0.18 to 0.28) | -0.47(-0.51 to -0.43) | 0.15(0.12 to 0.17) |
| Switzerland | 287(112-617) | 24.8(9.66-53.39) | 320(125-697) | 24.03(9.41-52.31) | -0.1(-0.12 to -0.08) | -0.09(-0.12 to -0.06) | -0.21(-0.28 to -0.14) | -0.02(-0.05 to 0) |
| United Kingdom | 2,801(1,088-6,233) | 25.65(9.97-57.07) | 2,971(1,156-6,605) | 25.22(9.81-56.05) | -0.05(-0.07 to -0.03) | -0.01(-0.04 to 0.02) | -0.36(-0.38 to -0.34) | 0.18(0.14 to 0.22) |
| Argentina | 1,887(736-4,201) | 18.62(7.26-41.45) | 1,827(714-4,066) | 17.94(7.01-39.93) | -0.12(-0.15 to -0.08) | -0.33(-0.35 to -0.31) | -0.2(-0.27 to -0.13) | 0.14(0.07 to 0.2) |
| Chile | 723(283-1,604) | 18.21(7.14-40.37) | 634(249-1,390) | 17.35(6.82-38.07) | -0.15(-0.18 to -0.13) | -0.18(-0.22 to -0.15) | -0.38(-0.4 to -0.35) | 0.07(0.01 to 0.12) |
| Uruguay | 149(58-333) | 18.16(7.09-40.68) | 116(45-253) | 17.55(6.87-38.44) | -0.11(-0.13 to -0.09) | -0.3(-0.33 to -0.27) | -0.05(-0.07 to -0.03) | 0.01(-0.03 to 0.05) |
| Canada | 445(171-1,008) | 7.73(2.98-17.53) | 460(175-1,030) | 7.45(2.84-16.69) | -0.12(-0.14 to -0.09) | -0.18(-0.24 to -0.12) | -0.35(-0.38 to -0.31) | 0.14(0.11 to 0.16) |
| United States of America | 4,103(1,656-8,706) | 7.34(2.96-15.57) | 4,337(1,749-9,222) | 7.3(2.94-15.52) | -0.01(-0.03 to 0) | -0.02(-0.05 to 0.02) | -0.16(-0.19 to -0.14) | 0.13(0.12 to 0.15) |
| Antigua and Barbuda | 5(2-11) | 28.94(11.44-63) | 5(2-11) | 28.58(11.26-62.63) | -0.04(-0.06 to -0.02) | -0.1(-0.16 to -0.04) | 0.02(0.02 to 0.03) | -0.06(-0.08 to -0.03) |
| Bahamas | 24(10-53) | 30.28(12.14-65.26) | 24(10-54) | 30.14(11.72-66.05) | -0.01(-0.03 to 0.01) | -0.05(-0.1 to 0) | -0.1(-0.13 to -0.07) | 0.08(0.06 to 0.11) |
| Barbados | 18(7-40) | 29.27(11.53-63.96) | 13(5-29) | 28.63(11.23-62.48) | -0.07(-0.09 to -0.05) | -0.23(-0.27 to -0.19) | -0.04(-0.07 to -0.02) | 0.04(0.01 to 0.06) |
| Belize | 24(9-51) | 28.84(11.44-61.92) | 36(14-78) | 29.13(11.35-63.54) | 0.03(0 to 0.06) | 0.21(0.17 to 0.25) | -0.17(-0.24 to -0.11) | 0.02(-0.01 to 0.05) |
| Cuba | 698(276-1,510) | 27.88(11.04-60.3) | 489(194-1,074) | 27.54(10.9-60.46) | -0.04(-0.06 to -0.02) | 0.02(-0.03 to 0.07) | -0.08(-0.12 to -0.04) | -0.04(-0.06 to -0.02) |
| Dominica | 7(3-16) | 29.79(11.73-65.08) | 4(2-9) | 30.81(12.01-67.32) | 0.11(0.09 to 0.13) | 0.05(0.01 to 0.09) | 0.1(0.08 to 0.13) | 0.16(0.11 to 0.2) |
| Dominican Republic | 797(313-1,727) | 29.58(11.63-64.06) | 852(337-1,850) | 29(11.47-62.95) | -0.07(-0.08 to -0.05) | -0.27(-0.3 to -0.24) | 0.09(0.08 to 0.1) | -0.06(-0.09 to -0.03) |
| Grenada | 10(4-21) | 29.39(11.65-63.48) | 6(2-14) | 29.19(11.39-62.84) | -0.02(-0.04 to 0) | -0.14(-0.17 to -0.11) | 0.05(0.01 to 0.09) | 0(-0.03 to 0.03) |
| Guyana | 90(36-194) | 30.64(12.29-66.09) | 65(26-141) | 30.65(12.16-66.18) | 0(-0.03 to 0.03) | 0.02(-0.05 to 0.08) | 0.2(0.14 to 0.27) | -0.2(-0.21 to -0.18) |
| Haiti | 869(350-1,829) | 32.04(12.89-67.43) | 1,367(556-2,935) | 31.41(12.77-67.43) | -0.07(-0.09 to -0.05) | -0.05(-0.09 to 0) | -0.13(-0.15 to -0.12) | -0.03(-0.05 to -0.02) |
| Jamaica | 240(96-526) | 28.69(11.47-62.97) | 169(67-372) | 28.94(11.48-63.65) | 0.03(0 to 0.06) | 0.01(-0.04 to 0.06) | 0(-0.09 to 0.1) | 0.07(0.05 to 0.08) |
| Saint Lucia | 15(6-33) | 29.46(11.70-63.56) | 9(3-19) | 29.05(11.26-63.25) | -0.05(-0.07 to -0.03) | -0.1(-0.13 to -0.06) | -0.03(-0.09 to 0.03) | -0.03(-0.06 to 0) |
| Saint Vincent and the Grenadines | 12(5-27) | 29.58(11.83-64.54) | 7(3-16) | 29.6(11.71-64.69) | 0(-0.02 to 0.02) | -0.04(-0.07 to -0.02) | -0.06(-0.1 to -0.03) | 0.09(0.05 to 0.12) |
| Suriname | 40(16-86) | 30.48(12.09-65.64) | 43(17-92) | 30.14(11.99-64.49) | -0.04(-0.06 to -0.02) | -0.04(-0.08 to -0.01) | -0.04(-0.05 to -0.03) | -0.03(-0.08 to 0.01) |
| Trinidad and Tobago | 124(50-265) | 30.42(12.19-65.2) | 81(32-175) | 29.66(11.73-64.34) | -0.09(-0.11 to -0.06) | 0.12(0.06 to 0.18) | -0.42(-0.45 to -0.39) | 0.07(0.05 to 0.09) |
| Bolivia (Plurinational State of) | 1,173(466-2,590) | 43.69(17.36-96.43) | 1,480(586-3,274) | 42.45(16.8-93.91) | -0.1(-0.11 to -0.08) | 0(-0.01 to 0.02) | -0.16(-0.18 to -0.15) | -0.12(-0.15 to -0.1) |
| Ecuador | 1,655(651-3,658) | 42.83(16.85-94.64) | 2,106(835-4,634) | 41.54(16.46-91.39) | -0.1(-0.13 to -0.08) | -0.37(-0.42 to -0.32) | 0.1(0.05 to 0.15) | -0.02(-0.05 to 0) |
| Peru | 3,748(1,475-8,175) | 45.15(17.77-98.49) | 4,095(1,626-9,022) | 42.93(17.05-94.6) | -0.17(-0.18 to -0.15) | -0.17(-0.2 to -0.15) | -0.12(-0.14 to -0.09) | -0.21(-0.23 to -0.18) |
| Colombia | 2,449(936-5,354) | 21(8.02-45.9) | 2,061(800-4,537) | 19.42(7.54-42.75) | -0.25(-0.27 to -0.23) | -0.45(-0.5 to -0.41) | -0.14(-0.16 to -0.11) | -0.2(-0.24 to -0.16) |
| Costa Rica | 221(86-483) | 19.69(7.65-42.99) | 197(76-437) | 19.36(7.5-42.97) | -0.06(-0.07 to -0.04) | 0.04(0.03 to 0.06) | -0.21(-0.24 to -0.19) | 0.01(-0.01 to 0.04) |
| El Salvador | 461(182-1,001) | 21.36(8.43-46.39) | 360(139-800) | 19.77(7.65-43.99) | -0.25(-0.27 to -0.23) | -0.57(-0.62 to -0.52) | -0.16(-0.18 to -0.13) | -0.08(-0.11 to -0.05) |
| Guatemala | 905(354-1,972) | 22.29(8.72-48.56) | 1,039(407-2,309) | 21.06(8.25-46.79) | -0.18(-0.2 to -0.17) | -0.41(-0.43 to -0.39) | -0.13(-0.16 to -0.11) | -0.03(-0.07 to 0.01) |
| Honduras | 485(187-1,060) | 21.97(8.44-48) | 703(276-1,567) | 21.45(8.44-47.82) | -0.08(-0.11 to -0.05) | -0.11(-0.14 to -0.07) | -0.07(-0.08 to -0.06) | -0.07(-0.14 to 0) |
| Mexico | 7,285(2,896-15,785) | 21.8(8.67-47.24) | 7,342(2,924-16,212) | 22.9(9.12-50.56) | 0.16(0.12 to 0.19) | -0.48(-0.52 to -0.45) | -0.11(-0.13 to -0.1) | 0.95(0.86 to 1.04) |
| Nicaragua | 376(147-798) | 20.63(8.10-43.83) | 394(152-879) | 19.89(7.67-44.37) | -0.12(-0.15 to -0.09) | 0.05(0.03 to 0.07) | -0.29(-0.35 to -0.23) | -0.1(-0.14 to -0.06) |
| Panama | 173(68-373) | 20.7(8.12-44.73) | 227(88-498) | 19.69(7.63-43.15) | -0.16(-0.17 to -0.15) | -0.35(-0.37 to -0.33) | -0.05(-0.07 to -0.02) | -0.11(-0.13 to -0.09) |
| Venezuela (Bolivarian Republic of) | 1,504(576-3,263) | 21.2(8.11-45.99) | 1,332(527-2,881) | 20.1(7.95-43.49) | -0.17(-0.2 to -0.14) | -0.33(-0.37 to -0.29) | -0.35(-0.41 to -0.28) | 0.09(0.05 to 0.13) |
| Brazil | 16,263(6,469-35,523) | 31.31(12.45-68.39) | 14,307(5,604-31,352) | 29.69(11.63-65.07) | -0.17(-0.19 to -0.16) | -0.29(-0.31 to -0.27) | -0.09(-0.11 to -0.06) | -0.16(-0.17 to -0.14) |
| Paraguay | 483(194-1,039) | 28.91(11.62-62.2) | 578(230-1,262) | 28.79(11.47-62.87) | -0.01(-0.02 to 0) | 0.08(0.06 to 0.09) | -0.04(-0.05 to -0.03) | -0.05(-0.07 to -0.04) |
| Algeria | 983(383-2,108) | 9.17(3.57-19.65) | 1,027(414-2,251) | 7.72(3.11-16.92) | -0.57(-0.59 to -0.54) | -0.75(-0.78 to -0.72) | -0.44(-0.49 to -0.39) | -0.55(-0.57 to -0.52) |
| Bahrain | 15(6-31) | 9.01(3.61-18.98) | 22(9-48) | 7.46(2.91-16.28) | -0.61(-0.64 to -0.58) | -0.68(-0.75 to -0.62) | -0.66(-0.72 to -0.6) | -0.49(-0.53 to -0.46) |
| Egypt | 3,144(1,259-6,749) | 14.17(5.67-30.43) | 2,835(1,134-5,813) | 7.69(3.08-15.77) | -1.97(-2.23 to -1.71) | -4.22(-4.98 to -3.45) | -0.61(-0.76 to -0.45) | -1.52(-1.82 to -1.23) |
| Iran (Islamic Republic of) | 2,429(963-5,095) | 9.57(3.79-20.07) | 1,655(654-3,651) | 8.2(3.24-18.09) | -0.5(-0.51 to -0.49) | -0.75(-0.77 to -0.73) | -0.25(-0.26 to -0.25) | -0.54(-0.57 to -0.51) |
| Iraq | 587(234-1,314) | 7.13(2.84-15.95) | 884(350-2,047) | 6.57(2.6-15.21) | -0.26(-0.29 to -0.24) | -0.07(-0.13 to -0.02) | -0.44(-0.47 to -0.42) | -0.24(-0.28 to -0.19) |
| Jordan | 146(58-308) | 8.93(3.53-18.88) | 273(107-593) | 7.53(2.93-16.32) | -0.55(-0.59 to -0.51) | -0.56(-0.62 to -0.5) | -0.78(-0.85 to -0.71) | -0.35(-0.43 to -0.28) |
| Kuwait | 45(18-97) | 8.12(3.16-17.48) | 61(24-136) | 7.24(2.78-16.1) | -0.39(-0.45 to -0.32) | -0.48(-0.68 to -0.29) | -0.27(-0.33 to -0.22) | -0.4(-0.48 to -0.33) |
| Lebanon | 89(35-191) | 8.55(3.32-18.29) | 93(36-204) | 7.28(2.84-15.93) | -0.52(-0.53 to -0.51) | -0.77(-0.78 to -0.75) | -0.46(-0.47 to -0.45) | -0.35(-0.38 to -0.33) |
| Libya | 157(63-339) | 8.66(3.47-18.72) | 116(46-256) | 7.81(3.07-17.16) | -0.34(-0.37 to -0.31) | -0.32(-0.36 to -0.27) | -0.42(-0.44 to -0.39) | -0.31(-0.39 to -0.23) |
| Morocco | 920(366-1,935) | 9.4(3.74-19.78) | 789(312-1,748) | 8.06(3.19-17.85) | -0.5(-0.52 to -0.48) | -0.55(-0.59 to -0.51) | -0.4(-0.45 to -0.36) | -0.55(-0.57 to -0.53) |
| Palestine | 82(33-175) | 8.51(3.36-18.1) | 142(55-308) | 7.58(2.94-16.48) | -0.37(-0.4 to -0.35) | -0.41(-0.46 to -0.36) | -0.25(-0.28 to -0.22) | -0.45(-0.51 to -0.39) |
| Oman | 73(29-154) | 8.71(3.43-18.29) | 92(36-200) | 7.53(2.91-16.37) | -0.47(-0.51 to -0.44) | -0.67(-0.7 to -0.64) | -0.18(-0.21 to -0.15) | -0.56(-0.65 to -0.47) |
| Qatar | 11(4-23) | 8.73(3.51-18.5) | 36(14-80) | 7.24(2.84-16.11) | -0.61(-0.64 to -0.58) | -0.38(-0.41 to -0.35) | -0.74(-0.8 to -0.67) | -0.67(-0.72 to -0.63) |
| Saudi Arabia | 622(243-1,349) | 9.49(3.71-20.58) | 588(225-1,310) | 7.78(2.98-17.31) | -0.64(-0.66 to -0.62) | -0.65(-0.67 to -0.62) | -0.7(-0.71 to -0.68) | -0.58(-0.64 to -0.52) |
| Syrian Arab Republic | 525(204-1,108) | 8.86(3.44-18.72) | 276(107-609) | 7.54(2.92-16.63) | -0.53(-0.56 to -0.49) | -0.72(-0.74 to -0.69) | -0.63(-0.71 to -0.56) | -0.32(-0.38 to -0.25) |
| Tunisia | 264(103-565) | 8.51(3.33-18.19) | 208(82-461) | 7.52(2.96-16.67) | -0.4(-0.42 to -0.38) | -0.45(-0.46 to -0.44) | -0.38(-0.39 to -0.37) | -0.38(-0.43 to -0.32) |
| Turkey | 1,944(783-4,320) | 9.49(3.82-21.08) | 1,428(552-3,104) | 7.71(2.98-16.76) | -0.68(-0.72 to -0.64) | -0.89(-0.93 to -0.85) | -1.1(-1.16 to -1.04) | -0.17(-0.27 to -0.07) |
| United Arab Emirates | 57(23-121) | 9.71(3.82-20.58) | 110(43-245) | 8.22(3.24-18.31) | -0.54(-0.57 to -0.51) | -0.42(-0.46 to -0.37) | -0.41(-0.45 to -0.37) | -0.76(-0.82 to -0.71) |
| Yemen | 660(260-1,405) | 9.31(3.67-19.8) | 1,170(466-2,545) | 8.48(3.38-18.45) | -0.31(-0.34 to -0.28) | -0.38(-0.42 to -0.34) | -0.29(-0.33 to -0.24) | -0.26(-0.31 to -0.21) |
| Afghanistan | 445(175-946) | 10.32(4.07-21.96) | 1,286(507-2,761) | 9.06(3.57-19.44) | -0.42(-0.45 to -0.39) | 0(-0.05 to 0.06) | -0.68(-0.69 to -0.67) | -0.55(-0.61 to -0.5) |
| Bangladesh | 19,295(7,208-42,629) | 39.45(14.74-87.16) | 14,554(5,753-32,690) | 31.8(12.57-71.43) | -0.7(-0.72 to -0.68) | -0.61(-0.63 to -0.6) | -0.91(-0.93 to -0.89) | -0.58(-0.62 to -0.53) |
| Bhutan | 99(39-215) | 37.69(14.7-82.13) | 59(23-132) | 31.75(12.42-70.67) | -0.56(-0.59 to -0.52) | -0.27(-0.3 to -0.23) | -0.82(-0.84 to -0.79) | -0.55(-0.62 to -0.47) |
| India | 153,101(58,158-338,268) | 46.89(17.81-103.6) | 150,015(57,791-328,504) | 40.94(15.77-89.66) | -0.45(-0.47 to -0.42) | -0.24(-0.26 to -0.21) | -0.58(-0.59 to -0.57) | -0.51(-0.57 to -0.45) |
| Nepal | 3,220(1,201-7,208) | 38.22(14.26-85.55) | 3,045(1,198-6,923) | 33(12.98-75.03) | -0.48(-0.51 to -0.45) | -0.68(-0.73 to -0.63) | -0.41(-0.47 to -0.35) | -0.34(-0.36 to -0.32) |
| Pakistan | 19,080(7,373-42,264) | 38.75(14.97-85.83) | 32,413(12,278-70,653) | 37.94(14.37-82.69) | -0.07(-0.1 to -0.04) | 0.41(0.37 to 0.44) | -0.41(-0.51 to -0.32) | -0.19(-0.21 to -0.16) |
| Angola | 6,754(2,551-14,904) | 143.25(54.11-316.12) | 17,781(6,805-40,498) | 116.63(44.63-265.64) | -0.67(-0.71 to -0.63) | -0.09(-0.13 to -0.05) | -0.79(-0.81 to -0.77) | -1.03(-1.13 to -0.93) |
| Central African Republic | 1,826(707-4,107) | 149.32(57.84-335.87) | 3,086(1,195-7,304) | 135.14(52.34-319.82) | -0.32(-0.36 to -0.28) | 0.11(0.08 to 0.14) | -0.51(-0.56 to -0.46) | -0.56(-0.66 to -0.47) |
| Congo | 1,573(621-3,511) | 149.39(58.96-333.43) | 2,288(878-5,376) | 118.61(45.5-278.65) | -0.75(-0.77 to -0.73) | -0.16(-0.18 to -0.15) | -1.2(-1.22 to -1.17) | -0.9(-0.95 to -0.86) |
| Democratic Republic of the Congo | 24,413(9,249-54,692) | 137.9(52.24-308.93) | 42,820(16,570-98,177) | 112.69(43.61-258.37) | -0.66(-0.69 to -0.63) | -0.41(-0.43 to -0.38) | -0.72(-0.73 to -0.7) | -0.83(-0.92 to -0.75) |
| Equatorial Guinea | 285(111-650) | 144.96(56.17-330.23) | 627(237-1,465) | 107.14(40.55-250.49) | -0.98(-1.02 to -0.95) | -0.5(-0.55 to -0.45) | -1.63(-1.66 to -1.6) | -0.86(-0.95 to -0.77) |
| Gabon | 554(227-1,169) | 136.01(55.59-286.82) | 679(278-1,397) | 106.23(43.46-218.58) | -0.81(-0.85 to -0.77) | -0.02(-0.07 to 0.02) | -0.77(-0.83 to -0.71) | -1.55(-1.65 to -1.44) |
| Burundi | 3,110(1,216-7,011) | 118.67(46.39-267.49) | 6,140(2,450-13,946) | 104.88(41.85-238.22) | -0.41(-0.46 to -0.36) | -0.29(-0.32 to -0.25) | -0.51(-0.54 to -0.48) | -0.41(-0.54 to -0.29) |
| Comoros | 242(96-533) | 113.67(45.14-250.49) | 253(101-562) | 105.36(41.99-234.1) | -0.27(-0.32 to -0.22) | 0.3(0.24 to 0.36) | -0.87(-0.93 to -0.8) | -0.25(-0.36 to -0.13) |
| Djibouti | 189(74-424) | 108.53(42.23-243.68) | 419(170-938) | 101.49(41.26-227.03) | -0.23(-0.27 to -0.19) | 0.36(0.28 to 0.44) | -0.13(-0.17 to -0.09) | -0.79(-0.85 to -0.72) |
| Eritrea | 1,920(754-4,307) | 120.61(47.37-270.53) | 2,864(1,127-6,346) | 113.43(44.64-251.38) | -0.24(-0.27 to -0.2) | 0.01(-0.02 to 0.03) | 0.01(-0.02 to 0.03) | -0.68(-0.76 to -0.59) |
| Ethiopia | 62,686(24,372-133,035) | 257.29(100.03-546.03) | 86,813(33,622-185,359) | 195.74(75.81-417.94) | -0.88(-0.89 to -0.87) | -0.64(-0.65 to -0.63) | -0.97(-0.99 to -0.96) | -0.98(-1.01 to -0.95) |
| Kenya | 8,527(3,333-19,128) | 76.34(29.84-171.24) | 16,245(6,396-36,630) | 87.03(34.26-196.25) | 0.42(0.24 to 0.59) | -0.04(-0.25 to 0.17) | 1.09(0.91 to 1.27) | 0.12(-0.32 to 0.56) |
| Madagascar | 4,027(1,581-8,617) | 73.82(28.98-157.95) | 8,087(3,202-16,984) | 68.92(27.29-144.75) | -0.25(-0.29 to -0.21) | -0.5(-0.59 to -0.42) | -0.06(-0.1 to -0.02) | -0.23(-0.31 to -0.15) |
| Malawi | 5,065(1,957-11,326) | 111.33(43.01-248.95) | 8,351(3,306-18,812) | 102.79(40.69-231.56) | -0.3(-0.38 to -0.21) | -0.26(-0.32 to -0.21) | 0.04(-0.1 to 0.18) | -0.61(-0.81 to -0.41) |
| Mauritius | 111(44-244) | 33.62(13.22-73.89) | 69(27-152) | 33.41(13.18-73.43) | -0.03(-0.06 to 0.01) | -0.38(-0.45 to -0.32) | 0.29(0.22 to 0.36) | -0.02(-0.07 to 0.02) |
| Mozambique | 5,729(2,334-11,865) | 92.34(37.62-191.25) | 12,923(5,181-27,059) | 90.59(36.32-189.68) | -0.1(-0.18 to -0.03) | -0.51(-0.6 to -0.43) | 0.6(0.41 to 0.79) | -0.35(-0.46 to -0.25) |
| Rwanda | 6,920(2,834-14,942) | 203.96(83.52-440.39) | 7,041(2,897-14,910) | 141.66(58.28-299.97) | -1.24(-1.35 to -1.13) | -0.73(-0.85 to -0.62) | -2.16(-2.47 to -1.85) | -0.81(-0.91 to -0.71) |
| Seychelles | 8(3-18) | 34.04(13.54-74.2) | 8(3-17) | 32.97(13.17-71.39) | -0.1(-0.13 to -0.07) | -0.03(-0.07 to 0) | -0.36(-0.43 to -0.3) | 0.06(0.02 to 0.09) |
| Somalia | 4,289(1,679-9,670) | 110.11(43.11-248.22) | 10,568(4,152-23,834) | 102.31(40.19-230.74) | -0.26(-0.29 to -0.24) | -0.19(-0.26 to -0.13) | 0.06(0.03 to 0.1) | -0.65(-0.69 to -0.6) |
| United Republic of Tanzania | 11,368(4,483-23,272) | 94.14(37.13-192.72) | 26,792(10,478-57,213) | 109.79(42.94-234.45) | 0.46(0.32 to 0.61) | 2.47(1.98 to 2.96) | -0.15(-0.19 to -0.1) | -0.54(-0.65 to -0.43) |
| Uganda | 8,462(3,399-19,069) | 100.5(40.37-226.49) | 19,776(7,961-44,176) | 99.7(40.13-222.71) | -0.08(-0.19 to 0.02) | 0.51(0.39 to 0.64) | -0.2(-0.29 to -0.11) | -0.45(-0.71 to -0.18) |
| Zambia | 4,405(1,747-10,081) | 117.33(46.53-268.5) | 8,817(3,502-19,773) | 106.6(42.34-239.05) | -0.33(-0.34 to -0.31) | 0.1(0.09 to 0.11) | -0.1(-0.12 to -0.09) | -0.88(-0.92 to -0.84) |
| Botswana | 270(107-586) | 45.81(18.04-99.21) | 314(125-683) | 44.93(17.84-97.79) | -0.15(-0.23 to -0.07) | 0.52(0.38 to 0.66) | -0.22(-0.39 to -0.05) | -0.69(-0.81 to -0.56) |
| Lesotho | 308(120-684) | 45.14(17.59-100.21) | 309(121-687) | 49.04(19.26-108.98) | 0.22(0.15 to 0.29) | 0.42(0.32 to 0.51) | 0.75(0.6 to 0.91) | -0.46(-0.56 to -0.35) |
| Namibia | 279(109-620) | 46.48(18.15-103.28) | 355(139-778) | 42.95(16.88-94.21) | -0.3(-0.36 to -0.25) | 0.07(-0.04 to 0.18) | -0.29(-0.4 to -0.18) | -0.65(-0.75 to -0.56) |
| South Africa | 6,106(2,421-13,232) | 44.85(17.79-97.2) | 6,693(2,653-14,414) | 44.02(17.45-94.8) | -0.08(-0.14 to -0.02) | 0.59(0.52 to 0.66) | -0.2(-0.25 to -0.15) | -0.51(-0.66 to -0.37) |
| Eswatini | 173(68-384) | 44.9(17.76-99.52) | 191(76-415) | 46.26(18.31-100.55) | 0.02(-0.03 to 0.06) | 0.41(0.36 to 0.45) | 0.41(0.36 to 0.45) | -0.69(-0.79 to -0.58) |
| Zimbabwe | 2,164(843-4,686) | 44.93(17.5-97.3) | 2,905(1,140-6,231) | 46.15(18.11-99) | 0.03(-0.04 to 0.1) | -0.17(-0.28 to -0.05) | 0.59(0.41 to 0.76) | -0.23(-0.31 to -0.15) |
| Benin | 2,430(938-5,462) | 100.35(38.73-225.56) | 5,436(2,147-12,272) | 89.4(35.31-201.84) | -0.41(-0.46 to -0.35) | -0.19(-0.25 to -0.12) | -0.8(-0.9 to -0.71) | -0.33(-0.44 to -0.22) |
| Burkina Faso | 4,379(1,723-9,745) | 92.78(36.52-206.5) | 9,189(3,638-20,216) | 88.6(35.07-194.9) | -0.18(-0.23 to -0.12) | -0.19(-0.27 to -0.12) | -0.56(-0.63 to -0.5) | 0.12(0.02 to 0.23) |
| Cameroon | 3,786(1,478-8,473) | 77.55(30.27-173.56) | 9,056(3,666-19,606) | 67.25(27.22-145.59) | -0.48(-0.58 to -0.38) | -0.17(-0.36 to 0.02) | -0.67(-0.75 to -0.59) | -0.66(-0.87 to -0.46) |
| Cabo Verde | 131(50-296) | 83.05(31.93-188.34) | 107(42-235) | 74.88(29.42-163.99) | -0.36(-0.39 to -0.32) | 0.1(0.03 to 0.16) | -0.9(-0.95 to -0.86) | -0.27(-0.34 to -0.2) |
| Chad | 2,798(1,091-6,199) | 95.6(37.3-211.83) | 8,270(3,226-18,241) | 91.74(35.79-202.35) | -0.14(-0.18 to -0.11) | 0.5(0.42 to 0.57) | -0.36(-0.43 to -0.29) | -0.53(-0.57 to -0.49) |
| Coted'Ivoire | 5,218(2,125-11,171) | 91.5(37.26-195.88) | 10,749(4,409-23,238) | 92.89(38.1-200.82) | 0.11(-0.04 to 0.26) | 0.65(0.5 to 0.8) | -1.44(-1.64 to -1.23) | 0.93(0.58 to 1.29) |
| Gambia | 430(168-958) | 93.19(36.33-207.67) | 876(343-1,969) | 88.17(34.52-198.22) | -0.19(-0.23 to -0.15) | 0.06(0 to 0.13) | -0.42(-0.53 to -0.32) | -0.22(-0.26 to -0.18) |
| Ghana | 4,660(1,915-9,573) | 69.38(28.51-142.53) | 8,202(3,389-16,798) | 63.66(26.31-130.39) | -0.3(-0.4 to -0.2) | -0.52(-0.63 to -0.41) | -0.09(-0.28 to 0.11) | -0.34(-0.52 to -0.15) |
| Guinea | 2,793(1,107-6,302) | 101.5(40.23-229.01) | 5,747(2,250-12,664) | 95.05(37.21-209.48) | -0.23(-0.28 to -0.19) | 0.01(-0.06 to 0.08) | -0.47(-0.53 to -0.41) | -0.29(-0.38 to -0.19) |
| Guinea-Bissau | 545(211-1,244) | 112.97(43.74-257.89) | 922(366-2,071) | 102.69(40.7-230.62) | -0.33(-0.38 to -0.29) | 0.06(0.02 to 0.09) | -0.61(-0.65 to -0.58) | -0.41(-0.53 to -0.29) |
| Liberia | 1,159(451-2,594) | 102.59(39.95-229.52) | 1,881(734-4,186) | 86.06(33.56-191.52) | -0.61(-0.76 to -0.47) | -0.88(-1.24 to -0.52) | -0.96(-1.14 to -0.77) | -0.16(-0.37 to 0.06) |
| Mali | 7,656(2,954-16,757) | 185.38(71.53-405.76) | 21,243(8,155-45,203) | 183.51(70.45-390.5) | -0.05(-0.12 to 0.03) | 0.38(0.15 to 0.62) | -0.3(-0.36 to -0.23) | -0.14(-0.22 to -0.06) |
| Mauritania | 970(378-2,223) | 104.96(40.86-240.48) | 1,551(606-3,497) | 83.69(32.69-188.72) | -0.75(-0.81 to -0.69) | -0.75(-0.77 to -0.74) | -0.75(-0.77 to -0.74) | -0.74(-0.9 to -0.59) |
| Niger | 4,856(1,949-10,391) | 119.51(47.98-255.75) | 12,881(5,216-27,674) | 100.92(40.86-216.81) | -0.55(-0.59 to -0.5) | -0.58(-0.61 to -0.55) | -1.39(-1.51 to -1.27) | 0.11(0.06 to 0.16) |
| Nigeria | 45,290(18,055-99,382) | 115.76(46.15-254.01) | 122,148(48,591-267,210) | 120.24(47.83-263.03) | 0.09(0.03 to 0.15) | 1(0.88 to 1.12) | -0.36(-0.43 to -0.29) | -0.23(-0.35 to -0.11) |
| Sao Tome and Principe | 55(21-120) | 96.24(37.06-212.6) | 65(25-148) | 83.5(32.59-190.04) | -0.48(-0.53 to -0.43) | -0.33(-0.41 to -0.26) | -0.6(-0.65 to -0.54) | -0.54(-0.65 to -0.44) |
| Senegal | 3,559(1,391-7,838) | 97.48(38.11-214.68) | 5,704(2,177-13,023) | 89.67(34.23-204.74) | -0.29(-0.33 to -0.24) | -0.52(-0.6 to -0.44) | -0.31(-0.37 to -0.26) | -0.04(-0.12 to 0.05) |
| Sierra Leone | 1,764(684-3,948) | 97.29(37.73-217.83) | 3,217(1,256-7,382) | 89.97(35.11-206.41) | -0.26(-0.3 to -0.22) | -0.11(-0.15 to -0.06) | -0.19(-0.2 to -0.17) | -0.46(-0.56 to -0.36) |
| Togo | 1,735(671-3,955) | 98.45(38.09-224.41) | 3,033(1,210-7,029) | 91.67(36.55-212.4) | -0.25(-0.28 to -0.23) | 0.18(0.16 to 0.19) | -0.68(-0.71 to -0.66) | -0.3(-0.37 to -0.24) |
| American Samoa | 4(2-9) | 21.22(8.38-45.73) | 3(1-7) | 22.14(8.58-48.97) | 0.14(0.11 to 0.16) | -0.03(-0.05 to -0.01) | 0.26(0.21 to 0.31) | 0.2(0.14 to 0.25) |
| Bermuda | 3(1-7) | 28.06(11.17-61.07) | 2(1-5) | 27.16(10.78-59.18) | -0.1(-0.12 to -0.09) | -0.31(-0.34 to -0.27) | -0.14(-0.16 to -0.12) | 0.08(0.05 to 0.1) |
| Cook Islands | 1(1-3) | 20.91(8.26-46.34) | 1(0-2) | 19.95(7.64-43.39) | -0.16(-0.18 to -0.13) | -0.37(-0.38 to -0.35) | -0.06(-0.11 to -0.01) | -0.03(-0.08 to 0.02) |
| Greenland | 1(1-3) | 9.48(3.68-20.95) | 1(0-2) | 8.61(3.33-18.76) | -0.31(-0.37 to -0.26) | -0.26(-0.41 to -0.11) | -0.53(-0.58 to -0.48) | -0.15(-0.23 to -0.07) |
| Guam | 8(3-18) | 19.89(7.80-44.03) | 7(3-16) | 20.32(7.96-44.93) | 0.07(0.03 to 0.1) | -0.16(-0.27 to -0.05) | 0.26(0.23 to 0.3) | 0.05(0.01 to 0.09) |
| Monaco | 1(0-2) | 24.85(9.88-54.52) | 1(0-3) | 24.19(9.51-52.4) | -0.09(-0.1 to -0.07) | -0.1(-0.13 to -0.07) | -0.07(-0.09 to -0.05) | -0.09(-0.11 to -0.07) |
| Nauru | 1(0-2) | 22.46(8.79-49.43) | 1(0-2) | 22.20(8.62-48.66) | -0.04(-0.06 to -0.02) | 0.24(0.22 to 0.26) | 0(-0.03 to 0.03) | -0.3(-0.34 to -0.27) |
| Niue | 0(0-0) | 21.86(8.58-47.25) | 0(0-0) | 21.29(8.28-45.8) | -0.09(-0.11 to -0.06) | -0.02(-0.09 to 0.06) | -0.28(-0.31 to -0.25) | -0.01(-0.04 to 0.02) |
| Northern Mariana Islands | 2(1-5) | 20.25(7.92-44.3) | 2(1-5) | 21.02(8.1-47.43) | 0.11(0 to 0.21) | -0.48(-0.76 to -0.21) | 0.73(0.54 to 0.93) | 0.13(0.05 to 0.2) |
| Palau | 1(0-2) | 21.89(8.52-48.96) | 1(0-2) | 21.58(8.35-47.55) | -0.05(-0.06 to -0.03) | -0.29(-0.33 to -0.24) | -0.03(-0.05 to -0.01) | 0.13(0.1 to 0.16) |
| Puerto Rico | 284(112-613) | 28.52(11.28-61.6) | 126(49-278) | 28.4(11.1-62.45) | -0.01(-0.04 to 0.01) | -0.18(-0.2 to -0.16) | -0.12(-0.15 to -0.1) | 0.2(0.15 to 0.25) |
| Saint Kitts and Nevis | 4(2-10) | 31.26(12.48-68.07) | 3(1-6) | 29.48(11.66-63.72) | -0.19(-0.2 to -0.18) | -0.39(-0.4 to -0.37) | -0.11(-0.15 to -0.07) | -0.09(-0.11 to -0.08) |
| San Marino | 1(0-2) | 25.2(9.91-54.67) | 1(0-2) | 24.72(9.67-54.27) | -0.06(-0.08 to -0.04) | -0.45(-0.48 to -0.42) | 0(-0.03 to 0.04) | 0.19(0.16 to 0.21) |
| Tokelau | 0(0-0) | 22.14(8.63-49.08) | 0(0-0) | 21.54(8.24-48.48) | -0.09(-0.13 to -0.06) | -0.05(-0.13 to 0.04) | -0.18(-0.23 to -0.13) | -0.08(-0.1 to -0.06) |
| Tuvalu | 1(0-2) | 22.48(9.04-48.4) | 1(0-2) | 21.81(8.57-48.04) | -0.1(-0.12 to -0.08) | -0.07(-0.1 to -0.03) | -0.08(-0.14 to -0.02) | -0.15(-0.18 to -0.13) |
| United States Virgin Islands | 9(4-20) | 29.27(11.52-63.43) | 4(2-9) | 29.23(11.61-63.63) | 0(-0.01 to 0) | -0.1(-0.11 to -0.09) | 0(-0.01 to 0.01) | 0.08(0.07 to 0.09) |
| South Sudan | 2,872(1,140-6,328) | 109.45(43.44-241.16) | 4,160(1,628-9,118) | 96.86(37.9-212.29) | -0.42(-0.48 to -0.37) | -0.3(-0.37 to -0.23) | -0.09(-0.22 to 0.04) | -0.82(-0.91 to -0.73) |
| Sudan | 843(337-1,767) | 9.48(3.79-19.87) | 1,388(533-3,124) | 8.37(3.21-18.83) | -0.41(-0.45 to -0.37) | -0.54(-0.62 to -0.46) | -0.23(-0.26 to -0.2) | -0.47(-0.55 to -0.4) |

**sTable8 Number of DALYs and DALYs rate of viral skin diseases and their AAPCs from 1990 to 2021 at the global, regional and national levels**

| *Rate per 100,000* | 1990 | | 2021 | | 1990-2021 | 1990-1999 | 2000-2009 | 2010-2021 |
| --- | --- | --- | --- | --- | --- | --- | --- | --- |
| *AAPC(95% CI)* | Number of DALYs | DALYs rate | Number of DALYs | DALYs rate | AAPC | AAPC | AAPC | AAPC |
| Viral skin diseases | 1,455,375(923,193-2,208,726) | 83.68(53.08-127) | 1,841,264(1,166,908-2,782,549) | 91.52(58-138.31) | 0.29(0.28 to 0.3) | 0.25(0.23 to 0.27) | 0.11(0.09 to 0.14) | 0.48(0.47 to 0.49) |
| Male | 774,940(489,920-1,176,613) | 86.73(54.83-131.69) | 985,372(621,328-1,490,079) | 94.91(59.85-143.53) | 0.29(0.28 to 0.3) | 0.27(0.25 to 0.29) | 0.07(0.04 to 0.1) | 0.51(0.5 to 0.52) |
| Female | 680,436(432,337-1,032,113) | 80.46(51.12-122.05) | 855,891(542,597-1,297,599) | 87.9(55.73-133.27) | 0.29(0.28 to 0.29) | 0.21(0.2 to 0.22) | 0.18(0.18 to 0.18) | 0.44(0.42 to 0.45) |
| **Age groups** |  |  |  |  |  |  |  |  |
| 0-4 years | 351,992(222,665-528,297) | 56.78(35.92-85.22) | 427,817(269,296-643,243) | 65(40.92-97.73) | 0.44(0.4 to 0.48) | 0.26(0.19 to 0.32) | 0.33(0.3 to 0.36) | 0.68(0.59 to 0.77) |
| 5-9 years | 614,118(376,379-934,955) | 105.24(64.5-160.22) | 789,981(484,644-1,201,116) | 114.98(70.54-174.82) | 0.29(0.28 to 0.3) | 0.23(0.21 to 0.25) | 0.24(0.24 to 0.25) | 0.38(0.36 to 0.4) |
| 10-14 years | 489,265(311,616-740,129) | 91.33(58.17-138.17) | 623,466(398,705-941,091) | 93.52(59.81-141.17) | 0.07(0.06 to 0.09) | -0.03(-0.04 to -0.01) | 0.05(0.03 to 0.06) | 0.16(0.14 to 0.19) |
| **SDI region** |  |  |  |  |  |  |  |  |
| High SDI | 279,623(176,683-430,220) | 150.49(95.09-231.54) | 275,809(174,924-421,804) | 159.86(101.38-244.47) | 0.2(0.18 to 0.21) | 0.31(0.28 to 0.35) | 0.09(0.06 to 0.11) | 0.19(0.17 to 0.21) |
| High-middle SDI | 248,596(156,407-378,047) | 90.85(57.16-138.16) | 239,416(150,136-364,552) | 103.69(65.02-157.89) | 0.43(0.42 to 0.44) | 0.51(0.48 to 0.54) | 0.24(0.23 to 0.25) | 0.54(0.52 to 0.55) |
| Middle SDI | 424,587(269,769-640,047) | 73.56(46.74-110.89) | 477,301(302,428-722,228) | 84.2(53.35-127.41) | 0.44(0.42 to 0.46) | 0.43(0.39 to 0.48) | 0.1(0.06 to 0.14) | 0.74(0.73 to 0.76) |
| Low-middle SDI | 316,811(202,171-474,662) | 67.11(42.82-100.54) | 449,837(284,709-679,989) | 77.58(49.1-117.27) | 0.47(0.46 to 0.48) | 0.35(0.34 to 0.36) | 0.41(0.4 to 0.43) | 0.62(0.6 to 0.63) |
| Low SDI | 184,649(116,553-281,459) | 80.66(50.92-122.95) | 397,648(250,998-604,562) | 86.40(54.54-131.36) | 0.22(0.21 to 0.23) | 0.07(0.05 to 0.09) | 0.24(0.23 to 0.25) | 0.33(0.32 to 0.34) |
| **GBD region** |  |  |  |  |  |  |  |  |
| Andean Latin America | 9,531(6,027-14,384) | 64.17(40.58-96.85) | 13,106(8,331-19,658) | 72.43(46.04-108.64) | 0.39(0.37 to 0.41) | 0.34(0.31 to 0.36) | 0.36(0.33 to 0.38) | 0.47(0.42 to 0.53) |
| Australasia | 7,796(4,954-12,004) | 169.99(108.02-261.74) | 10,298(6,462-15,781) | 179.69(112.75-275.35) | 0.18(0.14 to 0.22) | 0.27(0.23 to 0.31) | -0.07(-0.17 to 0.03) | 0.3(0.26 to 0.34) |
| Caribbean | 6,442(4,066-9,759) | 56.44(35.63-85.52) | 7,134(4,455-10,866) | 62.01(38.72-94.44) | 0.3(0.28 to 0.32) | 0.29(0.27 to 0.32) | 0.31(0.27 to 0.34) | 0.32(0.29 to 0.36) |
| Central Asia | 17,676(11,001-26,869) | 70.73(44.02-107.51) | 21,752(13,591-33,072) | 78.59(49.11-119.5) | 0.34(0.31 to 0.37) | 0.56(0.53 to 0.59) | -0.08(-0.11 to -0.05) | 0.53(0.47 to 0.59) |
| Central Europe | 23,869(15,161-35,840) | 80.96(51.42-121.56) | 16,006(10,161-24,042) | 90.42(57.41-135.82) | 0.36(0.34 to 0.38) | 0.57(0.51 to 0.62) | 0.12(0.11 to 0.13) | 0.37(0.33 to 0.41) |
| Central Latin America | 30,727(19,330-46,377) | 47.73(30.02-72.03) | 35,696(22,428-54,382) | 56.23(35.33-85.66) | 0.53(0.51 to 0.55) | 0.5(0.49 to 0.51) | 0.39(0.36 to 0.41) | 0.68(0.64 to 0.72) |
| Central Sub-Saharan Africa | 12,623(8,036-18,933) | 49.89(31.76-74.84) | 32,151(20,115-47,758) | 54.79(34.28-81.38) | 0.3(0.28 to 0.33) | -0.03(-0.08 to 0.01) | 0.19(0.14 to 0.24) | 0.7(0.66 to 0.73) |
| East Asia | 301,780(190,018-457,813) | 91.49(57.61-138.8) | 287,039(179,964-437,888) | 107.36(67.31-163.79) | 0.51(0.49 to 0.54) | 0.7(0.65 to 0.76) | 0.21(0.19 to 0.22) | 0.64(0.6 to 0.67) |
| Eastern Europe | 39,734(24,881-60,455) | 77.21(48.35-117.47) | 31,129(19,647-46,906) | 87.82(55.43-132.34) | 0.42(0.36 to 0.48) | 0.42(0.26 to 0.59) | 0.01(-0.03 to 0.05) | 0.83(0.74 to 0.93) |
| Eastern Sub-Saharan Africa | 110,515(70,189-168,264) | 122.02(77.5-185.78) | 231,510(146,282-354,361) | 129.75(81.98-198.6) | 0.2(0.18 to 0.21) | 0.06(0.03 to 0.09) | 0.22(0.21 to 0.23) | 0.29(0.27 to 0.32) |
| High-income Asia Pacific | 62,224(39,066-95,818) | 176.78(110.98-272.21) | 41,364(25,905-63,640) | 184.45(115.52-283.79) | 0.13(0.1 to 0.17) | -0.12(-0.18 to -0.07) | 0.27(0.24 to 0.31) | 0.26(0.19 to 0.32) |
| High-income North America | 112,973(72,021-169,841) | 183.17(116.77-275.37) | 126,100(80,606-189,270) | 192.17(122.84-288.44) | 0.16(0.14 to 0.17) | 0.3(0.27 to 0.33) | -0.07(-0.1 to -0.04) | 0.23(0.21 to 0.24) |
| North Africa and Middle East | 81,812(52,429-123,629) | 58.24(37.32-88) | 126,361(80,403-192,114) | 68.93(43.86-104.8) | 0.54(0.53 to 0.55) | 0.38(0.36 to 0.4) | 0.49(0.48 to 0.5) | 0.73(0.71 to 0.75) |
| Oceania | 2,058(1,311-3,122) | 76.8(48.93-116.51) | 4,095(2,532-6,253) | 80.59(49.83-123.08) | 0.16(0.15 to 0.17) | 0.17(0.16 to 0.19) | 0.06(0.04 to 0.08) | 0.24(0.23 to 0.26) |
| South Asia | 300,359(191,459-450,628) | 69.31(44.18-103.98) | 412,452(262,267-624,797) | 81.35(51.73-123.23) | 0.52(0.51 to 0.53) | 0.32(0.31 to 0.33) | 0.47(0.45 to 0.49) | 0.72(0.7 to 0.73) |
| Southeast Asia | 117,692(74,616-177,459) | 68.93(43.7-103.93) | 134,093(84,860-203,703) | 77.67(49.15-117.98) | 0.39(0.38 to 0.4) | 0.38(0.37 to 0.39) | 0.27(0.26 to 0.28) | 0.5(0.48 to 0.51) |
| Southern Latin America | 24,672(15,458-37,675) | 165.29(103.56-252.4) | 25,940(16,333-39,786) | 178.95(112.67-274.47) | 0.26(0.24 to 0.29) | 0.32(0.31 to 0.33) | 0.08(0.03 to 0.13) | 0.4(0.33 to 0.46) |
| Southern Sub-Saharan Africa | 14,643(9,262-22,361) | 70.78(44.77-108.08) | 18,202(11,484-27,471) | 75.64(47.72-114.15) | 0.21(0.2 to 0.23) | 0.29(0.28 to 0.3) | -0.03(-0.05 to -0.02) | 0.37(0.33 to 0.4) |
| Tropical Latin America | 30,617(19,474-46,522) | 57.11(36.32-86.77) | 32,032(20,301-48,854) | 63.82(40.45-97.33) | 0.36(0.35 to 0.37) | 0.2(0.16 to 0.23) | 0.34(0.32 to 0.36) | 0.51(0.5 to 0.52) |
| Western Europe | 95,106(59,836-146,508) | 133.92(84.25-206.3) | 94,549(60,116-144,199) | 138.8(88.25-211.69) | 0.12(0.1 to 0.13) | 0.15(0.14 to 0.17) | -0.08(-0.08 to -0.07) | 0.25(0.22 to 0.28) |
| Western Sub-Saharan Africa | 52,528(32,900-79,519) | 59.77(37.44-90.49) | 140,255(88,555-211,648) | 65.31(41.23-98.55) | 0.28(0.27 to 0.3) | 0.08(0.07 to 0.09) | 0.25(0.21 to 0.28) | 0.49(0.47 to 0.5) |
| **204 countries and territories** |  |  |  |  |  |  |  |  |
| China | 289,888(182,474-439,739) | 91.05(57.31-138.12) | 278,239(174,378-424,714) | 107.17(67.16-163.58) | 0.52(0.5 to 0.55) | 0.71(0.66 to 0.77) | 0.19(0.18 to 0.21) | 0.66(0.63 to 0.69) |
| Democratic People's Republic of Korea | 5,606(3,455-8,517) | 94.23(58.08-143.16) | 4,963(3,089-7,537) | 103.97(64.7-157.88) | 0.32(0.3 to 0.33) | 0.34(0.29 to 0.38) | 0.33(0.32 to 0.33) | 0.28(0.28 to 0.29) |
| Taiwan (Province of China) | 6,286(3,952-9,707) | 114.13(71.75-176.24) | 3,837(2,428-5,831) | 130.22(82.4-197.9) | 0.42(0.39 to 0.46) | 0.62(0.54 to 0.7) | 0.45(0.4 to 0.5) | 0.24(0.2 to 0.29) |
| Cambodia | 2,920(1,853-4,424) | 62.65(39.75-94.92) | 3,679(2,334-5,688) | 71.9(45.62-111.16) | 0.45(0.43 to 0.46) | 0.61(0.58 to 0.64) | 0.26(0.24 to 0.28) | 0.51(0.49 to 0.53) |
| Indonesia | 47,302(29,970-71,783) | 69.83(44.24-105.97) | 52,938(33,444-79,947) | 78.67(49.7-118.81) | 0.39(0.37 to 0.4) | 0.32(0.3 to 0.35) | 0.3(0.27 to 0.34) | 0.5(0.48 to 0.51) |
| Lao People's Democratic Republic | 1,160(733-1,786) | 62.96(39.76-96.88) | 1,655(1,051-2,505) | 72.05(45.76-109.09) | 0.43(0.42 to 0.45) | 0.35(0.33 to 0.38) | 0.5(0.49 to 0.52) | 0.45(0.41 to 0.48) |
| Malaysia | 4,729(3,016-7,095) | 71.95(45.89-107.94) | 6,232(3,957-9,500) | 81.86(51.98-124.77) | 0.42(0.39 to 0.45) | 0.5(0.42 to 0.58) | 0.42(0.4 to 0.43) | 0.34(0.33 to 0.36) |
| Maldives | 67(42-102) | 64.25(40.35-97.25) | 78(49-119) | 77.78(49.38-118.62) | 0.62(0.58 to 0.67) | 1.04(1.02 to 1.07) | 0.26(0.18 to 0.34) | 0.61(0.55 to 0.68) |
| Myanmar | 9,499(5,923-14,598) | 64.28(40.08-98.8) | 11,439(7,245-17,400) | 73.26(46.4-111.43) | 0.42(0.4 to 0.45) | 0.21(0.12 to 0.3) | 0.51(0.5 to 0.52) | 0.51(0.5 to 0.52) |
| Philippines | 17,444(11,017-26,332) | 69.18(43.69-104.43) | 25,923(16,488-39,189) | 76.25(48.5-115.27) | 0.32(0.31 to 0.33) | 0.29(0.28 to 0.3) | 0.16(0.14 to 0.18) | 0.48(0.47 to 0.5) |
| Sri Lanka | 4,013(2,547-6,070) | 72.53(46.02-109.71) | 4,170(2,629-6,321) | 81.7(51.51-123.84) | 0.39(0.35 to 0.43) | 0.31(0.22 to 0.4) | 0.25(0.16 to 0.34) | 0.58(0.56 to 0.61) |
| Thailand | 12,034(7,754-18,494) | 71.38(46-109.7) | 7,838(4,937-11,770) | 80.25(50.55-120.52) | 0.38(0.37 to 0.4) | 0.45(0.43 to 0.47) | 0.24(0.21 to 0.28) | 0.44(0.42 to 0.45) |
| Timor-Leste | 206(129-310) | 61.93(38.86-93.11) | 377(238-575) | 72.46(45.67-110.41) | 0.51(0.48 to 0.55) | 0.51(0.46 to 0.57) | 0.81(0.72 to 0.89) | 0.28(0.24 to 0.31) |
| Viet Nam | 17,891(11,341-26,976) | 67.48(42.78-101.75) | 19,391(12,206-29,760) | 78.31(49.29-120.18) | 0.48(0.45 to 0.51) | 0.58(0.55 to 0.62) | 0.17(0.12 to 0.21) | 0.67(0.65 to 0.7) |
| Fiji | 235(146-357) | 83.42(52.02-126.87) | 247(155-377) | 90.56(56.94-138.38) | 0.26(0.25 to 0.28) | 0.22(0.2 to 0.24) | 0.06(0.03 to 0.08) | 0.49(0.46 to 0.51) |
| Kiribati | 23(14-35) | 77.05(47.75-117.15) | 36(22-54) | 84.56(52.67-129.68) | 0.31(0.28 to 0.33) | 0.43(0.4 to 0.46) | 0.15(0.1 to 0.2) | 0.36(0.34 to 0.38) |
| Marshall Islands | 18(11-27) | 80.31(50.51-123.71) | 15(9-23) | 86.18(53.68-130.93) | 0.22(0.2 to 0.24) | 0.11(0.06 to 0.15) | 0.2(0.16 to 0.24) | 0.34(0.32 to 0.37) |
| Micronesia (Federated States of) | 37(23-56) | 80.97(51.07-122.49) | 27(17-40) | 87.68(54.83-132.22) | 0.26(0.25 to 0.27) | 0.33(0.3 to 0.36) | 0.25(0.24 to 0.25) | 0.21(0.2 to 0.23) |
| Papua New Guinea | 1,274(813-1,931) | 74.91(47.84-113.59) | 3,102(1,918-4,708) | 79.2(48.98-120.2) | 0.19(0.17 to 0.2) | 0.21(0.19 to 0.23) | 0.09(0.07 to 0.11) | 0.25(0.23 to 0.27) |
| Samoa | 59(36-90) | 82.63(50.48-126) | 71(44-108) | 88.34(55.39-135.57) | 0.22(0.21 to 0.23) | 0.24(0.22 to 0.26) | 0.17(0.16 to 0.19) | 0.24(0.22 to 0.26) |
| Solomon Islands | 116(72-177) | 74.45(46.18-113.85) | 209(129-320) | 80.21(49.8-123.03) | 0.24(0.23 to 0.25) | 0.13(0.12 to 0.14) | 0.2(0.18 to 0.21) | 0.35(0.33 to 0.38) |
| Tonga | 34(21-52) | 81.91(50.48-125.46) | 35(22-53) | 88.63(55.71-135.53) | 0.26(0.23 to 0.28) | 0.21(0.2 to 0.22) | 0.18(0.12 to 0.25) | 0.35(0.33 to 0.38) |
| Vanuatu | 52(32-80) | 76.22(47.7-117.33) | 97(60-147) | 83(51.26-126.29) | 0.28(0.27 to 0.29) | 0.2(0.19 to 0.21) | 0.22(0.21 to 0.23) | 0.39(0.36 to 0.41) |
| Armenia | 739(465-1,118) | 70.87(44.59-107.18) | 488(308-744) | 82.37(51.95-125.64) | 0.49(0.45 to 0.52) | 0.82(0.75 to 0.9) | -0.03(-0.06 to 0) | 0.69(0.63 to 0.75) |
| Azerbaijan | 1,759(1,104-2,678) | 72.49(45.5-110.34) | 1,947(1,211-2,932) | 82.48(51.3-124.2) | 0.42(0.38 to 0.45) | 0.57(0.53 to 0.62) | -0.07(-0.13 to -0.02) | 0.72(0.66 to 0.78) |
| Georgia | 1,055(665-1,608) | 77.07(48.62-117.51) | 610(380-934) | 82.87(51.57-126.91) | 0.23(0.15 to 0.31) | 0.3(0.19 to 0.4) | -0.14(-0.19 to -0.1) | 0.57(0.37 to 0.76) |
| Kazakhstan | 3,826(2,397-5,847) | 73.63(46.12-112.53) | 4,435(2,773-6,776) | 81.72(51.1-124.86) | 0.34(0.3 to 0.38) | 0.7(0.6 to 0.8) | -0.33(-0.38 to -0.28) | 0.59(0.55 to 0.64) |
| Kyrgyzstan | 1,191(752-1,813) | 71(44.82-108.09) | 1,768(1,108-2,691) | 77.72(48.73-118.32) | 0.3(0.23 to 0.36) | 0.53(0.45 to 0.61) | -0.35(-0.39 to -0.32) | 0.71(0.55 to 0.87) |
| Mongolia | 609(386-928) | 67.63(42.89-103.12) | 840(527-1,270) | 77.33(48.54-116.9) | 0.43(0.4 to 0.47) | 0.8(0.77 to 0.83) | -0.2(-0.24 to -0.17) | 0.78(0.68 to 0.88) |
| Tajikistan | 1,561(993-2,406) | 67.21(42.74-103.63) | 2,646(1,650-4,006) | 73.83(46.03-111.76) | 0.31(0.25 to 0.37) | 0.32(0.16 to 0.49) | 0.13(0.05 to 0.21) | 0.52(0.48 to 0.55) |
| Turkmenistan | 1,065(657-1,612) | 70.95(43.76-107.38) | 1,214(778-1,831) | 79.68(51.03-120.13) | 0.37(0.35 to 0.4) | 0.4(0.36 to 0.44) | 0.3(0.3 to 0.31) | 0.42(0.35 to 0.48) |
| Uzbekistan | 5,871(3,670-9,056) | 68.62(42.9-105.84) | 7,804(4,891-11,931) | 77.33(48.46-118.23) | 0.39(0.36 to 0.42) | 0.74(0.72 to 0.77) | 0.06(-0.02 to 0.14) | 0.43(0.41 to 0.44) |
| Albania | 852(524-1,270) | 76.28(46.94-113.68) | 384(244-588) | 86.49(54.9-132.55) | 0.42(0.39 to 0.45) | 0.56(0.53 to 0.6) | 0.45(0.38 to 0.52) | 0.29(0.24 to 0.33) |
| Bosnia and Herzegovina | 838(525-1,271) | 76.49(47.89-116.02) | 436(270-660) | 88.84(54.95-134.59) | 0.49(0.46 to 0.52) | 0.77(0.72 to 0.82) | 0.35(0.29 to 0.41) | 0.37(0.31 to 0.42) |
| Bulgaria | 1,411(882-2,131) | 81.26(50.79-122.71) | 884(554-1,329) | 90.54(56.76-136.15) | 0.35(0.3 to 0.41) | 0.55(0.41 to 0.7) | 0.03(-0.04 to 0.1) | 0.5(0.43 to 0.56) |
| Croatia | 827(521-1,249) | 83.75(52.8-126.55) | 551(346-839) | 92.32(57.95-140.52) | 0.32(0.28 to 0.36) | 0.14(0.06 to 0.23) | 0.26(0.21 to 0.32) | 0.49(0.43 to 0.55) |
| Czechia | 1,841(1,158-2,762) | 83.55(52.56-125.33) | 1,587(989-2,384) | 92.49(57.62-138.89) | 0.34(0.27 to 0.41) | 0.91(0.74 to 1.08) | -0.22(-0.34 to -0.1) | 0.35(0.28 to 0.42) |
| Hungary | 1,748(1,087-2,612) | 82.01(51-122.56) | 1,268(803-1,908) | 91.31(57.81-137.44) | 0.35(0.29 to 0.4) | 0.64(0.53 to 0.75) | 0.18(0.1 to 0.27) | 0.22(0.19 to 0.25) |
| North Macedonia | 423(267-642) | 80.29(50.73-121.86) | 296(187-454) | 90.34(57.22-138.62) | 0.38(0.34 to 0.43) | 0.39(0.32 to 0.45) | 0.36(0.26 to 0.45) | 0.43(0.36 to 0.5) |
| Montenegro | 135(84-201) | 83.29(52.29-124.1) | 102(64-155) | 91.14(57.17-139.13) | 0.29(0.26 to 0.32) | 0.06(0 to 0.13) | 0.43(0.39 to 0.48) | 0.35(0.34 to 0.36) |
| Poland | 7,411(4,690-11,292) | 77.39(48.97-117.91) | 5,173(3,268-7,780) | 87.88(55.52-132.19) | 0.41(0.39 to 0.43) | 0.64(0.6 to 0.69) | 0.02(-0.01 to 0.05) | 0.59(0.56 to 0.62) |
| Romania | 4,787(3,004-7,128) | 85.97(53.94-128.01) | 2,796(1,772-4,300) | 92.9(58.86-142.84) | 0.26(0.24 to 0.29) | 0.49(0.45 to 0.53) | 0.21(0.19 to 0.24) | 0.13(0.07 to 0.19) |
| Serbia | 1,763(1,103-2,646) | 81.27(50.87-122.02) | 1,219(775-1,855) | 91.77(58.39-139.72) | 0.39(0.34 to 0.44) | 0.34(0.25 to 0.43) | 0.42(0.35 to 0.49) | 0.33(0.23 to 0.42) |
| Slovakia | 1,095(690-1,655) | 82.58(52.04-124.85) | 782(499-1,179) | 91.24(58.2-137.69) | 0.32(0.29 to 0.36) | 0.72(0.66 to 0.78) | -0.03(-0.05 to 0) | 0.32(0.24 to 0.4) |
| Slovenia | 357(225-533) | 86.39(54.52-128.82) | 297(187-451) | 95.1(59.83-144.41) | 0.31(0.29 to 0.34) | 0.39(0.35 to 0.42) | 0.01(-0.02 to 0.04) | 0.55(0.51 to 0.6) |
| Belarus | 1,839(1,153-2,795) | 76.51(47.95-116.26) | 1,390(861-2,111) | 88.1(54.59-133.79) | 0.46(0.38 to 0.55) | 0.53(0.31 to 0.74) | 0.05(-0.02 to 0.11) | 0.86(0.74 to 0.97) |
| Estonia | 269(170-404) | 76.97(48.71-115.79) | 191(121-291) | 88.51(56.03-134.55) | 0.46(0.4 to 0.52) | 0.87(0.71 to 1.02) | -0.02(-0.1 to 0.06) | 0.54(0.48 to 0.6) |
| Latvia | 441(279-678) | 77.58(49.06-119.14) | 265(168-404) | 89.33(56.42-135.86) | 0.45(0.4 to 0.5) | 0.86(0.72 to 1.01) | 0.11(0.06 to 0.16) | 0.43(0.36 to 0.5) |
| Lithuania | 637(396-989) | 76.7(47.62-119) | 371(231-561) | 90.98(56.72-137.47) | 0.55(0.52 to 0.59) | 0.82(0.77 to 0.87) | 0.06(-0.03 to 0.16) | 0.74(0.7 to 0.78) |
| Republic of Moldova | 910(582-1,394) | 73.59(47.09-112.77) | 437(271-666) | 83.72(51.84-127.6) | 0.42(0.37 to 0.46) | 0.59(0.49 to 0.68) | -0.02(-0.05 to 0.01) | 0.67(0.59 to 0.75) |
| Russian Federation | 26,921(16,862-40,781) | 77.58(48.6-117.53) | 23,018(14,576-34,766) | 88.27(55.9-133.32) | 0.42(0.37 to 0.46) | 0.38(0.27 to 0.5) | 0.07(0.05 to 0.1) | 0.82(0.75 to 0.89) |
| Ukraine | 8,717(5,450-13,413) | 76.64(47.91-117.92) | 5,456(3,424-8,282) | 85.98(53.96-130.52) | 0.38(0.32 to 0.44) | 0.43(0.31 to 0.56) | -0.2(-0.25 to -0.15) | 0.85(0.74 to 0.95) |
| Brunei Darussalam | 147(91-226) | 162.42(100.93-249.64) | 168(105-258) | 177.19(111.37-272.5) | 0.29(0.27 to 0.31) | 0.47(0.45 to 0.48) | 0.48(0.44 to 0.53) | -0.03(-0.06 to 0) |
| Japan | 41,048(25,952-63,215) | 177.78(112.4-273.79) | 28,159(17,726-43,293) | 182.33(114.77-280.32) | 0.08(0.07 to 0.09) | -0.11(-0.13 to -0.09) | 0.12(0.1 to 0.14) | 0.23(0.21 to 0.24) |
| Republic of Korea | 19,924(12,386-30,596) | 175.23(108.93-269.08) | 11,601(7,293-17,615) | 190.98(120.06-289.98) | 0.27(0.2 to 0.34) | -0.12(-0.22 to -0.03) | 0.57(0.38 to 0.76) | 0.38(0.34 to 0.42) |
| Singapore | 1,105(691-1,691) | 170.13(106.46-260.42) | 1,436(895-2,202) | 176.84(110.27-271.18) | 0.13(0.08 to 0.19) | 0.08(0.01 to 0.16) | 0.35(0.22 to 0.49) | -0.02(-0.08 to 0.05) |
| Australia | 6,458(4,081-9,935) | 170.58(107.79-262.42) | 8,551(5,363-13,087) | 180.05(112.91-275.54) | 0.17(0.14 to 0.21) | 0.26(0.22 to 0.3) | -0.08(-0.19 to 0.03) | 0.31(0.27 to 0.34) |
| New Zealand | 1,338(848-2,069) | 167.19(105.95-258.56) | 1,747(1,096-2,671) | 177.91(111.67-272.05) | 0.21(0.18 to 0.23) | 0.34(0.3 to 0.38) | -0.05(-0.08 to -0.02) | 0.32(0.27 to 0.37) |
| Andorra | 12(7-18) | 124.4(77.33-191.77) | 13(8-20) | 131.26(81.8-200.25) | 0.17(0.09 to 0.26) | 0.15(0.01 to 0.3) | 0.22(0.08 to 0.37) | 0.16(0.06 to 0.26) |
| Austria | 1,632(1,014-2,507) | 121.07(75.23-185.92) | 1,647(1,039-2,488) | 127(80.12-191.79) | 0.16(0.12 to 0.2) | 0.34(0.28 to 0.39) | -0.04(-0.08 to 0.01) | 0.14(0.06 to 0.23) |
| Belgium | 2,167(1,377-3,352) | 119.96(76.24-185.62) | 2,455(1,557-3,826) | 128.4(81.45-200.08) | 0.22(0.2 to 0.25) | 0.31(0.24 to 0.37) | 0.03(0.01 to 0.04) | 0.35(0.31 to 0.38) |
| Cyprus | 233(148-364) | 117.92(74.56-183.9) | 276(172-425) | 126.19(78.80-194.39) | 0.22(0.2 to 0.24) | 0.48(0.44 to 0.53) | -0.09(-0.11 to -0.06) | 0.21(0.18 to 0.24) |
| Denmark | 1,303(820-1,999) | 147.48(92.78-226.28) | 1,481(930-2,241) | 155.2(97.44-234.88) | 0.17(0.14 to 0.2) | 0.42(0.37 to 0.46) | 0.03(-0.02 to 0.08) | 0.05(0.02 to 0.08) |
| Finland | 1,508(952-2,319) | 156.25(98.62-240.34) | 1,375(871-2,092) | 162.36(102.81-246.95) | 0.13(0.1 to 0.16) | 0.3(0.23 to 0.38) | -0.08(-0.11 to -0.04) | 0.17(0.13 to 0.22) |
| France | 14,124(8,932-21,469) | 120.57(76.25-183.28) | 14,788(9,359-22,545) | 127.4(80.62-194.23) | 0.18(0.14 to 0.22) | 0.14(0.05 to 0.23) | 0.19(0.18 to 0.21) | 0.23(0.18 to 0.27) |
| Germany | 25,721(16,372-39,437) | 198.68(126.46-304.63) | 24,617(15,724-37,597) | 205.75(131.42-314.24) | 0.11(0.09 to 0.13) | 0.11(0.07 to 0.14) | 0.14(0.1 to 0.19) | 0.15(0.12 to 0.19) |
| Greece | 2,440(1,541-3,796) | 120.56(76.17-187.61) | 1,739(1,088-2,641) | 124.65(78.03-189.38) | 0.1(0.06 to 0.14) | 0.06(-0.01 to 0.13) | 0.03(0 to 0.06) | 0.15(0.07 to 0.23) |
| Iceland | 77(48-116) | 120.72(76.12-183.43) | 86(54-133) | 127.14(79.68-196.77) | 0.17(0.15 to 0.2) | 0.37(0.31 to 0.43) | -0.07(-0.1 to -0.03) | 0.25(0.21 to 0.28) |
| Ireland | 1,194(740-1,844) | 121.51(75.29-187.65) | 1,287(812-1,984) | 129.09(81.43-199) | 0.2(0.16 to 0.24) | 0.04(0.02 to 0.06) | 0.16(0.04 to 0.28) | 0.4(0.35 to 0.46) |
| Israel | 908(576-1,380) | 59.25(37.55-89.99) | 1,709(1,075-2,616) | 65.05(40.9-99.55) | 0.3(0.26 to 0.35) | 0.33(0.32 to 0.35) | 0.25(0.17 to 0.33) | 0.35(0.27 to 0.42) |
| Italy | 10,816(6,898-16,779) | 117.19(74.74-181.8) | 9,484(6,048-14,567) | 124.79(79.59-191.68) | 0.21(0.19 to 0.22) | 0.26(0.25 to 0.28) | 0.13(0.1 to 0.16) | 0.24(0.21 to 0.27) |
| Luxembourg | 80(50-124) | 121.02(76.08-186.98) | 130(81-200) | 128.51(80.49-197.92) | 0.2(0.17 to 0.23) | 0.26(0.21 to 0.32) | 0.15(0.12 to 0.18) | 0.17(0.11 to 0.24) |
| Malta | 103(65-157) | 117.87(74.33-178.89) | 80(51-123) | 124.66(79.05-191.67) | 0.18(0.15 to 0.21) | 0.37(0.33 to 0.4) | -0.08(-0.16 to 0) | 0.24(0.22 to 0.26) |
| Netherlands | 2,818(1,768-4,356) | 103.4(64.87-159.85) | 2,948(1,833-4,509) | 109.91(68.36-168.11) | 0.2(0.17 to 0.24) | 0.34(0.31 to 0.37) | 0.25(0.19 to 0.3) | 0.08(0 to 0.16) |
| Norway | 874(552-1,345) | 109.52(69.09-168.45) | 1,092(693-1,678) | 118.25(75.05-181.6) | 0.25(0.23 to 0.27) | 0.46(0.41 to 0.51) | 0.05(0.03 to 0.08) | 0.27(0.24 to 0.3) |
| Portugal | 2,472(1,558-3,725) | 116.84(73.61-176.04) | 1,669(1,071-2,545) | 122.50(78.6-186.82) | 0.14(0.1 to 0.19) | 0.08(0.06 to 0.11) | 0.31(0.25 to 0.36) | 0.07(-0.03 to 0.16) |
| Spain | 12,113(7,644-18,727) | 154.58(97.56-238.98) | 10,466(6,590-16,110) | 161.49(101.68-248.58) | 0.15(0.12 to 0.18) | 0.06(0.01 to 0.12) | 0.05(-0.02 to 0.13) | 0.29(0.27 to 0.32) |
| Sweden | 2,227(1,407-3,411) | 144.19(91.09-220.86) | 2,811(1,790-4,312) | 154.39(98.29-236.84) | 0.23(0.19 to 0.26) | 0.7(0.59 to 0.82) | -0.16(-0.18 to -0.13) | 0.21(0.19 to 0.23) |
| Switzerland | 1,438(907-2,206) | 124.43(78.47-190.9) | 1,745(1,112-2,681) | 130.94(83.43-201.22) | 0.17(0.15 to 0.19) | 0.32(0.28 to 0.37) | -0.02(-0.05 to 0) | 0.23(0.19 to 0.26) |
| United Kingdom | 10,759(6,669-16,412) | 98.52(61.07-150.28) | 12,555(7,846-19,136) | 106.55(66.59-162.41) | 0.26(0.24 to 0.27) | 0.4(0.37 to 0.43) | -0.1(-0.12 to -0.07) | 0.4(0.37 to 0.44) |
| Argentina | 16,859(10,627-25,720) | 166.34(104.85-253.77) | 18,210(11,537-28,010) | 178.82(113.29-275.05) | 0.24(0.21 to 0.26) | 0.2(0.18 to 0.22) | 0.09(0.08 to 0.11) | 0.43(0.36 to 0.49) |
| Chile | 6,448(4,023-9,964) | 162.35(101.3-250.87) | 6,556(4,075-9,986) | 179.51(111.6-273.44) | 0.33(0.29 to 0.36) | 0.61(0.56 to 0.67) | 0.02(-0.05 to 0.1) | 0.37(0.31 to 0.43) |
| Uruguay | 1,364(866-2,094) | 166.68(105.75-255.86) | 1,173(738-1,812) | 177.82(111.92-274.81) | 0.21(0.19 to 0.23) | 0.12(0.09 to 0.14) | 0.31(0.28 to 0.33) | 0.19(0.16 to 0.22) |
| Canada | 10,460(6,648-15,818) | 181.86(115.59-275.02) | 11,888(7,490-17,925) | 192.62(121.36-290.44) | 0.19(0.16 to 0.22) | 0.43(0.38 to 0.47) | -0.16(-0.22 to -0.1) | 0.32(0.27 to 0.36) |
| United States of America | 102,486(65,315-154,299) | 183.3(116.82-275.98) | 114,189(73,219-171,808) | 192.13(123.19-289.07) | 0.15(0.13 to 0.17) | 0.28(0.24 to 0.31) | -0.07(-0.1 to -0.04) | 0.22(0.2 to 0.24) |
| Antigua and Barbuda | 11(7-17) | 60.59(36.95-93.07) | 12(7-18) | 69.05(43.96-106.29) | 0.42(0.39 to 0.45) | 0.45(0.41 to 0.49) | 0.47(0.41 to 0.54) | 0.34(0.3 to 0.38) |
| Bahamas | 52(33-79) | 64.98(40.34-97.99) | 58(36-88) | 71.56(44.7-108.48) | 0.31(0.27 to 0.35) | 0.59(0.51 to 0.68) | 0.24(0.18 to 0.31) | 0.16(0.09 to 0.22) |
| Barbados | 39(25-60) | 62.87(39.75-96.82) | 32(20-50) | 68.73(43.46-105.68) | 0.29(0.26 to 0.33) | 0.32(0.26 to 0.38) | 0.48(0.42 to 0.54) | 0.16(0.11 to 0.22) |
| Belize | 45(28-69) | 55.28(34.48-84.35) | 78(50-120) | 63.65(40.36-97.49) | 0.45(0.42 to 0.48) | 0.55(0.5 to 0.6) | 0.52(0.47 to 0.57) | 0.31(0.28 to 0.34) |
| Cuba | 1,494(926-2,285) | 59.67(36.98-91.24) | 1,185(743-1,824) | 66.68(41.83-102.66) | 0.36(0.31 to 0.42) | 0.23(0.15 to 0.31) | 0.37(0.28 to 0.46) | 0.59(0.5 to 0.67) |
| Dominica | 15(9-22) | 59.05(36.49-89.93) | 9(6-14) | 69.34(43.45-105.54) | 0.52(0.5 to 0.55) | 0.79(0.74 to 0.85) | 0.5(0.44 to 0.55) | 0.35(0.32 to 0.39) |
| Dominican Republic | 1,472(915-2,264) | 54.62(33.94-83.99) | 1,850(1,163-2,838) | 62.96(39.57-96.57) | 0.46(0.41 to 0.51) | 0.38(0.27 to 0.48) | 0.56(0.54 to 0.58) | 0.44(0.33 to 0.55) |
| Grenada | 19(12-28) | 56.2(35.21-85.02) | 14(9-22) | 65.83(41.75-100) | 0.51(0.48 to 0.54) | 0.6(0.54 to 0.66) | 0.52(0.47 to 0.56) | 0.44(0.39 to 0.5) |
| Guyana | 160(100-245) | 54.5(34.11-83.28) | 137(85-210) | 64.21(39.8-98.44) | 0.53(0.49 to 0.57) | 0.76(0.7 to 0.82) | 0.28(0.16 to 0.39) | 0.55(0.52 to 0.57) |
| Haiti | 1,375(863-2,098) | 50.68(31.81-77.34) | 2,457(1,514-3,757) | 56.44(34.79-86.32) | 0.35(0.33 to 0.37) | 0.35(0.31 to 0.39) | 0.29(0.27 to 0.31) | 0.41(0.37 to 0.44) |
| Jamaica | 491(306-748) | 58.84(36.58-89.6) | 388(242-591) | 66.45(41.42-101.19) | 0.39(0.36 to 0.42) | 0.53(0.48 to 0.58) | 0.28(0.25 to 0.31) | 0.36(0.31 to 0.41) |
| Saint Lucia | 29(18-45) | 57.12(35.46-87.8) | 20(12-30) | 66.08(42.06-99.99) | 0.47(0.46 to 0.49) | 0.71(0.66 to 0.75) | 0.37(0.35 to 0.39) | 0.38(0.36 to 0.4) |
| Saint Vincent and the Grenadines | 23(15-36) | 56.76(35.6-87.43) | 16(10-25) | 65.14(41.34-98.63) | 0.45(0.42 to 0.48) | 0.46(0.37 to 0.55) | 0.39(0.36 to 0.42) | 0.5(0.47 to 0.52) |
| Suriname | 75(47-115) | 57.85(35.81-88.24) | 92(58-142) | 64.53(40.44-98.92) | 0.35(0.31 to 0.39) | 0.24(0.2 to 0.27) | 0.42(0.3 to 0.53) | 0.35(0.29 to 0.41) |
| Trinidad and Tobago | 256(162-396) | 62.98(39.95-97.39) | 194(122-299) | 71.12(44.82-109.58) | 0.4(0.37 to 0.42) | 0.17(0.13 to 0.21) | 0.5(0.47 to 0.52) | 0.48(0.43 to 0.52) |
| Bolivia (Plurinational State of) | 1,631(1,026-2,468) | 60.71(38.18-91.89) | 2,411(1,520-3,662) | 69.14(43.6-105.03) | 0.43(0.41 to 0.45) | 0.5(0.48 to 0.52) | 0.31(0.27 to 0.34) | 0.5(0.47 to 0.52) |
| Ecuador | 2,492(1,568-3,776) | 64.48(40.57-97.7) | 3,675(2,311-5,545) | 72.47(45.58-109.34) | 0.38(0.34 to 0.42) | 0.26(0.2 to 0.33) | 0.37(0.28 to 0.46) | 0.5(0.44 to 0.55) |
| Peru | 5,408(3,427-8,151) | 65.15(41.28-98.2) | 7,020(4,417-10,645) | 73.61(46.31-111.61) | 0.4(0.36 to 0.44) | 0.35(0.34 to 0.37) | 0.39(0.37 to 0.4) | 0.45(0.35 to 0.55) |
| Colombia | 5,674(3,592-8,565) | 48.65(30.8-73.44) | 6,019(3,774-9,162) | 56.71(35.56-86.33) | 0.49(0.44 to 0.55) | 0.48(0.41 to 0.54) | 0.38(0.26 to 0.51) | 0.6(0.5 to 0.71) |
| Costa Rica | 575(356-891) | 51.18(31.71-79.24) | 604(381-921) | 59.35(37.43-90.56) | 0.48(0.45 to 0.52) | 0.45(0.37 to 0.52) | 0.31(0.26 to 0.36) | 0.7(0.67 to 0.72) |
| El Salvador | 988(608-1,517) | 45.8(28.18-70.29) | 996(633-1,511) | 54.77(34.8-83.05) | 0.59(0.52 to 0.65) | 0.75(0.67 to 0.84) | 0.37(0.21 to 0.53) | 0.6(0.52 to 0.69) |
| Guatemala | 1,786(1,126-2,710) | 43.99(27.74-66.73) | 2,615(1,610-4,013) | 53(32.64-81.33) | 0.6(0.55 to 0.65) | 0.63(0.56 to 0.7) | 0.71(0.69 to 0.73) | 0.51(0.39 to 0.62) |
| Honduras | 990(628-1,501) | 44.81(28.41-67.96) | 1,697(1,062-2,587) | 51.77(32.42-78.93) | 0.47(0.45 to 0.49) | 0.42(0.37 to 0.47) | 0.5(0.49 to 0.52) | 0.48(0.46 to 0.49) |
| Mexico | 15,931(10,082-24,088) | 47.67(30.17-72.09) | 18,350(11,546-27,906) | 57.22(36.01-87.02) | 0.59(0.56 to 0.62) | 0.48(0.47 to 0.49) | 0.43(0.4 to 0.45) | 0.84(0.77 to 0.92) |
| Nicaragua | 824(523-1,250) | 45.26(28.71-68.63) | 1,042(651-1,601) | 52.59(32.85-80.82) | 0.49(0.46 to 0.53) | 0.6(0.58 to 0.62) | 0.33(0.3 to 0.37) | 0.53(0.44 to 0.61) |
| Panama | 427(266-645) | 51.21(31.94-77.41) | 688(432-1,053) | 59.69(37.48-91.27) | 0.5(0.45 to 0.54) | 0.37(0.34 to 0.4) | 0.35(0.3 to 0.4) | 0.74(0.64 to 0.84) |
| Venezuela (Bolivarian Republic of) | 3,530(2,209-5,287) | 49.76(31.14-74.52) | 3,687(2,334-5,657) | 55.65(35.24-85.4) | 0.36(0.3 to 0.43) | 0.64(0.59 to 0.68) | 0.1(-0.06 to 0.26) | 0.4(0.29 to 0.52) |
| Brazil | 29,673(18,873-45,076) | 57.12(36.33-86.78) | 30,720(19,475-46,816) | 63.75(40.42-97.16) | 0.35(0.34 to 0.37) | 0.18(0.15 to 0.22) | 0.34(0.32 to 0.36) | 0.51(0.5 to 0.52) |
| Paraguay | 944(595-1,436) | 56.52(35.64-85.98) | 1,312(825-2,031) | 65.34(41.11-101.16) | 0.47(0.43 to 0.51) | 0.5(0.44 to 0.57) | 0.36(0.31 to 0.41) | 0.53(0.46 to 0.6) |
| Algeria | 6,513(4,118-9,960) | 60.73(38.4-92.87) | 9,382(6,010-13,882) | 70.53(45.18-104.36) | 0.49(0.45 to 0.52) | 0.46(0.41 to 0.51) | 0.15(0.1 to 0.19) | 0.77(0.71 to 0.84) |
| Bahrain | 104(65-157) | 63.56(39.57-96.36) | 224(139-340) | 75.53(46.85-114.65) | 0.56(0.51 to 0.61) | 0.48(0.38 to 0.57) | 0.6(0.55 to 0.66) | 0.59(0.5 to 0.68) |
| Egypt | 12,774(8,043-19,160) | 57.59(36.26-86.38) | 25,721(16,315-39,150) | 69.79(44.27-106.23) | 0.62(0.58 to 0.66) | 0.51(0.44 to 0.58) | 0.41(0.39 to 0.43) | 0.94(0.86 to 1.02) |
| Iran (Islamic Republic of) | 15,247(9,631-22,982) | 60.06(37.94-90.53) | 14,549(9,138-22,102) | 72.1(45.28-109.53) | 0.59(0.56 to 0.62) | 0.54(0.47 to 0.61) | 0.46(0.45 to 0.47) | 0.78(0.73 to 0.82) |
| Iraq | 4,749(3,027-7,244) | 57.65(36.75-87.96) | 9,534(5,986-14,607) | 70.82(44.46-108.51) | 0.66(0.63 to 0.7) | 0.3(0.26 to 0.35) | 0.73(0.7 to 0.76) | 0.9(0.81 to 0.99) |
| Jordan | 1,010(635-1,541) | 61.86(38.87-94.36) | 2,641(1,654-3,978) | 72.7(45.54-109.5) | 0.53(0.5 to 0.56) | 0.34(0.32 to 0.35) | 0.65(0.58 to 0.72) | 0.56(0.5 to 0.61) |
| Kuwait | 379(240-576) | 68.36(43.29-103.91) | 674(417-1,017) | 79.71(49.35-120.24) | 0.5(0.45 to 0.54) | 0.01(-0.09 to 0.11) | 0.54(0.47 to 0.61) | 0.77(0.74 to 0.8) |
| Lebanon | 625(392-942) | 59.75(37.52-90.09) | 923(590-1,416) | 72.18(46.18-110.83) | 0.62(0.57 to 0.66) | 0.4(0.37 to 0.44) | 0.58(0.55 to 0.6) | 0.81(0.7 to 0.92) |
| Libya | 1,091(686-1,619) | 60.27(37.86-89.43) | 1,075(670-1,611) | 72.09(44.93-108.02) | 0.59(0.54 to 0.63) | 0.88(0.78 to 0.98) | 0.82(0.8 to 0.84) | 0.11(0.03 to 0.19) |
| Morocco | 5,576(3,466-8,396) | 56.99(35.41-85.79) | 6,493(4,109-10,064) | 66.32(41.97-102.77) | 0.49(0.47 to 0.52) | 0.27(0.26 to 0.29) | 0.44(0.42 to 0.45) | 0.73(0.66 to 0.79) |
| Palestine | 534(338-800) | 55.19(34.9-82.63) | 1,252(800-1,922) | 67.05(42.82-102.94) | 0.63(0.62 to 0.65) | 0.49(0.48 to 0.5) | 0.57(0.56 to 0.58) | 0.79(0.75 to 0.84) |
| Oman | 499(311-762) | 59.4(36.98-90.7) | 936(576-1,434) | 76.51(47.08-117.25) | 0.82(0.79 to 0.86) | 1(0.9 to 1.1) | 0.64(0.61 to 0.67) | 0.85(0.79 to 0.92) |
| Qatar | 81(50-122) | 64.48(40.15-97.9) | 389(245-599) | 78.77(49.57-121.21) | 0.65(0.61 to 0.69) | 0.76(0.73 to 0.79) | 0.35(0.32 to 0.38) | 0.82(0.71 to 0.93) |
| Saudi Arabia | 4,048(2,514-6,090) | 61.78(38.36-92.93) | 5,887(3,694-9,015) | 77.82(48.82-119.17) | 0.74(0.71 to 0.78) | 0.71(0.69 to 0.73) | 0.76(0.67 to 0.86) | 0.75(0.7 to 0.8) |
| Syrian Arab Republic | 3,410(2,141-5,135) | 57.58(36.16-86.7) | 2,498(1,572-3,798) | 68.2(42.9-103.69) | 0.55(0.53 to 0.57) | 0.52(0.5 to 0.54) | 0.87(0.85 to 0.9) | 0.28(0.23 to 0.32) |
| Tunisia | 1,865(1,150-2,851) | 60.07(37.05-91.81) | 1,977(1,257-2,991) | 71.47(45.44-108.14) | 0.57(0.53 to 0.6) | 0.63(0.6 to 0.67) | 0.43(0.39 to 0.48) | 0.64(0.56 to 0.73) |
| Turkey | 12,260(7,713-18,562) | 59.84(37.64-90.6) | 13,544(8,575-20,732) | 73.13(46.3-111.94) | 0.65(0.63 to 0.67) | 0.4(0.38 to 0.43) | 0.71(0.7 to 0.72) | 0.79(0.73 to 0.85) |
| United Arab Emirates | 391(244-599) | 66.42(41.33-101.69) | 1,092(692-1,639) | 81.58(51.68-122.4) | 0.67(0.6 to 0.75) | 0.88(0.83 to 0.92) | 0.47(0.26 to 0.69) | 0.71(0.59 to 0.83) |
| Yemen | 3,697(2,313-5,612) | 52.11(32.6-79.11) | 8,487(5,409-12,901) | 61.55(39.23-93.56) | 0.54(0.5 to 0.58) | 0.36(0.3 to 0.42) | 0.56(0.49 to 0.64) | 0.65(0.57 to 0.73) |
| Afghanistan | 2,212(1,396-3,349) | 51.34(32.39-77.74) | 8,204(5,161-12,482) | 57.77(36.34-87.9) | 0.37(0.29 to 0.46) | 0.05(-0.03 to 0.13) | 0.59(0.36 to 0.83) | 0.44(0.36 to 0.52) |
| Bangladesh | 33,733(21,325-50,574) | 68.97(43.6-103.4) | 37,274(23,529-56,538) | 81.44(51.41-123.54) | 0.54(0.51 to 0.57) | 0.52(0.44 to 0.6) | 0.39(0.34 to 0.44) | 0.68(0.64 to 0.73) |
| Bhutan | 176(110-269) | 67.1(41.77-102.76) | 147(93-220) | 78.66(49.94-117.66) | 0.53(0.51 to 0.54) | 0.4(0.36 to 0.43) | 0.58(0.56 to 0.59) | 0.6(0.58 to 0.61) |
| India | 226,623(144,755-338,814) | 69.41(44.33-103.76) | 299,633(191,082-453,623) | 81.78(52.15-123.81) | 0.53(0.52 to 0.54) | 0.29(0.28 to 0.3) | 0.5(0.48 to 0.52) | 0.75(0.73 to 0.77) |
| Nepal | 6,272(3,930-9,483) | 74.45(46.65-112.55) | 8,062(5,013-12,157) | 87.37(54.33-131.75) | 0.52(0.5 to 0.54) | 0.43(0.4 to 0.46) | 0.64(0.59 to 0.68) | 0.51(0.49 to 0.53) |
| Pakistan | 33,555(21,403-51,040) | 68.14(43.46-103.65) | 67,337(42,666-102,353) | 78.81(49.94-119.79) | 0.47(0.46 to 0.48) | 0.35(0.33 to 0.37) | 0.35(0.34 to 0.37) | 0.65(0.63 to 0.67) |
| Angola | 2,317(1,473-3,541) | 49.15(31.24-75.1) | 8,662(5,429-13,187) | 56.82(35.61-86.5) | 0.47(0.44 to 0.49) | 0.19(0.17 to 0.22) | 0.41(0.36 to 0.45) | 0.74(0.69 to 0.79) |
| Central African Republic | 590(370-885) | 48.28(30.24-72.39) | 1,168(749-1,757) | 51.14(32.81-76.92) | 0.19(0.14 to 0.24) | 0.1(0.09 to 0.12) | 0.2(0.08 to 0.31) | 0.26(0.2 to 0.33) |
| Congo | 564(346-854) | 53.59(32.84-81.13) | 1,159(715-1,781) | 60.07(37.07-92.33) | 0.37(0.34 to 0.41) | 0.11(0.05 to 0.18) | 0.17(0.1 to 0.23) | 0.74(0.69 to 0.79) |
| Democratic Republic of the Congo | 8,833(5,605-13,158) | 49.89(31.66-74.32) | 20,385(12,835-30,157) | 53.65(33.78-79.36) | 0.23(0.19 to 0.28) | -0.13(-0.2 to -0.06) | 0.1(0.01 to 0.18) | 0.68(0.6 to 0.75) |
| Equatorial Guinea | 95(60-140) | 48.27(30.61-71.28) | 374(236-567) | 63.9(40.37-96.98) | 0.91(0.87 to 0.95) | 0.78(0.68 to 0.88) | 1.21(1.18 to 1.23) | 0.74(0.66 to 0.81) |
| Gabon | 223(141-337) | 54.71(34.63-82.8) | 404(249-608) | 63.18(38.89-95.06) | 0.46(0.43 to 0.49) | 0.43(0.41 to 0.45) | 0.35(0.32 to 0.37) | 0.59(0.51 to 0.67) |
| Burundi | 3,044(1,929-4,629) | 116.13(73.6-176.61) | 7,205(4,538-11,148) | 123.07(77.52-190.43) | 0.19(0.16 to 0.21) | 0.42(0.37 to 0.48) | -0.23(-0.28 to -0.18) | 0.41(0.37 to 0.45) |
| Comoros | 257(160-392) | 120.94(75.08-184.28) | 316(200-486) | 131.61(83.20-202.54) | 0.28(0.26 to 0.3) | 0.25(0.21 to 0.29) | 0.28(0.26 to 0.3) | 0.31(0.29 to 0.33) |
| Djibouti | 213(133-326) | 122.24(76.38-187.02) | 533(334-818) | 129.08(80.84-197.92) | 0.18(0.15 to 0.21) | 0.11(0.01 to 0.21) | -0.02(-0.04 to 0) | 0.45(0.42 to 0.47) |
| Eritrea | 1,886(1,175-2,877) | 118.5(73.8-180.75) | 3,202(2,013-4,856) | 126.84(79.72-192.35) | 0.22(0.18 to 0.26) | 0.31(0.2 to 0.43) | 0.2(0.16 to 0.24) | 0.15(0.12 to 0.18) |
| Ethiopia | 32,706(20,904-49,828) | 134.24(85.8-204.52) | 61,666(39,150-94,515) | 139.04(88.27-213.11) | 0.12(0.1 to 0.14) | 0.05(0 to 0.1) | 0.27(0.24 to 0.31) | 0.01(0 to 0.02) |
| Kenya | 13,587(8,639-20,918) | 121.64(77.34-187.27) | 24,905(15,827-38,321) | 133.43(84.79-205.3) | 0.3(0.28 to 0.31) | 0.15(0.12 to 0.19) | 0.2(0.18 to 0.22) | 0.51(0.49 to 0.53) |
| Madagascar | 6,494(4,113-9,895) | 119.02(75.38-181.37) | 14,968(9,321-22,542) | 127.56(79.44-192.12) | 0.22(0.2 to 0.25) | -0.05(-0.08 to -0.02) | 0.25(0.23 to 0.28) | 0.44(0.38 to 0.49) |
| Malawi | 5,220(3,250-7,976) | 114.74(71.43-175.31) | 10,454(6,501-16,093) | 128.69(80.03-198.09) | 0.38(0.33 to 0.43) | -0.03(-0.18 to 0.12) | 0.46(0.42 to 0.5) | 0.62(0.59 to 0.66) |
| Mauritius | 238(151-363) | 72.03(45.82-110.09) | 168(106-257) | 81.06(51.06-124.03) | 0.38(0.34 to 0.42) | 0.45(0.38 to 0.53) | 0.4(0.3 to 0.5) | 0.32(0.29 to 0.36) |
| Mozambique | 7,230(4,565-10,917) | 116.53(73.58-175.97) | 17,746(11,163-26,917) | 124.39(78.25-188.68) | 0.21(0.18 to 0.24) | -0.27(-0.36 to -0.18) | 0.39(0.37 to 0.41) | 0.46(0.44 to 0.47) |
| Rwanda | 4,029(2,532-6,131) | 118.74(74.64-180.69) | 6,414(4,025-9,771) | 129.04(80.97-196.59) | 0.27(0.24 to 0.31) | 0.12(0.06 to 0.19) | 0.29(0.23 to 0.36) | 0.38(0.35 to 0.41) |
| Seychelles | 17(11-26) | 73.48(46.27-111.67) | 19(12-29) | 81.61(51.29-124.52) | 0.34(0.32 to 0.37) | 0.48(0.46 to 0.5) | 0.03(-0.03 to 0.1) | 0.49(0.47 to 0.51) |
| Somalia | 4,473(2,839-6,791) | 114.82(72.88-174.33) | 11,869(7,490-18,054) | 114.9(72.51-174.79) | 0(-0.02 to 0.03) | -0.16(-0.21 to -0.12) | 0.04(0.02 to 0.07) | 0.1(0.06 to 0.13) |
| United Republic of Tanzania | 14,210(9,124-21,701) | 117.68(75.56-179.71) | 31,003(19,476-47,605) | 127.05(79.81-195.08) | 0.25(0.24 to 0.26) | 0.01(-0.01 to 0.03) | 0.25(0.23 to 0.27) | 0.45(0.43 to 0.46) |
| Uganda | 9,525(5,984-14,356) | 113.13(71.08-170.51) | 25,009(15,797-38,516) | 126.08(79.64-194.18) | 0.35(0.34 to 0.37) | 0.23(0.19 to 0.26) | 0.36(0.35 to 0.37) | 0.45(0.44 to 0.46) |
| Zambia | 4,450(2,791-6,645) | 118.51(74.33-176.99) | 10,740(6,760-16,568) | 129.84(81.73-200.3) | 0.3(0.27 to 0.32) | -0.06(-0.11 to 0) | 0.3(0.28 to 0.32) | 0.61(0.56 to 0.65) |
| Botswana | 408(259-617) | 69.07(43.8-104.43) | 534(340-807) | 76.52(48.73-115.51) | 0.33(0.3 to 0.36) | 0.45(0.4 to 0.5) | 0.25(0.24 to 0.27) | 0.3(0.24 to 0.36) |
| Lesotho | 455(283-688) | 66.65(41.4-100.85) | 459(288-719) | 72.73(45.73-113.98) | 0.28(0.25 to 0.31) | 0.26(0.22 to 0.31) | 0.31(0.27 to 0.34) | 0.29(0.22 to 0.35) |
| Namibia | 416(258-630) | 69.21(42.87-104.88) | 624(389-953) | 75.6(47.18-115.47) | 0.28(0.25 to 0.32) | 0.35(0.29 to 0.41) | 0.06(0 to 0.12) | 0.44(0.4 to 0.49) |
| South Africa | 9,815(6,169-14,854) | 72.1(45.31-109.11) | 11,836(7,423-17,854) | 77.84(48.82-117.42) | 0.25(0.23 to 0.26) | 0.3(0.29 to 0.31) | 0(-0.03 to 0.04) | 0.4(0.36 to 0.44) |
| Eswatini | 262(167-399) | 67.8(43.36-103.52) | 308(191-472) | 74.62(46.39-114.32) | 0.31(0.28 to 0.34) | 0.38(0.32 to 0.44) | 0.16(0.1 to 0.22) | 0.39(0.36 to 0.42) |
| Zimbabwe | 3,288(2,066-5,013) | 68.27(42.89-104.09) | 4,442(2,819-6,717) | 70.57(44.79-106.73) | 0.11(0.09 to 0.13) | 0.13(0.08 to 0.18) | -0.19(-0.22 to -0.16) | 0.39(0.35 to 0.42) |
| Benin | 1,418(891-2,140) | 58.54(36.81-88.35) | 3,821(2,383-5,814) | 62.84(39.19-95.63) | 0.23(0.2 to 0.25) | 0.17(0.14 to 0.21) | 0.07(0.05 to 0.09) | 0.4(0.35 to 0.45) |
| Burkina Faso | 2,674(1,715-4,079) | 56.66(36.34-86.44) | 6,217(3,913-9,367) | 59.94(37.73-90.31) | 0.18(0.17 to 0.19) | 0.07(0.05 to 0.09) | 0.2(0.19 to 0.21) | 0.26(0.25 to 0.28) |
| Cameroon | 2,961(1,874-4,547) | 60.64(38.39-93.13) | 9,060(5,730-13,742) | 67.28(42.55-102.04) | 0.34(0.32 to 0.36) | 0.25(0.22 to 0.29) | 0.14(0.12 to 0.17) | 0.59(0.56 to 0.62) |
| Cabo Verde | 98(62-149) | 61.99(39.38-94.47) | 101(62-155) | 70.23(43.53-108.32) | 0.41(0.39 to 0.42) | 0.46(0.44 to 0.48) | 0.26(0.23 to 0.3) | 0.49(0.47 to 0.52) |
| Chad | 1,627(1,023-2,456) | 55.59(34.97-83.92) | 5,301(3,344-8,088) | 58.81(37.09-89.72) | 0.19(0.18 to 0.2) | 0.09(0.07 to 0.11) | 0.21(0.2 to 0.21) | 0.26(0.25 to 0.27) |
| Coted'Ivoire | 3,397(2,146-5,201) | 59.56(37.63-91.19) | 7,470(4,718-11,481) | 64.56(40.77-99.21) | 0.27(0.23 to 0.3) | 0.22(0.14 to 0.29) | 0.08(0.04 to 0.13) | 0.49(0.42 to 0.55) |
| Gambia | 272(168-412) | 59(36.38-89.29) | 641(402-987) | 64.49(40.5-99.3) | 0.29(0.27 to 0.3) | 0.1(0.08 to 0.12) | 0.26(0.25 to 0.27) | 0.46(0.44 to 0.48) |
| Ghana | 4,212(2,610-6,329) | 62.72(38.85-94.23) | 9,025(5,668-13,855) | 70.05(43.99-107.54) | 0.37(0.35 to 0.38) | 0.28(0.26 to 0.3) | 0.21(0.18 to 0.23) | 0.59(0.57 to 0.61) |
| Guinea | 1,577(1,007-2,390) | 57.3(36.6-86.86) | 3,763(2,365-5,625) | 62.25(39.11-93.04) | 0.28(0.23 to 0.33) | 0.29(0.18 to 0.4) | 0.1(-0.01 to 0.21) | 0.43(0.4 to 0.46) |
| Guinea-Bissau | 284(180-433) | 58.86(37.28-89.67) | 568(361-876) | 63.26(40.24-97.48) | 0.23(0.2 to 0.26) | 0.18(0.14 to 0.21) | 0.06(0.02 to 0.1) | 0.44(0.4 to 0.47) |
| Liberia | 659(408-1,012) | 58.3(36.1-89.54) | 1,390(876-2,120) | 63.58(40.06-96.99) | 0.25(0.17 to 0.32) | -0.26(-0.39 to -0.13) | 0.35(0.16 to 0.53) | 0.59(0.51 to 0.68) |
| Mali | 2,310(1,456-3,497) | 55.93(35.24-84.68) | 6,931(4,380-10,356) | 59.88(37.83-89.46) | 0.22(0.2 to 0.24) | 0.07(0.05 to 0.09) | 0.27(0.25 to 0.29) | 0.29(0.25 to 0.33) |
| Mauritania | 565(350-859) | 61.17(37.89-92.98) | 1,263(785-1,909) | 68.17(42.35-103.01) | 0.35(0.33 to 0.37) | 0.28(0.25 to 0.31) | 0.19(0.16 to 0.22) | 0.55(0.51 to 0.58) |
| Niger | 2,241(1,406-3,417) | 55.16(34.61-84.09) | 7,303(4,656-11,116) | 57.21(36.48-87.09) | 0.12(0.11 to 0.14) | -0.19(-0.21 to -0.17) | 0.25(0.22 to 0.28) | 0.25(0.23 to 0.28) |
| Nigeria | 23,952(15,321-35,922) | 61.22(39.16-91.82) | 68,844(43,931-102,789) | 67.77(43.24-101.18) | 0.33(0.31 to 0.35) | 0(-0.01 to 0.01) | 0.35(0.3 to 0.4) | 0.57(0.56 to 0.59) |
| Sao Tome and Principe | 35(22-54) | 62.28(38.75-95.15) | 54(33-81) | 68.84(42.64-103.9) | 0.32(0.3 to 0.35) | -0.02(-0.04 to 0) | 0.33(0.28 to 0.39) | 0.6(0.56 to 0.64) |
| Senegal | 2,147(1,344-3,238) | 58.81(36.81-88.68) | 4,113(2,535-6,242) | 64.66(39.85-98.13) | 0.31(0.28 to 0.34) | 0.23(0.18 to 0.27) | 0.18(0.15 to 0.2) | 0.5(0.43 to 0.56) |
| Sierra Leone | 1,040(638-1,594) | 57.36(35.19-87.93) | 2,240(1,373-3,419) | 62.63(38.4-95.59) | 0.28(0.25 to 0.31) | 0.16(0.07 to 0.24) | 0.26(0.24 to 0.28) | 0.41(0.39 to 0.43) |
| Togo | 1,058(666-1,597) | 60.02(37.81-90.63) | 2,149(1,343-3,289) | 64.94(40.58-99.4) | 0.25(0.23 to 0.27) | 0.13(0.09 to 0.16) | 0.05(0.02 to 0.08) | 0.54(0.51 to 0.57) |
| American Samoa | 16(10-24) | 84.36(52.32-128.18) | 13(8-20) | 95.13(59.12-144.13) | 0.39(0.36 to 0.42) | 0.41(0.34 to 0.47) | 0.34(0.31 to 0.37) | 0.42(0.36 to 0.48) |
| Bermuda | 8(5-12) | 63.89(40.65-97.67) | 6(4-9) | 72.64(46.13-112.18) | 0.41(0.39 to 0.43) | 0.41(0.38 to 0.43) | 0.5(0.48 to 0.53) | 0.32(0.28 to 0.36) |
| Cook Islands | 6(4-9) | 87.13(54.3-133.17) | 4(2-6) | 96.09(60.06-145.42) | 0.32(0.28 to 0.35) | 0.22(0.15 to 0.3) | 0.34(0.26 to 0.42) | 0.33(0.29 to 0.37) |
| Greenland | 24(15-37) | 170.93(106.13-260.18) | 22(14-33) | 185.73(116.17-281.14) | 0.26(0.16 to 0.36) | 0.67(0.56 to 0.79) | 0.19(0.14 to 0.24) | -0.03(-0.29 to 0.23) |
| Guam | 36(23-56) | 86.99(54.09-134.12) | 35(22-54) | 95.93(59.92-146.83) | 0.32(0.25 to 0.38) | 0.43(0.26 to 0.59) | 0.21(0.07 to 0.35) | 0.25(0.22 to 0.28) |
| Monaco | 4(3-7) | 122.53(77.87-184.49) | 7(4-10) | 130.97(82.92-202.18) | 0.22(0.2 to 0.25) | 0.32(0.28 to 0.36) | 0.17(0.14 to 0.2) | 0.2(0.17 to 0.24) |
| Nauru | 3(2-5) | 81.17(50.26-123.87) | 4(2-5) | 88.58(55.33-136.36) | 0.29(0.26 to 0.31) | 0.01(-0.03 to 0.05) | 0.19(0.15 to 0.24) | 0.6(0.55 to 0.64) |
| Niue | 1(0-1) | 86.3(54.01-131.8) | 0(0-1) | 93.5(58.98-140.95) | 0.26(0.24 to 0.29) | 0.19(0.14 to 0.25) | 0.26(0.21 to 0.31) | 0.3(0.28 to 0.33) |
| Northern Mariana Islands | 11(7-16) | 86.8(53.87-131.55) | 11(7-17) | 97.46(61.03-147.07) | 0.39(0.31 to 0.46) | 0.13(-0.05 to 0.32) | 0.87(0.73 to 1.01) | 0.39(0.35 to 0.43) |
| Palau | 4(2-6) | 87.11(53.63-133.77) | 3(2-5) | 95.04(59.87-144.98) | 0.28(0.24 to 0.33) | 0.24(0.18 to 0.3) | 0.11(0.04 to 0.17) | 0.45(0.38 to 0.53) |
| Puerto Rico | 629(396-952) | 63.12(39.8-95.57) | 327(207-503) | 73.58(46.5-113.13) | 0.5(0.48 to 0.52) | 0.48(0.45 to 0.52) | 0.38(0.34 to 0.41) | 0.57(0.54 to 0.61) |
| Saint Kitts and Nevis | 9(5-13) | 60.64(38.36-92.77) | 7(4-10) | 69.7(43.58-104.8) | 0.45(0.42 to 0.49) | 0.51(0.48 to 0.54) | 0.53(0.44 to 0.62) | 0.35(0.3 to 0.39) |
| San Marino | 5(3-8) | 123.04(78.82-188.45) | 6(4-9) | 129(81.86-199.92) | 0.16(0.13 to 0.18) | 0.12(0.05 to 0.2) | 0.24(0.23 to 0.24) | 0.1(0.08 to 0.12) |
| Tokelau | 0(0-1) | 81.98(51.62-125.59) | 0(0-1) | 92.65(57.52-141.99) | 0.41(0.32 to 0.51) | 0.55(0.38 to 0.72) | 0.26(0.08 to 0.45) | 0.53(0.45 to 0.62) |
| Tuvalu | 3(2-4) | 76.56(47.8-115.74) | 3(2-5) | 87.07(54.88-133.13) | 0.42(0.4 to 0.44) | 0.68(0.62 to 0.73) | 0.38(0.36 to 0.39) | 0.25(0.23 to 0.26) |
| United States Virgin Islands | 20(13-31) | 63.52(39.6-96.78) | 10(6-15) | 72.81(44.92-111.12) | 0.44(0.42 to 0.47) | 0.51(0.44 to 0.58) | 0.53(0.51 to 0.56) | 0.31(0.27 to 0.35) |
| South Sudan | 3,112(1,961-4,764) | 118.57(74.73-181.54) | 5,278(3,304-7,973) | 122.89(76.93-185.63) | 0.12(0.09 to 0.14) | -0.09(-0.11 to -0.07) | 0.11(0.09 to 0.13) | 0.31(0.24 to 0.38) |
| Sudan | 4,700(2,960-7,159) | 52.86(33.29-80.51) | 10,759(6,614-16,261) | 64.86(39.87-98.02) | 0.66(0.63 to 0.69) | 0.38(0.31 to 0.45) | 0.7(0.65 to 0.74) | 0.86(0.81 to 0.9) |

**sTable9 Slope index of inequality and concentration index of infectious skin diseases from 1990 to 2021**

|  | Infectious skin diseases | | Bacterial skin diseases | | Fungal skin diseases | | Viral skin diseases | |
| --- | --- | --- | --- | --- | --- | --- | --- | --- |
| Year | Slope index of inequality | Concentration index | Slope index of inequality | Concentration index | Slope index of inequality | Concentration index | Slope index of inequality | Concentration index |
| 1990 | -111.81(-139.61,-84) | -0.12(-0.15,-0.09) | -59.91(-71.86,-47.96) | -0.32(-0.38,-0.26) | -82.32(-94.85,-69.78) | -0.35(-0.41,-0.28) | 30.42(17.35,43.5) | 0.09(0.06-0.12) |
| 1991 | -112.59(-140.32,-84.85) | -0.12(-0.15,-0.09) | -59.87(-71.65,-48.08) | -0.32(-0.38,-0.26) | -83.46(-95.98,-70.93) | -0.35(-0.42,-0.29) | 30.74(17.62,43.86) | 0.09(0.06-0.12) |
| 1992 | -112.64(-140.22,-85.06) | -0.13(-0.16,-0.09) | -59.62(-71.23,-48) | -0.33(-0.39,-0.27) | -83.67(-96.17,-71.16) | -0.35(-0.42,-0.29) | 30.64(17.52,43.76) | 0.09(0.06-0.12) |
| 1993 | -110.63(-138.22,-83.05) | -0.12(-0.15,-0.09) | -58.64(-70.14,-47.13) | -0.32(-0.38,-0.26) | -83.26(-95.86,-70.65) | -0.35(-0.41,-0.28) | 31.26(18.13,44.38) | 0.09(0.06-0.11) |
| 1994 | -109.25(-136.57,-81.94) | -0.12(-0.15,-0.09) | -57.13(-68.48,-45.79) | -0.32(-0.38,-0.25) | -83.34(-95.92,-70.77) | -0.35(-0.42,-0.29) | 31.22(18.1,44.34) | 0.08(0.06-0.11) |
| 1995 | -107.67(-134.85,-80.49) | -0.12(-0.15,-0.09) | -55.72(-67.05,-44.39) | -0.31(-0.38,-0.25) | -83.45(-95.95,-70.94) | -0.35(-0.42,-0.29) | 31.50(18.4,44.59) | 0.08(0.06-0.11) |
| 1996 | -106.38(-133.27,-79.49) | -0.12(-0.15,-0.09) | -54.82(-65.92,-43.73) | -0.31(-0.38,-0.25) | -83.44(-95.93,-70.94) | -0.36(-0.42,-0.3) | 31.88(18.77,44.99) | 0.09(0.06-0.12) |
| 1997 | -105.68(-132.26,-79.09) | -0.12(-0.15,-0.09) | -53.64(-64.62,-42.66) | -0.31(-0.37,-0.25) | -84.09(-96.54,-71.65) | -0.37(-0.43,-0.3) | 32.06(18.88,45.23) | 0.09(0.06-0.11) |
| 1998 | -104.78(-131.11,-78.46) | -0.12(-0.16,-0.09) | -52.71(-63.52,-41.9) | -0.31(-0.38,-0.25) | -84.03(-96.43,-71.63) | -0.37(-0.43,-0.31) | 31.96(18.76,45.16) | 0.08(0.06-0.11) |
| 1999 | -103.53(-129.75,-77.3) | -0.12(-0.16,-0.09) | -52.36(-63.03,-41.69) | -0.31(-0.38,-0.25) | -83.31(-95.77,-70.84) | -0.37(-0.43,-0.31) | 32.14(18.91,45.36) | 0.09(0.06-0.12) |
| 2000 | -104.23(-130.41,-78.05) | -0.13(-0.16,-0.1) | -52.81(-63.34,-42.29) | -0.31(-0.38,-0.25) | -83.72(-96.18,-71.27) | -0.38(-0.44,-0.32) | 32.31(19.01,45.61) | 0.09(0.06-0.12) |
| 2001 | -104.49(-130.44,-78.53) | -0.13(-0.16,-0.1) | -52.69(-62.91,-42.47) | -0.31(-0.37,-0.24) | -83.94(-96.32,-71.56) | -0.38(-0.44,-0.32) | 32.14(18.78,45.5) | 0.09(0.06-0.11) |
| 2002 | -103.54(-129.38,-77.71) | -0.13(-0.16,-0.1) | -51.74(-61.96,-41.53) | -0.31(-0.37,-0.24) | -84.16(-96.44,-71.88) | -0.38(-0.44,-0.32) | 32.36(18.96,45.77) | 0.09(0.06-0.11) |
| 2003 | -101.93(-127.68,-76.18) | -0.13(-0.16,-0.1) | -50.17(-60.45,-39.89) | -0.31(-0.37,-0.24) | -84.22(-96.37,-72.07) | -0.38(-0.44,-0.32) | 32.46(19.03,45.89) | 0.09(0.06-0.11) |
| 2004 | -102.16(-127.74,-76.59) | -0.13(-0.16,-0.1) | -50.37(-60.45,-40.3) | -0.31(-0.38,-0.25) | -83.87(-95.86,-71.88) | -0.38(-0.44,-0.32) | 32.08(18.64,45.51) | 0.08(0.06-0.11) |
| 2005 | -101.37(-126.9,-75.83) | -0.13(-0.16,-0.1) | -50.14(-60.2,-40.09) | -0.31(-0.37,-0.24) | -83.81(-95.72,-71.9) | -0.38(-0.44,-0.32) | 32.59(19.14,46.04) | 0.08(0.06-0.11) |
| 2006 | -100.83(-126.25,-75.41) | -0.13(-0.16,-0.1) | -49.63(-59.71,-39.55) | -0.31(-0.37,-0.24) | -83.61(-95.41,-71.81) | -0.38(-0.43,-0.32) | 32.41(18.94,45.88) | 0.08(0.05-0.11) |
| 2007 | -99.39(-124.7,-74.08) | -0.13(-0.16,-0.1) | -49.15(-59.21,-39.1) | -0.3(-0.37,-0.24) | -83.04(-94.74,-71.33) | -0.38(-0.43,-0.32) | 32.79(19.35,46.24) | 0.08(0.05-0.11) |
| 2008 | -98.07(-123.23,-72.91) | -0.12(-0.15,-0.1) | -48.55(-58.44,-38.66) | -0.3(-0.37,-0.24) | -82.59(-94.2,-70.98) | -0.38(-0.43,-0.32) | 33.07(19.62,46.53) | 0.08(0.05-0.11) |
| 2009 | -96.59(-121.5,-71.68) | -0.12(-0.15,-0.09) | -47.46(-57.15,-37.78) | -0.3(-0.37,-0.24) | -82.52(-94.02,-71.01) | -0.38(-0.43,-0.32) | 33.39(19.92,46.85) | 0.08(0.05-0.11) |
| 2010 | -95.75(-120.48,-71.01) | -0.12(-0.15,-0.09) | -46.72(-56.19,-37.26) | -0.31(-0.37,-0.24) | -82.87(-94.29,-71.45) | -0.38(-0.43,-0.33) | 33.85(20.34,47.36) | 0.08(0.05-0.11) |
| 2011 | -93.13(-117.69,-68.56) | -0.12(-0.15,-0.09) | -45.38(-54.74,-36.02) | -0.31(-0.37,-0.25) | -82.24(-93.6,-70.87) | -0.38(-0.43,-0.33) | 34.49(21.04,47.94) | 0.08(0.05-0.11) |
| 2012 | -90.46(-114.84,-66.08) | -0.12(-0.15,-0.09) | -44(-53.15,-34.86) | -0.3(-0.36,-0.24) | -81.44(-92.8,-70.08) | -0.38(-0.43,-0.32) | 34.98(21.56,48.4) | 0.08(0.05-0.11) |
| 2013 | -88.52(-112.63,-64.41) | -0.12(-0.15,-0.09) | -43.06(-52.08,-34.04) | -0.3(-0.36,-0.24) | -80.6(-91.94,-69.27) | -0.38(-0.43,-0.32) | 35.14(21.76,48.52) | 0.08(0.05-0.11) |
| 2014 | -86.05(-109.97,-62.14) | -0.12(-0.15,-0.09) | -41.77(-50.68,-32.87) | -0.3(-0.36,-0.24) | -79.64(-90.95,-68.34) | -0.38(-0.43,-0.32) | 35.36(22.06,48.66) | 0.08(0.05-0.1) |
| 2015 | -84.01(-107.73,-60.29) | -0.12(-0.15,-0.09) | -40.67(-49.44,-31.91) | -0.3(-0.35,-0.24) | -78.77(-90.04,-67.5) | -0.38(-0.43,-0.32) | 35.44(22.18,48.7) | 0.08(0.05-0.1) |
| 2016 | -82.07(-105.47,-58.67) | -0.12(-0.14,-0.09) | -39.87(-48.38,-31.36) | -0.3(-0.35,-0.24) | -77.75(-88.9,-66.6) | -0.38(-0.43,-0.33) | 35.55(22.35,48.74) | 0.08(0.05-0.1) |
| 2017 | -78.59(-101.81,-55.36) | -0.11(-0.14,-0.09) | -38.14(-46.54,-29.74) | -0.29(-0.35,-0.24) | -76.64(-87.68,-65.6) | -0.38(-0.43,-0.33) | 36.19(23.03,49.35) | 0.08(0.05-0.1) |
| 2018 | -75.46(-98.39,-52.54) | -0.11(-0.14,-0.08) | -36.55(-44.65,-28.45) | -0.29(-0.35,-0.24) | -75.43(-86.38,-64.49) | -0.38(-0.43,-0.33) | 36.52(23.37,49.68) | 0.08(0.05-0.1) |
| 2019 | -72.82(-95.45,-50.19) | -0.11(-0.14,-0.08) | -34.98(-42.83,-27.14) | -0.29(-0.35,-0.23) | -74.35(-85.2,-63.49) | -0.38(-0.43,-0.32) | 36.51(23.35,49.67) | 0.08(0.05-0.1) |
| 2020 | -69.43(-91.98,-46.88) | -0.11(-0.13,-0.08) | -32.6(-40.78,-24.43) | -0.3(-0.36,-0.25) | -73.35(-84.14,-62.56) | -0.38(-0.43,-0.32) | 36.52(23.35,49.7) | 0.08(0.05-0.1) |
| 2021 | -68.8(-91.18,-46.41) | -0.11(-0.13,-0.08) | -30.9(-38.8,-23) | -0.3(-0.35,-0.24) | -73.73(-84.68,-62.79) | -0.38(-0.43,-0.33) | 35.84(22.66,49.02) | 0.08(0.05-0.1) |
