# Supplementary material for: Global burden and cross-country inequality of infectious skin diseases in children: the Global Burden of Disease Study 2021
Source: Int Health. 2025 Oct 14;18(3):480–9. doi: 10.1093/inthealth/ihaf109 (PMC13154834; doi:10.1093/inthealth/ihaf109)
Supplement: ihaf109_Supplemental_Files [file ihaf109_supplemental_files.zip › Figure S12.pdf]

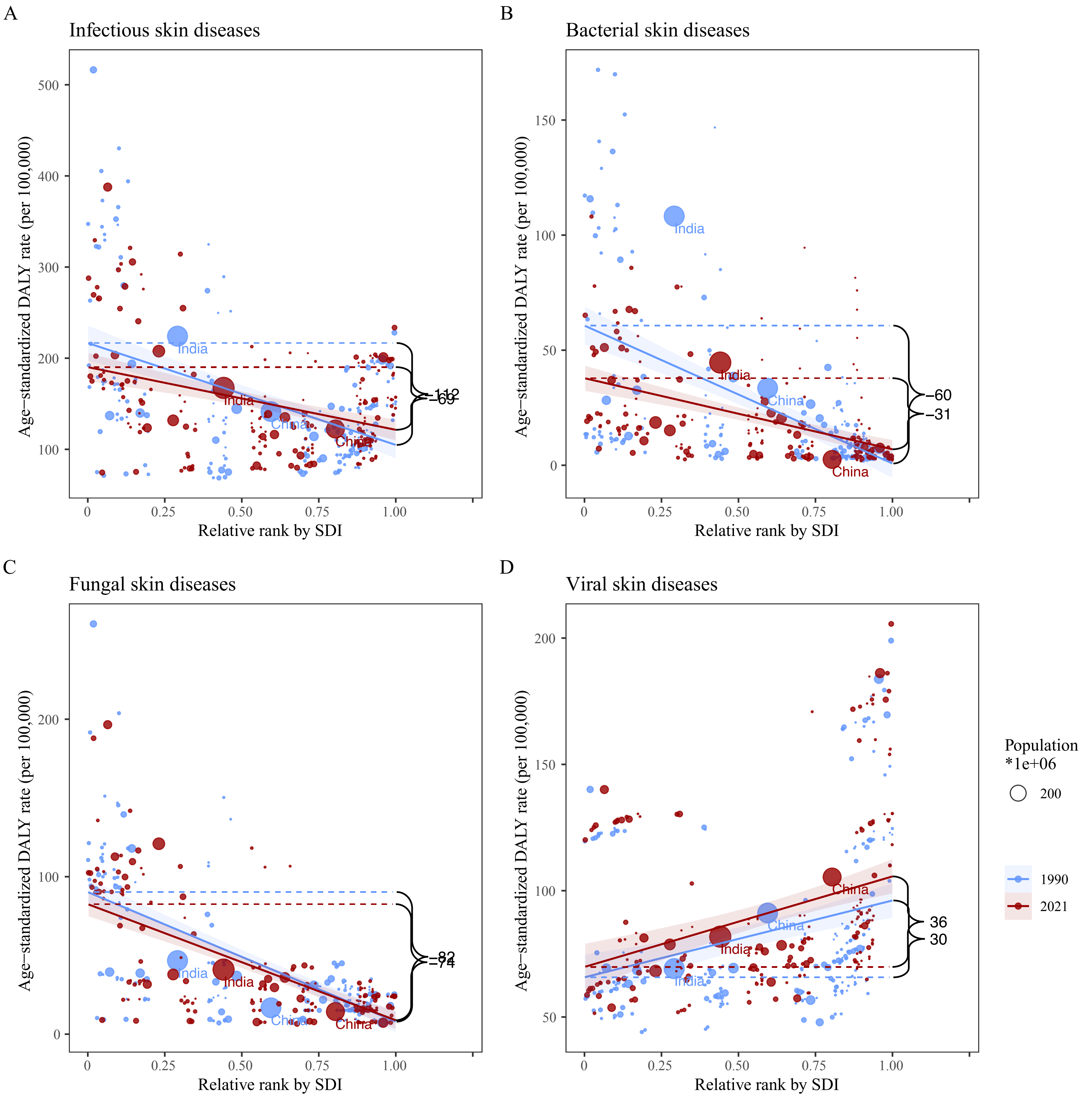
A
Infectious skin diseases
B
Bacterial skin diseases
C
Fungal skin diseases
D
Viral skin diseases
Age−standardized DALY rate (per 100,000)
Relative rank by SDI
India
China
−112
−69
−60
−31
−82
−74
36
30
Population *1e+06
200
1990
2021
