# Supplementary material for: Global burden and cross-country inequality of infectious skin diseases in children: the Global Burden of Disease Study 2021
Source: Int Health. 2025 Oct 14;18(3):480–9. doi: 10.1093/inthealth/ihaf109 (PMC13154834; doi:10.1093/inthealth/ihaf109)
Supplement: ihaf109_Supplemental_Files [file ihaf109_supplemental_files.zip › Figure S13.pdf]

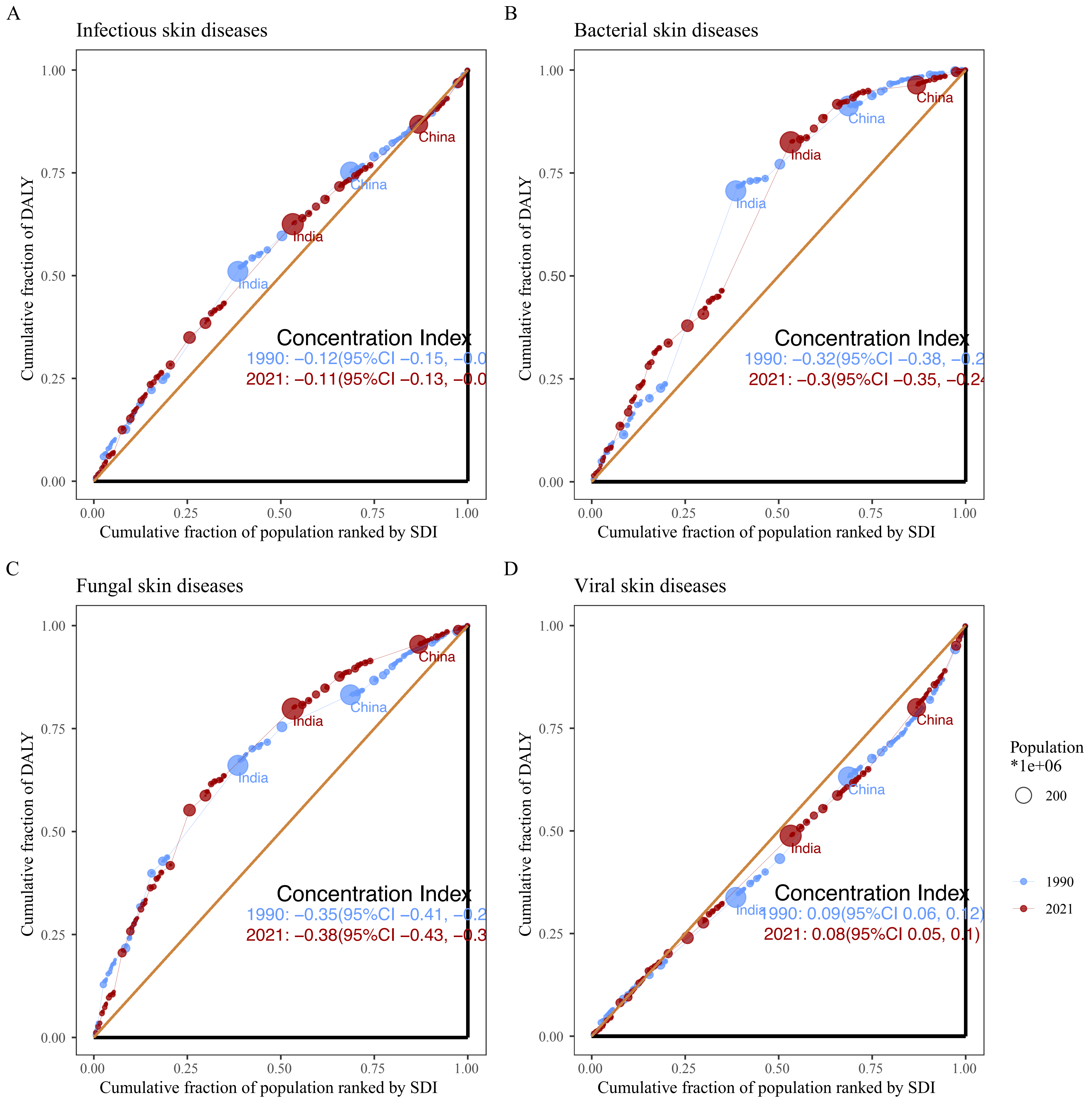
A
Infectious skin diseases
Cumulative fraction of DALY
Cumulative fraction of population ranked by SDI
China
India
Concentration Index
1990: −0.12(95%CI −0.15, −0.0
2021: −0.11(95%CI −0.13, −0.0
B
Bacterial skin diseases
Concentration Index
1990: −0.32(95%CI −0.38, −0.2
2021: −0.3(95%CI −0.35, −0.24
C
Fungal skin diseases
Concentration Index
1990: −0.35(95%CI −0.41, −0.2
2021: −0.38(95%CI −0.43, −0.3
D
Viral skin diseases
Concentration Index
1990: 0.09(95%CI 0.06, 0.12)
2021: 0.08(95%CI 0.05, 0.1)
Population *1e+06
200
1990
2021
